# Versatile Glycan Probes for Multiplatform Investigation of Glycan Interactions with Proteins, Viruses, and Cells

**DOI:** 10.1038/s41467-026-75206-2

**Published:** 2026-07-17

**Authors:** Jin Yu, Alexiane Decout, Jiayun Yang, Antonio Di Maio, Wengang Chai, W. Bruce Turnbull, Thilo Stehle, Steven H. Sacks, Thomas P. Peacock, Munir Iqbal, Ten Feizi, Yan Liu

**Affiliations:** 1 Glycosciences Laboratory, Department of Metabolism, Digestion and Reproduction, Faculty of Medicine, Imperial College London, London, UK; 2 Institute of Reproductive and Developmental Biology, Department of Metabolism, Digestion and Reproduction, Imperial College London, London, UK; 3 The Pirbright Institute, Woking, UK; 4 School of Chemistry and Astbury Centre for Structural Molecular Biology, University of Leeds, Leeds, UK; 5 Interfaculty Institute of Biochemistry, University of Tübingen, Tübingen, Germany; 6 Medical Research Council (MRC) Centre for Transplantation, King’s College London, London, UK; 7 Present Address: Division of Biomedical Sciences, Warwick Medical School, University of Warwick, Coventry, UK

**Keywords:** High-throughput screening, Glycoconjugates

## Abstract

Glycan-mediated interactions are vital to development, microbial colonisation, immune signalling, and cancer progression. Glycan microarrays have revolutionised glycobiology by enabling high-throughput analysis of these complex interactions, supported by techniques that reveal kinetics and dynamics in solution or at the cellular level. We introduce multifunctional glycan probes based on a tri-functional Fmoc-Amino-Azido (FAA) linker, enabling multi-platform investigation of glycan-mediated interactions. These FAA probes support glycan presentation on both covalent and non-covalent array platforms, allowing direct comparison of glycan recognition by diverse proteins. Notably, certain viral adhesins and immune lectins show a preference for the non-covalent platform. The azido group allows further functionalisation via ‘click chemistry’, enabling biotinylation for immobilisation on bio-layer interferometry biosensors for influenza virus binding, or fluorescent tagging for flow cytometry analysis of glycan-lectin interactions on cells. These versatile probes offer a unified platform for in-depth interrogation of glycan interactions using complementary approaches, with strong potential to advance glycan-based diagnostics and therapeutics.

## Abstract (web summary)

This study presents a versatile glycan probe strategy that enables consistent analysis of glycan interactions across multiple platforms, including microarrays, biophysical assays, and cell-based experiments.

## Introduction

Glycans are ubiquitously displayed at the surface of cells and their surroundings in all living organisms. They interact with numerous binding partners, endogenous and exogenous, and mediate many key biological processes, including cell-cell adhesion, cell signalling, intracellular trafficking and ‘two-way’ microbe-host interactions^1,2^. Viruses^3^, fungi^4^, bacteria^5^ and parasites^6^ often select and use host cell surface glycans for attachment at the initial stages of infection or colonisation. On the other hand, the host responds to infective agents through recognition of microbial glycans by lectin receptors of the innate immune system^7^ and antimicrobial antibodies. Elucidation of the specificities of the diverse glycan-binding systems is crucial for our understanding of the glycan-mediated biological recognition events and pathways.

To investigate glycan–protein interactions, various analytical techniques are employed. Whilst solution-based methods such as NMR and isothermal titration calorimetry (ITC) allow the use of native glycans, most other approaches require glycans to be derivatised and immobilised on appropriate surfaces to enhance the multivalency (cluster) effect and enable efficient detection of protein binding. Glycan microarray technologies for sequence-defined glycans, first introduced in 2002^8^, have gained momentum internationally and have revolutionised the molecular dissection of glycan-protein interactions in a miniaturised and high-throughput fashion^9–11^. Considerable advances have been made in the expansion of glycan libraries both through chemoenzymatic synthesis approaches^12–15^ and isolation and characterisation of glycan ligands from natural sources^8,16–18^. To enable glycans to be immobilised on microarray surfaces for binding studies, a variety of linkers or tags have been developed^19–22^. The glycans can be converted into lipid-linked probes, neoglycolipids (NGLs), and immobilised noncovalently on nitrocellulose-coated glass slides^9^, side-by-side with natural glycolipids. Alternatively, glycans can be covalently attached to microarray slides via various surface chemistries; among these the most popular are amine-terminating glycan derivatives immobilised on N-Hydroxysuccinimide (NHS) functionalised glass slides^23,24^.

Other techniques, such as surface plasmon resonance (SPR) and bio-layer interferometry (BLI) are widely employed to study glycan interactions, offering real-time, label free measurements of binding specificity, kinetics, and affinity with protein or viral analytes. Functionalised glycans or glycopolymers, such as biotinylated forms, are often used for immobilising glycans onto streptavidin-coated biosensor surface to achieve reliable and reproducible results. Fluorescently labelled glycans are also widely used as probes in various systems for studies of glycan interactions and dynamics both in solution, such as the fluorescence polarisation method^25^, or on cellular surfaces, using traditional flow cytometry or the emerging high-resolution imaging technique, Glyco-PAINT^26^.

**Fig. 1:**
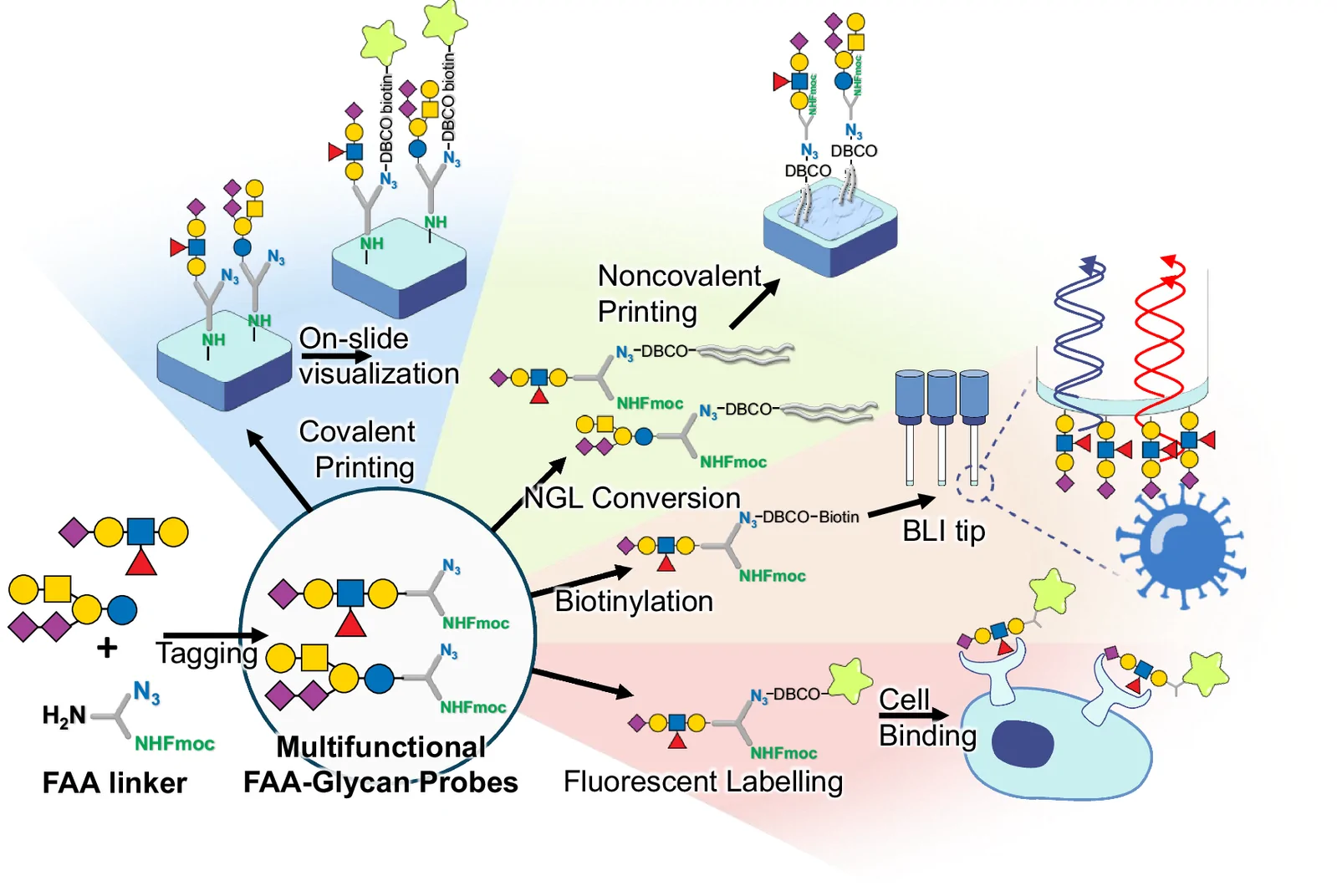
Design and applications of multifunctional FAA-linked glycan probes. FAA-linked glycan probes provide a versatile platform for glycan immobilisation, labelling, and presentation across a range of analytical, biophysical, and biological applications.

Developing a straightforward strategy to present a diverse range of glycan structures across various assay platforms is therefore ideal for achieving a comprehensive understanding of glycan-mediated interactions. In glycan derivatisation, factors such as the core monosaccharide status, the chemical properties of the linkers, and the density and mode of glycans display^27,28^ are known to influence glycan interaction readouts, making them important considerations in the glycan recognition knowledgebase. Here we present the design of a tri-functional, Fmoc-amino-azido (FAA), linker (Fig. 1), to allow efficient conversion of free reducing glycans into functional glycan probes. The FAA-linked glycan probes can be readily transformed into amino-terminating or lipid-tagged glycan probes for generating covalent and noncovalent arrays, respectively. This approach minimises potential confounding effects introduced by the linker itself, allowing direct comparisons between different array platforms. The versatility of the FAA-linked glycan probes was further demonstrated by their facile conversion into biotinylated glycan probes for influenza virus binding studies in the BLI system, as well as the incorporation of fluorescent tags to ‘light up’ the glycans for the interrogation of glycan-lectin interactions on intact cell surfaces via flow cytometry.

## Results

### Design of the FAA linker and preparation of FAA-linked glycan probes

**Fig. 2:**
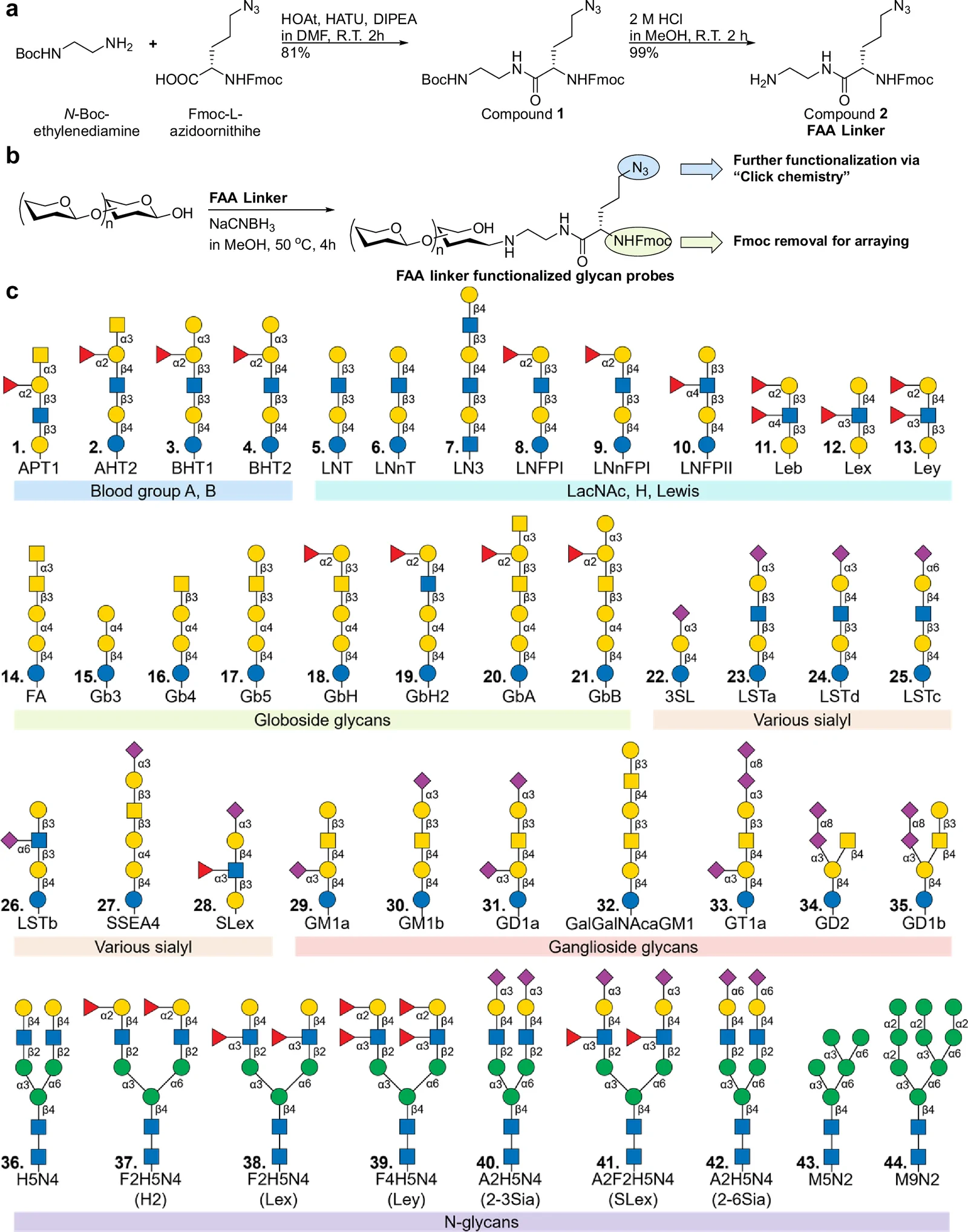
Schematic representation of the generation of the FAA linker, its conjugation to glycans and the symbolic sequences of the 44 FAA-linked glycan probes for arraying. **a** Synthesis of the FAA linker. **b** Glycan conjugation to FAA linker. **c** The 44 FAA-linked glycan probes used for glycan array construction comprised 39 probes prepared from reducing glycans and an additional 5 probes (#37–41) derived from probe #36 through enzymatic modification. Details of the FAA linker synthesis, glycan sources and sequences, conjugation procedures, and probe characterisation are provided in the SI.

The synthesis of the trifunctional linker was achieved by a two-step procedure using as a commercially available precursor, fluorenylmethyloxycarbonyl (Fmoc)-protected azido-containing amino acid, Fmoc-L-azidoornithine (Fig. 2a). The carboxyl group of the amino acid was activated and coupled to the amino group of N-Boc-ethylenediamine to give the Boc-protected amino-functionalised azidoornithine (compound **1**) as the single product in high yield. After purification it was deprotected by acid treatment to give the FAA linker, in quantitative yield. The FAA-linked glycan probes were prepared via microscale reductive amination (Fig. 2b). The efficiency of glycan conjugation was systemically evaluated using maltotriose (milligram scale), a cocktail of 6 oligosaccharide standards (low nmol scale) and oligosaccharide mixtures released from glycoproteins, as detailed in under Methods and Supplementary Information (SI), all achieving yields above 80%. The fluorescence sensitivity of the FAA glycan labelling method was compared with two widely used aromatic glycan labelling reagents 2-aminobenzamide (2AB)^29^ and 2-amino-N-(2-amino-ethyl)-benzamide (AEAB)^30^. The results demonstrated that similar HPLC profiles in terms of the retention times and peak shapes were observed (Supplementary Fig. 1), whereas FAA-linked glycans exhibited significantly higher sensitivity for fluorescent detection, aligning with previous studies on Fmoc-containing linkers^22^. The detection limit of the FAA-linked glycan was in the low pmol range, rendering the approach suitable for labelling natural glycans often available only in limited in amounts.

The optimised procedure (20 eq FAA linker, pH 6, 50 ^o^C, 4 h) was used to label a total of 39 reducing glycans with the FAA linker; these included different blood group antigens, A, B, H and Lewis (Le^*a*, *b*, *x*, *y*^) sequences, sialyl glycans, globosides, gangliosides and N-glycans (Fig. 2c). The FAA glycan probes can also readily serve as glycan acceptors in enzymatic reactions for further elongations or modifications. Starting from the asialo-biantennary N-glycan (probe #36), five additional N-glycan probes (#37-#41) with different modifications (sialylation and fucosylation) were enzymatically synthesised at 10–100 nmol scale. The hydrophobic Fmoc moiety facilitated chromatographic purification and monitoring of the synthetic products.

### On-array visualisation of immobilised FAA glycan probes

**Fig. 3:**
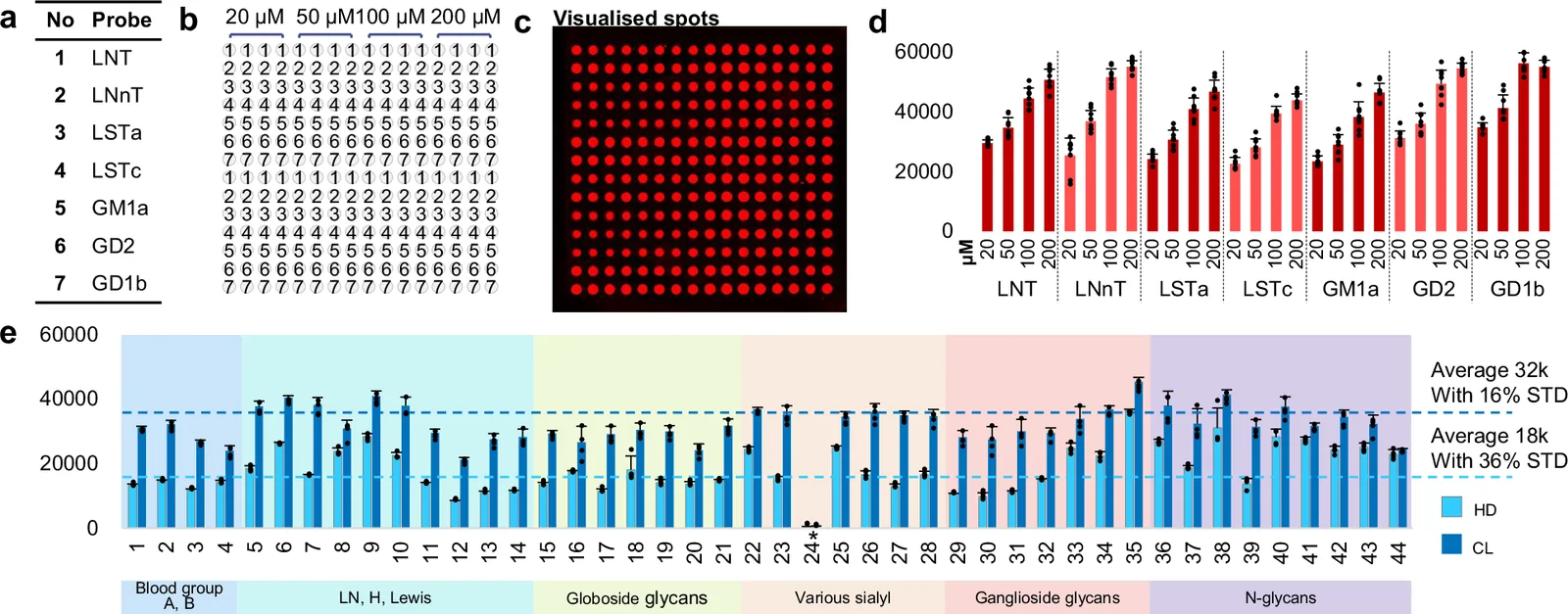
On-array visualisation and quantification of the arrayed FAA-linked glycan probes. **a** Seven FAA-linked glycan probes and their designations. The glycan sequences are included in Fig. 2c. **b** Printing layout of the seven FAA probes. The glycan probes were printed on CodeLink (CL) slides at four levels (20 μM to 200 μM) in two rows, with eight technical replicates per concentration, as indicated. **c** Representative array images of the seven FAA-linked probes following on-slide reaction with DBCO-biotin (250 μM), followed by detection with Alexa Fluor 647-conjugated streptavidin (1 μg/mL). **d** Averages of fluorescence intensities recorded for the seven arrayed probes after background subtraction. **e** Averages of quadruplicate spot fluorescence intensities of the 44 sequence-defined FAA probes (Fig. 2c) printed on normal density CL and high-density CodeLink (HD) slides, followed by overlay with DBCO-biotin (250 μM) and detection with Alexa Fluor 647-conjugated streptavidin (1 μg/mL). *Probe 24 was misprinted and did not pass quality control.

The Fmoc protecting group in the FAA linker can be readily removed to generate a primary alkyl amine for reacting with NHS-functionalised glass microarray slides. The presence of the azido functionality in the immobilised FAA glycan probe enables the introduction of biotin via the highly efficient copper-free click chemistry, strain-promoted azide-alkyne cycloaddition (SPAAC) reaction. Subsequent incubation with streptavidin-Alexa Fluor 647 allows visualisation and fluorescence measurement of the glycan probes on the array surface using a microarray scanner. Here a “dose–response” array of 7 FAA-glycan probes was generated to investigate the relationship between concentrations of printed probes (acidic and neutral) and the amounts detected on array after blocking and washing steps (Fig. 3). Four concentrations of each probe were printed and the unreacted NHS groups on the arrayed slide were blocked with ethanolamine. For 100 μM glycan the fluorescence signals were in the region of 40,000–50,000 units indicating that the relative amounts of glycans immobilised (Fig. 3d), whether acidic or neutral, varied by no more than 20%.

Covalent glycan arrays were prepared with the 44 FAA glycan probes shown in Fig. 2c following Fmoc deprotection and using the optimal probe printing concentrations determined above (100 μM). Two types of NHS-functionalised glass slides, CodeLink slides with normal NHS group density (CL) and high-density (HD) slides were used. According to the manufacturer, the HD slides possess a higher density of NHS groups potentially providing further insights into how glycan density on the covalent array surface influences glycan recognition. The immobilisation efficiency of glycan probes was assessed (Fig. 3e) and the readouts revealed that the various glycan probes were immobilised with a 16% variation on the CL slides and a 36% variation on HD slides. The same volume of printing solution was deposited onto the two types of slides side by side. This difference can be attributable to the distinct surface properties of the two types of slides: larger and more uniform spots were visualised on the CL slides, and more variable spot sizes on the HD slides. Of note is the printing of the probe LSTd (#24), appeared satisfactory according to the automatic optical dispense test of the arrayer instrument (see SI). However, the “on-array” visualisation method revealed that there was only a negligible amount of this probe was immobilised on the array surface (Fig. 3e). This information is annotated in the subsequent microarray binding results to avoid ‘false negative’ results of this probe in the covalent arrays. This highlights the advantage of having the post-printing “on-array” probe visualisation and quantitation step as part of the array quality control process.

### Cross-platform comparisons of FAA-based covalent and noncovalent glycan microarrays

**Fig. 4:**
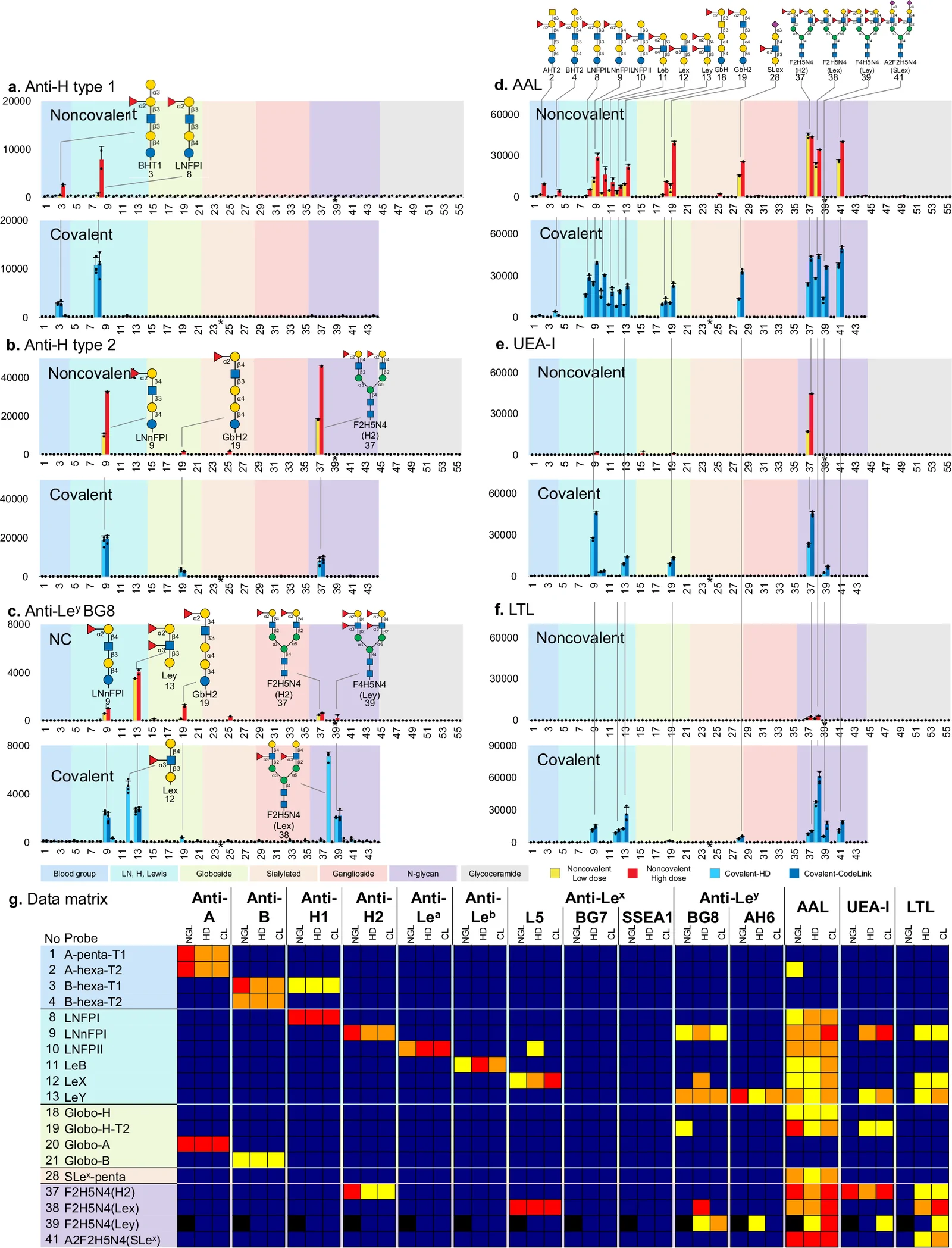
Glycan binding studies with sequence-defined noncovalent and covalent (CL and HD) arrays using antibodies to blood group A, B, H and Lewis type glycans and fucose-binding lectins. Histogram charts showing fluorescence intensities of binding of anti-H type 1 (**a**), anti-H type 2 (**b**), anti-Le^y^ BG8 (**c**), AAL (**d**), UEA-I (**e**), and LTL (**f**). Results are shown as fluorescence intensities at 2 and 5 fmol per spot printing levels for each probe on the noncovalent NGL arrays, calculated from averaged duplicate spot intensities at each printing level (error bars represent half of the difference between the two values), and of the averaged quadruplicate spot fluorescence intensities of each glycan probe in the covalent arrays (technical replicates, *n* = 4, with error bars representing the SD of the four values). **g** Heatmap comparing the relative binding intensities of fucosylated glycan probes with the 14 proteins analysed across three FAA array formats: noncovalent (5 fmol per spot level), HD and CL. For each protein, the highest signal intensity observed across all three array types was taken as 100%. Colour scale: red, >70%; orange, 30–70%; yellow, 10–30%; blue, <10%. *Black indicates probes that were misprinted and did not pass quality control: probe 24 on the covalent arrays and probe 39 on the noncovalent array.

**Fig. 5:**
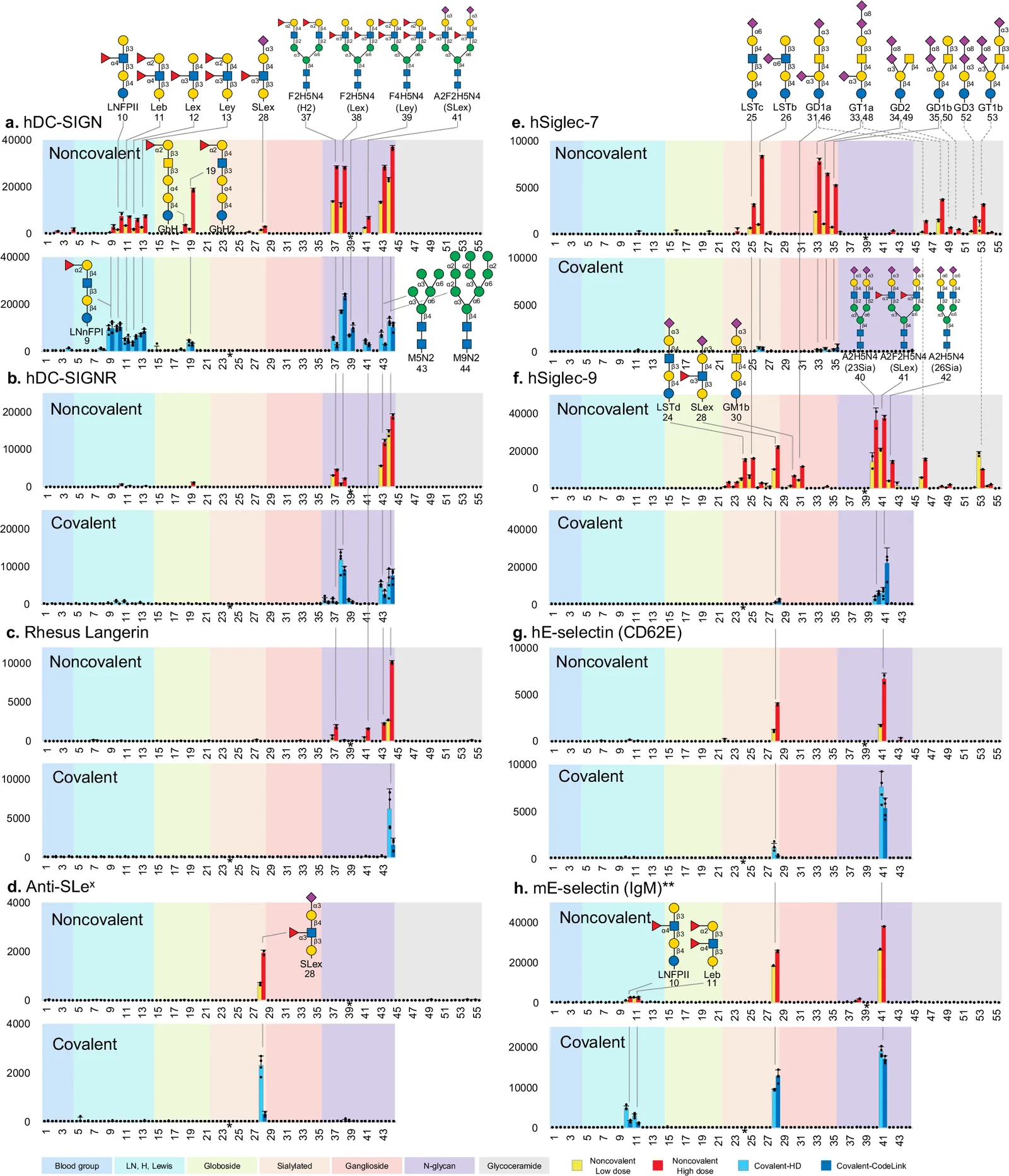
Distinct binding preferences of the receptors of the innate immune system observed in FAA glycan arrays. Microarray analysis of human DC-SIGN (**a**), human DC-SIGNR (**b**), rhesus Langerin (**c**), anti-SLe^x^ (**d**), human Siglec-7 (**e**), human Siglec-9 (**f**), human E-selectin (His-tagged) (**g**), and murine E-selectin (human IgM chimera) (**h**). Results are shown as fluorescence intensities at 2 and 5 fmol per spot printing levels for each probe on the noncovalent NGL arrays, calculated from averaged duplicate spot intensities at each printing level (error bars represent half of the difference between the two values), and of the averaged quadruplicate spot fluorescence intensities of each glycan probe in the covalent arrays (technical replicates, *n* = 4, with error bars representing the SD of the four values). *Probes that were misprinted and did not pass quality control: probe 24 on the covalent arrays and probe 39 on the noncovalent array.

**Fig. 6:**
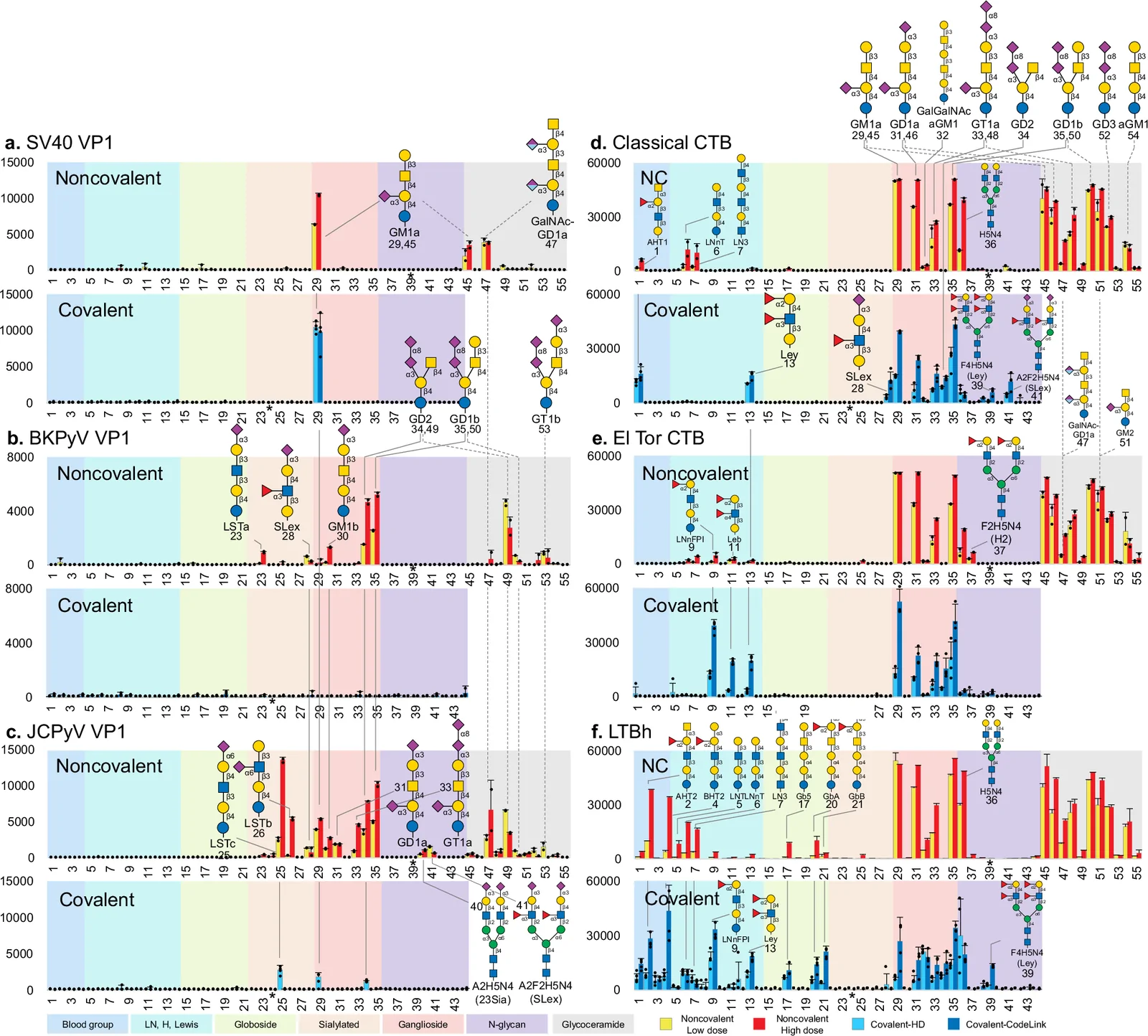
Preferential binding of polyomavirus VP1 proteins in the noncovalent arrays and different glycan binding characteristics revealed for bacterial toxins. Histogram charts showing fluorescence intensities of binding of His-tagged SV40 VP1 (**a**), BKPyV VP1 (**b**), JCPyV VP1 (**c**), classical CBT (**d**), EI Tor CTB (**e**) and LTBh (**f**). Results are shown as fluorescence intensities at 2 and 5 fmol per spot printing levels for each probe on the noncovalent NGL arrays, calculated from averaged duplicate spot intensities at each printing level (error bars represent half of the difference between the two values), and of the averaged quadruplicate spot fluorescence intensities of each glycan probe in the covalent arrays (technical replicates, *n* = 4, with error bars representing the SD of the four values). *Probes that were misprinted and did not pass quality control: probe 24 on the covalent arrays and probe 39 on the noncovalent array.

The azido group in the FAA-glycan probes allows their facile conversion into NGLs by a single SPAAC reaction step using the lipid reagent DBCO-DHPE (Supplementary Fig. 2). An aliquot (10-20 nmol) each of the 44 FAA glycan probes (Fig. 2c) were converted into the corresponding FAA-NGL probes. These were arrayed in a liposomal formulation with carrier lipids on to NC-coated glass slides using the established protocol^31^, alongside 11 natural glycolipids (glycosylceramides) as standards (designated “noncovalent NGL array set”, Supplementary Data 1). The construction of this array set allowed a well-designed comparative microarray study between the noncovalent NGL array and covalent glycan arrays on CL and HD slides, featuring a range of glycan recognition systems, including 14 anti-glycan antibodies, 9 plant lectins, 3 viral adhesins, 3 bacterial toxins, and 7 immune lectin-type receptors. The results are detailed in Figs. 4–6 and Supplementary Figs. 3-7. Results with different classes of glycan-binding proteins (GBPs) are described below, with more detailed information provided in the SI.

#### Recognition of fucosylated glycans by antibodies and lectins

Fucose-containing glycans are integral parts of the blood group antigen system and have important roles in cell recognition and signalling, tumour metastasis and host-pathogen interactions. Lectins and sequence-specific antibodies have long been used as histochemical tools as micro-sequencing reagents^32,33^. A panel of 11 blood group-related antibodies and 3 commonly used fucose-binding lectins, *Aleuria Aurantia* Lectin (AAL), *Ulex Europaeus* Agglutinin I (UEA-I) and *Lotus Tetragonolobus* Lectin (LTL), are among the lectins analysed here using the three types of FAA glycan arrays.

Overall, most of the antibodies analysed recognised their respective glycan antigens as predicted, with comparable signal strengths observed on the three types of arrays (Fig. 4). A few exceptions regarding antibody specificity are described in the SI. Worthy of note, in the covalent HD array only, weak binding of anti-L5 to Le^a^ probe LNFPII and anti-Le^y^ BG8 binding to the two Le^x^ probes (#12 and #38) were observed, suggesting that the high glycan density of the HD array increases the likelihood of detecting weak binders, as seen in earlier studies on anti-HIV antibody recognition^34^.

Among the fucose-specific lectins, AAL exhibited the broadest specificity for fucosylated glycans, with the exception of blood group A and B-related epitopes on the type 1 (Galβ1-3GlcNAc-) backbone, which were not recognised across all three array types (Fig. 4d). UEA-I and LTL gave better results in the covalent arrays. UEA-I, known for its specificity towards α1-2-fucosylated type 2 backbone glycans, indeed showed binding to α1-2-Fuc-terminating biantennary *N*-glycan in both covalent and noncovalent arrays (#37, Fig. 4e). In contrast, LNnFPI (#9), strongly bound by the anti-H type 2 antibody BE2 across all arrays (Fig. 4b), was only recognised by UEA-I on the covalent arrays. LTL, a lectin specific for α1-3-fucose, showed good binding to Le^x^ and Le^y^-related probes in the covalent arrays but not in the noncovalent NGL array (Fig. 4f) despite strong binding detected to Le^x^ and Le^y^ NGL probes with their respective antibodies respectively (Fig. 4g). LTL binding detected here to the sialyl Le^x^ (SLe^x^) on a pentasaccharide (#28) or the antennae of *N*-glycan (#41) and H type 2 probe LNnFPI (#9) has not been described^35^, thus adding new knowledge on the specificity of this lectin.

#### Glycan binding by lectin receptors of the innate immune system

Seven immune lectin receptors were investigated, including three closely related C-type lectin receptors (CLRs) DC-SIGN, DC-SIGNR, Langerin, and two siglecs, Siglec-7 and Siglec-9, as well as two E-selectin constructs. These are immune regulatory receptors playing important roles in immune cell signalling and modulation^36^.

With the CLRs, the signal-to-noise ratio was better in the noncovalent compared to the covalent arrays overall (Fig. 5a-c). All three CLRs bound to oligomannose N-glycans as predicted^37,38^, with human DC-SIGN also binding to H type 2 and Lewis (Le^a^, Le^b^, Le^x^ and Le^y^) probes^39^, whilst rhesus Langerin bound almost exclusively to Man_9_GlcNAc_2_ (weaker intensity in the CL array compare to the HD and noncovalent arrays). Notably, hDC-SIGNR showed additional binding to the Le^x^-N-glycan, which had not been reported previously.

The two human siglecs showed binding signals mainly in the noncovalent arrays but not the covalent arrays (Fig. 5e, f). Siglec-7 showed binding to α2,6-sialyl LSTb (#26) and α2,8-sialylated GT1a, GD2, GD1b (#33-35) in agreement with previous findings^40–42^. These probes, along with LSTc (#25), were bound in the noncovalent NGL array but not in the covalent arrays. Siglec-9, known to bind to α2,3- and α2,6-linked sialyl sequences, showed binding to SLe^x^ *N*-glycan probe (#41), and α2,3- or α2,6-sialylated probes (#24-26) were mainly detected in the noncovalent array. This contrasts with the binding signals detected with the sialic acid-binding plant lectins SNA and MAA-I, which showed stronger binding in the covalent than the noncovalent arrays (Supplementary Fig. 5).

We analysed an IgM and a His-tagged construct of E-selectin, an adhesion molecule known to bind SLe^x^^43^, in comparison to an anti-SLe^x^ antibody (clone CSLEX1). E-selectin gave potent binding to both SLe^x^ terminating probes (#28, #41, Fig. 5h), whereas the antibody did not bind to the biantennary *N*-glycan probe (#41) across all arrays (Fig. 5d). This hindrance with the biantennary *N*-glycan probe is reminiscent of observation with a similar anti-SLe^x^ (anti-human CD15S, clone CHO131) in an earlier report^44^.

#### Sialyl glycan binding by polyomavirus VP1 proteins

The pentameric VP1 proteins are major capsid components of polyomaviruses and are known to mediate host cell attachment through highly specific sialyl glycan recognition. SV40 VP1 showed strong binding in all three arrays to its well-known ligand GM1a^45^, in the form of FAA glycan probe (#29) (Fig. 6a). In the noncovalent array, binding was also detected to natural glycolipid GM1a (#45), and the disialyl glycolipid GalNAc-GD1a (#47)^46^, the latter not included in the previous microarray study^45^. There was a striking difference of strong VP1 binding in the noncovalent and little or no binding in the covalent arrays for the other two VP1s (Fig. 6b, c). BKPyV VP1 bound mainly to the GD2 and GD1b probes, consistent with the previous study^47,48^. In an earlier NGL-based array^49^ the JCPyV VP1 protein was shown to bind exclusively to the L-shaped α2-6-sialyl sequence LSTc. Using the noncovalent FAA-NGL array with an improved liposomal formulation and protein construct, we detected binding to the LSTc probe (#25) with the strongest signal intensity, along with interactions with several other sialyl glycan probes, in particular gangliosides, GM1a, GD2 and GD1b. These findings are consistent with a comprehensive structural and functional study demonstrating the VP1s of different JCPyV strains can engage multiple gangliosides in addition to LSTc^50^.

#### Glycan recognition by bacterial toxins

Three structurally related toxins of the cholera toxin family: *Vibrio cholerae* Toxin Subunit B of the O1 classical biotype (Classical CTB, cCTB) and the El Tor biotype (El Tor CTB), and the enterotoxigenic *Escherichia coli* heat-labile Toxin (LTBh)^51^ were investigated here. Unlike the BKPyV and JCPyV VP1s, both covalent and noncovalent arrays exhibited robust binding (Fig. 6d-f). Although GM1a is known as the most potent ligand for cholera toxins^52^, when the toxins were overlaid at 5 μg/mL, binding signals for GM1a, GD1a, and GD1b were almost equally strong in the noncovalent arrays, followed by GT1a, GD3 and asialo-GM1. Binding to ganglioside oligosaccharides was also well detected in the covalent CL arrays but less strongly in the HD arrays, highlighting the influence of the ligand density or orientation for toxin binding. At 0.5 μg/mL toxin concentration (Supplementary Fig. 7), signal intensity in the covalent arrays decreased to approximately one-fourth of that in the noncovalent arrays, suggesting the noncovalent platform has higher sensitivity for studying toxin binding.

Our findings are in overall agreement with those from earlier reports determined by the microtiter well and SPR assays^53,54^. The strong binding observed with cCTB to GD1a in both covalent and noncovalent arrays comes as a surprise, given that previous crystallographic study demonstrated a tightly-bound unsubstituted terminal galactose in GM1a at the protein surface leaving no room for an extra sialic acid^55^. This implies that GD1a when presented multivalently on the arrays may engage with the receptor in a distinct orientation compared to GM1a. An observation of note is the binding to GD2 detectable in the covalent arrays (#34) but not in the noncovalent arrays (#34, #49).

In addition to ganglioside glycans, differential binding of the three toxins to blood group antigens was also observed, mainly in the covalent arrays. Details are provided in the SI and summarised as follows: cCTB bound to A type 1, Le^y^, and SLe^x^; El Tor CTB bound to H type 2, Le^y^, and Le^b^; and LTBh bound to A and B (type 2 more strongly than type 1), also A and B on globo-backbone, H type 2, and Le^y^. Thus, it appears that *E. coli* have evolved to produce toxins with differential preferences to blood group-related glycans and their backbones, the majority of which are found in the intestine.

### Biotinylated FAA glycan probes reveal highly sensitive detection of influenza virus binding via BLI

The FAA-linked glycans can be conveniently converted into biotinylated probes for BLI assays, simply through the attachment of a biotin moiety to the azido branch via SPAAC click chemistry. Here we demonstrate their application in studying binding by three live influenza viruses: A/Anhui/1/2013 (AH/13 H7N9), A/England/195/2009 (Eng/195 H1N1), and A/Chicken/Vietnam/OIE-2202/2012 (OIE H5N1). The biotinylated FAA-glycan probes were compared to commercially available biotinylated poly[N-(2-hydroxyethyl) acrylamide] (PAA) probes to assess their performance in virus binding in the BLI system.

**Fig. 7:**
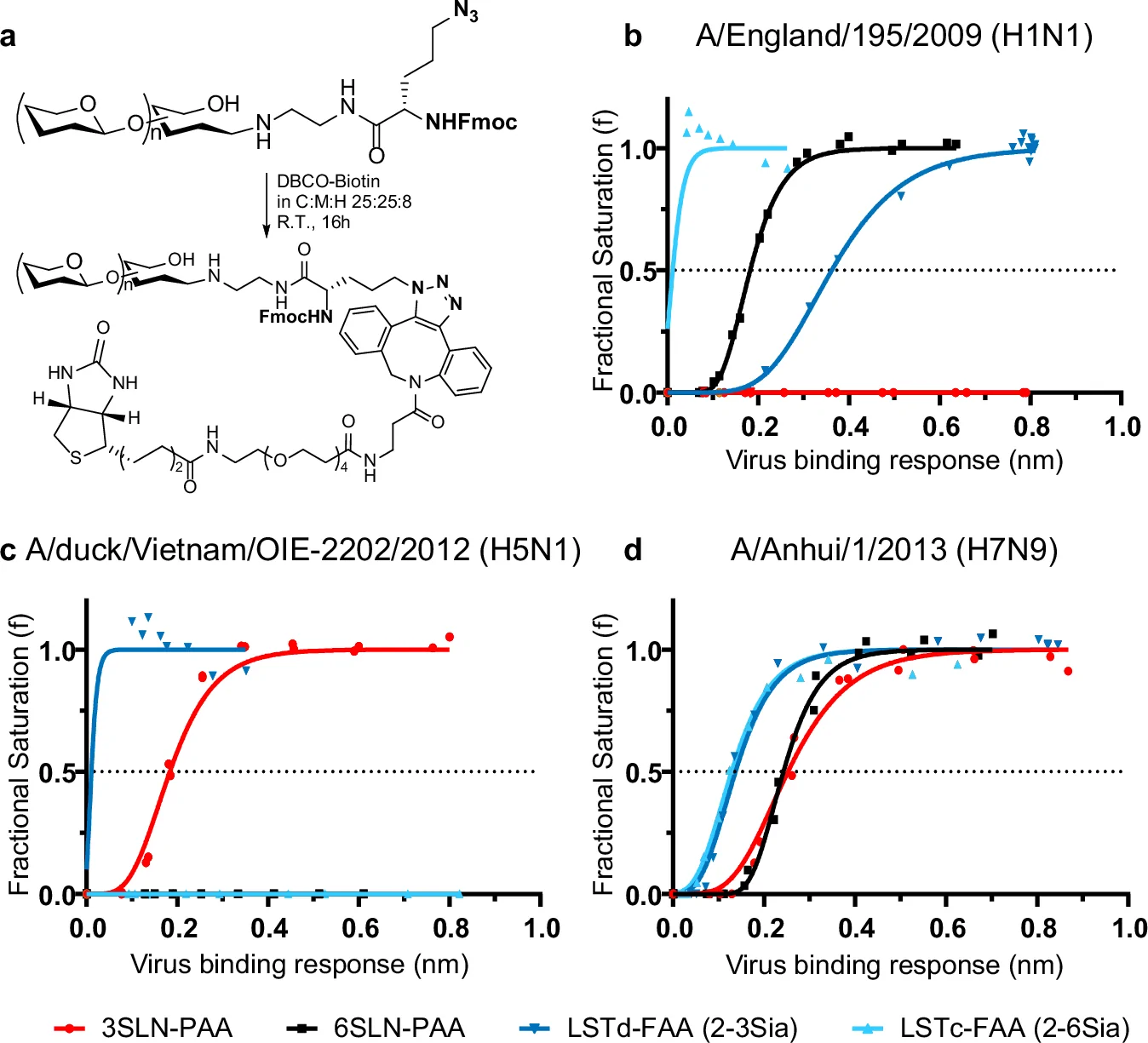
BLI analysis of influenza virus binding to biotinylated FAA glycans. **a** Scheme for biotinylation of FAA glycans by click chemistry. **b–d** Binding curve of three influenza viruses with biotinylated FAA probes (LSTd-FAA and LSTc FAA) compared with commercial biotinylated polyacrylamide (PAA) probes (3SLN-PAA and 6SLN-PAA). The x-axis represents the amount of glycan probe loaded onto the biosensor, measured as sensor response (µm). The y-axis shows the fractional saturation of virus binding normalised to the maximal binding plateau (maximum value = 1).

The results indicated that biotin-FAA sialyl glycan probes were generally more sensitive than the PAA-based probes of α-2,3-sialyl N-acetyllactosamine (3SLN) and 6SLN. H1N1 binding to LSTc-FAA and H5N1 binding to LSTd-FAA exhibited saturated signals at the lowest glycan probe loading concentration (11 nmol) in the first round of the analyses (Fig. 7). The receptor binding profiles of H5N1 (2-3 Sia specific) and H7N9 (binding to both 2-3 and 2-6 Sia) were consistent between PAA and FAA probes. In further analyses, the stronger interactions of the FAA sialyl glycan probes with the viruses, compared to the PAA probes, were reproducible across a wider glycan loading range, and the negative control of the two nonsialylated FAA probes (LNnT and Mal3) showed no detectable binding as expected (Supplementary Fig. 8). H1N1, which is known to bind 2-6 Sia, showed strong binding to 6SLN-PAA and LSTc-FAA as predicted, but also demonstrated additional weak binding to LSTd-FAA. This is likely due to the longer glycan backbone of the pentasaccharide LSTd-FAA probe compared to the commercial trisaccharide 3SLN-PAA, or potentially the higher density or more favourable orientated presentation of the FAA-linked glycans on the sensor chip. Further glycan microarray analysis of binding H1N1 virus also detected interactions with LSTd, though markedly weaker than LSTc (Supplementary Fig. 9), supporting these observations.

### Fluorescent FAA glycan probes illuminate Siglec-9-mediated cellular interactions

**Fig. 8:**
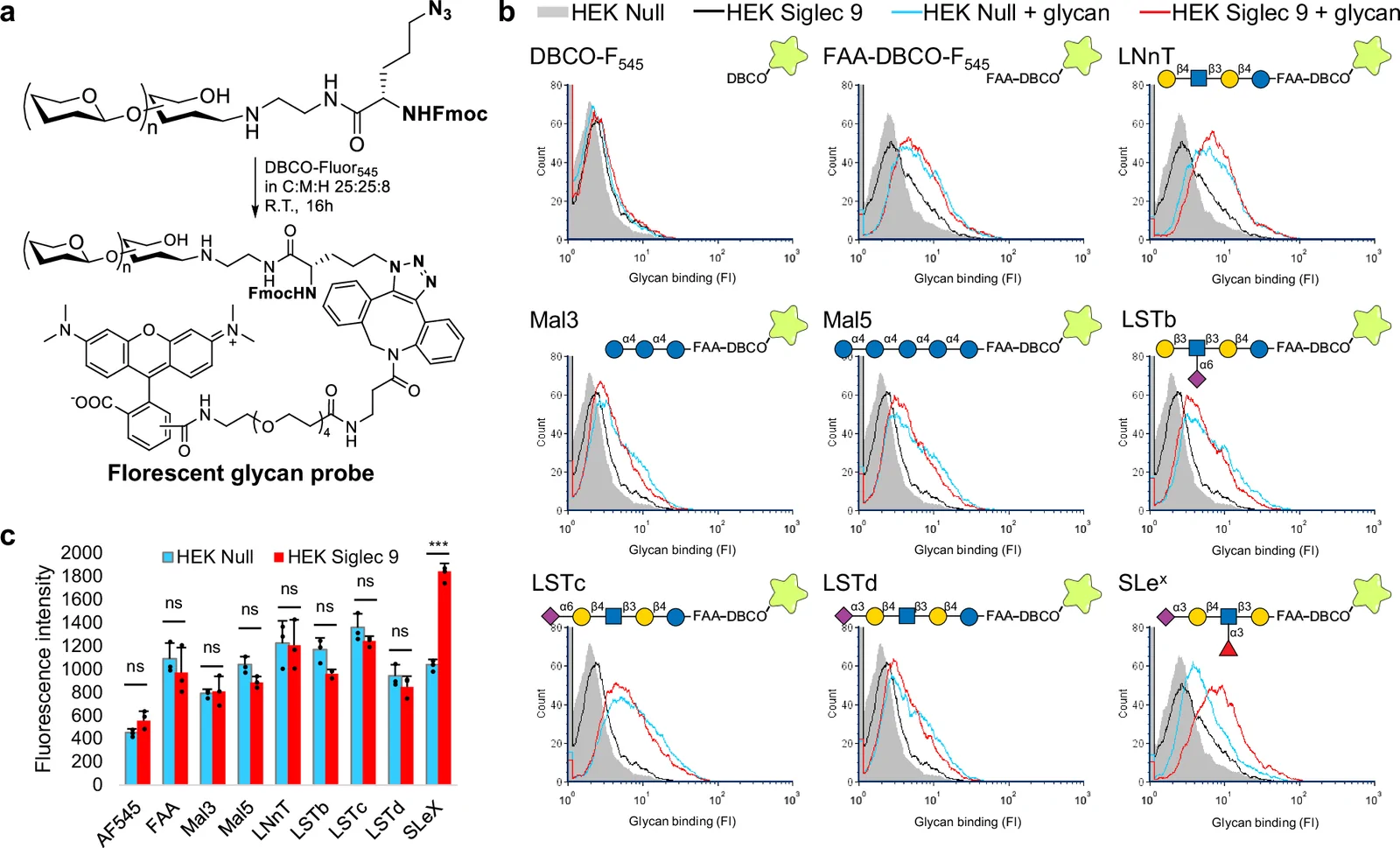
Analysis of HEK Siglec-9 cells incubated with fluorescently labelled FAA glycans. **a** Scheme for fluorescent labelling of FAA-tagged glycan probes. **b** Flow cytometry-based glycan binding profiles of HEK Siglec-9 and control cell line HEK Null1. **c** Fluorescence intensities of the HEK Siglec-9 and HEK Null1 cells incubated with the fluorescently labelled glycans in the microwell experiments (mean ± SD, *n* = 3 independent experiments, ***: *p* < 0.001). No statistical hypothesis testing was performed.

To further broaden the applications of FAA glycan probes, we have incorporated a fluorescent dye into the probes to render them suitable for fluorescence-based cell binding assays. By a simple SPAAC click reaction, the DBCO-PEG4-Fluor 545 (DBCO-F545) reagent was coupled with the azido group of the FAA glycan probes with high efficiency (Fig. 8a). This has allowed facile assembly of a panel of seven F545-FAA glycan probes: four sialyl (SLe^x^, LSTc, LSTd and LSTb) and three neutral (LNnT, Mal3, and Mal5). The preparation of these F545-labelled FAA probes and their characterisation are in the SI.

Cell binding analyses were performed with the fluorescent glycan probes using HEK cells transfected with Siglec-9, alongside HEK null1 cells as controls. The unconjugated dye reagent DBCO-F545 and the F545-labelled blank FAA linker (FAA-F545, without glycan) were also included as controls for the F545-FAA-glycan probes. The results of the flow cytometry analysis and the corresponding microwell-plate fluorescence readouts are in Fig. 8b, c. Whereas little or no interaction was observed with DBCO-F545, the FAA-F545 control showed some ‘background’ binding of comparable intensity to both HEK Null1 and HEK Siglec-9 cells. This binding is likely attributable to hydrophobic interactions stemming from the FAA linker.

Cell binding profiles similar to those of FAA-F545 tag were observed with the F545-FAA-glycans tested with the exception of the high affinity Siglec-9 ligand SLe^x^, with which a clear shift in fluorescence intensity was observed with the HEK Siglec-9 cells compared to the control HEK Null1 cells (Fig. 8b). The microwell assay gave results comparable to those of the flow cytometry read out, a significantly higher binding to the Siglec 9-expressing cells being observed only with the SLe^x^ probe (Fig. 8c). There was no shift in cell fluorescence intensity with the F545-FAA glycans LSTc and LSTd which are weaker ligands for Siglec-9 than SLe^x^, as observed in the binding experiments with Siglec-9 using noncovalent glycan arrays (Fig. 5f). The fluorescent glycans are in monomeric state in solution thus only with the high affinity ligand could be detected to the Siglec-9 expressing cells.

## Discussion

Over the past two decades, glycan microarrays have become indispensable tools in glycoscience, with technological advancements and increasing applications in laboratories worldwide. These technologies have greatly accelerated the acquisition of data on glycan-mediated interactions. However, interpreting data obtained from various array platforms is not always straightforward, as multiple variables exist throughout glycan microarray experiments^28^, potentially yielding differing results even with the same GBP. So far there are few reports on microarray platform comparisons in respect to binding signals elicited with a given glycan recognition system^56–59^, and such comparisons have been focused on data with plant lectins which typically give robust binding signals with arrayed glycan ligands. Comparative data on other endogenous and microbial glycan recognition systems are limited. Another limitation thus far in all the slide-based planar array platforms has been the lack of a means to monitor and measure, using microarray scanners, the amounts of the glycans immobilised on the array surface. These are parameters to note in the quality control of arrays generated. The ‘glycan array community’ is recognising the importance of adhering to the minimal information requirements outlined in the MIRAGE (minimum information required for a glycomics experiment) Guidelines for reporting glycan microarray analysis data^60^ to ensure the generation of interpretable data. Nonetheless, significant questions persist regarding the performance of different platforms raising the possibility of standardising glycan array methodologies.

To impart native glycans with maximised multifunctionality, the FAA linker was designed with several features in mind: i) efficient conjugation with minute amounts of glycans to give single anomeric products for resolving heterogeneous natural glycan populations, ‘glycomes’ of cells and tissues; ii) incorporation of a fluorescent group to assist the purification and quantitation of the derivatised glycans; iii) inclusion of an amino group to allow arraying onto NHS functionalised glass slides; iv) including an additional azido functionality to confer further versatility for conversion into other forms of functional glycan probes; these include for example lipid-linked, biotinylated, fluorescent forms that could be used in different settings for glycan recognition studies.

Systematic comparisons between the covalent and non-covalent array systems with the identical glycan derivatisation method have not been carried out so far. A focus of this study has been to closely compare the presentation of 44 FAA-linked glycans on the covalent and noncovalent arrays. The 36 GBPs tested were not intended as a comprehensive survey but rather as a representative set that includes cases where we anticipated potential differences between covalent and noncovalent arrays based on prior knowledge. The observation that roughly half exhibited comparable binding supports the robustness of both platforms while highlighting GBPs that require specific glycan presentation for recognition. Salient observations are summarised in Supplementary Table 1. Notably, LTL showed binding in the covalent arrays only, whereas Siglec-7, Siglec-9, and the VP1 proteins of JCPyV and BKPyV showed binding primarily on the noncovalent arrays only. Subtle but significant differences were observed with the remaining GBPs tested, including the three structurally related bacterial toxins that showed similarly strong binding to ganglioside-related probes in both arrays but distinct binding preferences with the low-affinity glycan ligands such as blood group and galactose-terminating sequences.

The preference for glycan ligands across different array platforms is likely influenced by the intrinsic affinity of the interaction, as well as specific requirements for ligand density and presentation that are critical for engagement by the multivalent binding sites of GBPs. The accessibility and flexibility of a given glycan ligand immobilised covalently or noncovalently on the microarray surface, together with possible linker effects, may differ. These factors can influence how effectively the ligand binds to its cognate GBP. The same principles apply in physiologic settings, where the accessibility of glycan ligands depends on their spatial presentation on specific protein carriers or the lipid compositions of glycolipids on membranes. Moreover, close evaluation of ligand density requirements is sometimes called for as there are instances of preferential binding to ligands at low density^61^. The side-by-side comparison of covalent arrays on CL and HD slides further illustrates how glycan density influences binding patterns, once again emphasising its importance in interpreting glycan array data.

The experience gained from the present study indicates that, for evaluating glycan binding by GBPs with lower affinities, where multivalent engagement is crucial for detectable binding, the noncovalent NGL-based array platform may offer advantages. However, covalent arrays on glass surfaces can be superior for analysing the binding of fluorescently labelled proteins or microbes, such as whole bacterial cells^62^, owing to their lower background compared with the nitrocellulose-coated surfaces used in noncovalent arrays. Thus, the comparisons presented here are not intended to assess the superiority of one platform over another, but rather to highlight their complementary nature. A lack of binding in one array platform does not necessarily indicate that a protein is unable to recognise glycans. Our aim is to draw attention to the importance of glycan presentation, minimise the likelihood of ‘false negative’ results in glycan array analyses, and promote flexibility across different technological platforms to enhance confidence in the knowledge gained on glycan-mediated interactions.

When determining the glycan specificity of a novel GBP, collecting data from both covalent and noncovalent array platforms provides a more comprehensive and reliable definition of binding preferences. A recent example is the characterisation of the melon lectin CMA1, whose binding specificity was elucidated by integrating datasets from the Consortium for Functional Glycomics covalent array and our NGL-based non-covalent array platform^63^.

The ease of converting FAA-linked glycans into biotinylated or fluorescent probes makes them highly versatile tools for glycan interaction studies beyond microarray screening. This approach provides access to biotinylated glycan probes that are not commercially available, facilitating their use in BLI (or SPR) assays for kinetic analyses of protein and virus binding partners across a wide range of glycan ligands. The high sensitivity of biotinylated FAA-glycan probes allows their use in minimal quantities, in the picomolar range, for BLI analyses of live, intact influenza viruses, offering promising opportunities to advance the discovery of novel receptor-binding specificities in emerging influenza strains, including those with pandemic potential. In parallel, fluorescent glycan probes can be directly applied in cell-binding assays. Our proof-of-concept experiments demonstrated distinct binding signals between Siglec-9-expressing cells and the fluorescently labelled high-affinity ligand SLe^x^-FAA probe. These fluorescent glycan probes serve as a flexible platform for analysing glycan-GBP interactions on cell surfaces using flow cytometry, microscopy, or plate-based screening assays. Beyond current applications, fluorescent glycan probes hold significant potential for emerging technologies such as Glyco-PAINT^26^, which enables high-resolution spatial mapping of glycan–protein interactions at the single-molecule level on cellular surfaces.

Together, these applications highlight the broad utility of FAA-linked glycan probes as powerful, adaptable tools for advancing glycoscience across virology, immunology, and cellular glycan biology, offering a broadly accessible and scalable toolkit to enhance current methodologies for investigating the glycan interactome.

## Methods

General methods and chemical reagents used are provided in SI under Supplementary Methods.

### Glycan materials

#### Sequence-defined glycans and glycosylceramides

Thirty-nine sequence-defined glycans were purchased from Elicityl (Crolles, France) and Dextra (Reading, UK), as indicated in Supplementary Table 2. The glycan sequences are given in Fig. 2 and Supplementary Data 2. In addition, the eleven glycosylceramide standards were from the collection of Glycosciences Laboratory, their sequences are given in Supplementary Data 1.

#### Glycans released from glycoproteins

Ribonuclease B (RNaseB) was purchased from TCI chemicals (Tokyo, Japan). Fetuin was from Merck. Endoglycosidase H (EndoH) and peptide N-glycanse F (PNGF) were from New England Biolabs (Manchester, UK). Oligomannose N-glycans were released from RNaseB (40 mg) using EndoH, and complex type N-glycans from fetuin (40 mg) using PNGF according to the recommended procedures from the manufacturers. 2-6-Sialylated biantennary N-glycan was released from sialylglycopeptides (TCI chemicals, Tokyo, Japan) using PNGF. The released glycans were purified using C18 and porous graphitised carbon (PGC) cartridges following previously published procedures^64^.

### Generation of multifunctional glycan probes

The FAA linker was chemically synthesised in a two-step procedure using Fmoc-L-azidoornithine and N-Boc-ethylenediamine (Supplementary Fig. 2). Detailed synthetic procedures and characterisation data for both the intermediate and final FAA linker are provided in SI under Supplementary Methods.

The efficiency of glycan conjugation was initially evaluated using maltotriose at the milligram scale. The starting glycans were fully labelled within 4 h using 2 equivalents of the FAA linker as shown by TLC. A glycan standard mixture of 6 oligosaccharides (3 nmol of each glycan) was labelled using 15 equivalents of FAA reagent at pH 6 in anhydrous methanol (Supplementary Fig. 2a). Labelling yields were quantified based on the fluorescence intensity of the product on HPLC. Almost full conversion of the starting glycans was achieved, regardless of sizes, neutral or acidic features, into the FAA-linked glycan probes.

For labelling oligosaccharide mixtures released from glycoproteins which have the less reactive HexNAc at the reducing end, the reaction conditions were further optimised. The final protocol involved dissolving lyophilised glycans in a solution of 0.18 M FAA linker and 0.5 M NaCNBH₃ in anhydrous methanol. Typically, 20 eq FAA linker was used relative to the glycans (1 nmol to 10 µmol). Where the glycans were in trace amounts, a minimum of 10 μL of solution was required. The pH of the solution was adjusted to 6 using a solution of 0.1% w/v NaOMe in MeOH and the reaction mixture was vortexed at 50 °C on a ThermoMixer (Eppendorf ^TM^) for 4 h. Thereafter, the excess reagents were removed using a cellulose cartridge. For a typical glycan (200 nmol) labeling using 1.7 mg of FAA linker, a cellulose cartridge with a 50 μL bed volume was used. The cartridge was first activated by washing sequentially with 0.3 ml of MeOH, followed by 0.3 mL of MeCN, followed by 0.3 mL of 0.1% TFA in H_2_O, and then equilibrated with 90% MeCN in H_2_O. The reaction mixture was diluted with 10 volumes of MeCN and loaded onto the cartridge. The cartridge was then washed with 1 ml of 90% MeCN in H_2_O to remove excess reagents, and the FAA-glycan probes were collected by eluting with 0.5 mL 0.1% TFA in H_2_O, concentrated and subjected to normal-phase (NP) HPLC (amide column) for further purification.

For generation of glycan microarrays, the 39 sequence-defined glycans (Supplementary Table 2) were labelled with FAA. Additionally, five N-glycan probes were synthesised through enzymatic modification using FAA-linked probe 36 as the substrate. Detailed procedures for the enzymatic synthesis are provided in the SI. The HPLC profiles and MS spectra corresponding to all 44 glycan probes are in Supplementary Fig. 15. All 44 probes were used for array generation.

### Generation of glycan probes for arrays

The FAA-tagged glycan probes can be printed on NHS-coated covalent array after removing the Fmoc groups. By exploiting the presence of the azido functional group, the FAA-linked glycan probes were converted to NGLs by a single SPAAC reaction step using the lipid reagent DBCO-DHPE (Supplementary Fig. 2c). An aliquot (10–20 nmol) each of the 44 FAA glycan probes (Fig. 2c) was converted into the corresponding FAA-NGL probes (Supplementary Fig. 2f), which were purified by HPLC, and were quantified based on their lipid content^31,65^. The detailed procedures used for Fmoc deprotection and NGL synthesis together with characterisation data are described in the SI.

### Glycan microarray analyses

Covalent glycan arrays were prepared with the 44 FAA glycan probes (Fig. 2c) on two types of NHS-functionalised glass slides, CodeLink (now known as TRIDIA) slides with normal NHS group density and the high-density Codelink slides (CL and HD slides, respectively). The noncovalent array was prepared in parallel using the NGL form of these 44 FAA glycan probes (Fig. 2c) including 11 natural glycolipid (glycosylceramide) standards using established procedure^31^. Details on the arraying instrument, printing conditions, array layout, imaging and data analysis are in the MIRAGE document (Supplementary Table 8). The list of 36 glycan-binding proteins investigated and assay conditions used in the cross-platform comparison microarray analyses are in Supplementary Tables 3–7. Fluorescence intensities and heat maps of the relative binding strengths of glycans with all the GBPs investigated are in the Supplementary Data 3 and 4. Highlights of glycan binding readouts from the covalent and noncovalent arrays are in Supplementary Table 1.

### Generation of biotinylated FAA glycan probes

Four biotinylated glycan probes were prepared from FAA-linked Maltotriose (Mal3), LNnT, LSTc and LSTd (Supplementary Fig. 2d). In brief, 5 nmol of the FAA-linked glycans were dissolved in 40 μL of a CHCl_3_:MeOH:100 mM ammonium acetate in H_2_O (25:25:8, v/v) mixture containing 20 nmol of DBCO-PEG4-Biotin. The reactions were kept at R.T. for 16 h and MALDI-MS indicated full conversion of the starting material. The products were subsequently purified by NP HPLC (amide column), giving four biotinylated FAA probes: LSTc, LSTd, LNnT, and Mal3. MS spectra of these glycan probes are shown in Supplementary Fig. 17.

### Virus purification and quantification

A/Chicken/Vietnam/OIE-2202/2012 (H5N1) and A/Anhui/1/2013 (H7N9) have been generated by reverse genetics as previously described^66^. A/England/195/2009 (H1N1) was gifted by Professor Wendy S. Barclay. H7N9 and H5N1 viruses were propagated in 9–11 day-old embryonated chicken embryos. H1N1 virus was propagated in MDCK cells. The viruses were then purified by ultracentrifugation at 27,000 rpm for 2 h. Virus pellets were then purified through a 30–60% sucrose gradient for 2 h at 4 °C. Purified viruses were then quantified by enzyme-linked immunosorbent assay (ELISA) as previously described by comparing to viral nucleoprotein detection to a known standard^67^.

### BLI assays using the influenza virus

Avian-like sugar analogues 3SLN poly[N-(2-hydroxyethyl) acrylamide] (PAA) conjugate and human-like sugar analogue 6SLN PAA conjugates (20% mol saccharide, 5% mol biotin) were from GlycoNZ and were compared with the two biotinylated 2,3/6 sialylated FAA probes (LSTd, LSTc). Biotinylated FAA probes of LNnT and Mal3 were included as non-sialylated controls. Receptor binding was assessed using BLI methods essentially as previously described^68^. In brief, purified viruses containing 100 pmol of nucleoprotein were diluted with 70 μL of HBS-EP buffer (TEKnova) containing 10 μM oseltamivir carboxylate (Roche) and 10 μM zanamivir (GSK) as virus neuraminidase inhibitors. The receptor binding against each receptor analogue was tested with Octet® R8 system (Sartorius) run at 5 Hz using streptavidin biosensors (Sartorius). Both PAA and FAA glycan probes were prepared as a 1.75-fold serial dilution across eight concentrations ranging from 532 nM to 11 nM for Fig. 7, or as a threefold dilution series ranging from 266 nM to 0.1 nM for Supplementary Fig. 8. Biotinylated probes were exposed to the streptavidin biosensors for between 30 and 600 s (determine by a pilot run) to maximise the datapoints across the curve. Assays were run at 30 °C throughout. Virus binding affinity was normalised to fractional saturation and the concentrations of sugar loadings as previously described using the bespoke programmes ‘Octet 1’ and ‘Octet 3’ generated by Stephen R. Martin^68^. Curves were fit using Graphpad Prism using an asymmetric sigmoidal 5PL curve.

### Generation of fluorescent FAA glycan probes

Seven FAA-lniked glycans were labelled with florescence (Supplementary Fig. 2e), and these included LSTb, LSTc, LSTd, LNnT, Mal3, Mal5 and SLe^x^. In brief, 5 nmol of the FAA-linked glycans were dissolved in 40 μL of a CHCl_3_:MeOH:100 mM ammonium acetate in H2O (25:25:8) mixture containing 20 nmol of DBCO-PEG4-Flour545. The reactions were kept at R.T. for 16 h and MALDI-MS indicated full conversion of the starting material. The solvent was removed under reduced pressure and the reaction residue was used directly without purification in cell incubation experiments. MS spectra of DBCO-PEG4-Flour545 modified glycan probes are in Supplementary Fig. 18.

### Mammalian cell culture and generation of HEK Siglec-9 cells

HEK Null1 cells were obtained from InvivoGen and were cultured and maintained under standard conditions in DMEM medium (Sigma-Aldrich) containing 10% decomplemented foetal bovine serum (Sigma-Aldrich) and 1% penicillin–streptomycin (Sigma-Aldrich). The cells were cultured at 37 °C and 5% CO_2_ in a humidified incubator, were free of mycoplasma and routinely tested using the MycoStrip Detection Kit (InvivoGen). HEK Null1 cells were seeded at a density of 2 × 10^6^ cells per well in a 6-well plate (Corning) and incubated 4 h, after which cells were transfected with a mixture of pCMV SPORT6 Siglec-9 (2.5 μg; Horizon Discovery) and Lipofectamine 2000 (10 μL; ThermoFisher) in OptiMEM (250 μL; Gibco). 24 h after transfection, the medium was removed, and the cells were gently detached in PBS by flushing.

### Glycan binding to HEK Siglec-9 cells

The F545-FAA-glycan probes were added to 5 × 10^5^ HEK Null1 cells or HEK Siglec-9 cells in 500ul PBS at a final concentration of 50pM and the cells were stirred on a wheel at 4 °C for 1 h. Glycan labelling of the cells was determined by flow cytometry analysis on a FACSCalibur (BD Biosciences) and analysed using the CellQuest software (BD Biosciences). Viability of the cells before and after the glycan binding were approximately 85% for all the conditions tested as determined by trypan blue cell counting. Additionally, 10^5^ cells were transferred in a black 96 well plate and total fluorescence was measured on a BMG PHERAStar Plus plate reader (BMG Labtech).

### Reporting summary

Further information on research design is available in the Nature Portfolio Reporting Summary linked to this article.

## Supplementary information

Supplementary Information

Description of Additional Supplementary Files

Supplementary Data 1

Supplementary Data 2

Supplementary Data 3

Supplementary Data 4

Reporting Summary

Transparent Peer Review file

## Source data

Source Data

## Data Availability

Unless otherwise stated, all data supporting the results of this study can be found in the article, supplementary information, and appendix source data files. The experimental metadata for the glycan microarray analyses in compliance with the MIRAGE Glycan Microarray Guidelines are provided in Supplementary Table 8. The microarray data will be made available through the newly developed International Glycan Array Data Repository (https://glygen.ccrc.uga.edu/ggarray/), which is currently in its final testing phase. In the meantime, GlyTouGan IDs (from the international glycan structure repository, https://glytoucan.org/) for all glycan probes included in the microarrays are listed in Supplementary Data 1 and 2, and the glycan array results are organised into two matrix data sheets Supplementary Data 3 and 4. The source data corresponding to Figs. 3 to 8 and Supplementary Figs. S3 to S9 are included in the Source Data File. Source data are provided with this paper.
