# Supplementary material for: Versatile Glycan Probes for Multiplatform Investigation of Glycan Interactions with Proteins, Viruses, and Cells
Source: Nat Commun. 2026 Jul 17;17:8750. doi: 10.1038/s41467-026-75206-2 (PMC13493820; doi:10.1038/s41467-026-75206-2)
Supplement: Supplementary file 1 [file 41467_2026_75206_MOESM1_ESM.pdf]

Versatile Glycan Probes for Multiplatform Investigation of Glycan Interactions with Proteins, Viruses, and Cells

Jin Yu[1], Alexiane Decout[2,†] Jiayun Yang[3], Antonio Di Maio[1], Wengang Chai[1], W. Bruce Turnbull[4], Thilo Stehle[5], Steven Sacks[6], Thomas P Peacock[3], Munir Iqbal[3], Ten Feizi[1], Yan Liu[1*]

[1] Glycosciences Laboratory, Department of Metabolism, Digestion and Reproduction, Faculty of Medicine, Imperial College London, London, United Kingdom

[2] Institute of Reproductive and Developmental Biology, Department of Metabolism Digestion and Reproduction, Imperial College London, London, United Kingdom

[3] The Pirbright Institute, Pirbright, Woking, United Kingdom

[4] School of Chemistry and Astbury Centre for Structural Molecular Biology, University of Leeds, Leeds, United Kingdom

[5] Interfaculty Institute of Biochemistry, University of Tübingen, Germany

[6] Medical Research Council (MRC) Centre for Transplantation, King's College London, London, United Kingdom.

[†] Current address: Division of Biomedical Sciences, Warwick Medical School, University of Warwick, Coventry CV4 7AL, UK

***Email:** yan.liu2@imperial.ac.uk

Supplementary Information

SUPPLEMENTARY FIGURES 3

Supplementary Fig. 1. Comparative HPLC chromatographs of glycans tagged with FAA, 2AB and AEAB. 3

Supplementary Fig. 2. Overall synthesis scheme of glycan probes. 4

Supplementary Fig. 3. Binding analyses of noncovalent and covalent arrays using anti-blood group antibodies. 5

Supplementary Fig. 4. Binding analyses of noncovalent and covalent arrays using anti-glycolipid antibodies. 6

Supplementary Fig. 5. Binding analyses of noncovalent and covalent arrays using Sialic acid binding lectins. 7

Supplementary Fig. 6. Binding analyses of noncovalent and covalent arrays using plant lectins. 8

Supplementary Fig. 7. Binding analyses of noncovalent and covalent arrays using bacteria toxins at a lower concentration. 9

Supplementary Fig. 8. BLI binding analyses of H7N9, H5N1, and H1N1 live influenza viruses with FAA and PAA probes. 10

Supplementary Fig. 9. NGL array incubated with A/England/195/2009 (H1N1) virus. 11

Supplementary Fig. 10. NMR spectra of compound 1. 13

Supplementary Fig. 11. NMR spectra of compound 2. 15

Supplementary Fig. 12. HI-ESI-MS spectra of compound 1 16

Supplementary Fig. 13. HI-ESI-MS spectra of compound 2 16

Supplementary Fig. 14. Generation of correlation 'curve'. 17

Supplementary Fig. 15. HPLC profiles and MS spectra of 44 FAA-linked sequence-defined glycans and corresponding NGLs 18

Supplementary Fig. 16. Examples of MALDI spectra of Fmoc deprotected glycan probes....................... 62
Supplementary Fig. 17. MALDI spectra of biotinylated glycan probes. 64
Supplementary Fig. 18. MALDI spectra of F_{545} labeled glycan probes. 65
Supplementary Fig. 19. Enzymatic modifications of N glycan probe 36 to generate biantennary *N*-glycan probes 37-41. 67
Supplementary Fig. 20. Spotting record of covalent 44 probe FAA glycan array. 68
Supplementary Fig. 21. Array images. 69
SUPPLEMENTARY TABLES 76
Supplementary Table 1. Highlights of glycan binding readouts from covalent and noncovalent arrays.... 76
Supplementary Table 2. Designations of sequence-defined glycans. 77
Supplementary Table 3. Anti-carbohydrate antibodies. 78
Supplementary Table 4. Detection antibodies. 78
Supplementary Table 5. Biotinylated lectins. 78
Supplementary Table 6. Glycan binding receptors of the immune system 79
Supplementary Table 7. Microbial glycan binding proteins used in array analysis. 79
Supplementary Table 8. MIRAGE compliant Glycan Microarray Document 80
SUPPLEMENTARY METHODS 83
General methods 83
Synthesis of the FAA linker 83
Preparation of 2AB- and AEAB- tagged glycans for comparing with FAA-tagged glycans' chromatographic profiles 84
Enzymatic modifications on FAA-*N*-glycan probe 36 84
Generation of Fmoc-deprotected FAA glycan probes for covalent arrays 85
Generation of FAA-NGL probes for noncovalent glycan arrays 85
SUPPLEMENTARY DISCUSSION 86
Details of binding signals given by fucosylated glycans using antibodies and plant lectins 86
Details of binding signals given by two anti-ganglioside antibodies.. 86
Details of binding signals given by Sia, Man, Gal, GalNAc terminating glycans using plant lectins.......... 86
Details of binding signals given by CLRs 87
Details of binding signals given by E-selectins 87
Details of binding signals given by bacterial toxins on non-sialyl glycans 87
SUPPLEMENTARY REFERENCES 88

SUPPLEMENTARY FIGURES

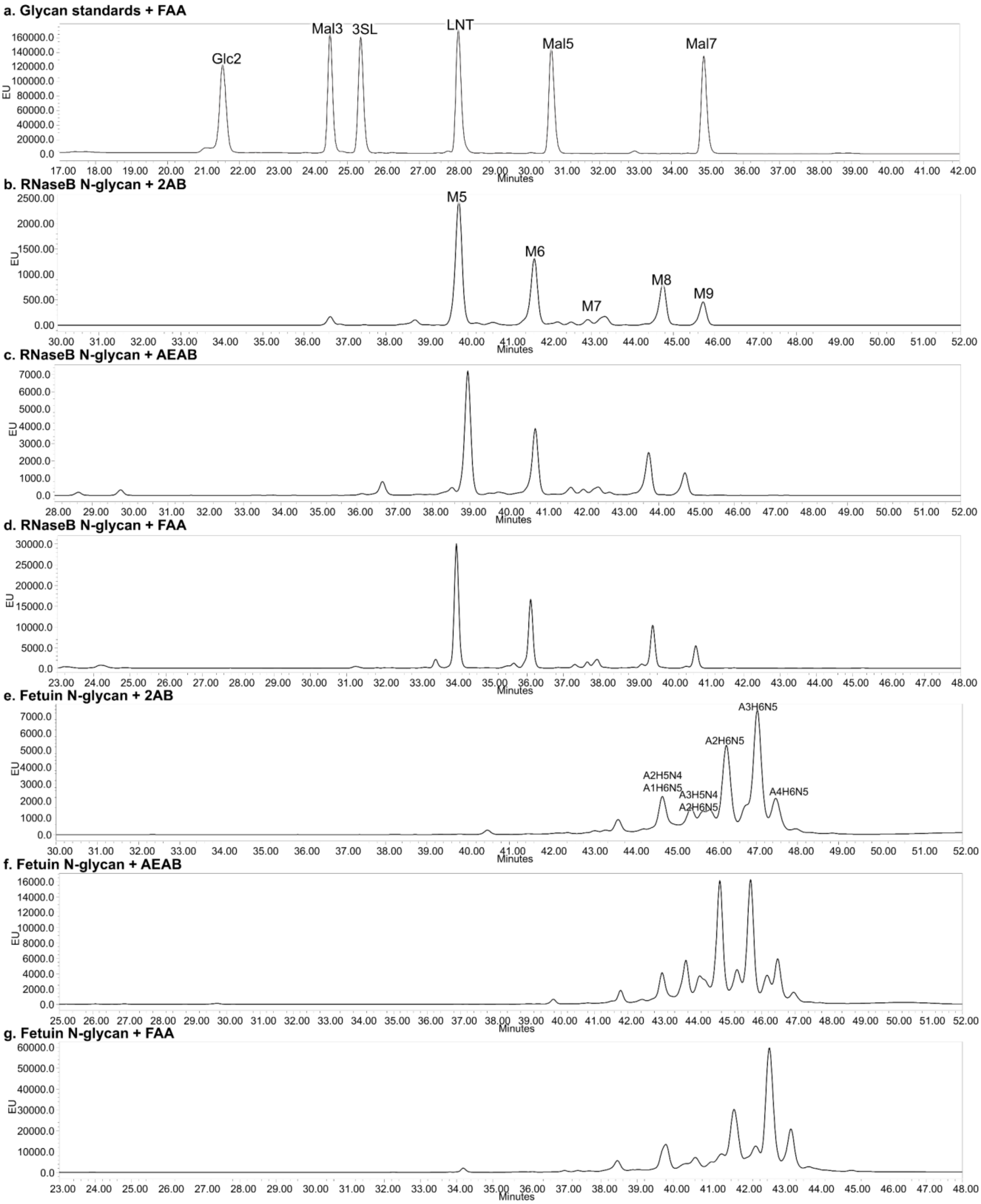

Supplementary Fig. 1. Comparative HPLC chromatographs of glycans tagged with FAA, 2AB and AEAB. A Mixture of sequence-defined glycans, 3 nmol of each, tagged with FAA. **b-d**: Aliquots, 0.5% of oligomannose mixture released with EndoH from 40 mg RNaseB tagged with 2AB (**b**), with AEAB (**c**), and with FAA (**d**). **e-g**: Aliquots, 0.5% of *N*-glycan mixture released with PNGF from 40 mg fetuin tagged with 2AB (**e**), with AEAB (**f**) and with FAA (**g**). The y-axis of the HPLC chromatogram represents the emission units (EU) corresponding to the fluorescence intensity of the labelled glycans.

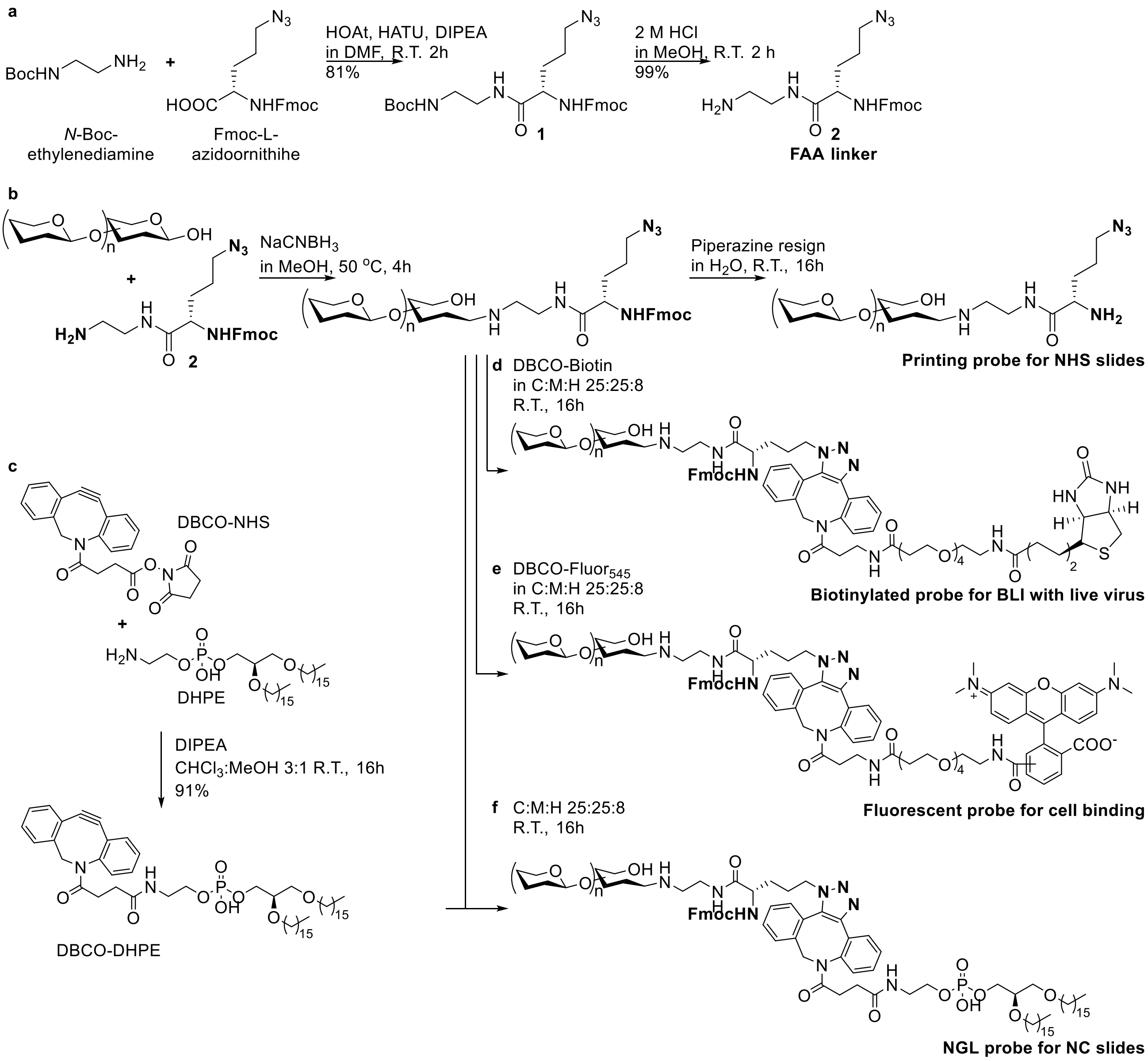

Supplementary Fig. 2. Overall synthesis scheme of glycan probes.
a Synthesis of FAA linker from commercial Fmoc amino acid. **b** Glycan labeling by reductive method and Fmoc remove on solid support. **c** Synthesis of DBCO-DHPE reagent. **d** Biotinylation on the glycan probe. **e** Fluorescent labelling of the glycan probe. **f** Conversion of the labelled glycan into NGL form.

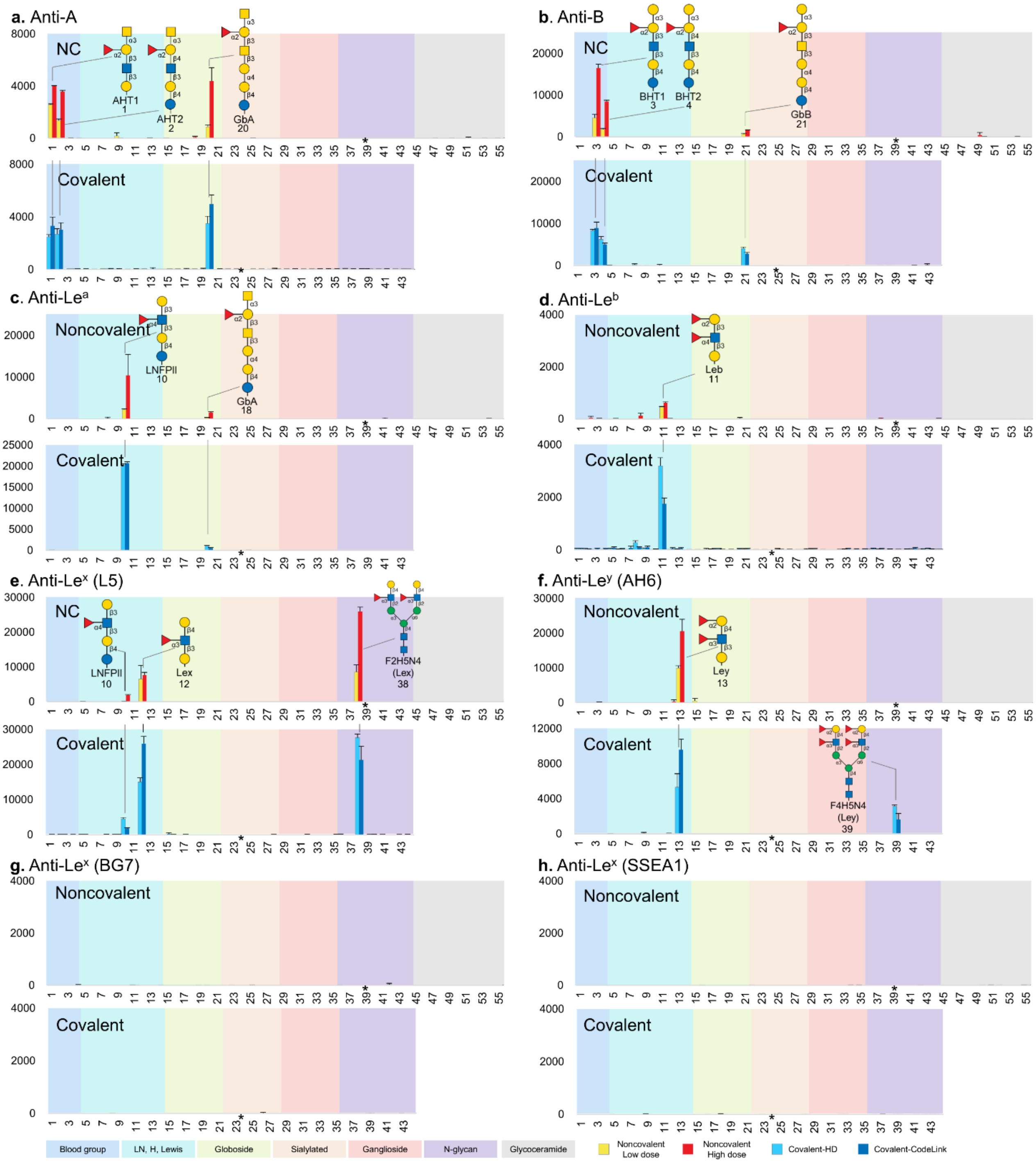

Supplementary Fig. 3. Binding analyses of noncovalent and covalent arrays using anti-blood group antibodies. Results are shown as fluorescence intensities at 2 and 5 fmol per spot printing levels for each probe on the noncovalent NGL arrays, calculated from averaged duplicate spot intensities at each printing level (error bars represent half of the difference between the two values), and of the averaged quadruplicate spot fluorescence intensities of each glycan probe in the covalent arrays (technical replicates, n = 4, with error bars representing the SD of the four values). *Probes that were misprinted and did not pass quality control: probe 24 on the covalent arrays and probe 39 on the noncovalent array.

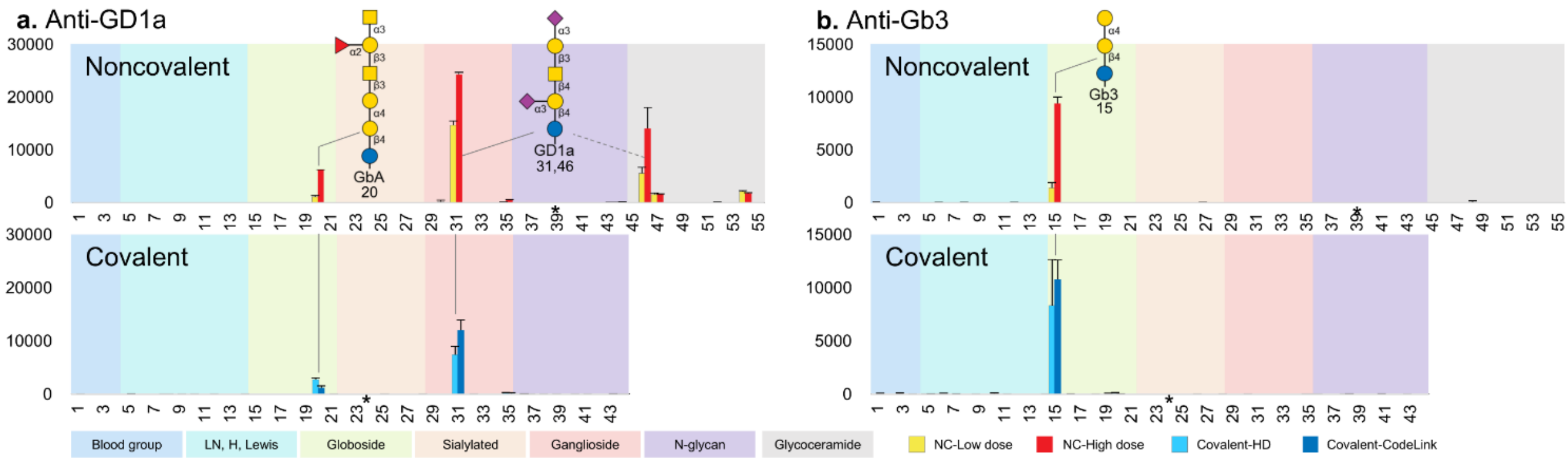

Supplementary Fig. 4. Binding analyses of noncovalent and covalent arrays using anti-glycolipid antibodies. Results are shown as fluorescence intensities at 2 and 5 fmol per spot printing levels for each probe on the noncovalent NGL arrays, calculated from averaged duplicate spot intensities at each printing level (error bars represent half of the difference between the two values), and of the averaged quadruplicate spot fluorescence intensities of each glycan probe in the covalent arrays (technical replicates, n = 4, with error bars representing the SD of the four values). *Probes that were misprinted and did not pass quality control: probe 24 on the covalent arrays and probe 39 on the noncovalent array.

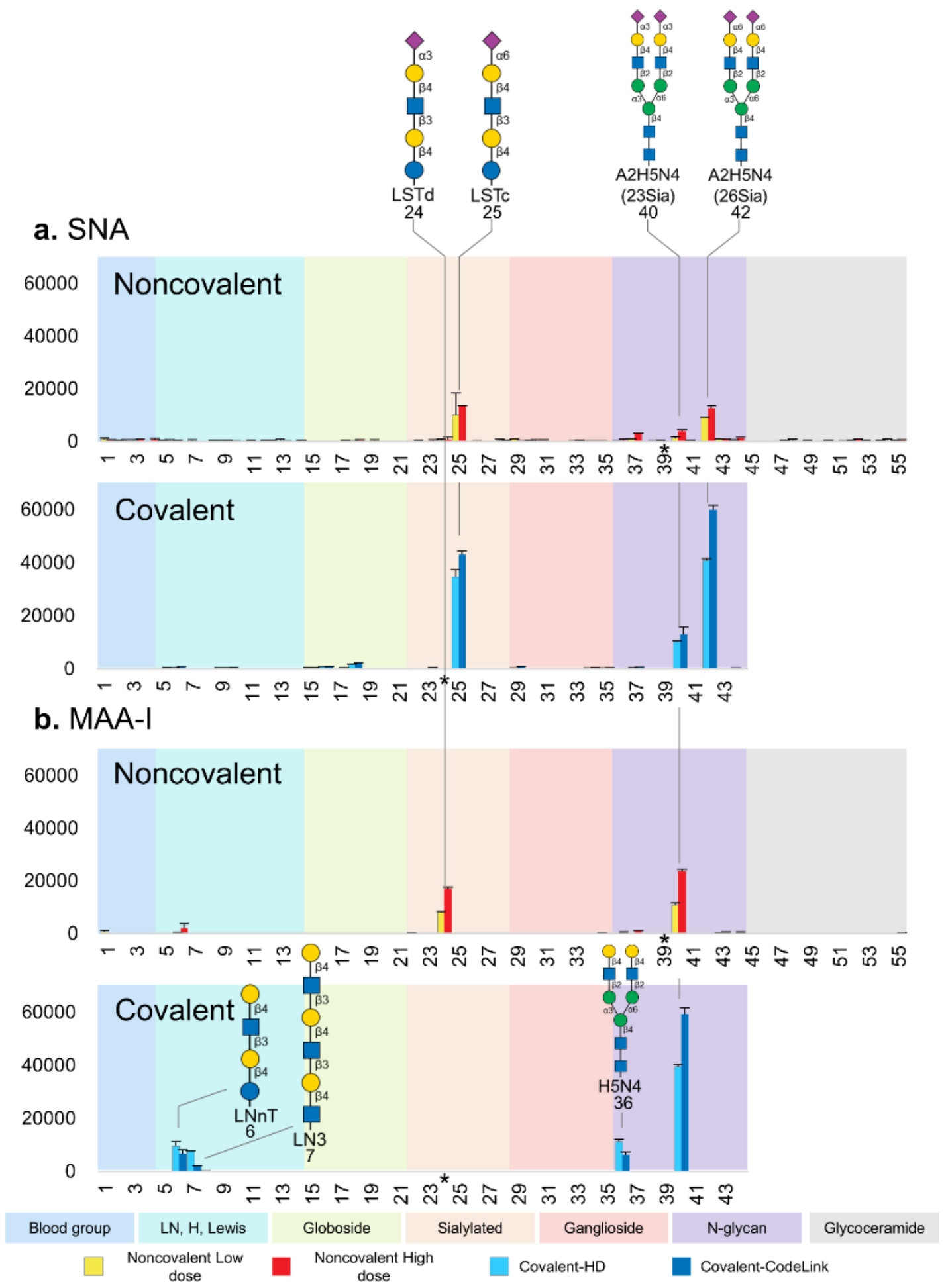

Supplementary Fig. 5. Binding analyses of noncovalent and covalent arrays using Sialic acid binding lectins. Results are shown as fluorescence intensities at 2 and 5 fmol per spot printing levels for each probe on the noncovalent NGL arrays, calculated from averaged duplicate spot intensities at each printing level (error bars represent half of the difference between the two values), and of the averaged quadruplicate spot fluorescence intensities of each glycan probe in the covalent arrays (technical replicates, n = 4, with error bars representing the SD of the four values). *Probes that were misprinted and did not pass quality control: probe 24 on the covalent arrays and probe 39 on the noncovalent array.

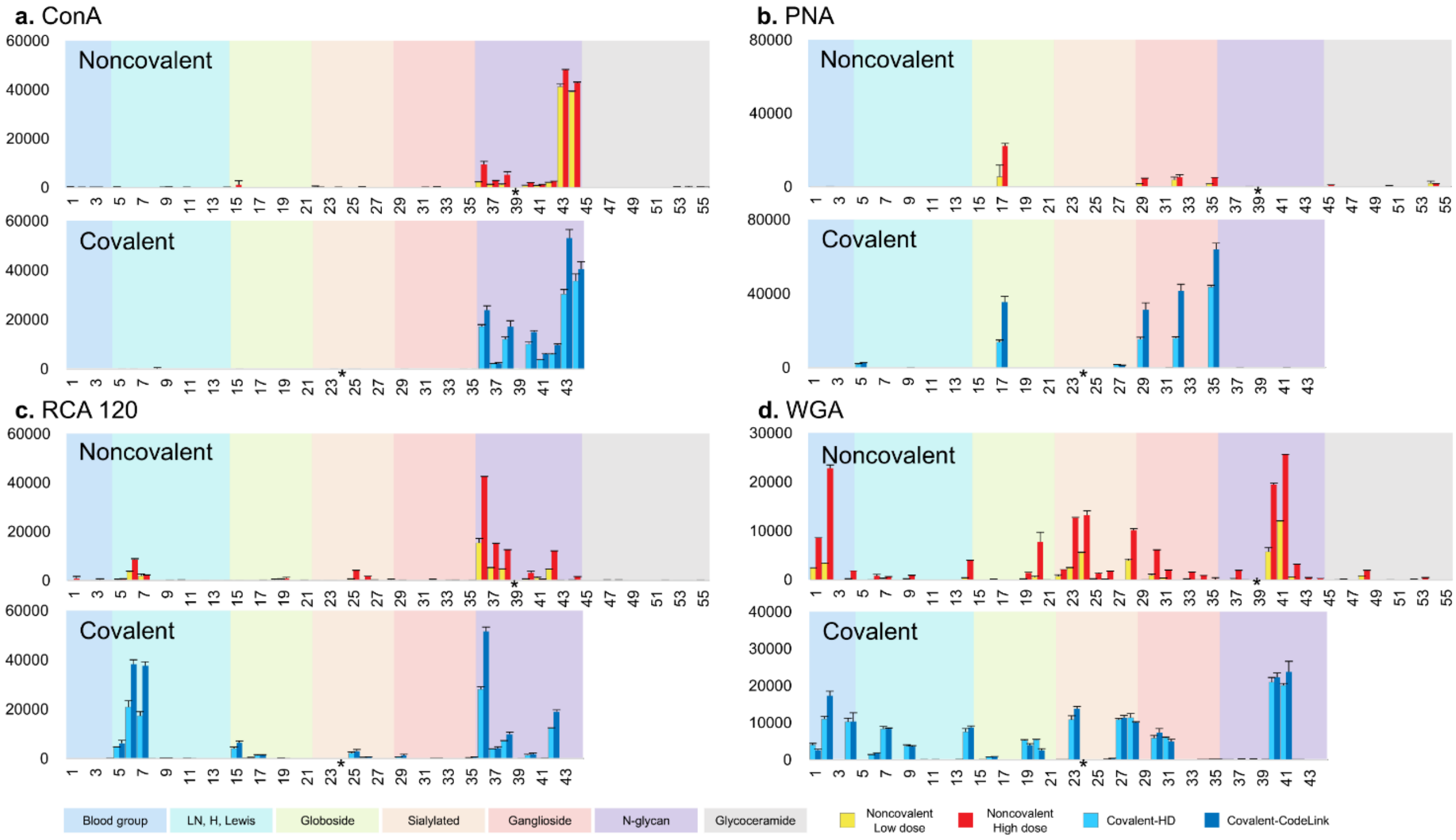

Supplementary Fig. 6. Binding analyses of noncovalent and covalent arrays using plant lectins. Results are shown as fluorescence intensities at 2 and 5 fmol per spot printing levels for each probe on the noncovalent NGL arrays, calculated from averaged duplicate spot intensities at each printing level (error bars represent half of the difference between the two values), and of the averaged quadruplicate spot fluorescence intensities of each glycan probe in the covalent arrays (technical replicates, n = 4, with error bars representing the SD of the four values). *Probes that were misprinted and did not pass quality control: probe 24 on the covalent arrays and probe 39 on the noncovalent array.

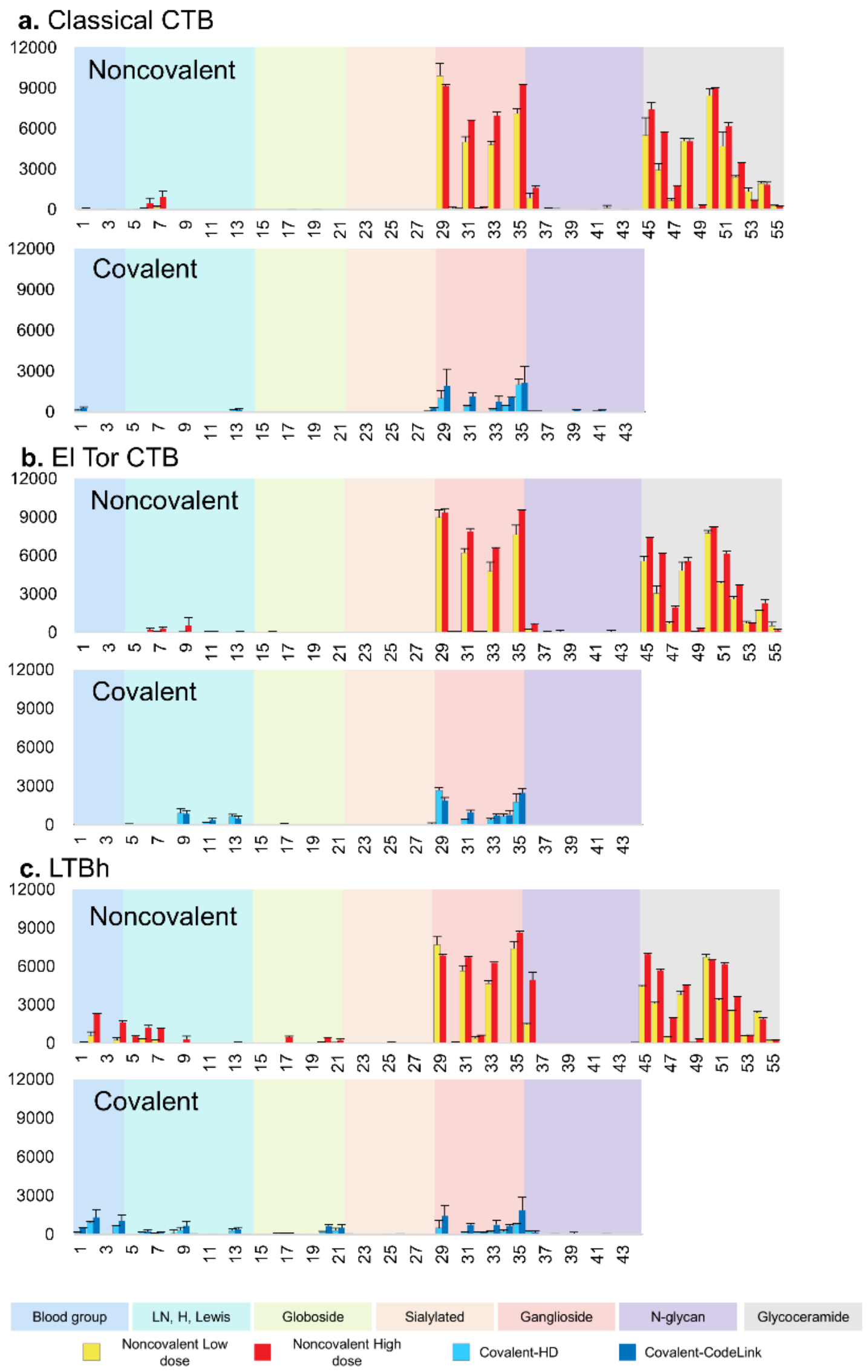

Supplementary Fig. 7. Binding analyses of noncovalent and covalent arrays using bacteria toxins at a lower concentration.

Protein concentration of 0.5 µg/mL was used, in comparison with incubations using 5 µg/mL shown in Fig. 6d-f in the main text. Results are shown as fluorescence intensities at 2 and 5 fmol per spot printing levels for each probe on the noncovalent NGL arrays, calculated from averaged duplicate spot intensities at each printing level (error bars represent half of the difference between the two values), and of the averaged quadruplicate spot fluorescence intensities of each glycan probe in the covalent arrays (technical replicates, n = 4, with error bars representing the SD of the four values). *Probes that were misprinted and did not pass quality control: probe 24 on the covalent arrays and probe 39 on the noncovalent array.

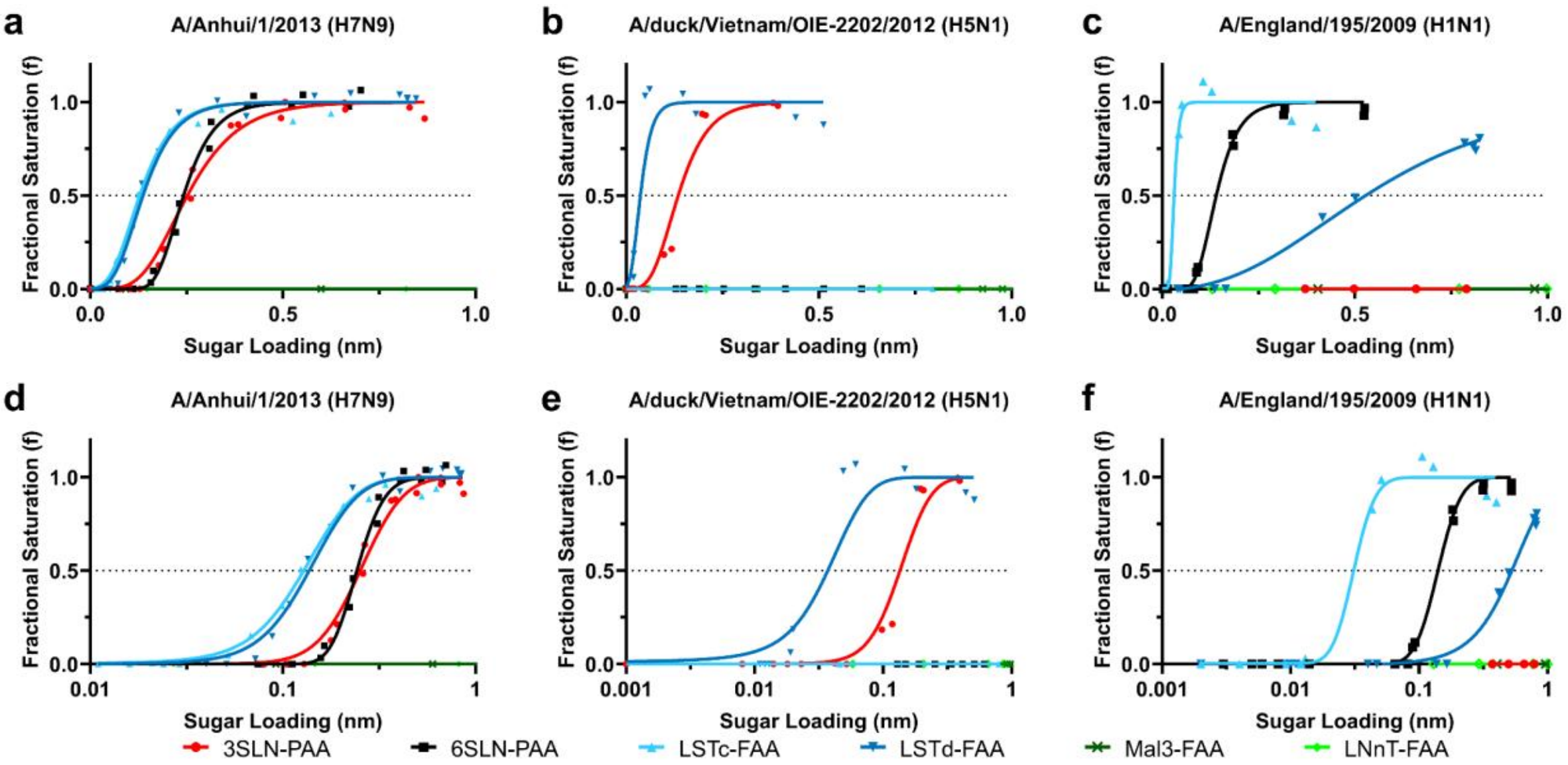

Supplementary Fig. 8. BLI binding analyses of H7N9, H5N1, and H1N1 live influenza viruses with FAA and PAA probes.

The x-axis, expressed in nanometers (nm), represents the wavelength shift corresponding to the change in optical thickness that occurs when glycans are immobilized and binding events take place on the probe surface, thereby reflecting the glycan density on the sensor. The y-axis (fractional saturation) denotes the normalized binding signal (0–1), indicating the extent of ligand occupancy relative to saturation. Panels **a–c** are plotted using a linear scale for glycan loading, whereas panels **d–f** are plotted using a log-mid scale. In panels **b**, **c**, **e**, and **f**, the starting glycan concentration was reduced by half relative to panels **a** and **d**, and the dilution factor was increased from 1.75-fold (532 ~ 11 nM) to 3-fold (266 ~ 0.1 nM), thereby extending the dynamic range of the binding data. The experimental datasets corresponding to these analyses are provided in **Source Data.**

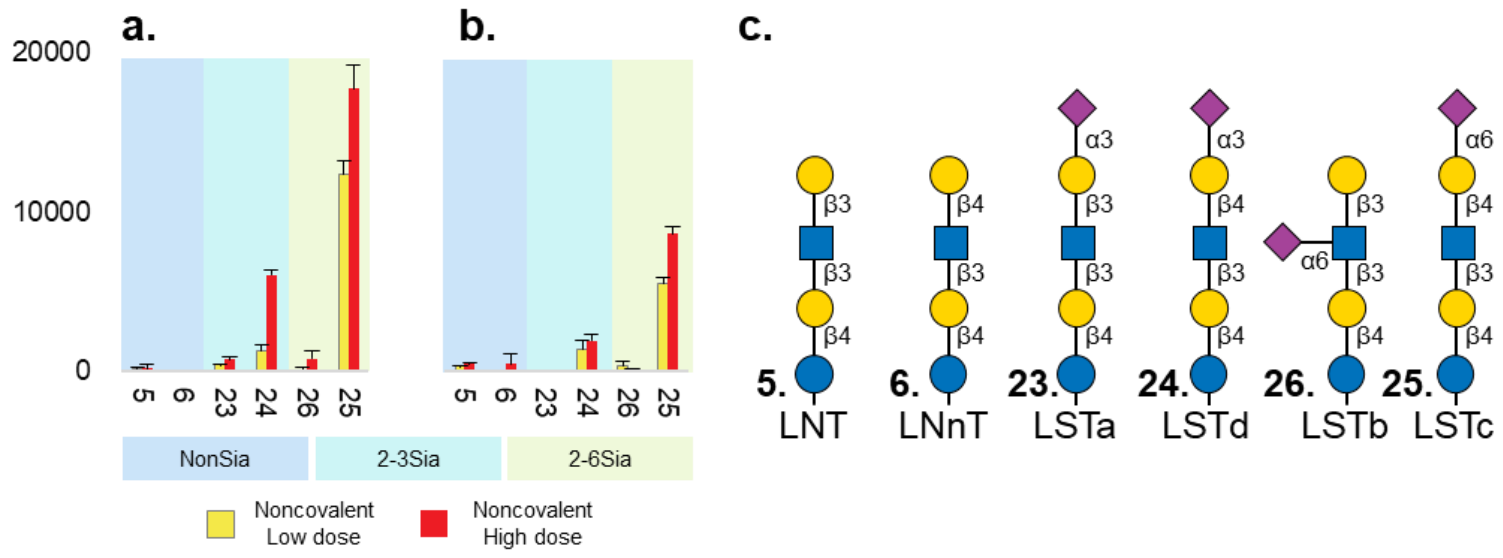

Supplementary Fig. 9. NGL array incubated with A/England/195/2009 (H1N1) virus.
a. H1N1 virus incubated at 1.4 μM. **b**. H1N1 virus incubated at 0.14 μM. **c**. Six FAA probes in this collection. The same virus incubation conditions as those used in the BLI setup were applied. In brief, purified viruses were diluted in HBS-EP buffer containing 10 μM oseltamivir carboxylate and 10 μM zanamivir to inhibit neuraminidase activity, and incubated on the slide for 1.5 h. Unbound viruses were removed by aspiration, and the slides were fixed with 1% formaldehyde on ice for 30 min. Subsequently, the samples were sequentially incubated with H1N1 2-12C monoclonal antibody (1:100, 1 h), biotinylated anti-human IgG (1:200, 1 h), and finally visualized by overlaying with Alexa Fluor 647–labeled streptavidin (1 μg/mL, 30 min).

a. 1H spectrum

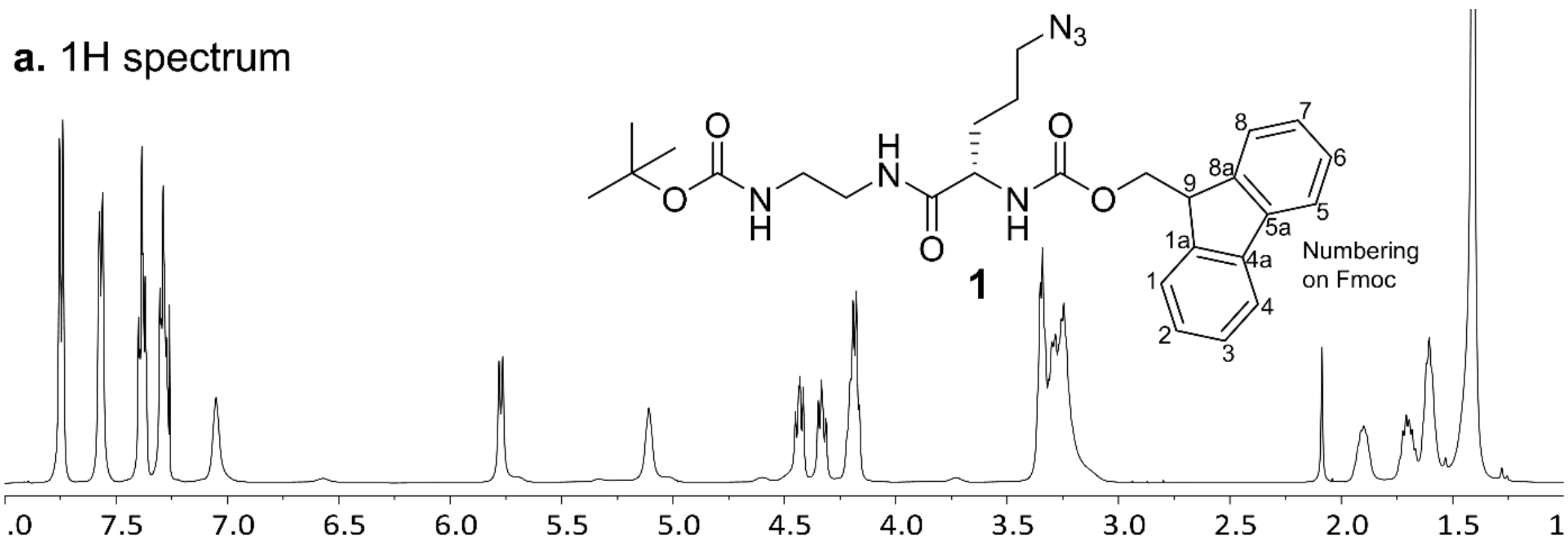

b. 13C spectrum

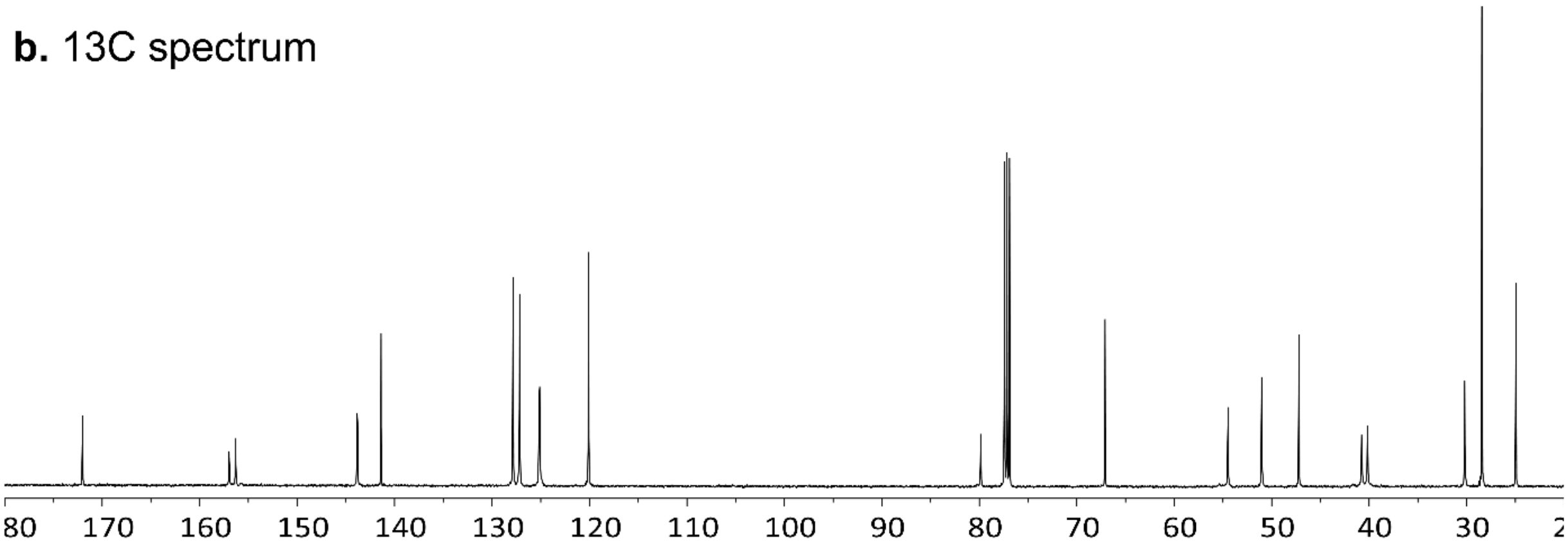

c. HSQC spectrum

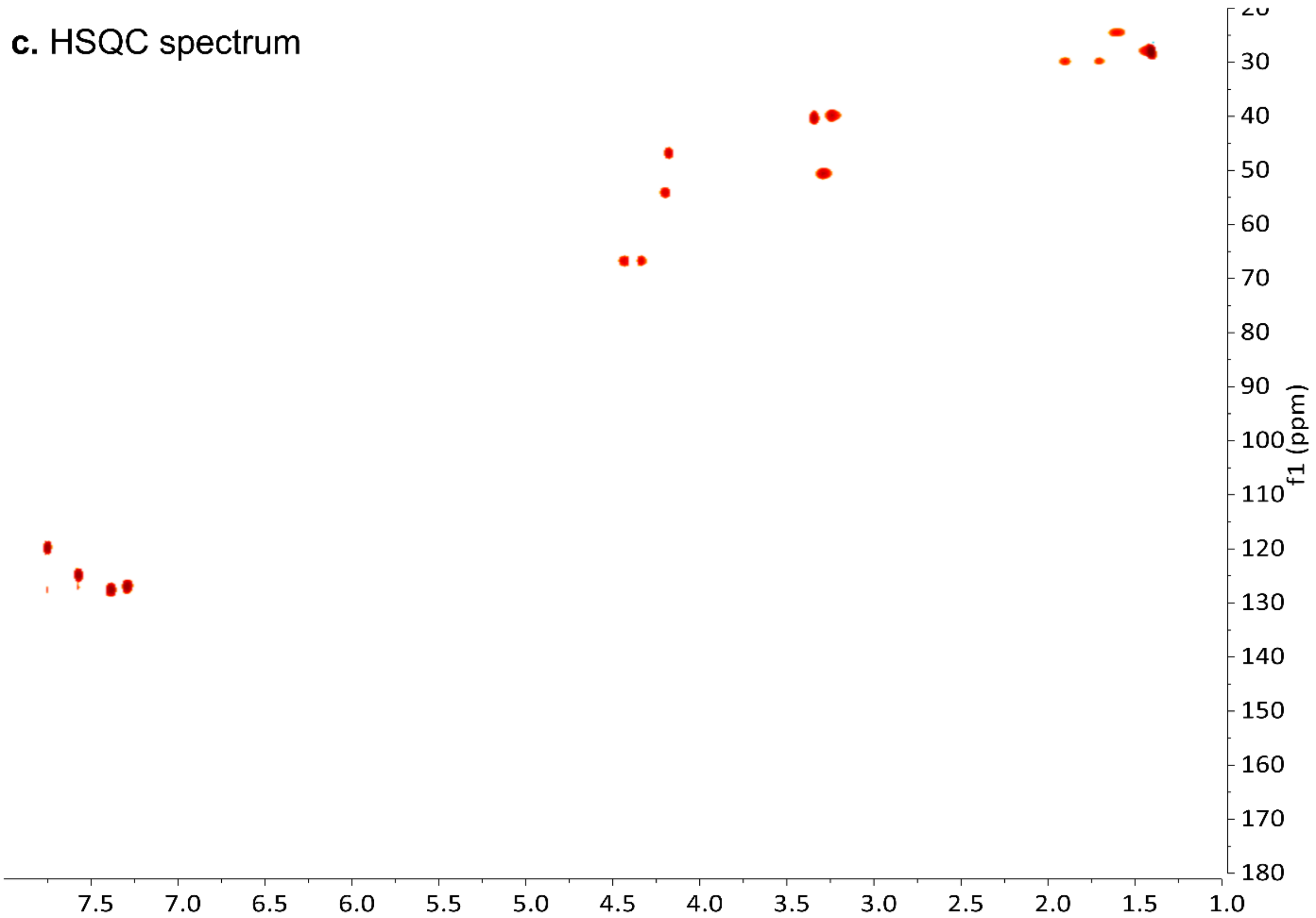

d. COSY spectrum

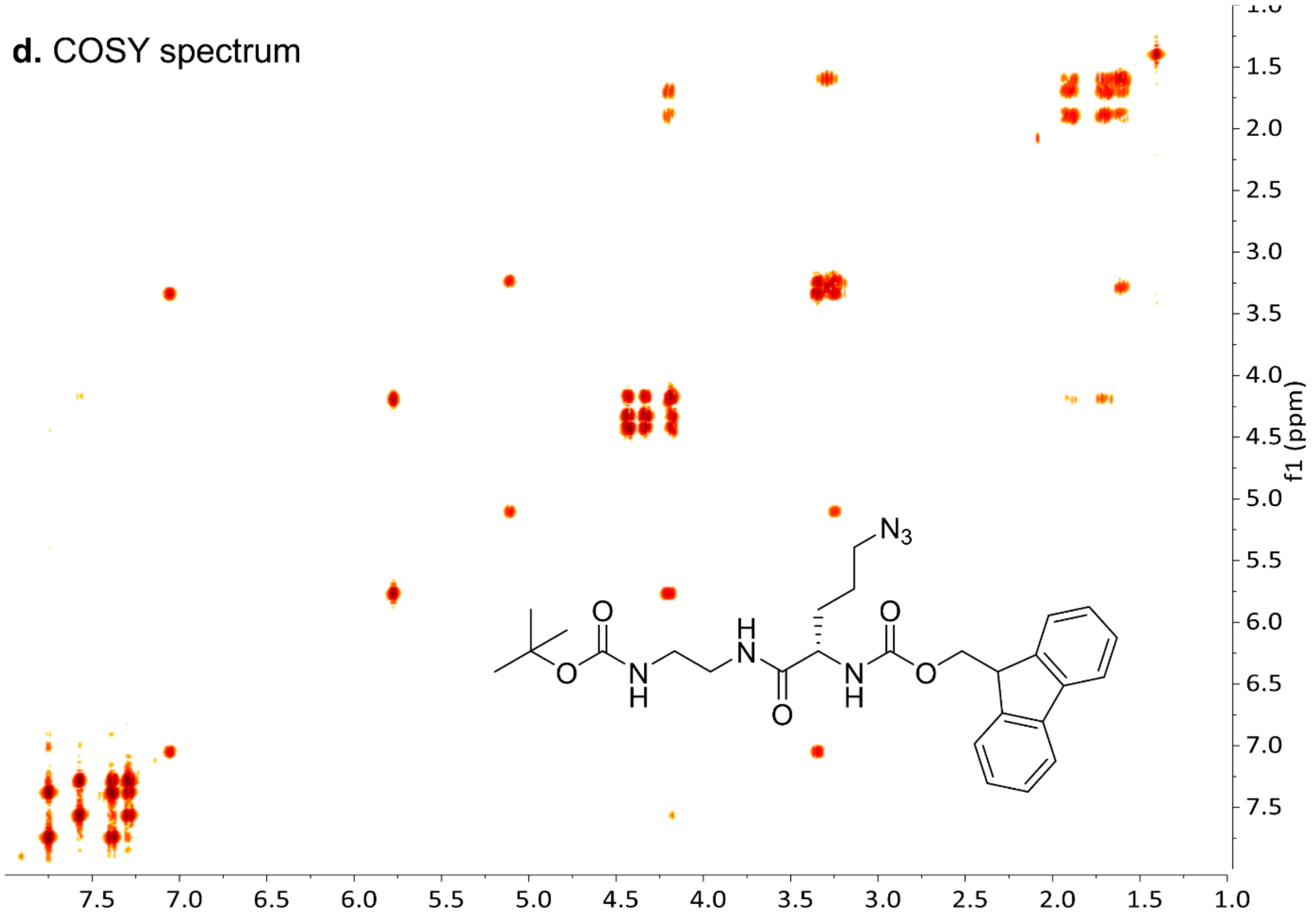

e. HMBC spectrum

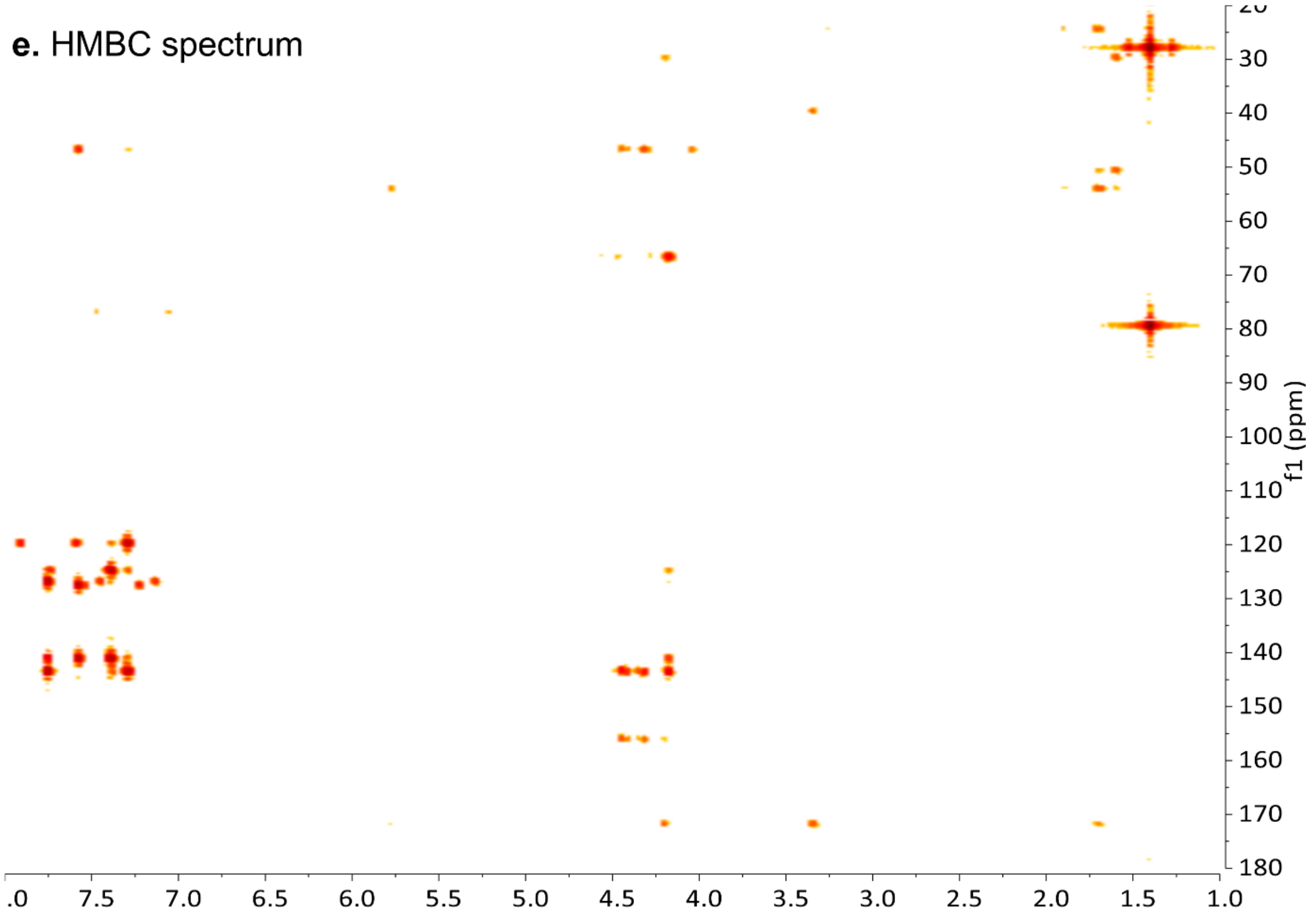

Supplementary Fig. 10. NMR spectra of compound 1.
a 1H spectrum. **b** 13C spectrum. **c** HSQC spectrum. **d** COSY spectrum. **e** HMBC spectrum.

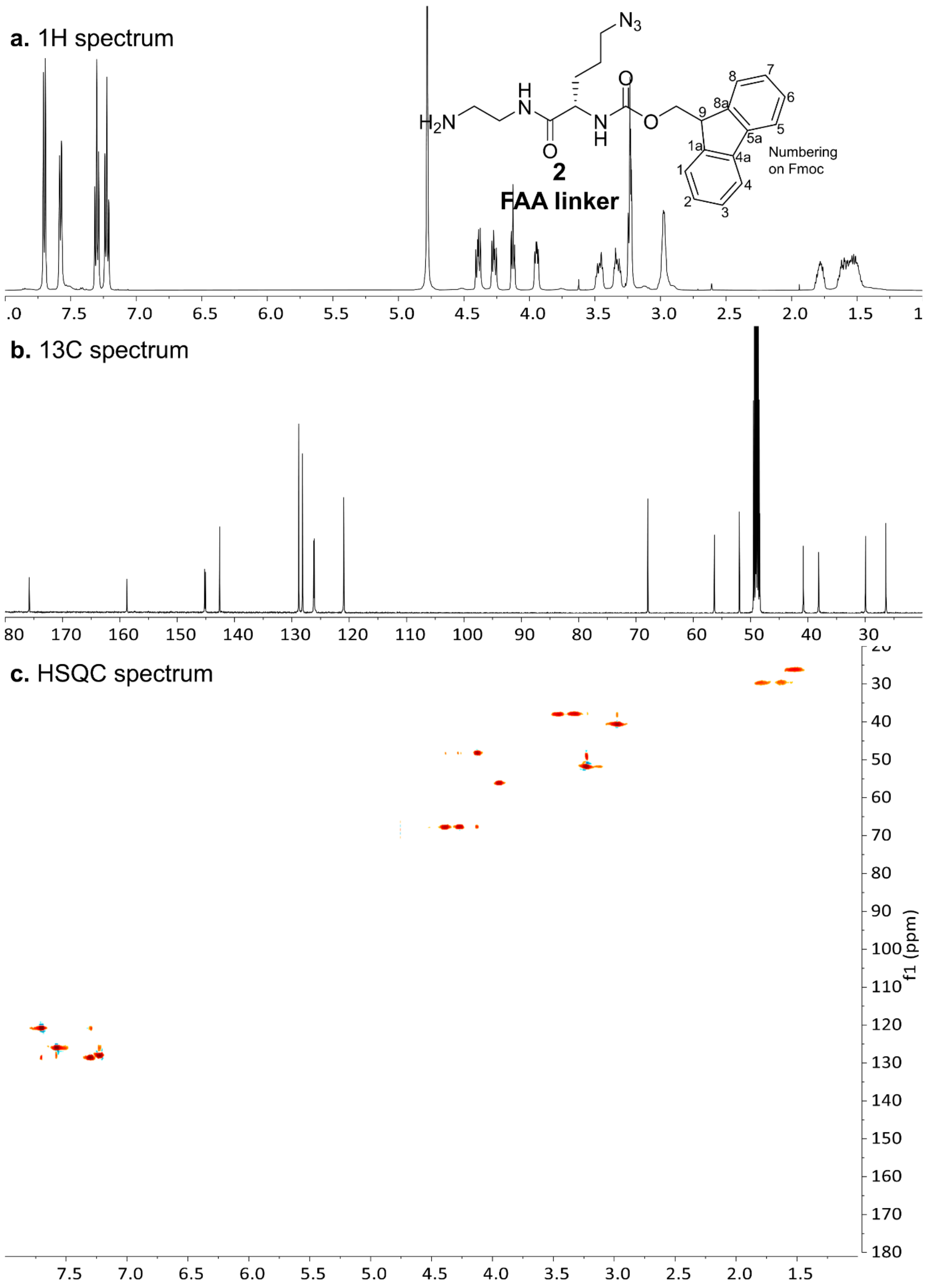
a. 1H spectrum
N3
H
N
H2N
O
N
H
O
O
2
FAA linker
9
8
7
6
5
5a
8a
1a
4a
1
2
3
4
Numbering
on Fmoc
b. 13C spectrum
c. HSQC spectrum
f1 (ppm)

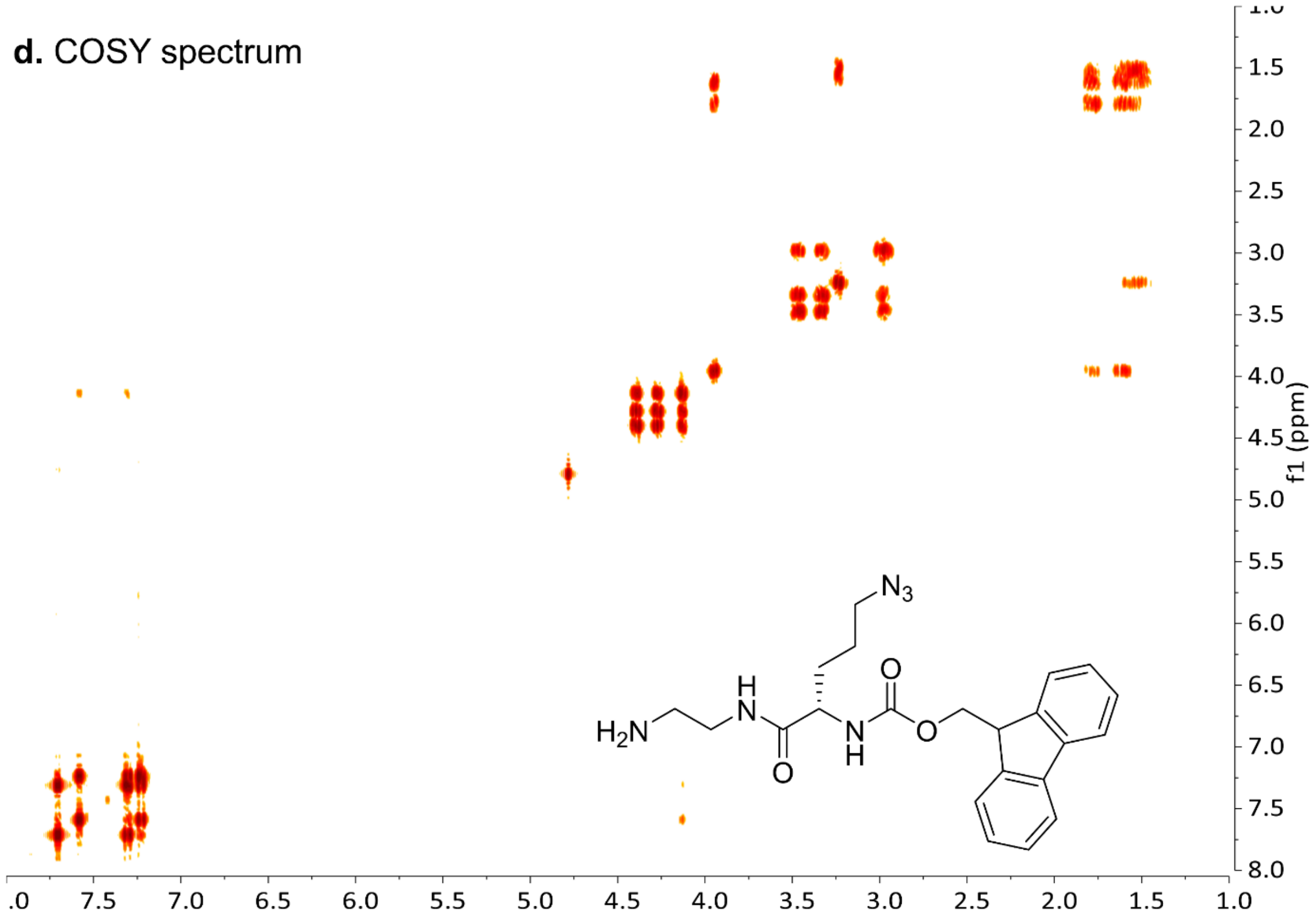

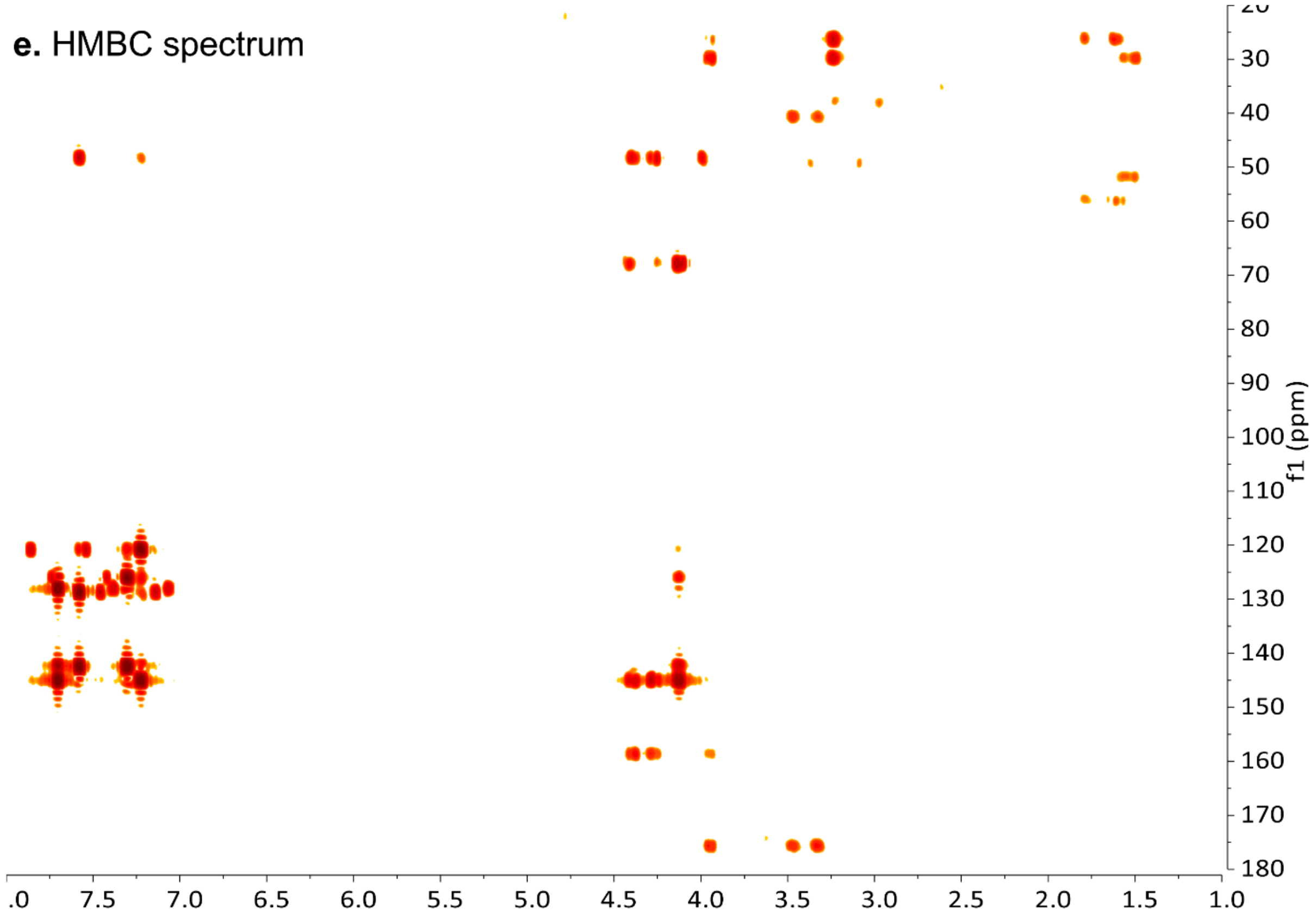

Supplementary Fig. 11. NMR spectra of compound 2.
a 1H spectrum. **b** 13C spectrum. **c** HSQC spectrum. **d** COSY spectrum. **e** HMBC spectrum.

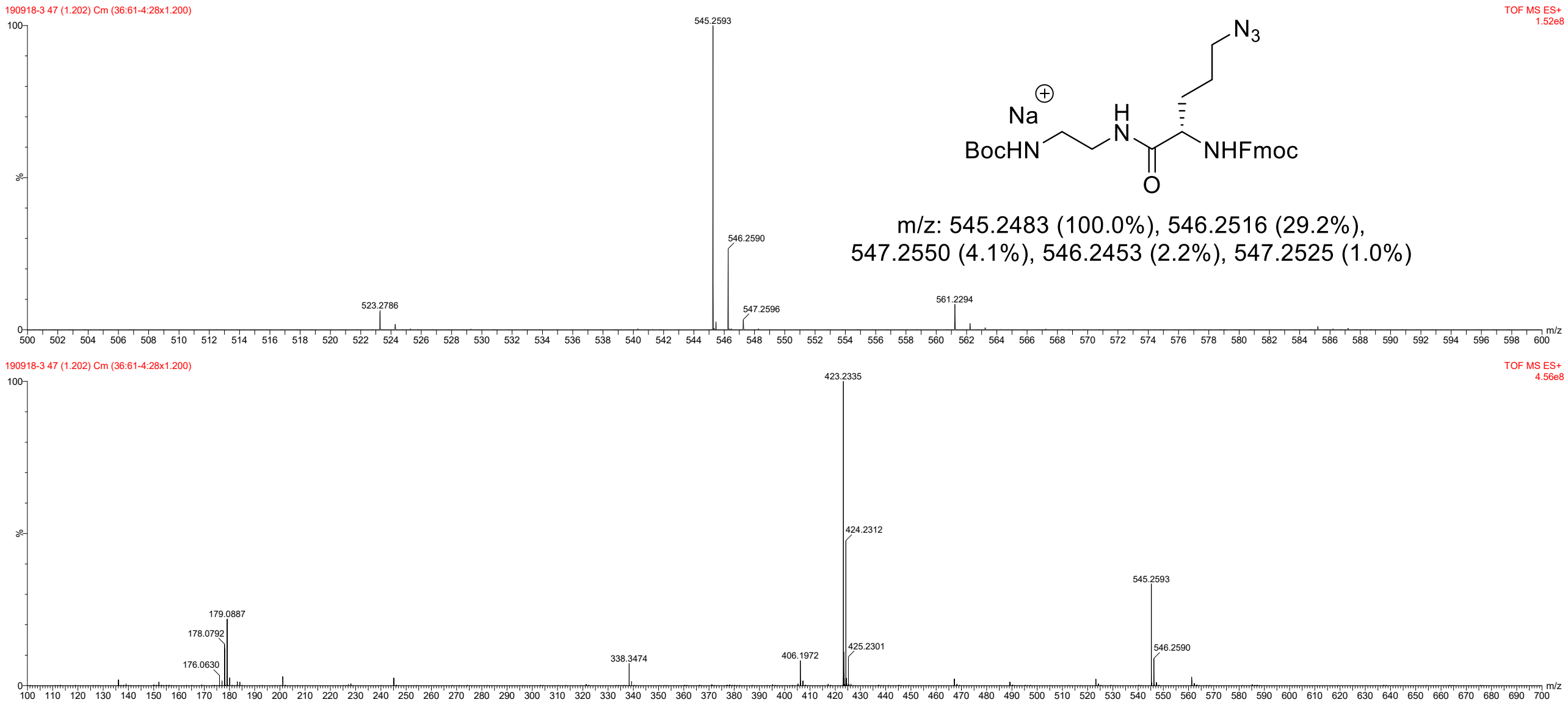

Supplementary Fig. 12. HI-ESI-MS spectra of compound 1

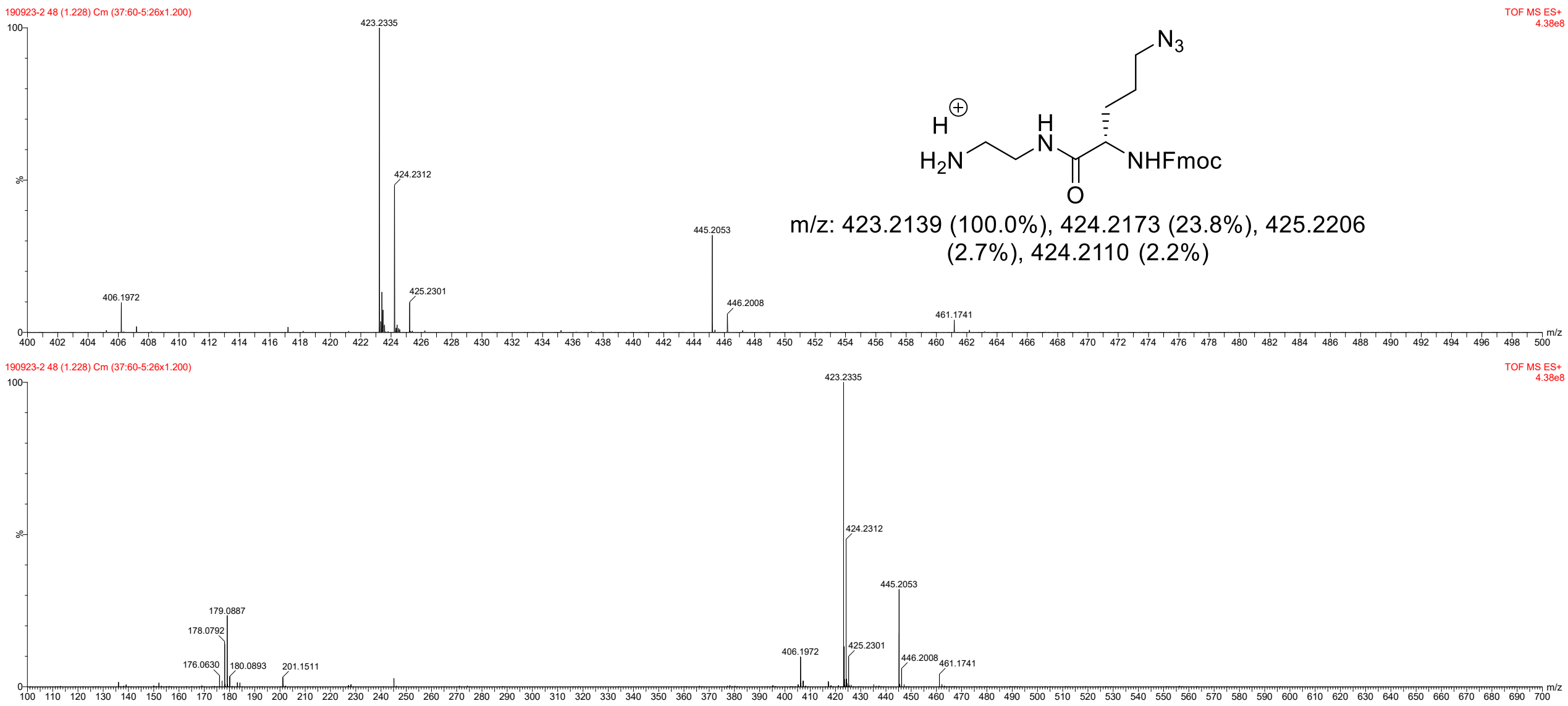

Supplementary Fig. 13. HI-ESI-MS spectra of compound 2

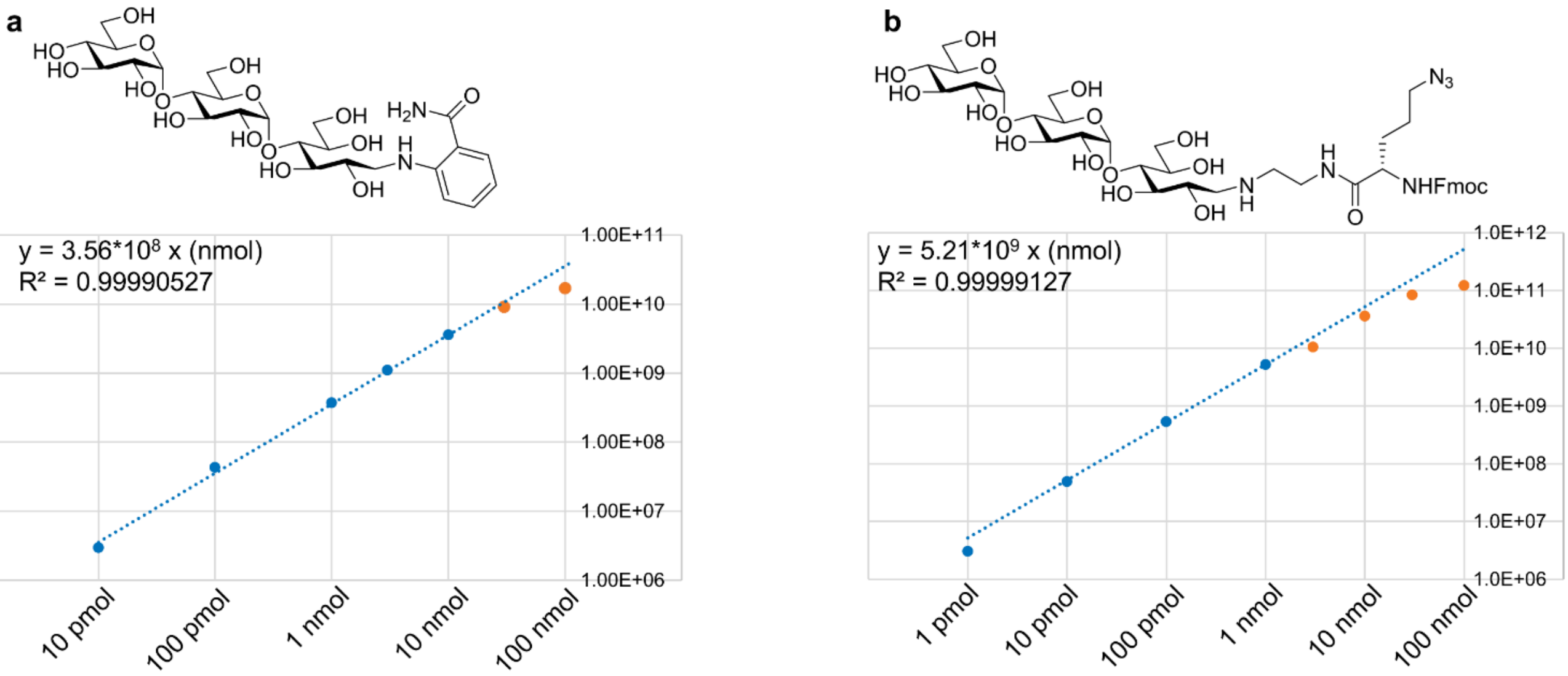

Supplementary Fig. 14. Generation of correlation 'curve'.
Correlation curve of florescence intensity with amounts (nmol) of tagged glycans using maltotriose as a standard tagged with 2AB in **a** and with FAA in **b**. The blue data points within the linear range were used for quantitation rather than the red data points which were at saturation of florescence detection.

Supplementary Fig. 15. HPLC profiles and MS spectra of 44 FAA-linked sequence-defined glycans and corresponding NGLs

Probe No 1

A penta Type1-FAA

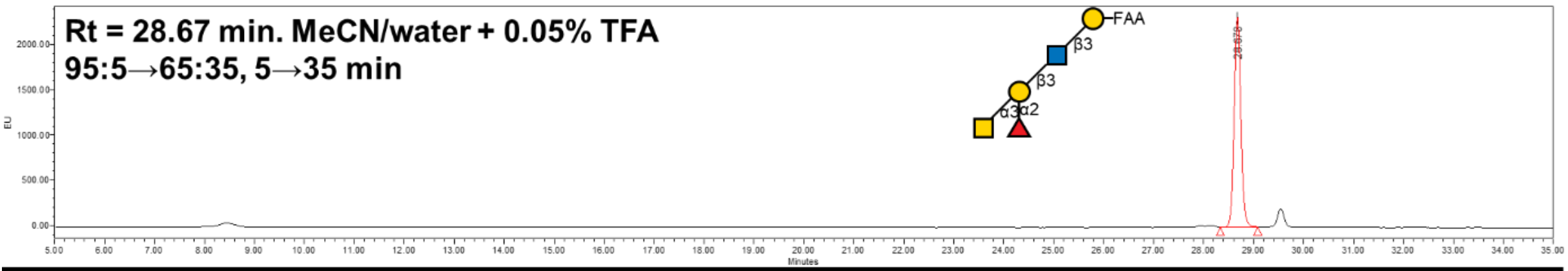

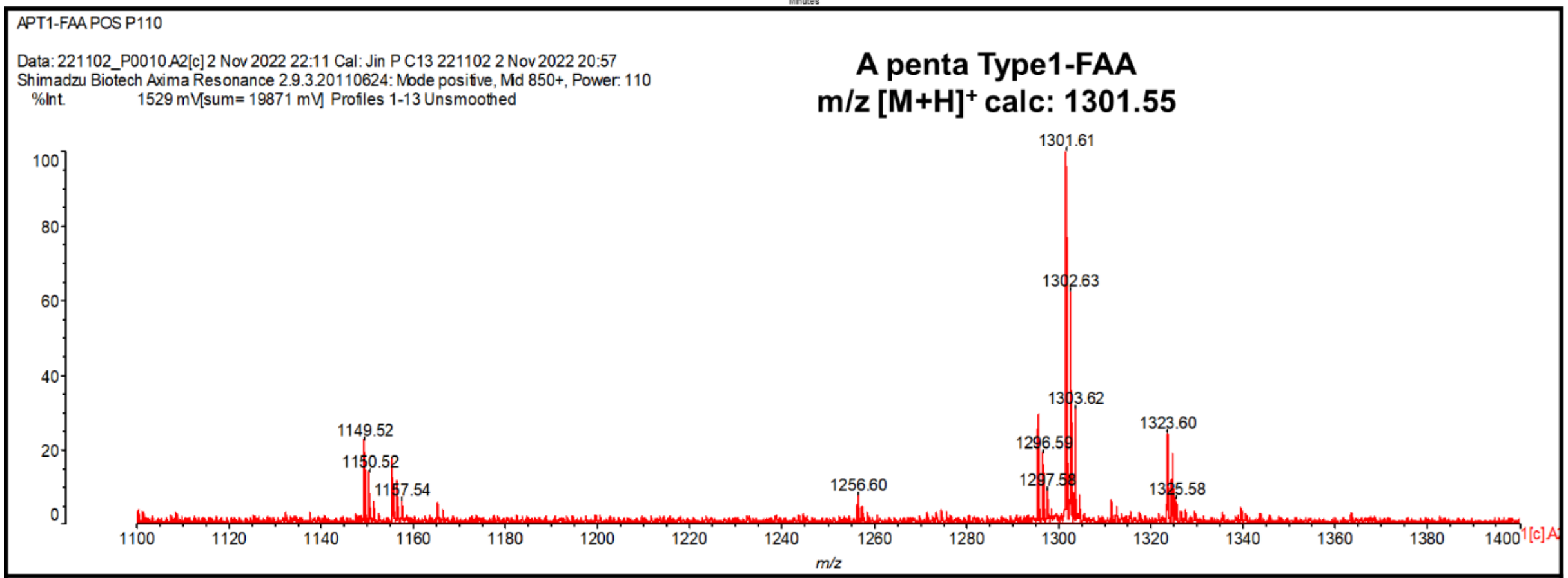

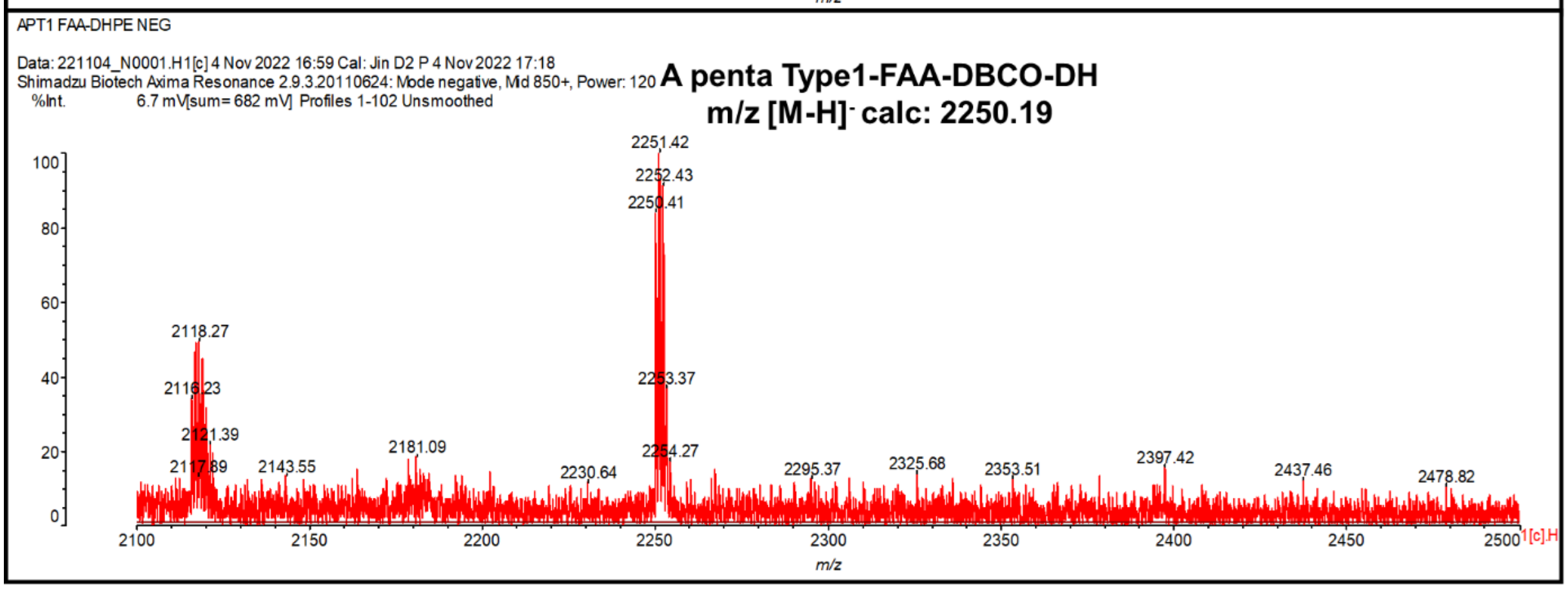

Probe No 2

A hexa Type2-FAA

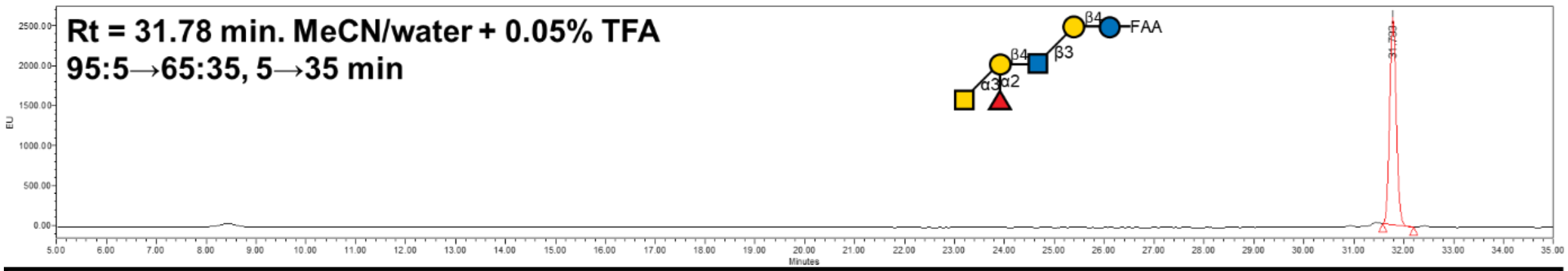

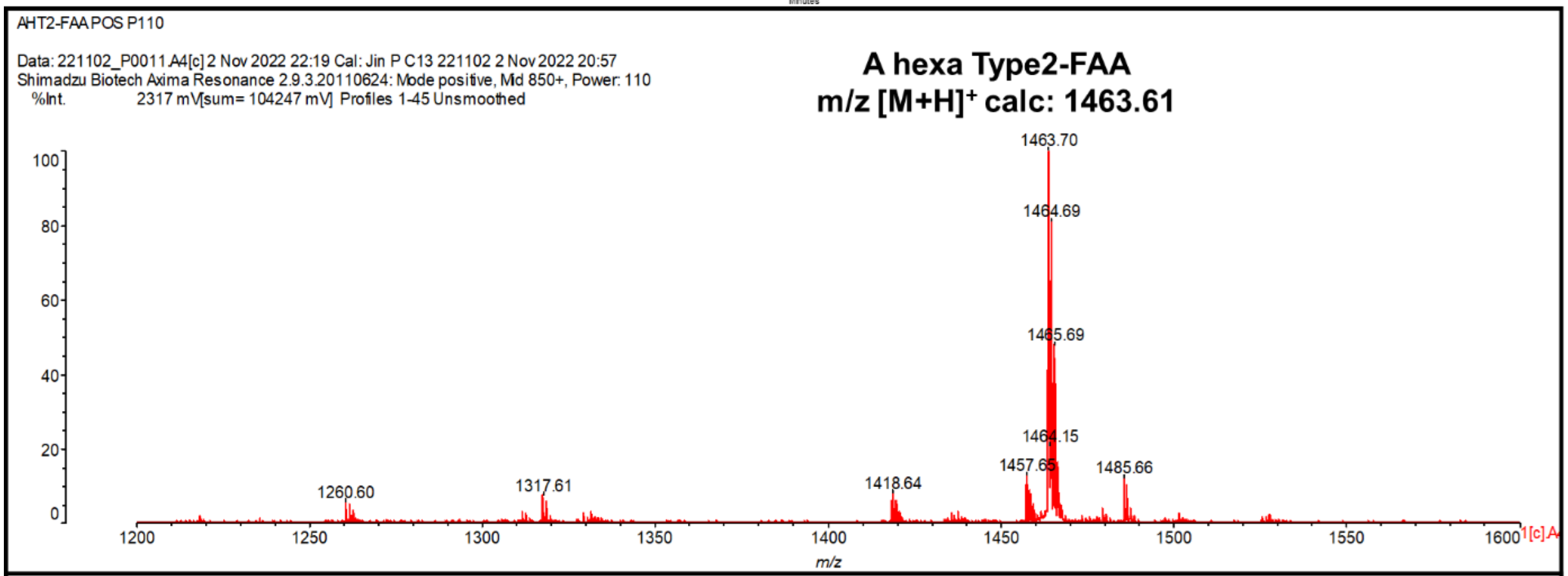

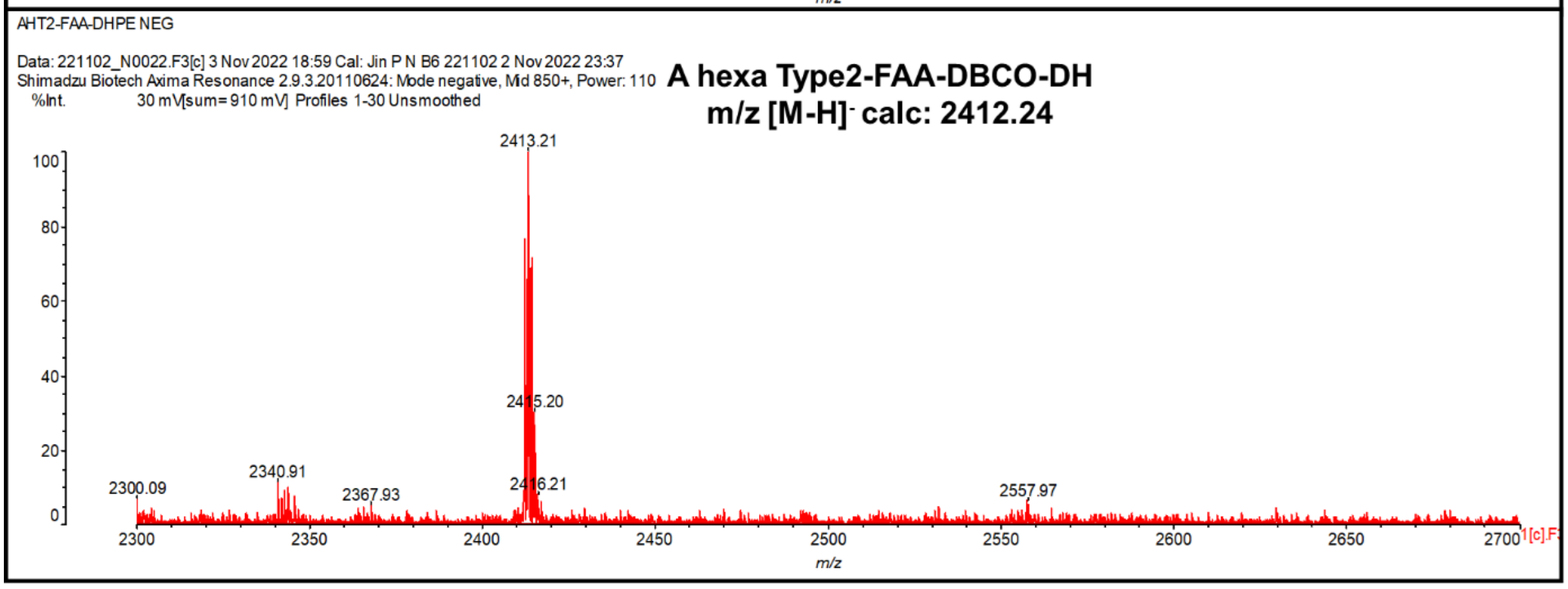

Probe No 3

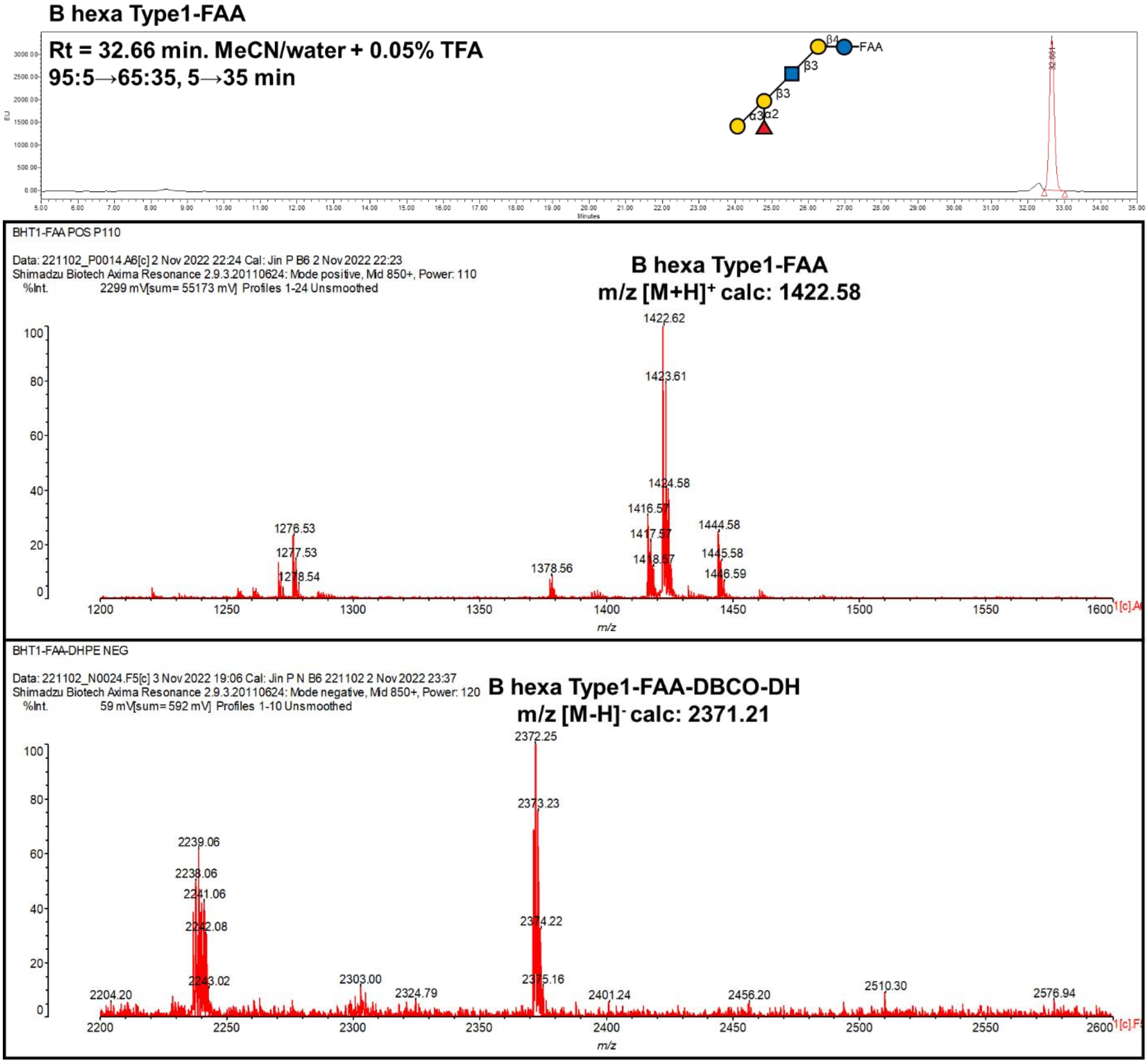
B hexa Type1-FAA
Rt = 32.66 min. MeCN/water + 0.05% TFA
95:5→65:35, 5→35 min
BHT1-FAA POS P110
Data: 221102_P0014.A6[c] 2 Nov 2022 22:24 Cal: Jin P B6 2 Nov 2022 22:23
Shimadzu Biotech Axima Resonance 2.9.3.20110624: Mode positive, Mid 850+, Power: 110
%Int. 2299 mV[sum= 55173 mV] Profiles 1-24 Unsmoothed
B hexa Type1-FAA
m/z [M+H]+ calc: 1422.58
1422.62
1423.61
1424.58
1416.57
1444.58
1276.53
1277.53
1378.56
m/z
BHT1-FAA-DHPE NEG
Data: 221102_N0024.F5[c] 3 Nov 2022 19:06 Cal: Jin P N B6 221102 2 Nov 2022 23:37
Shimadzu Biotech Axima Resonance 2.9.3.20110624: Mode negative, Mid 850+, Power: 120
%Int. 59 mV[sum= 592 mV] Profiles 1-10 Unsmoothed
B hexa Type1-FAA-DBCO-DH
m/z [M-H]- calc: 2371.21
2372.25
2373.23
2374.22
2375.16
2239.06
2238.06
2241.06
2242.08
2243.02
2204.20
2303.00
2324.79
2401.24
2456.20
2510.30
2576.94
m/z

Probe No 4

B hexa Type2-FAA

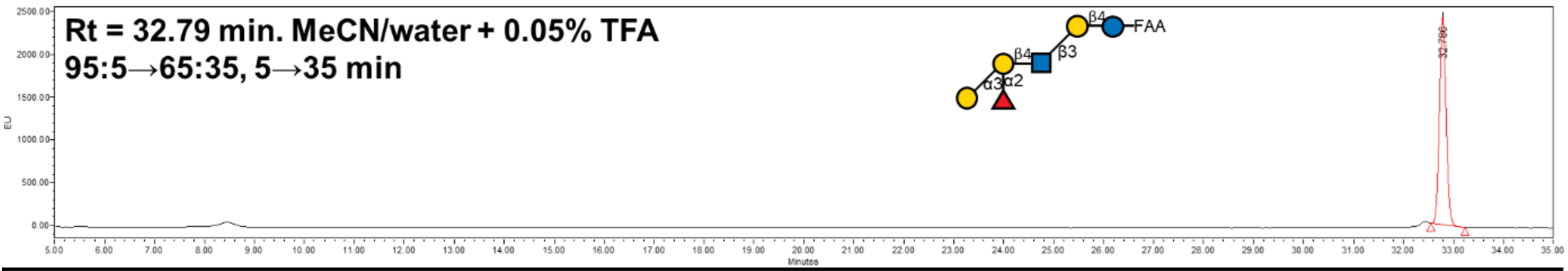

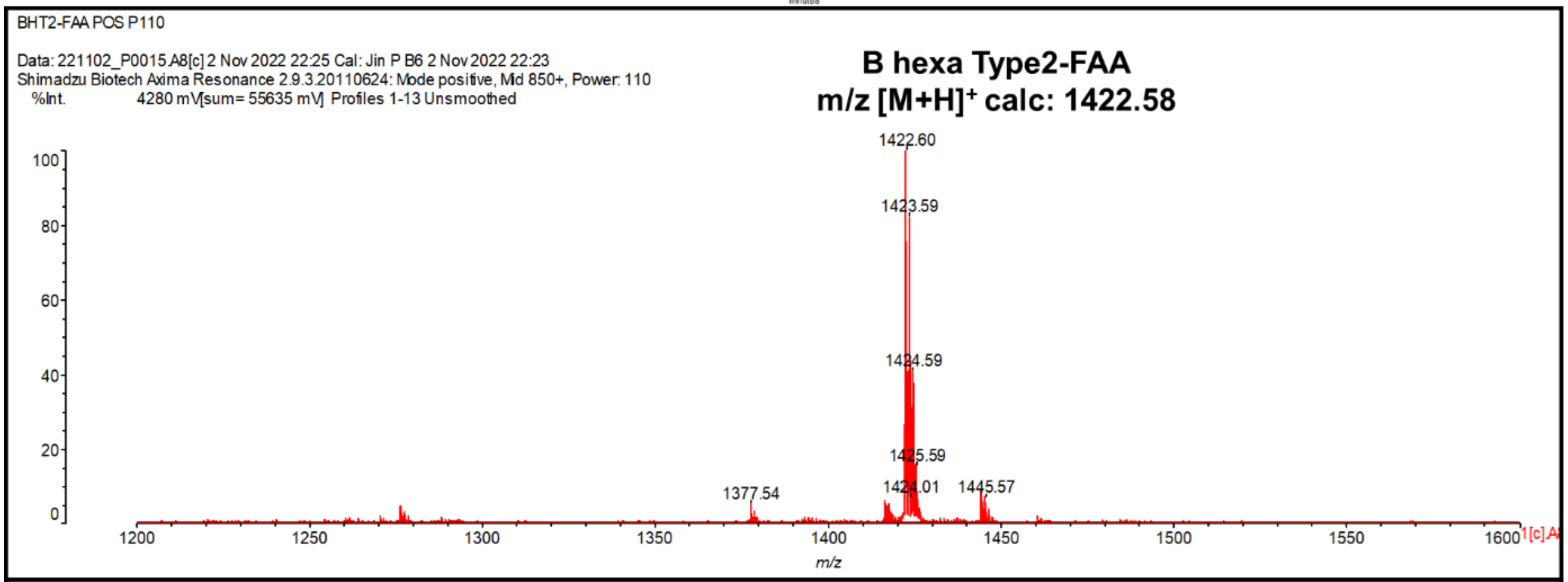

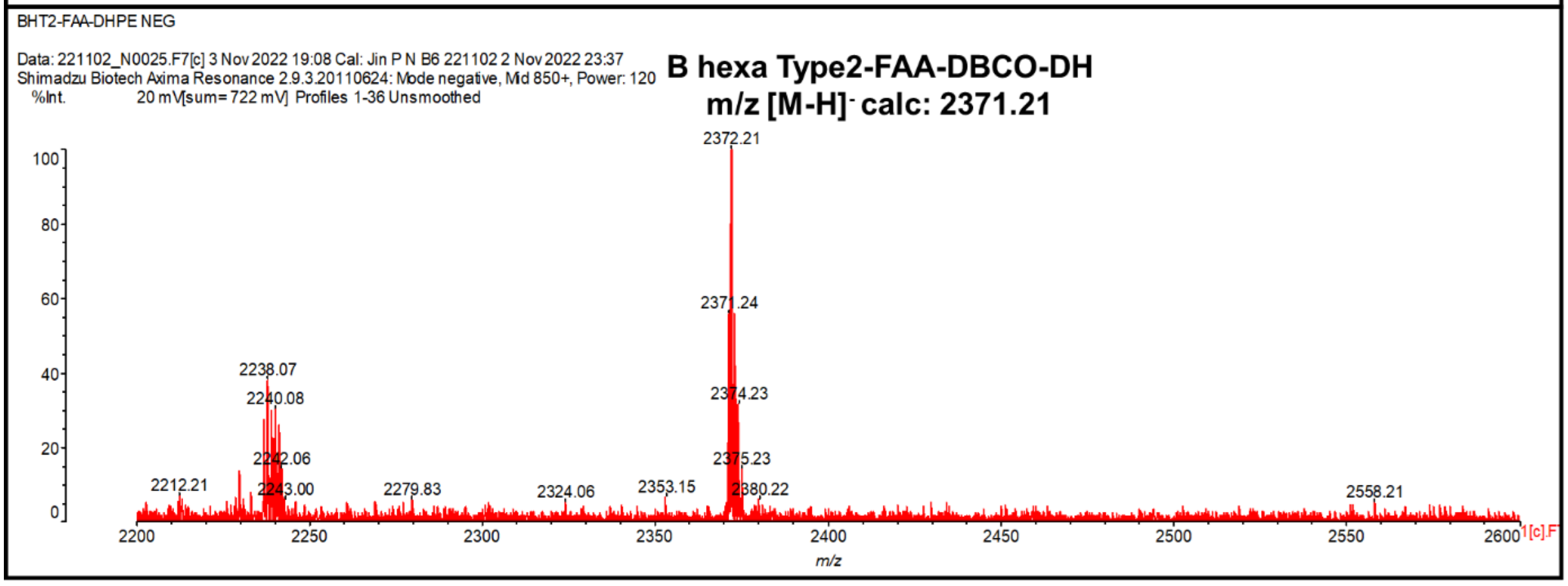

Probe No 5

LNT-FAA

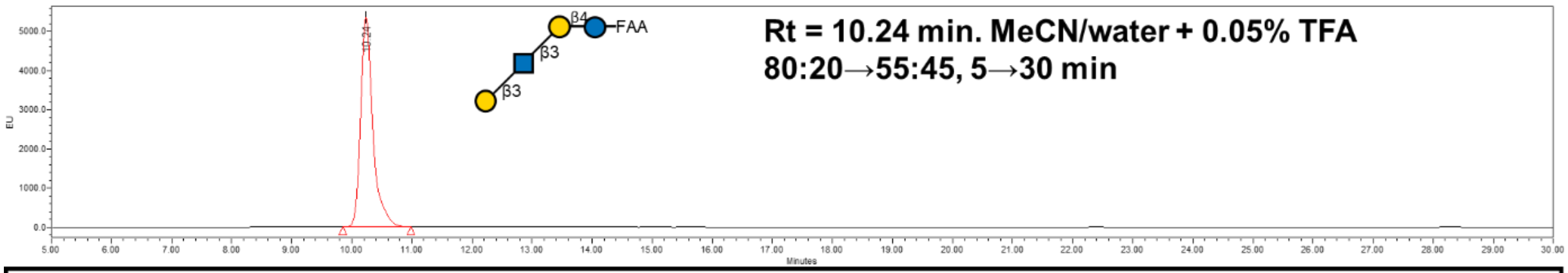

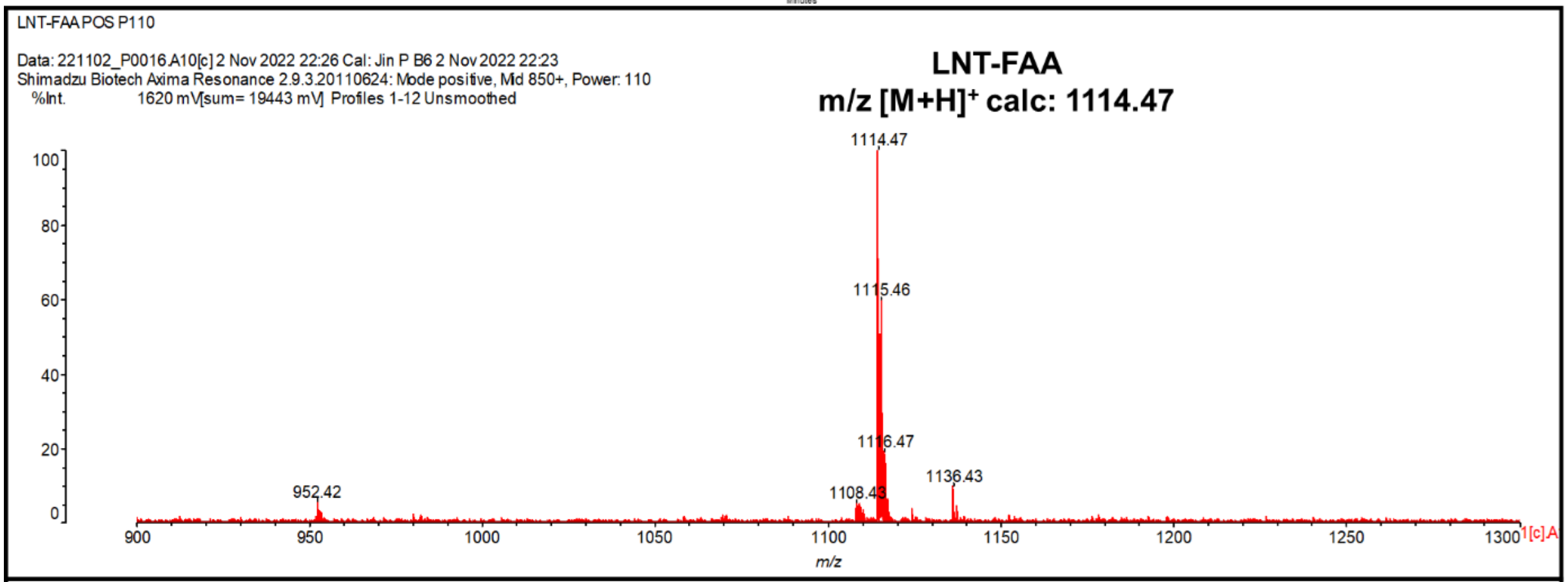

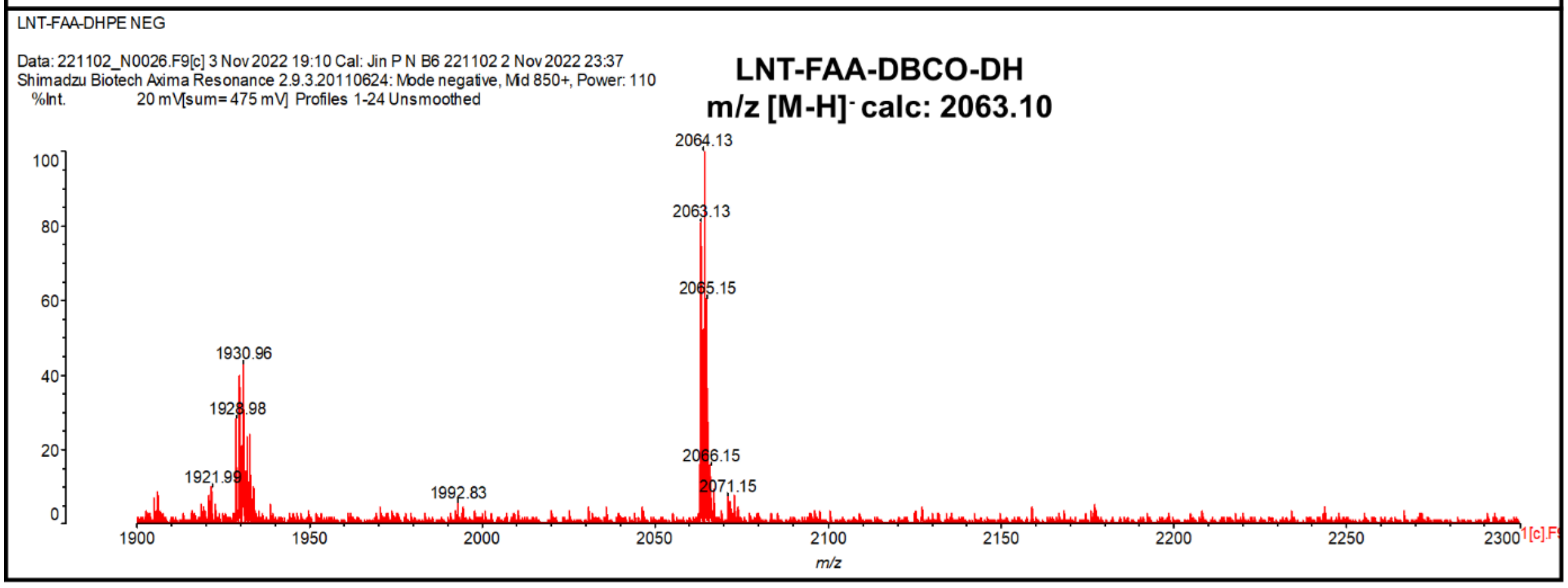

Probe No 6

LNnT-FAA

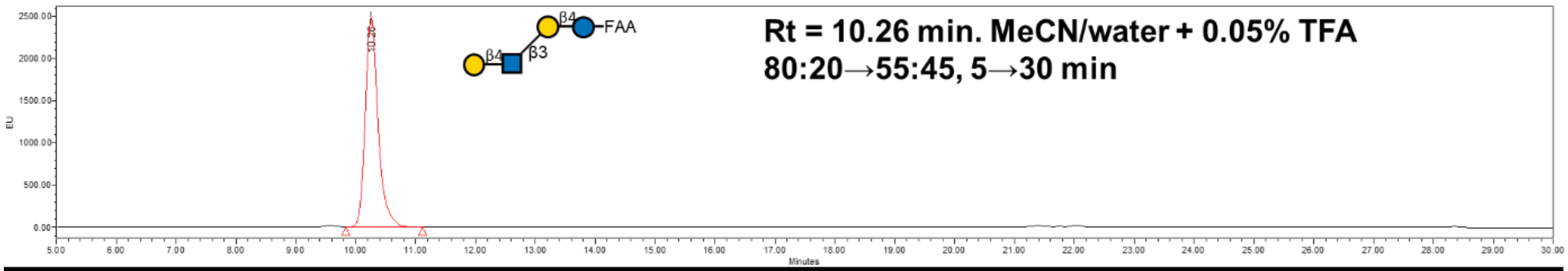

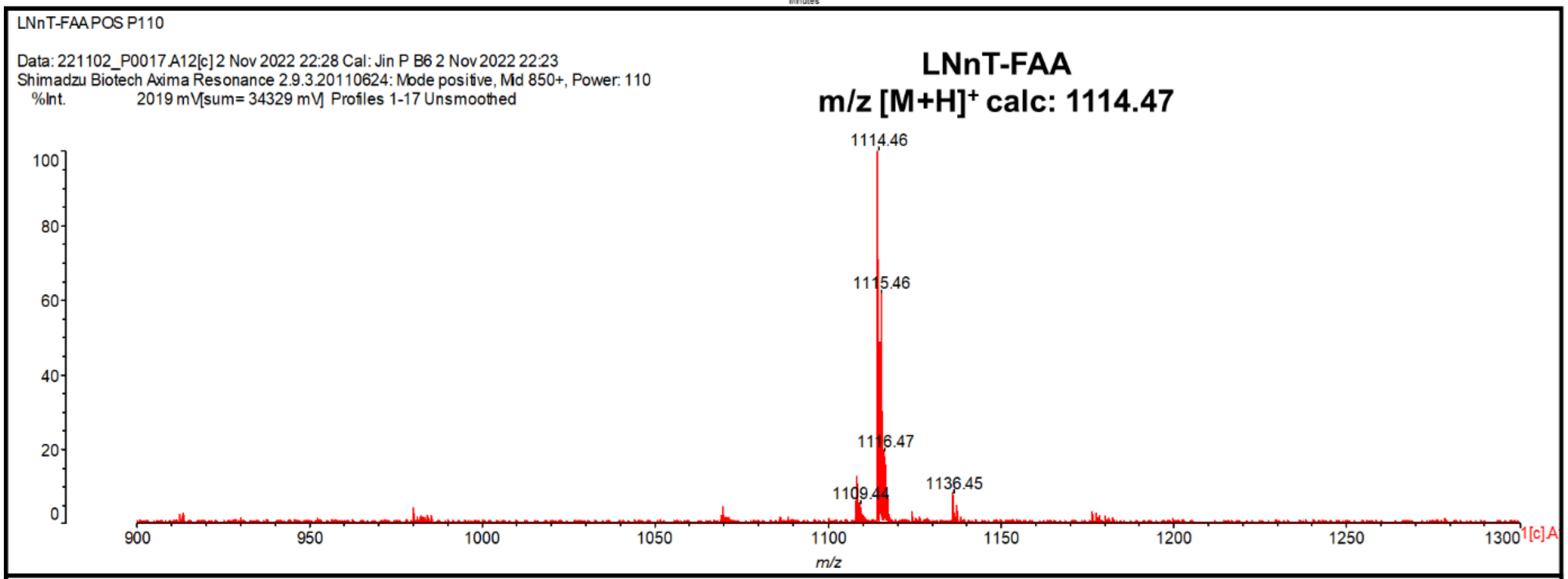

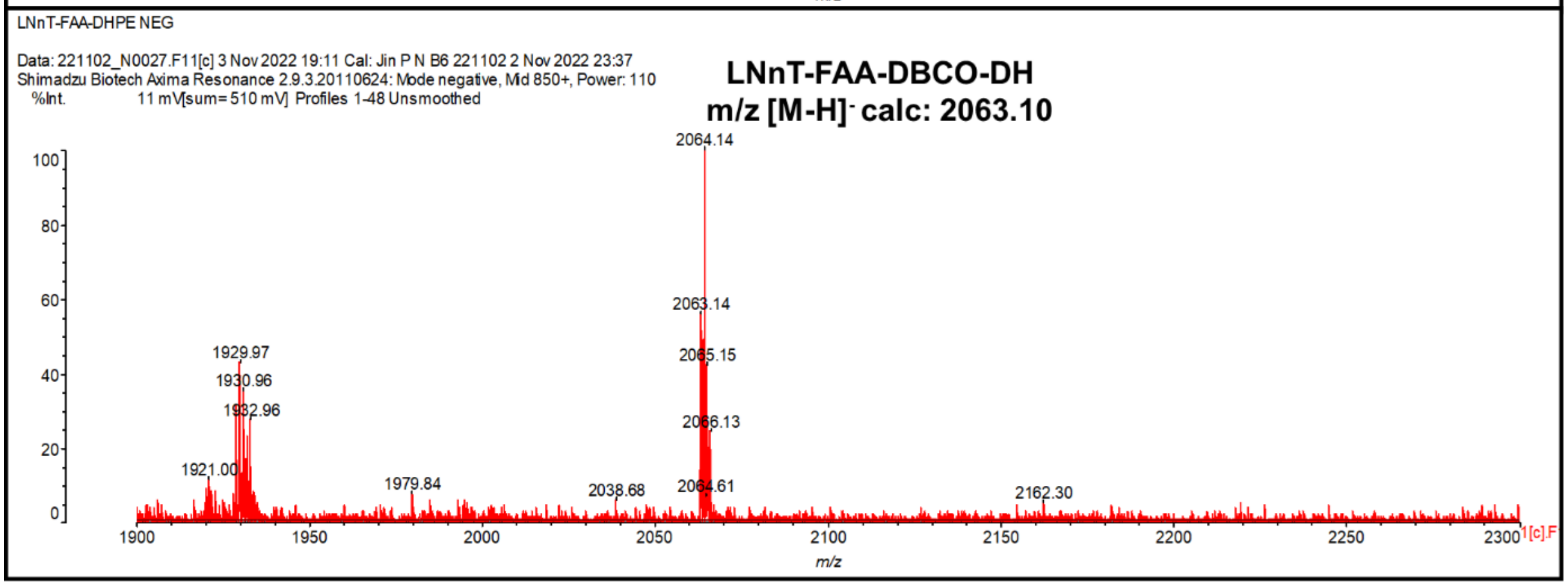

Probe No 7

LN3-FAA

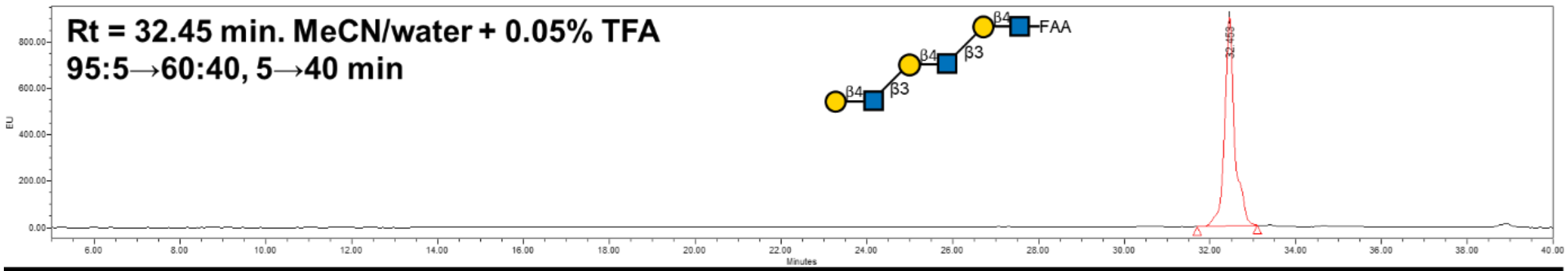

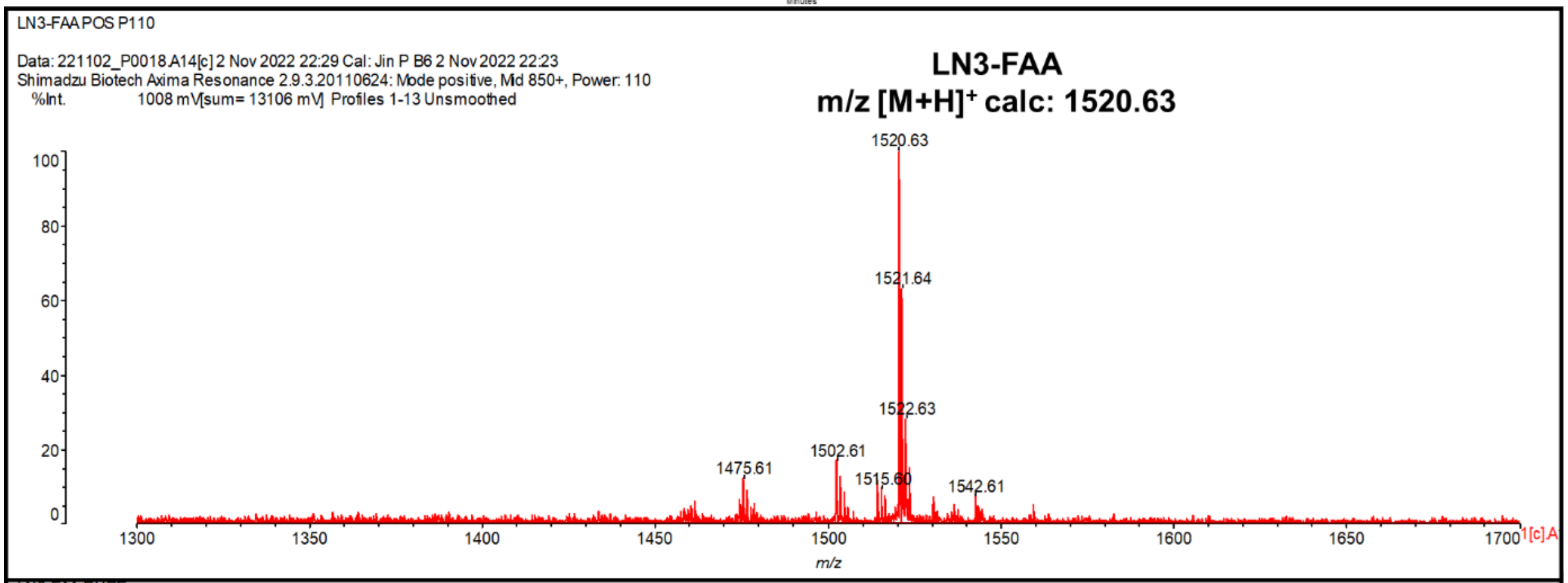

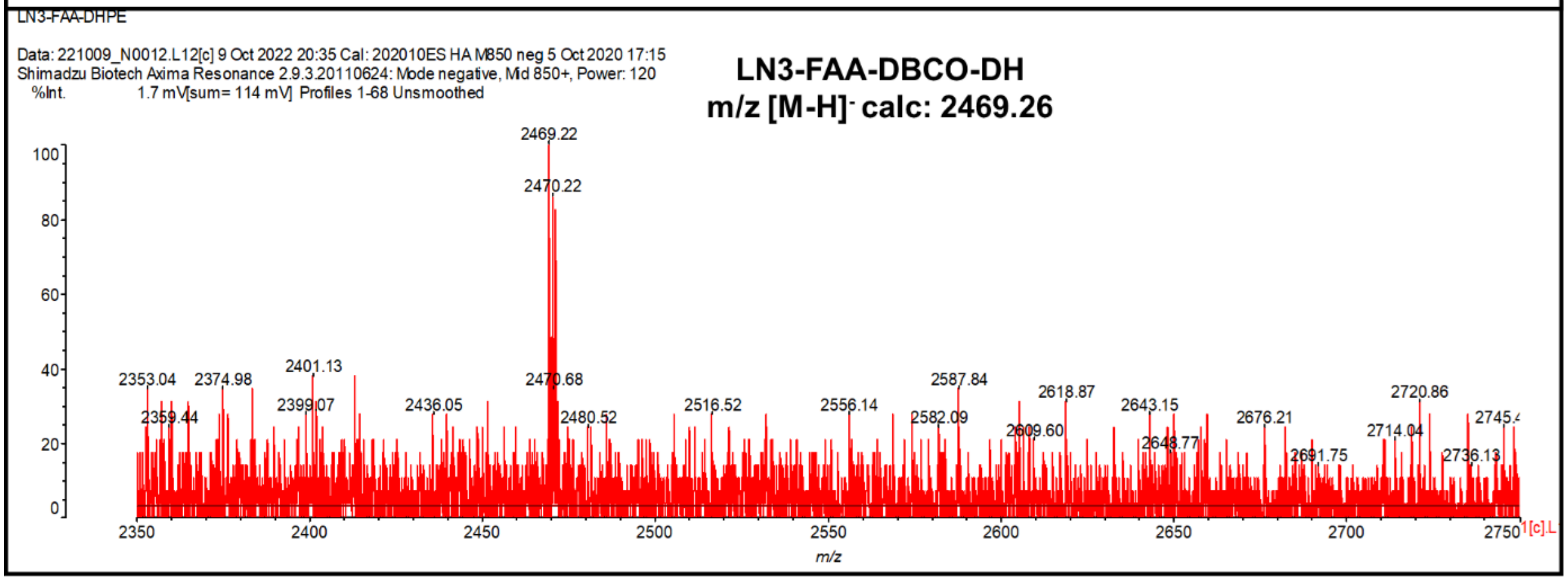

Probe No 8

LNFPI-FAA

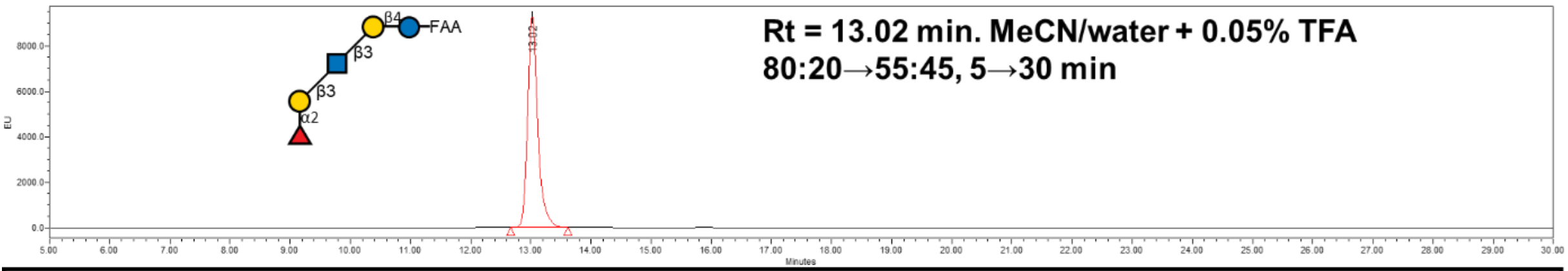

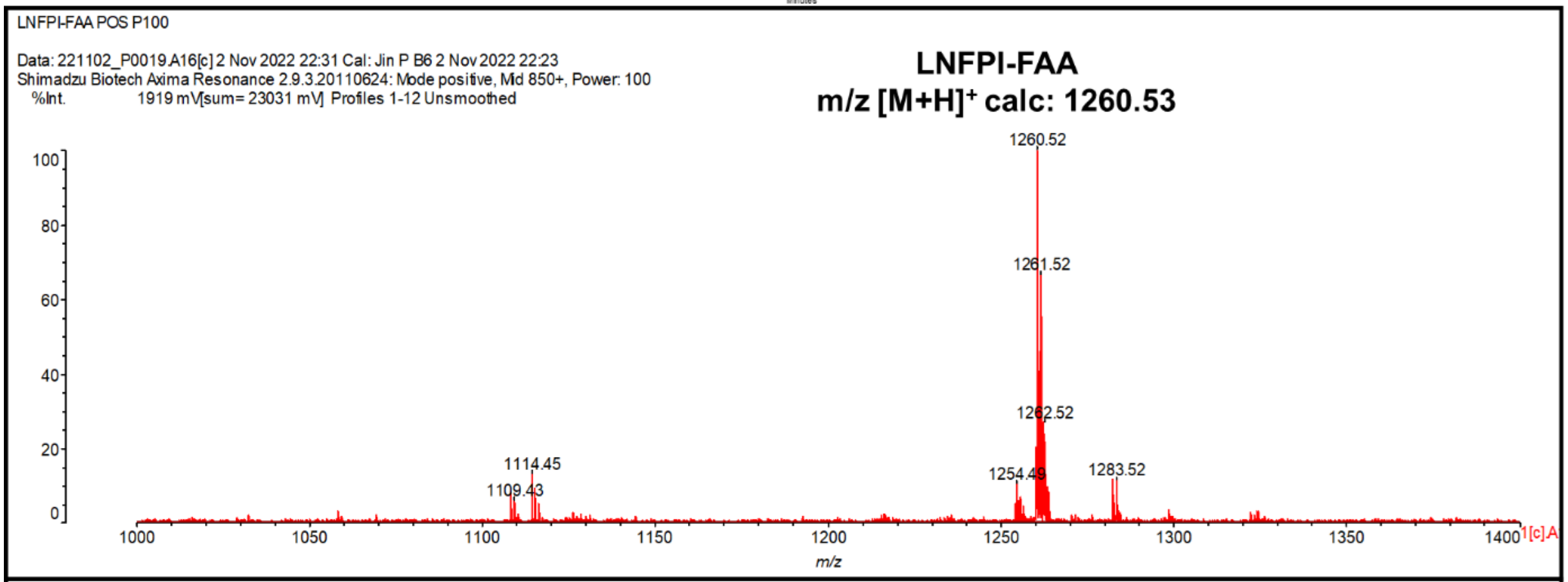

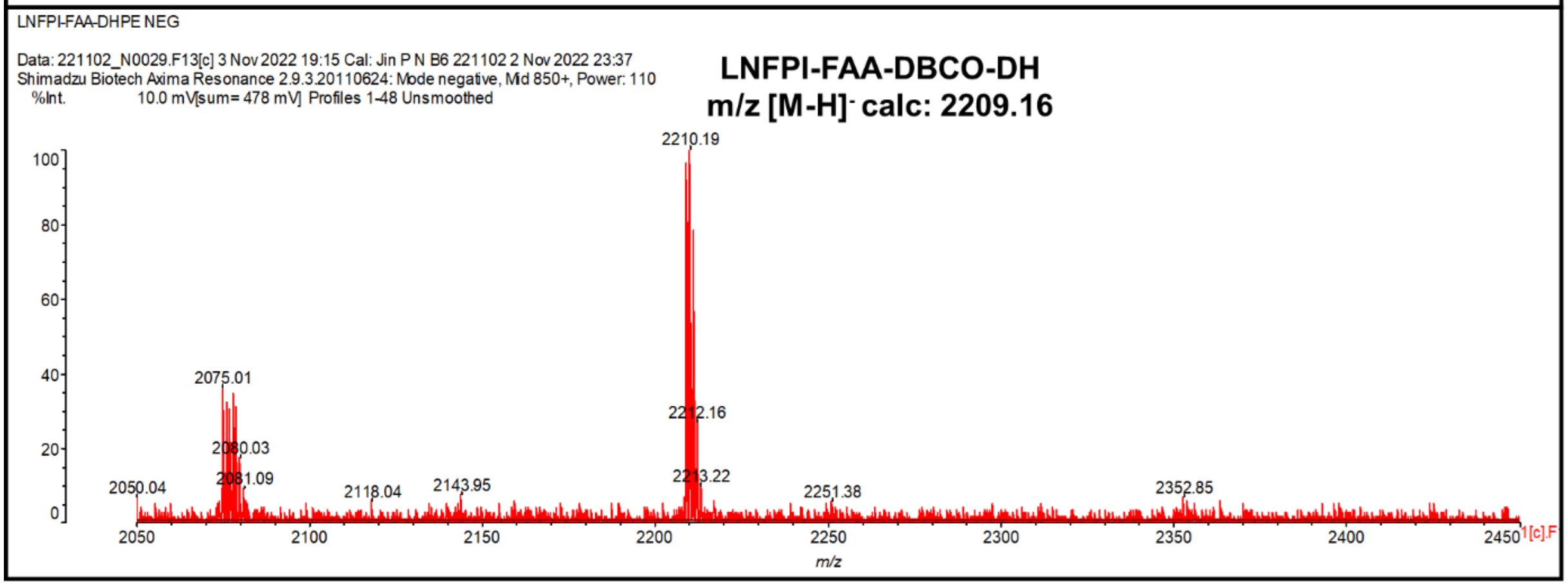

Probe No 9

LNnFPI-FAA

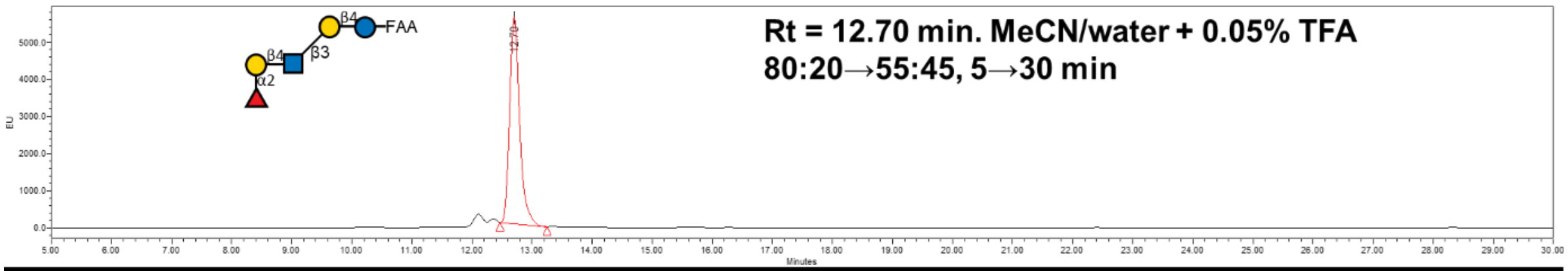

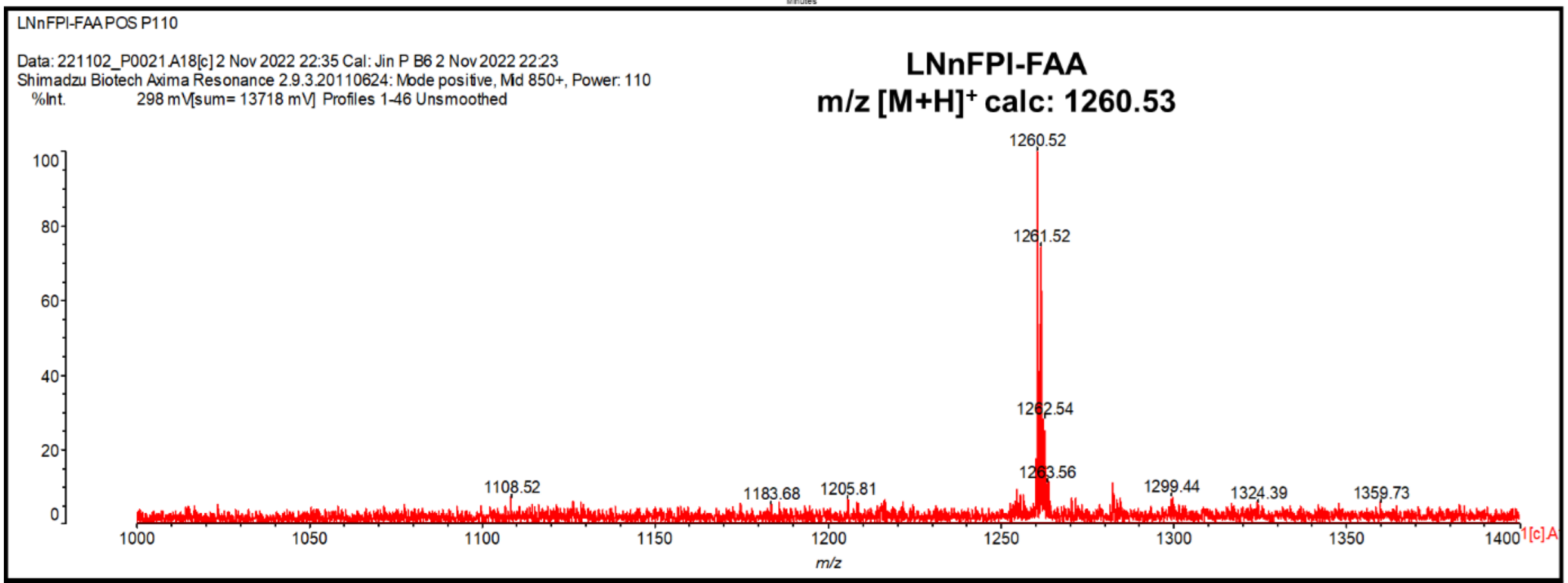

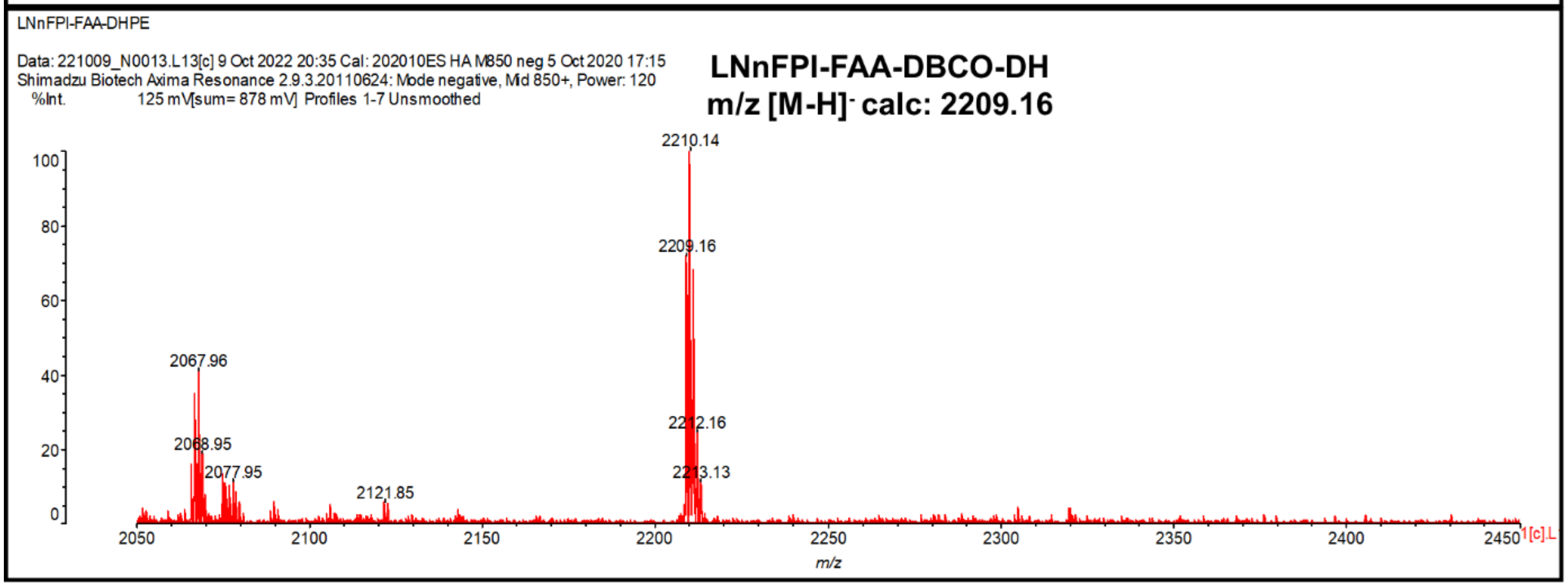

Probe No 10

LNFPII-FAA

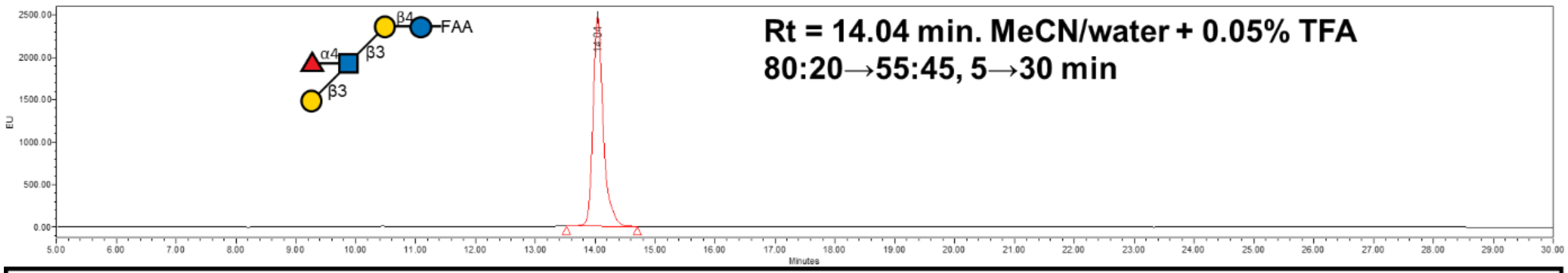

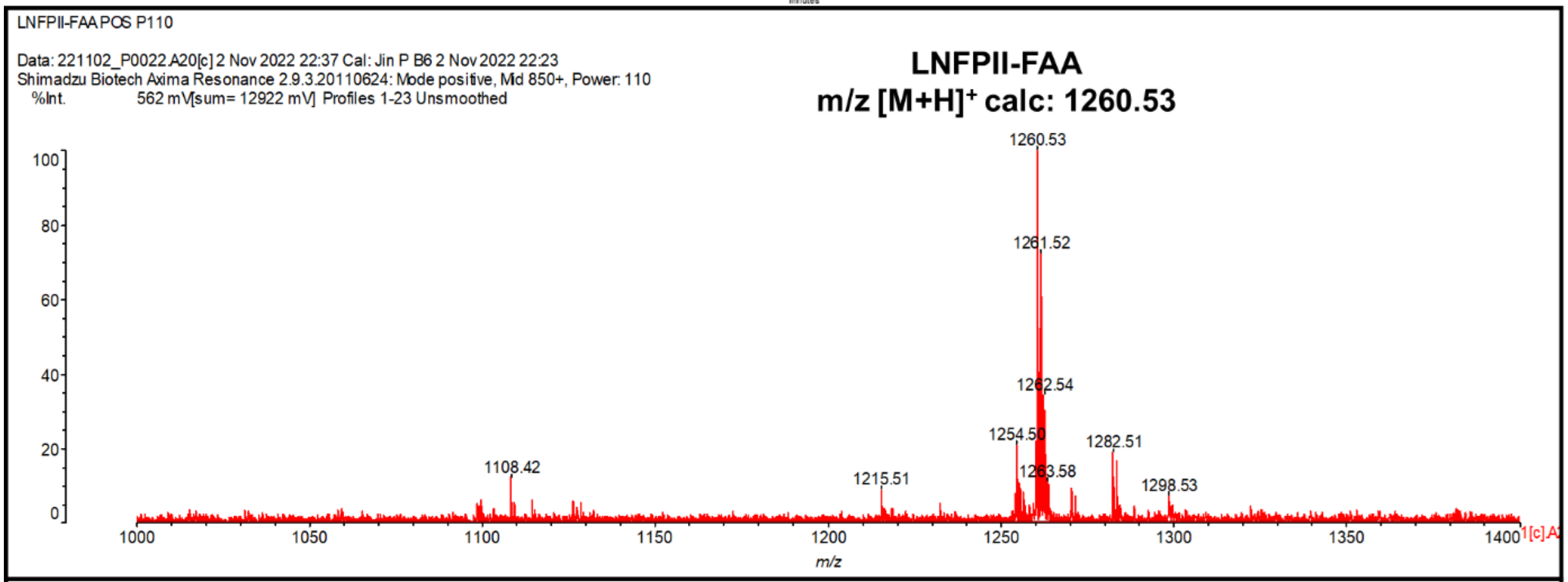

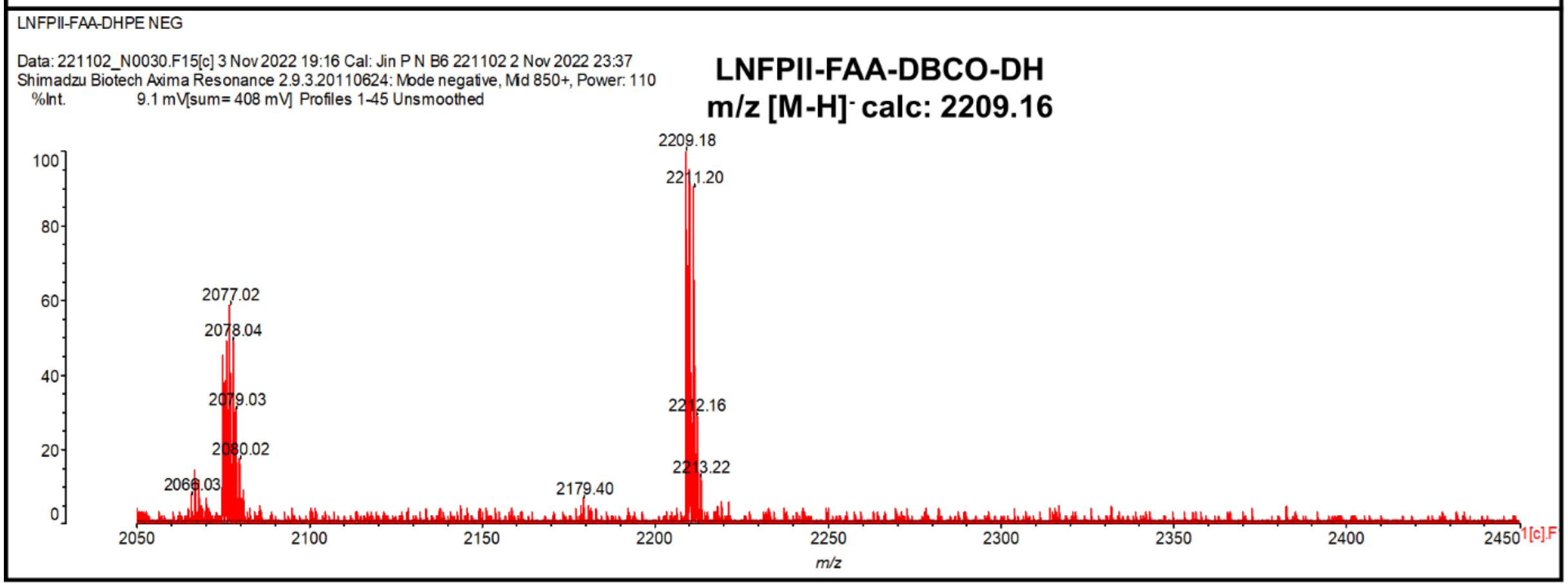

Probe No 11

Leb-FAA

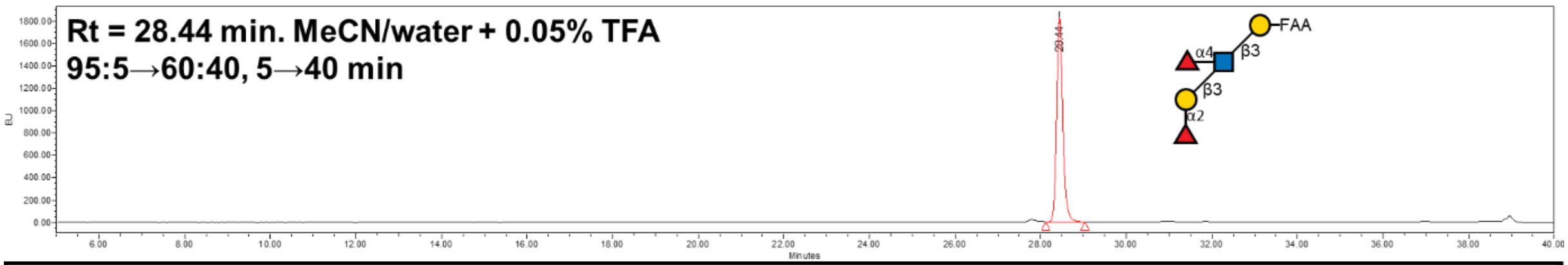

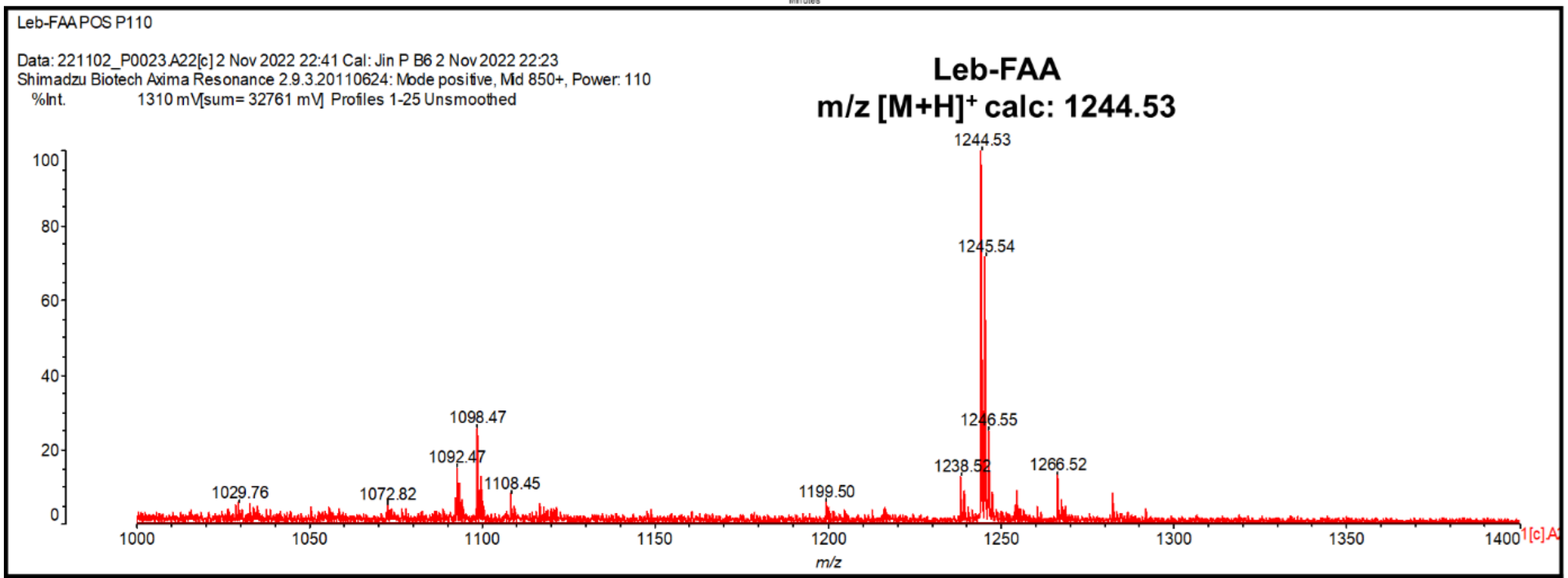

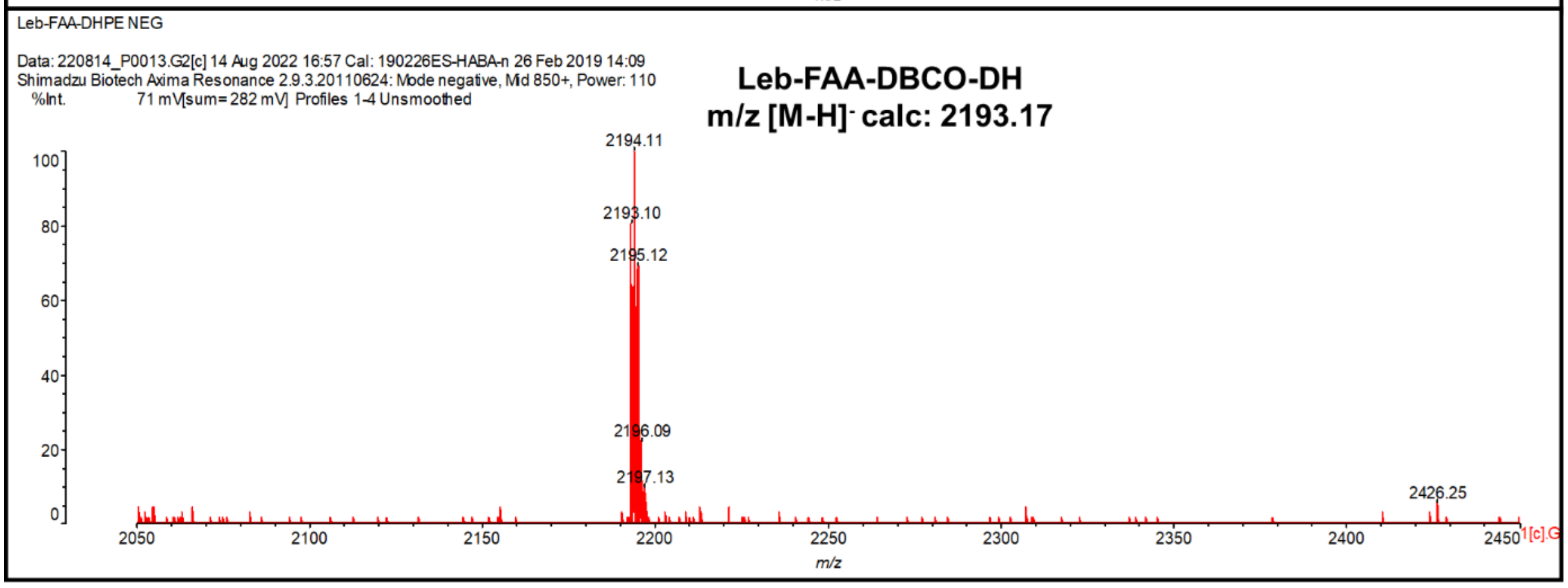

Probe No 12

Lex-FAA

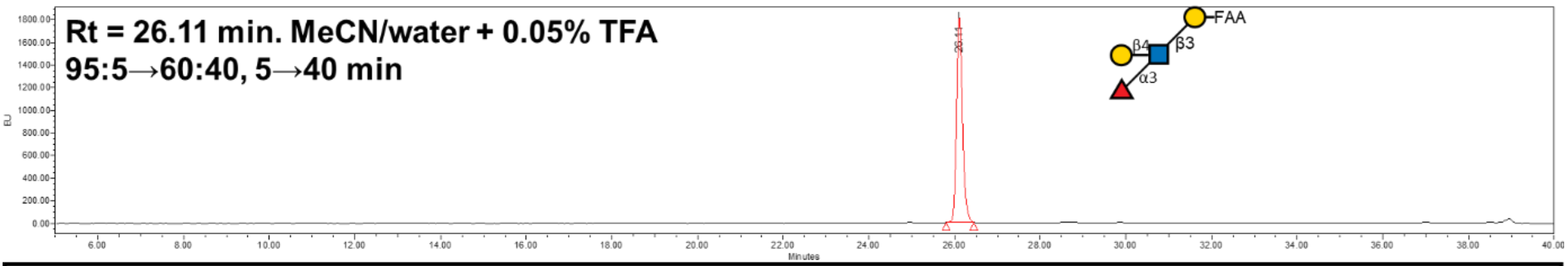

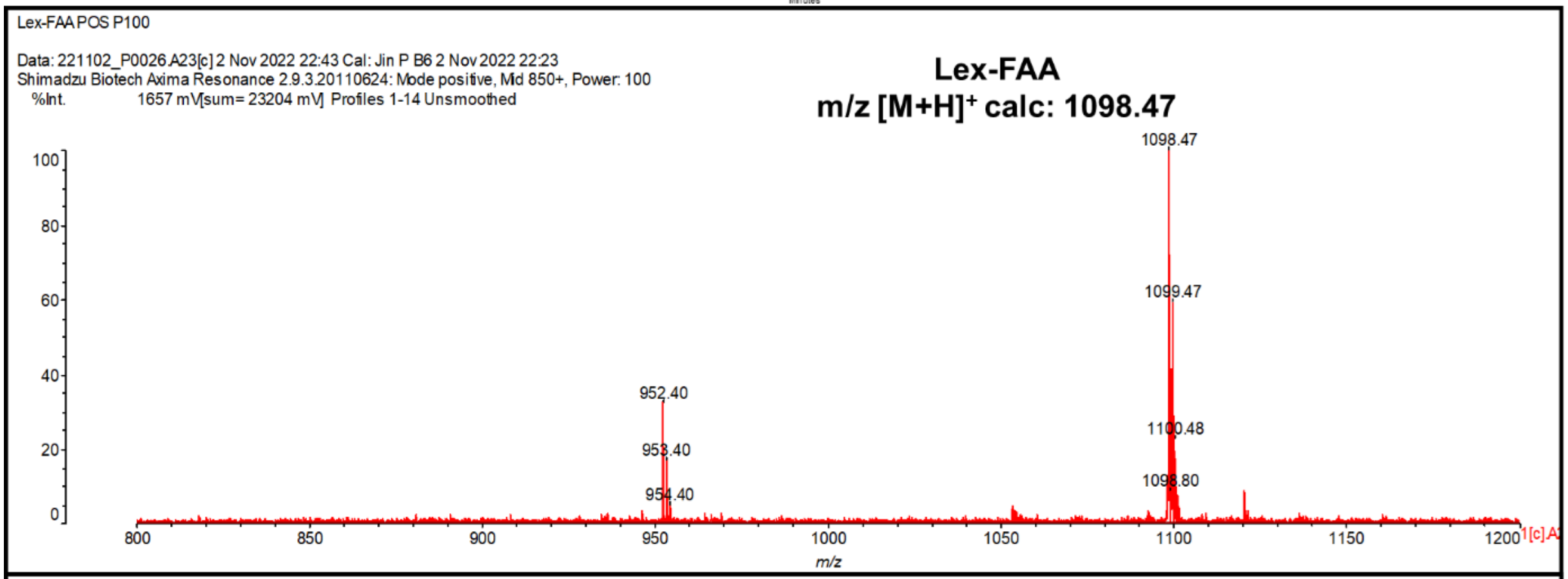

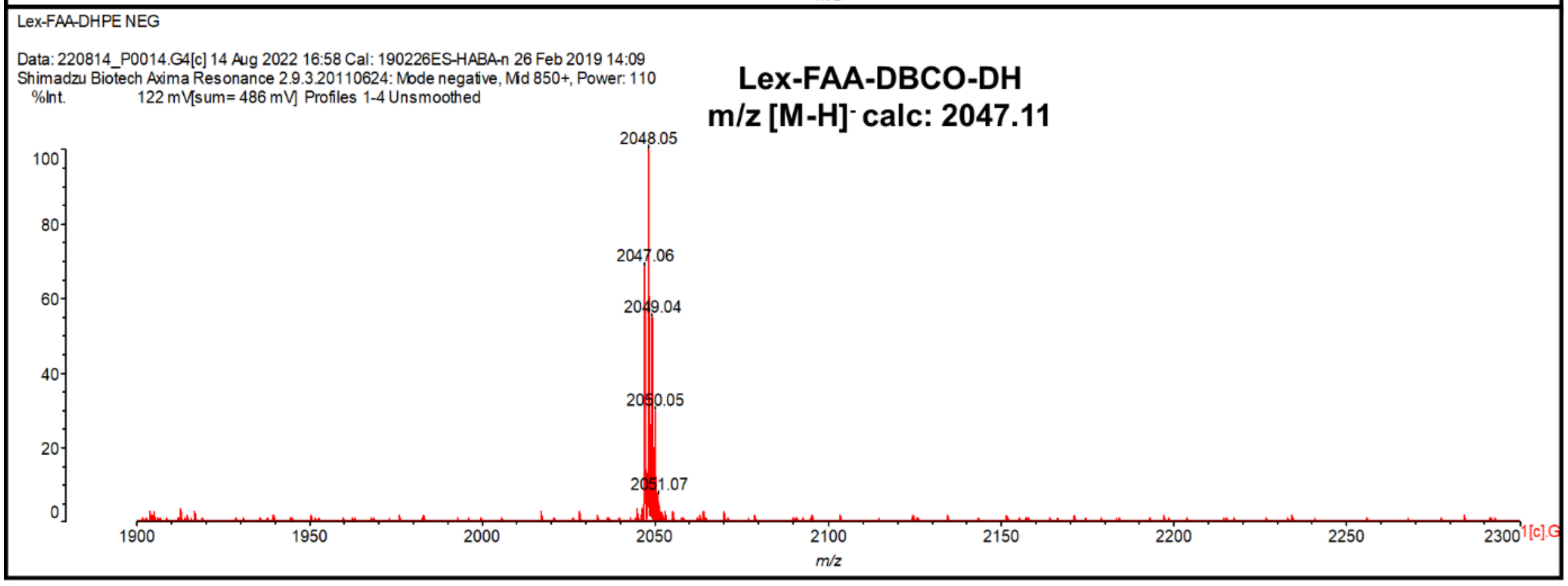

Probe No 13

Ley-FAA

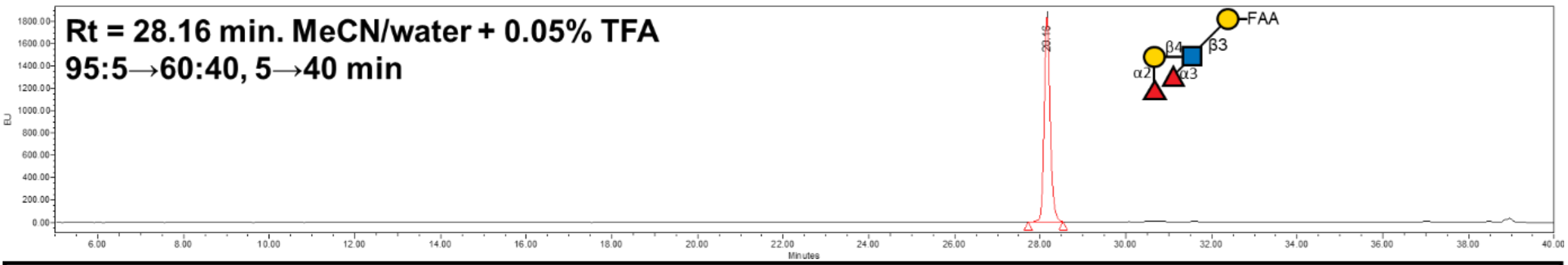

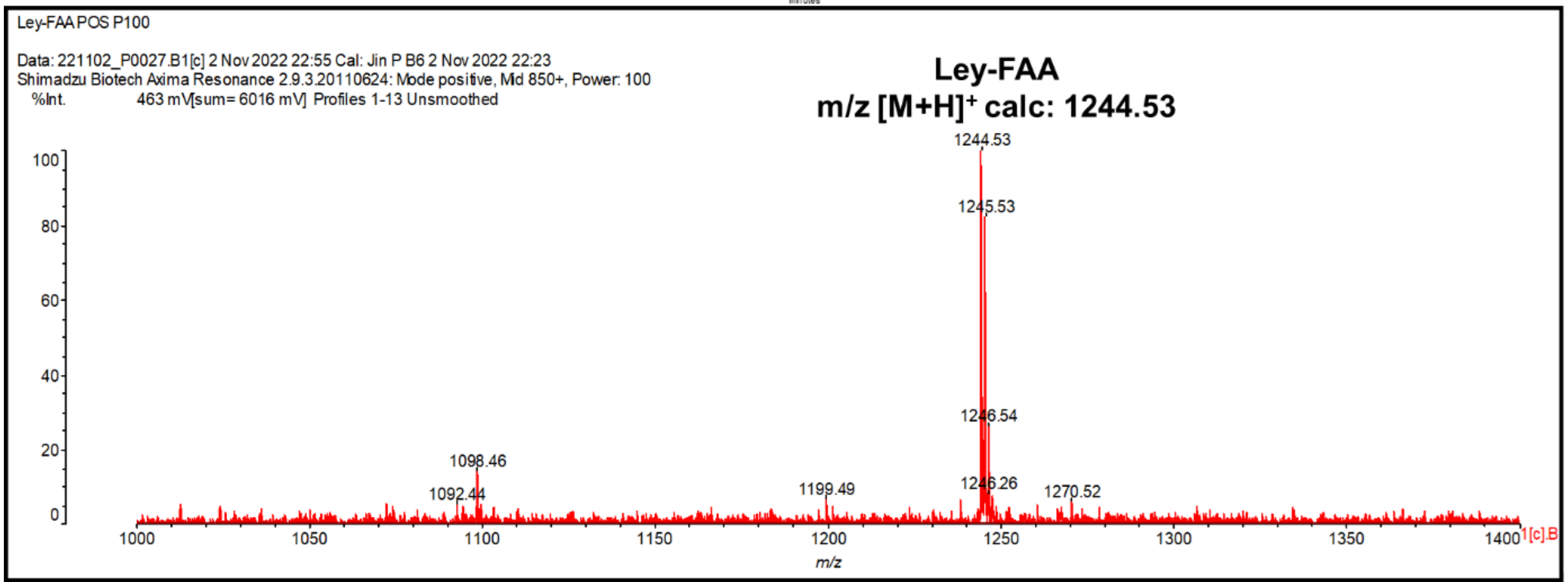

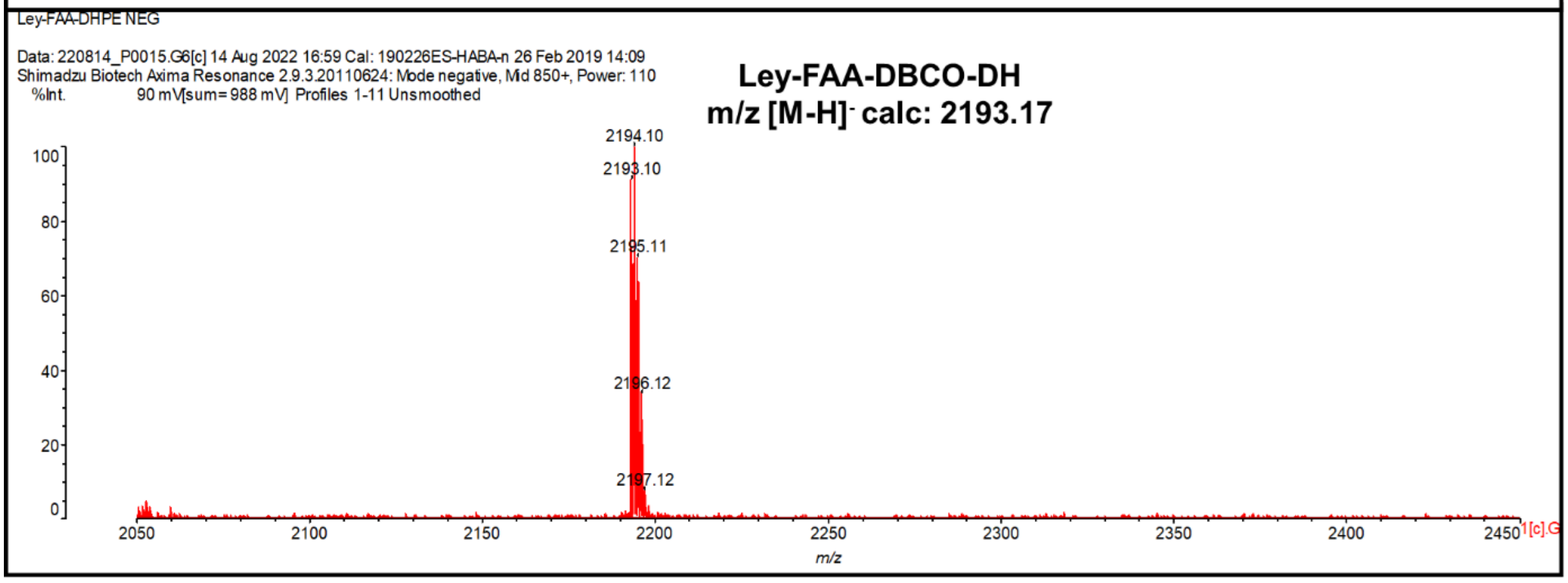

Probe No 14

Forssman antigen penta-FAA

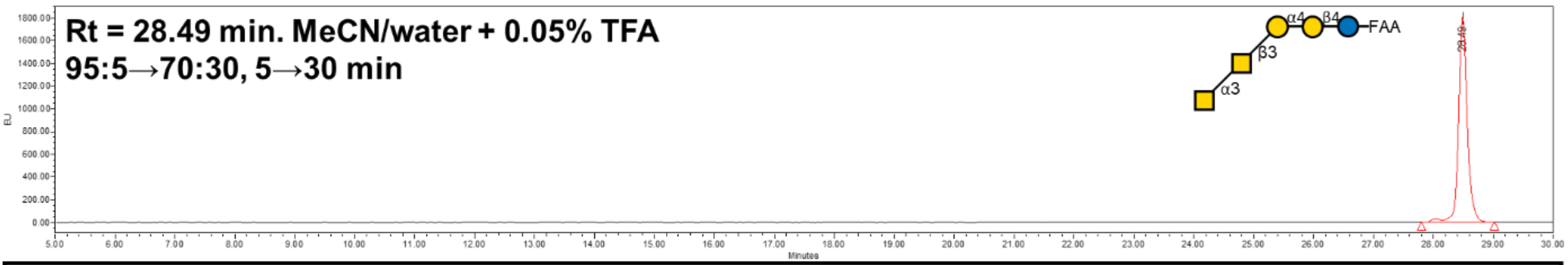

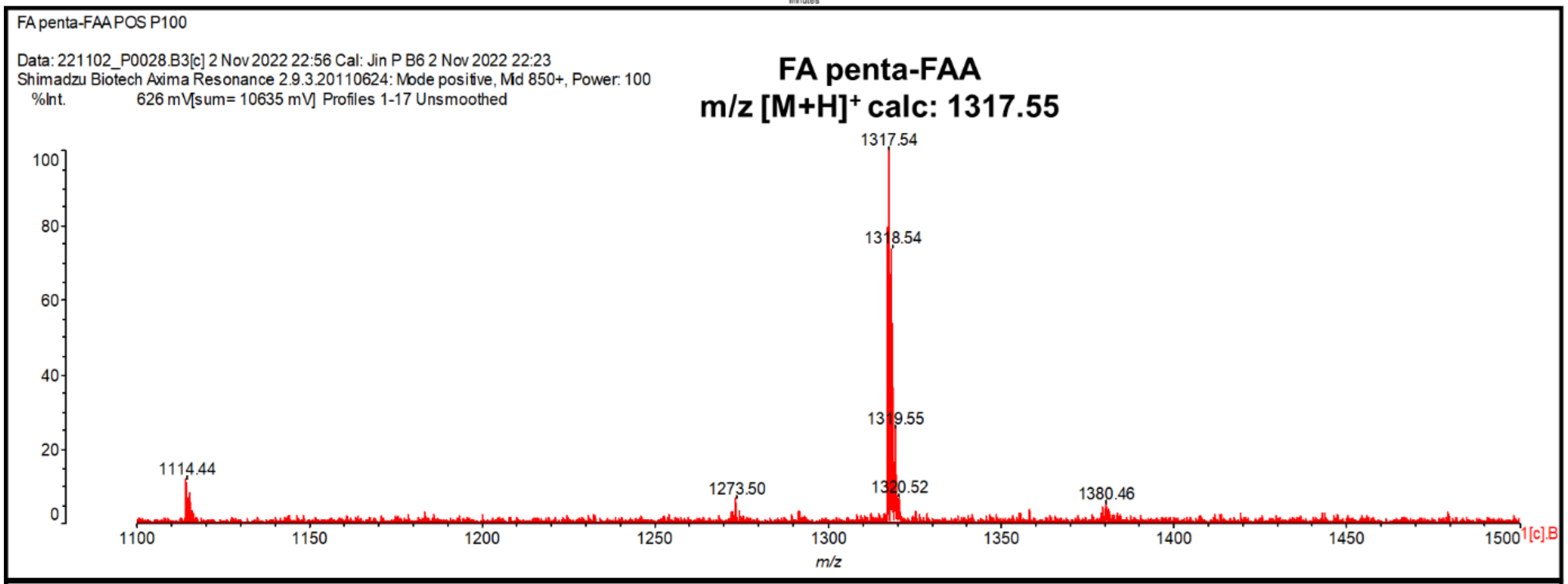

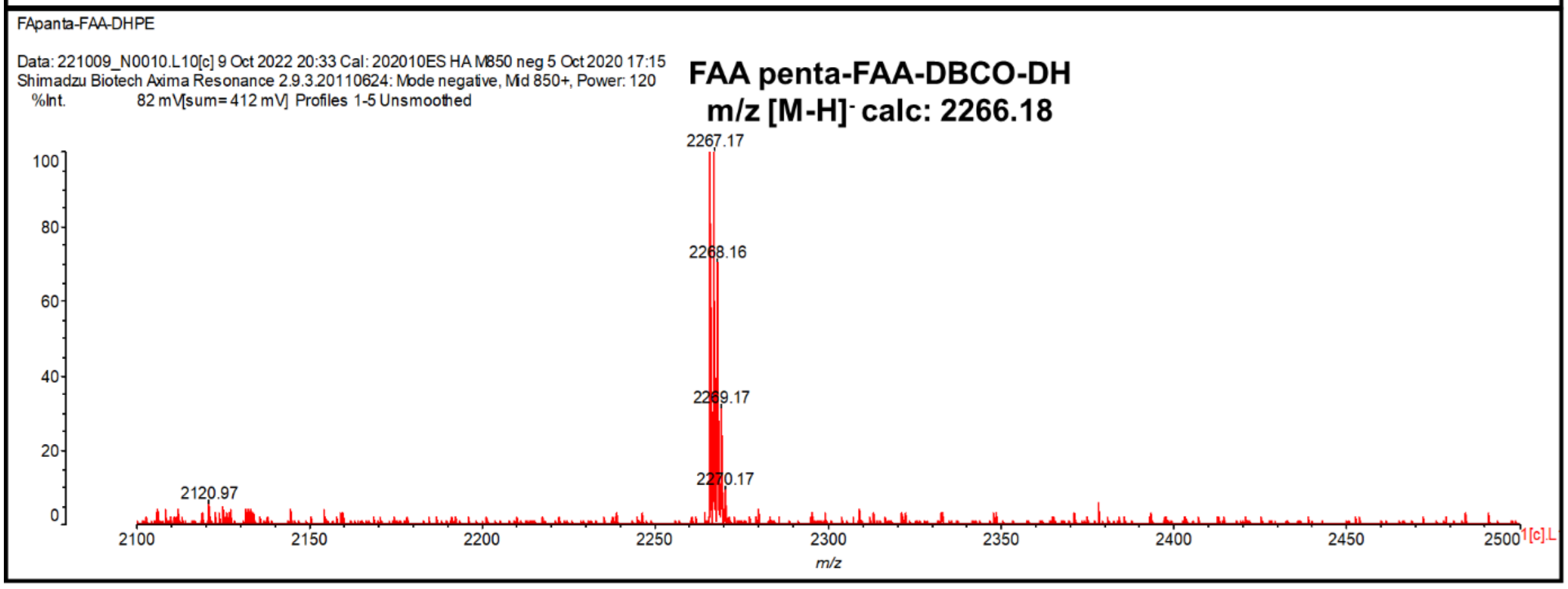

Probe No 15

Globo3-FAA

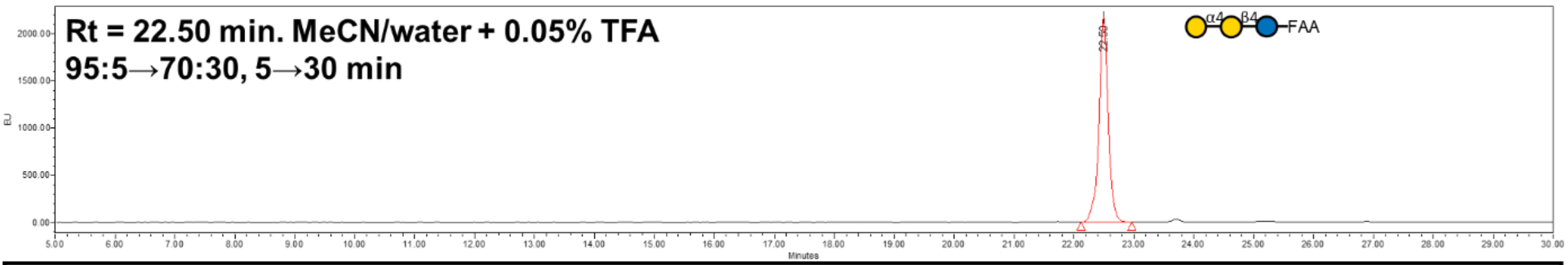

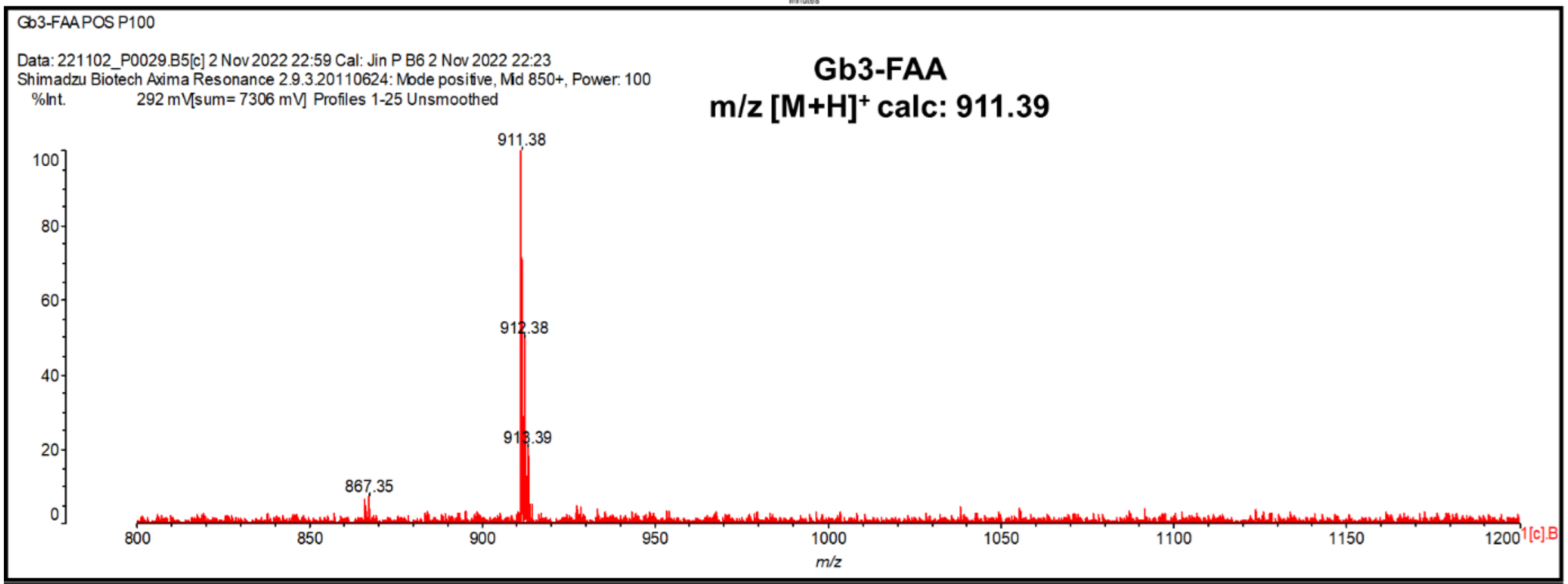

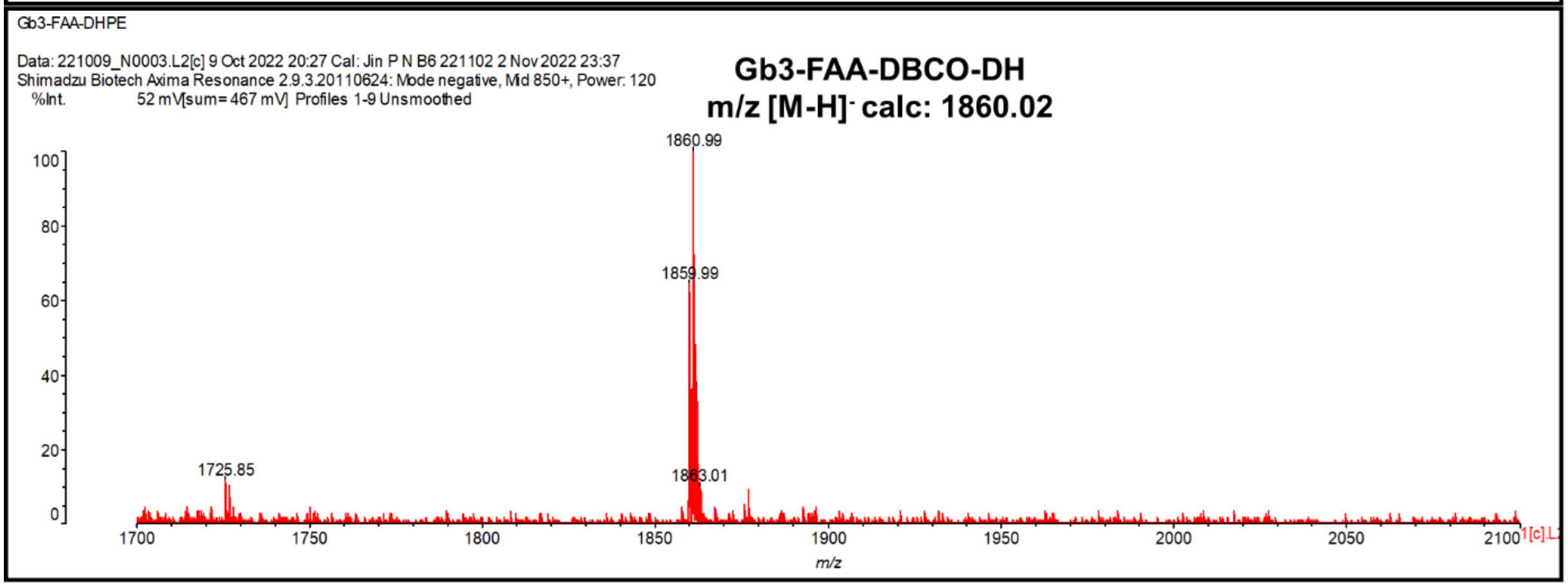

Probe No 16

Globo4-FAA

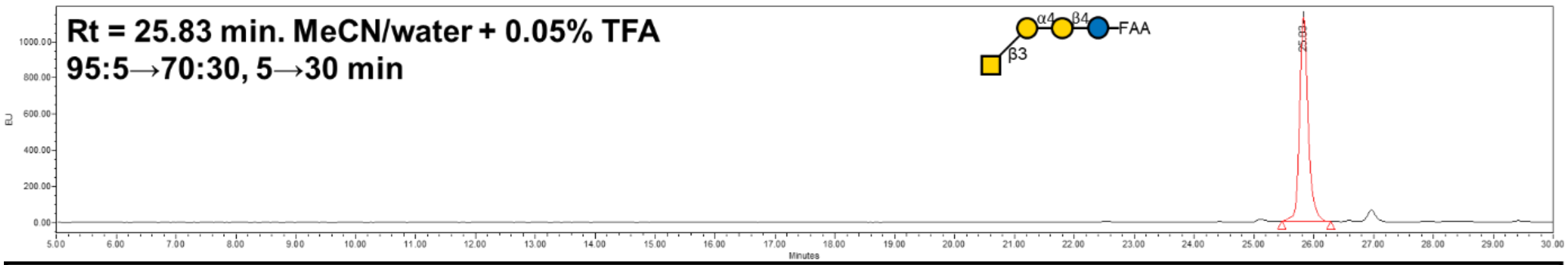

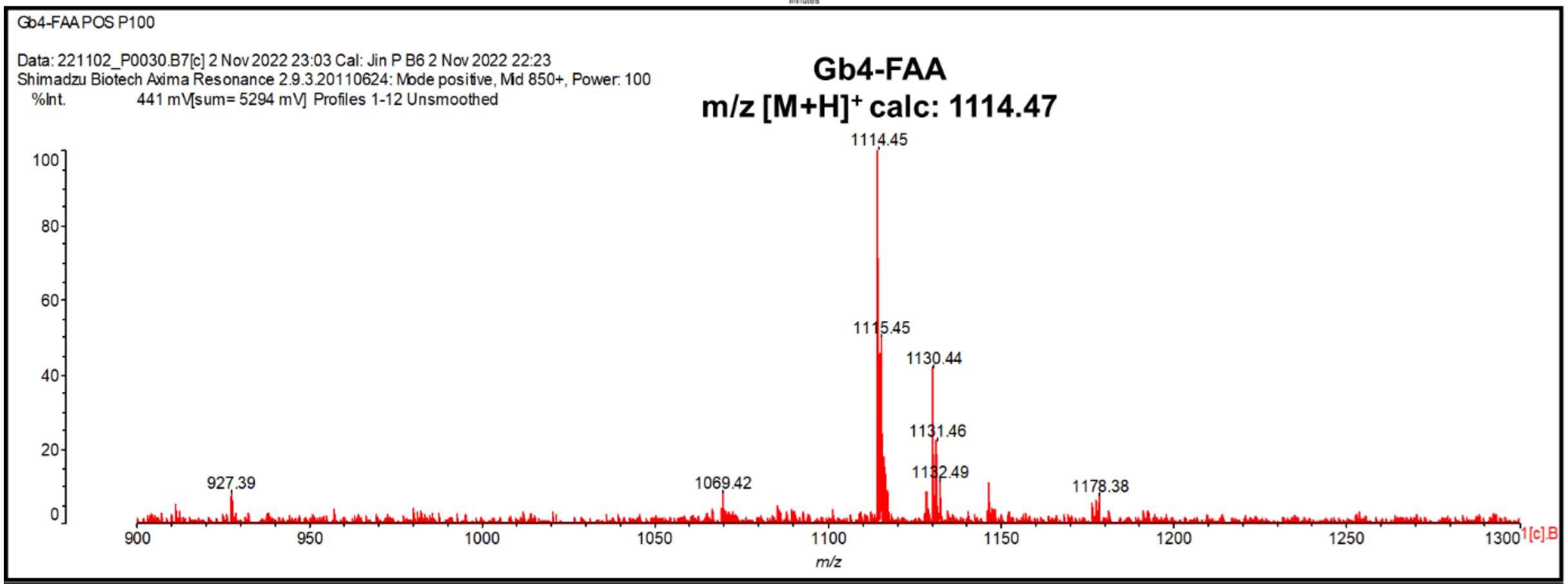

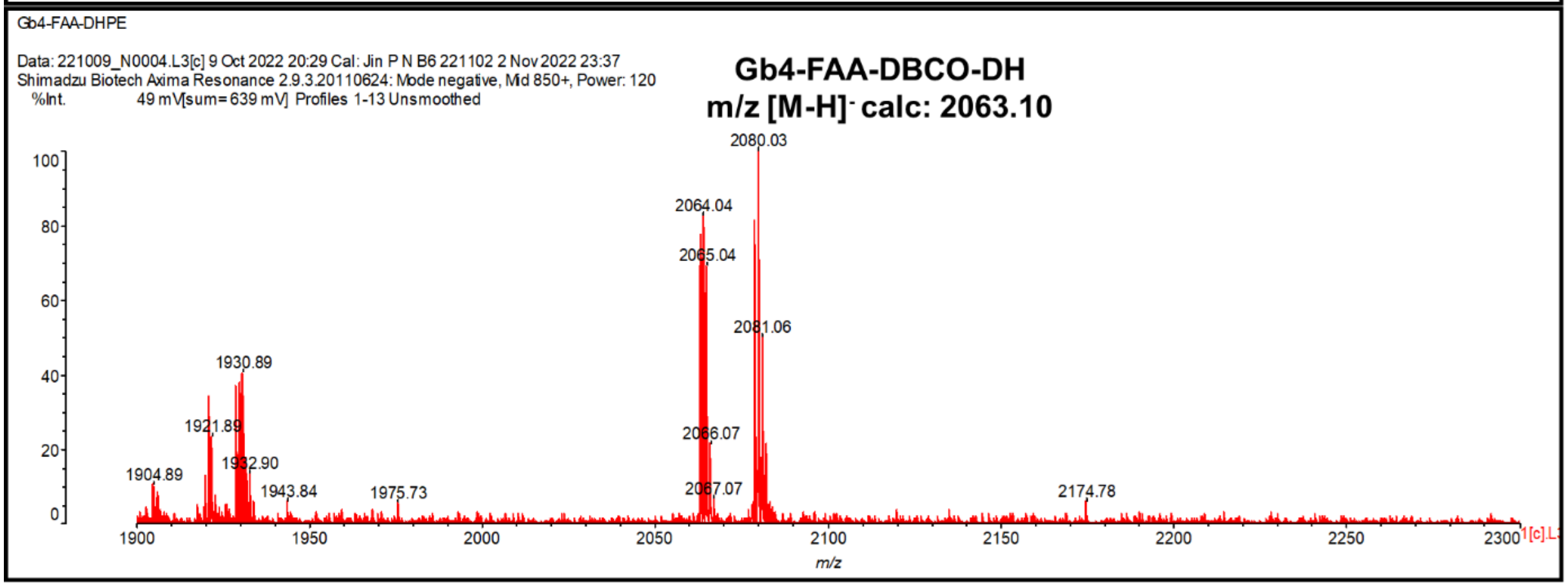

Probe No 17

Globo5-FAA

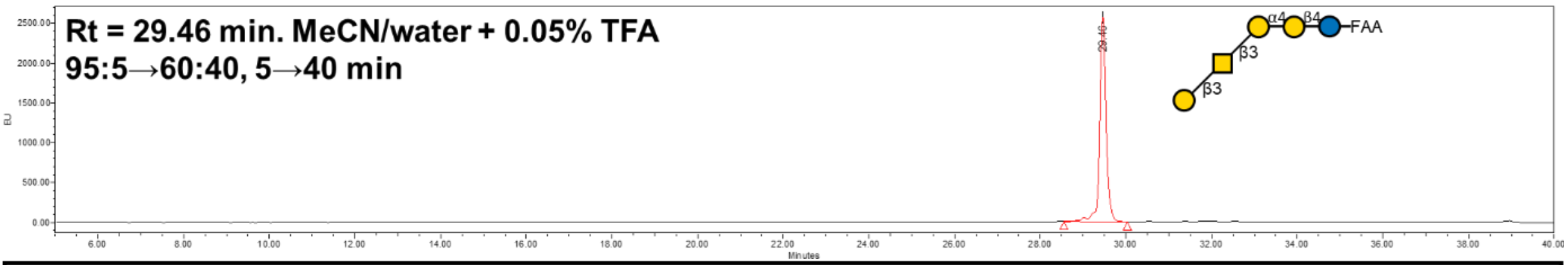

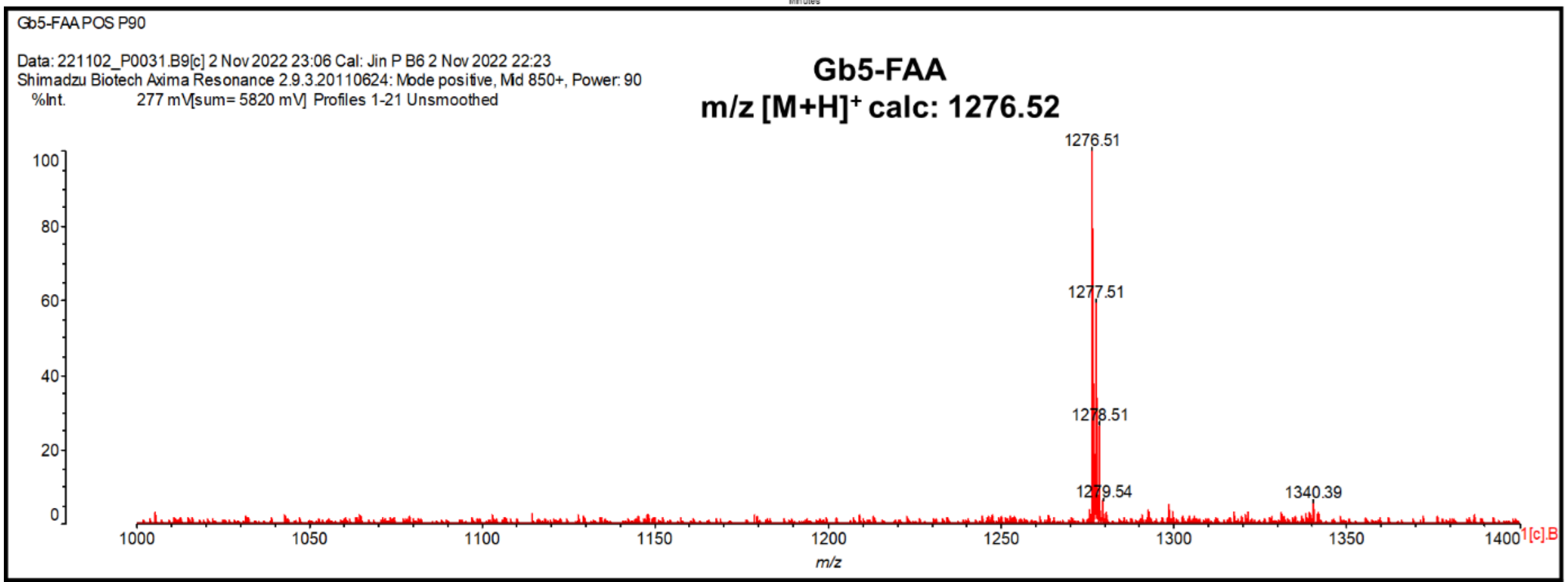

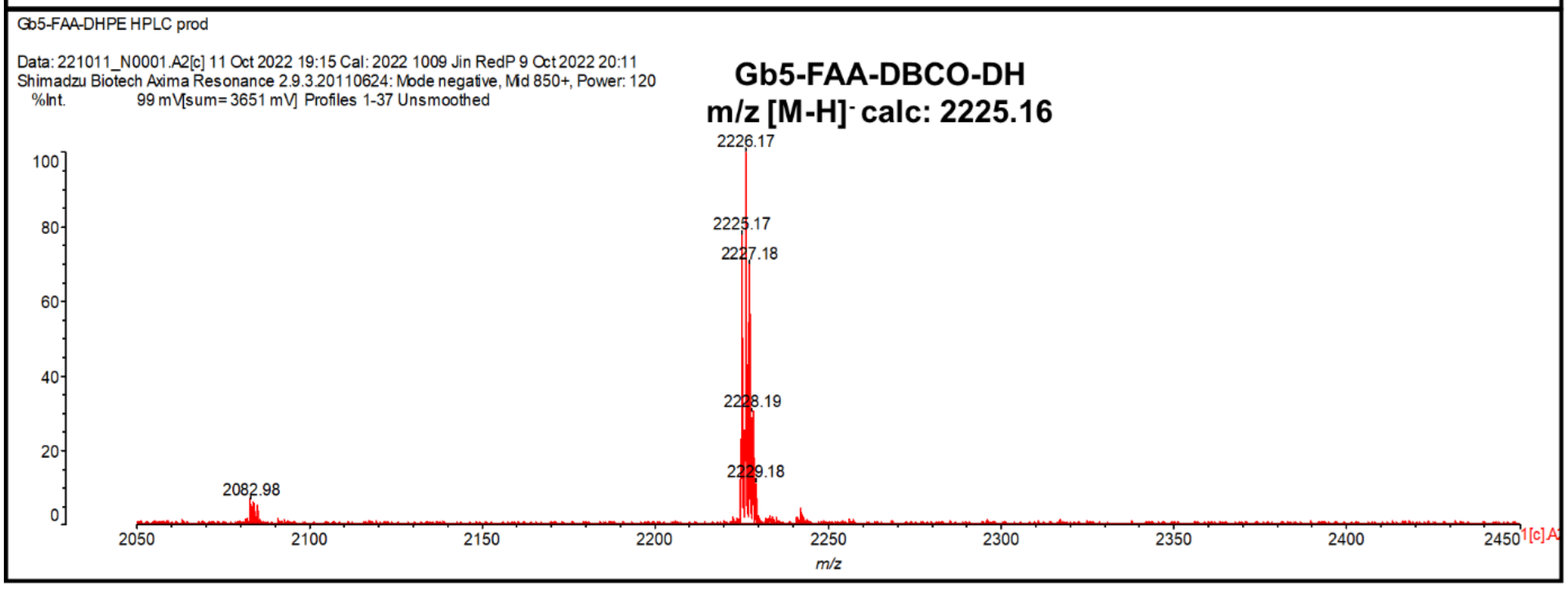

Probe No 18

GloboH-FAA

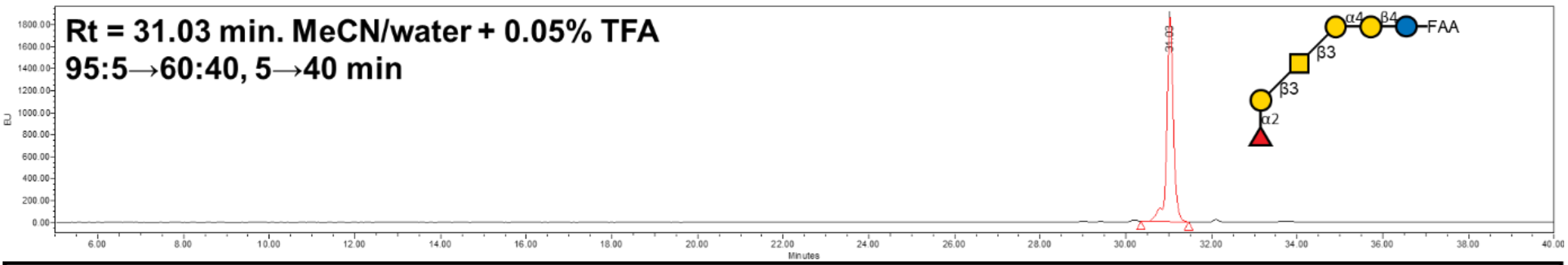

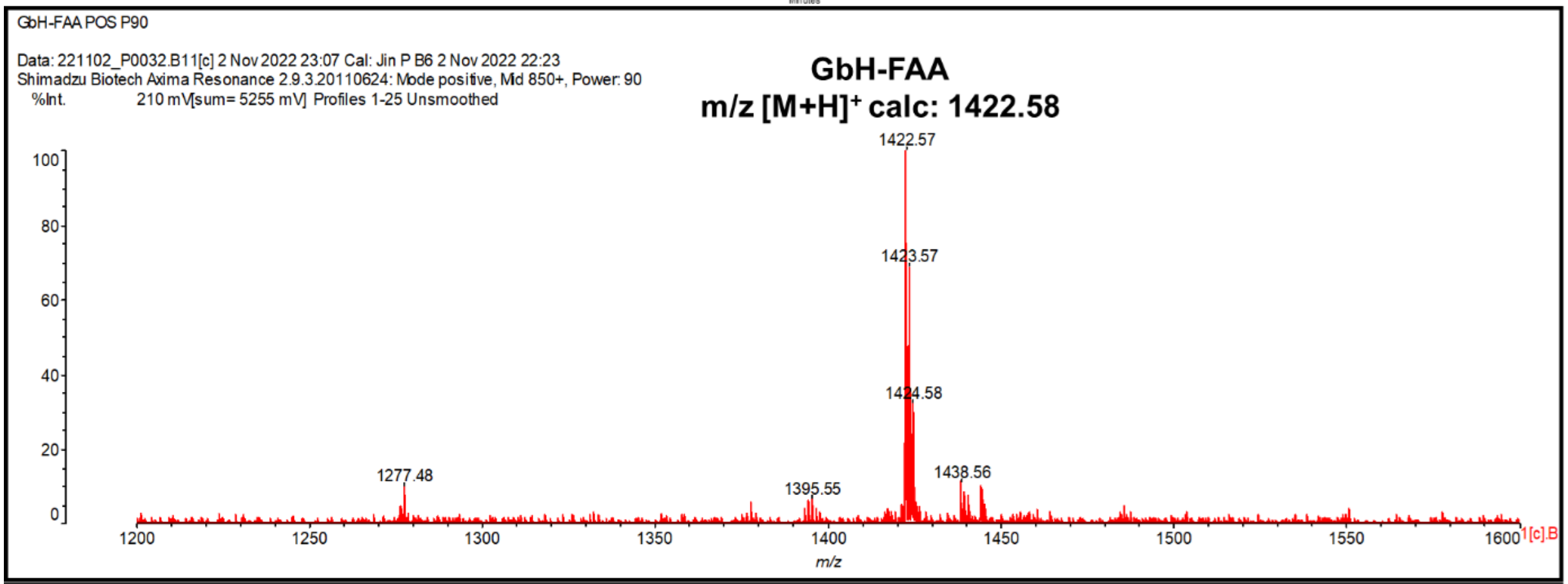

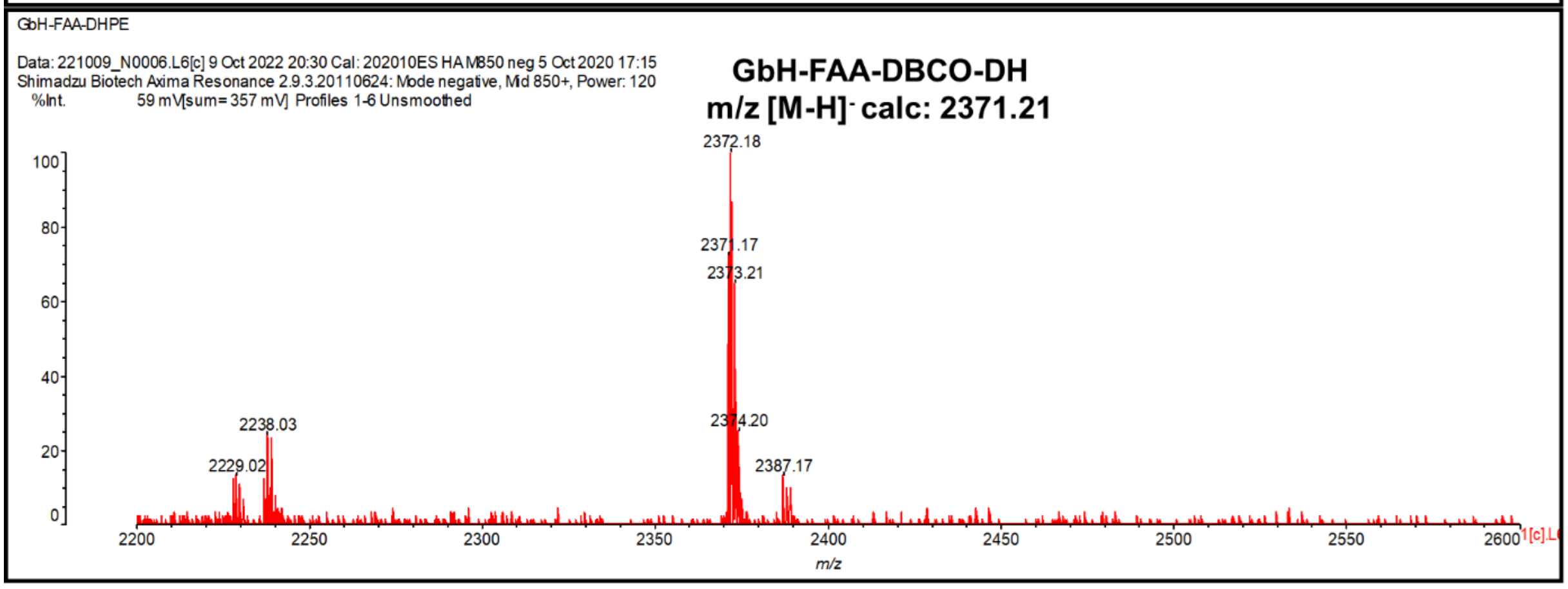

Probe No 19

GloboH analogue 2-FAA

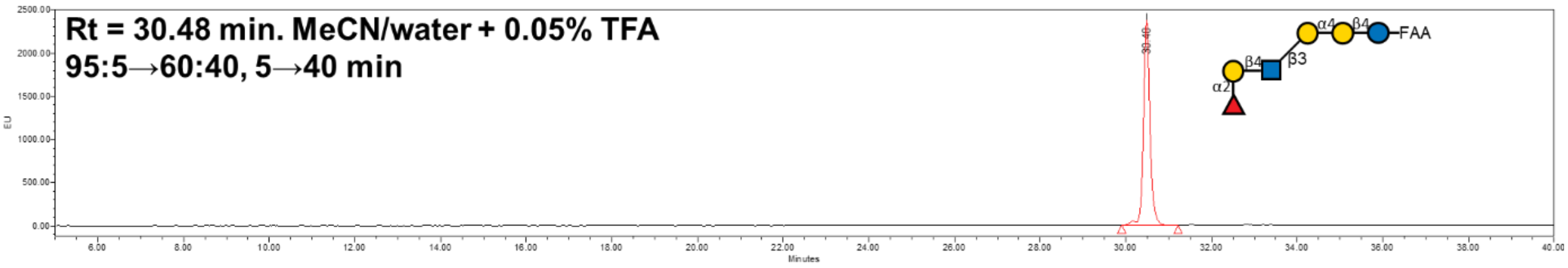

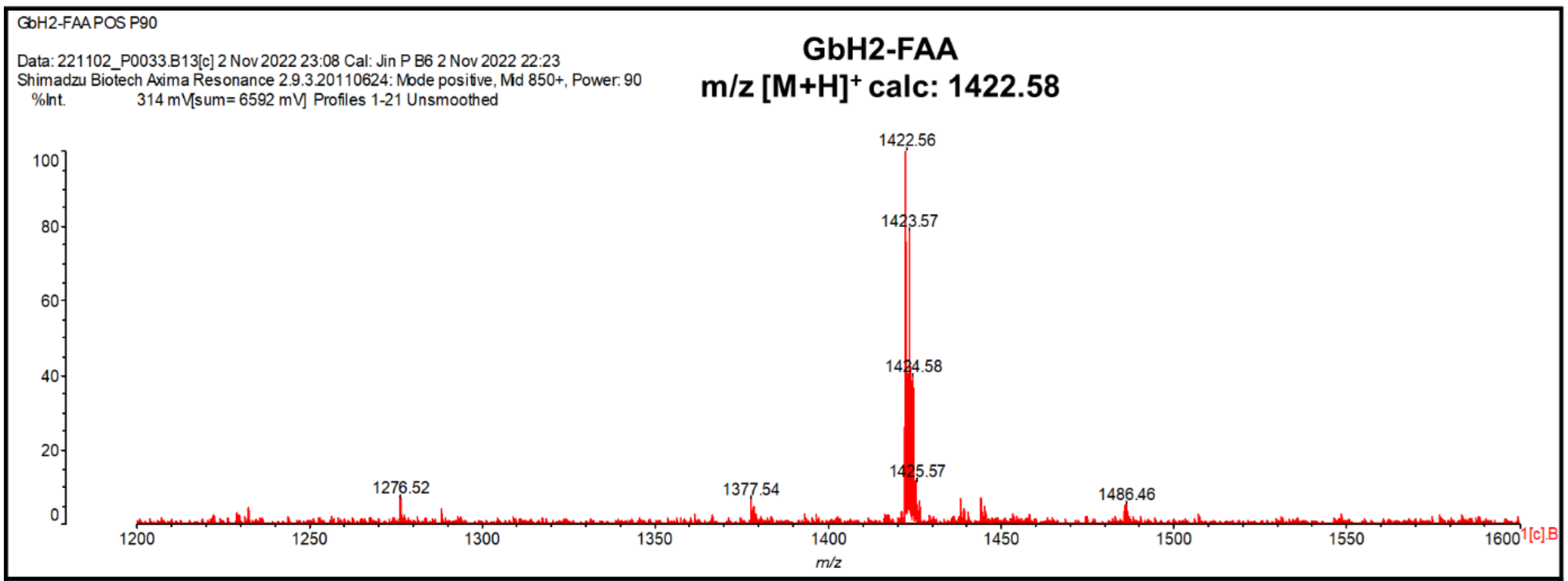

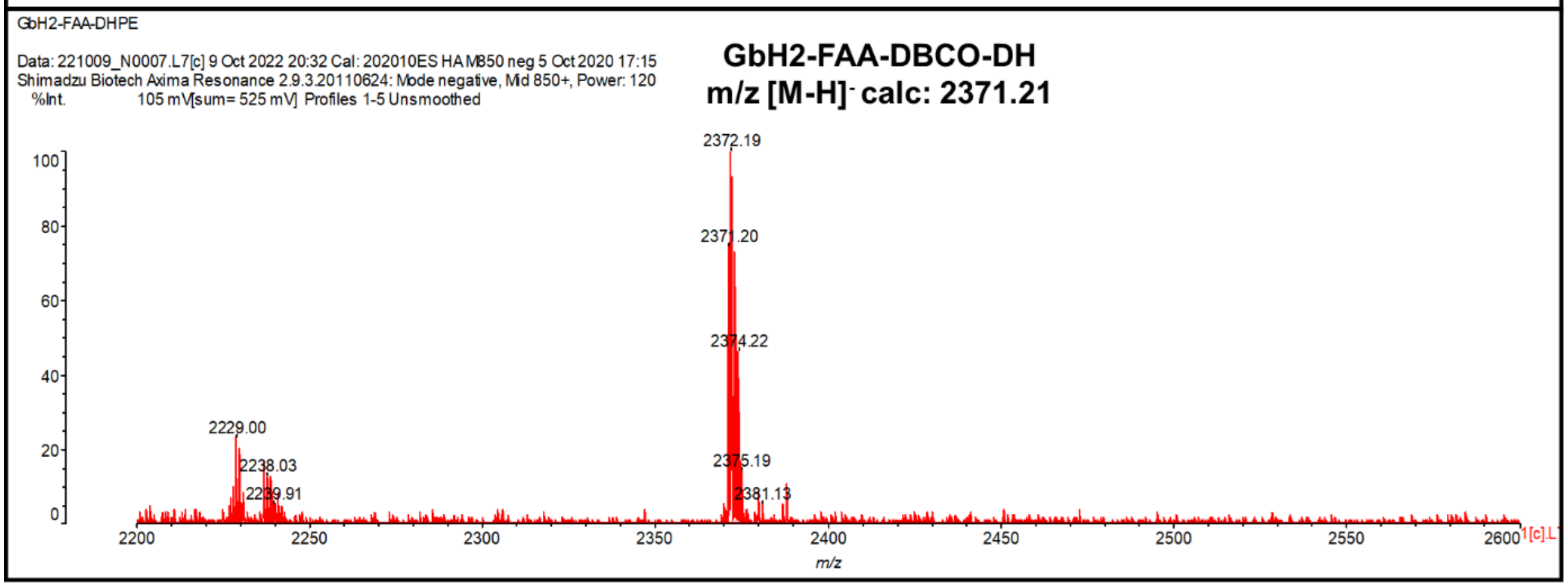

Probe No 20

GloboA-FAA

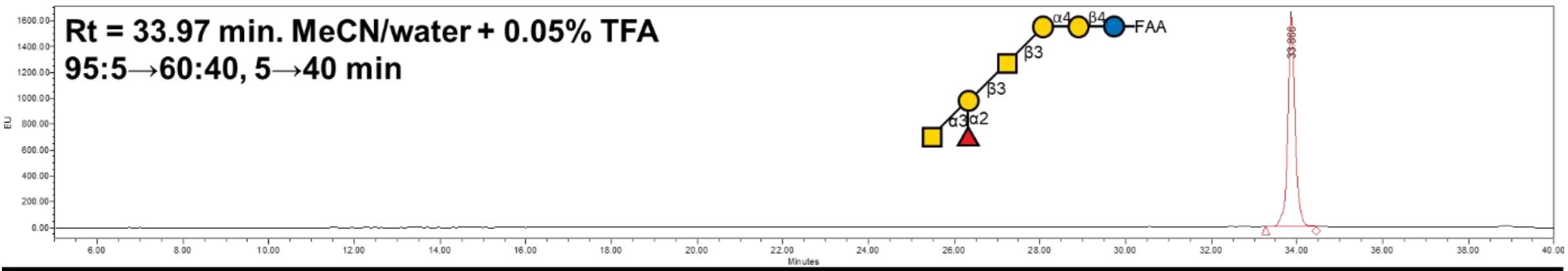

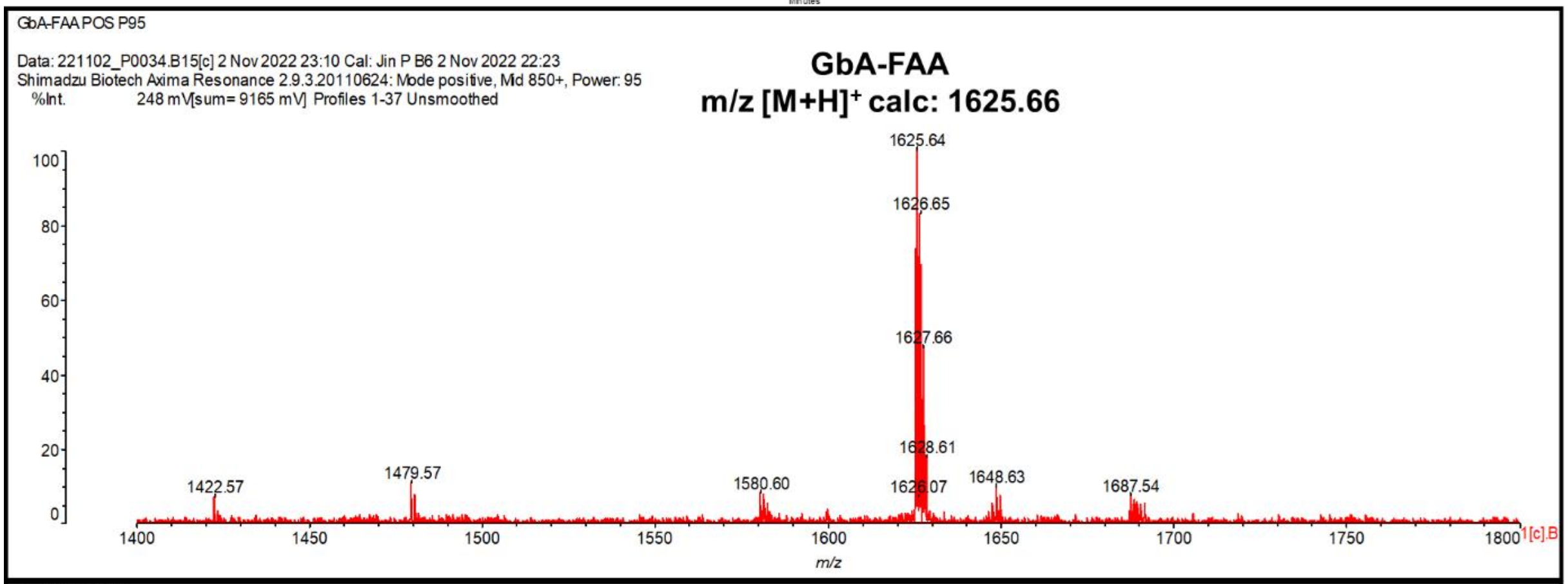

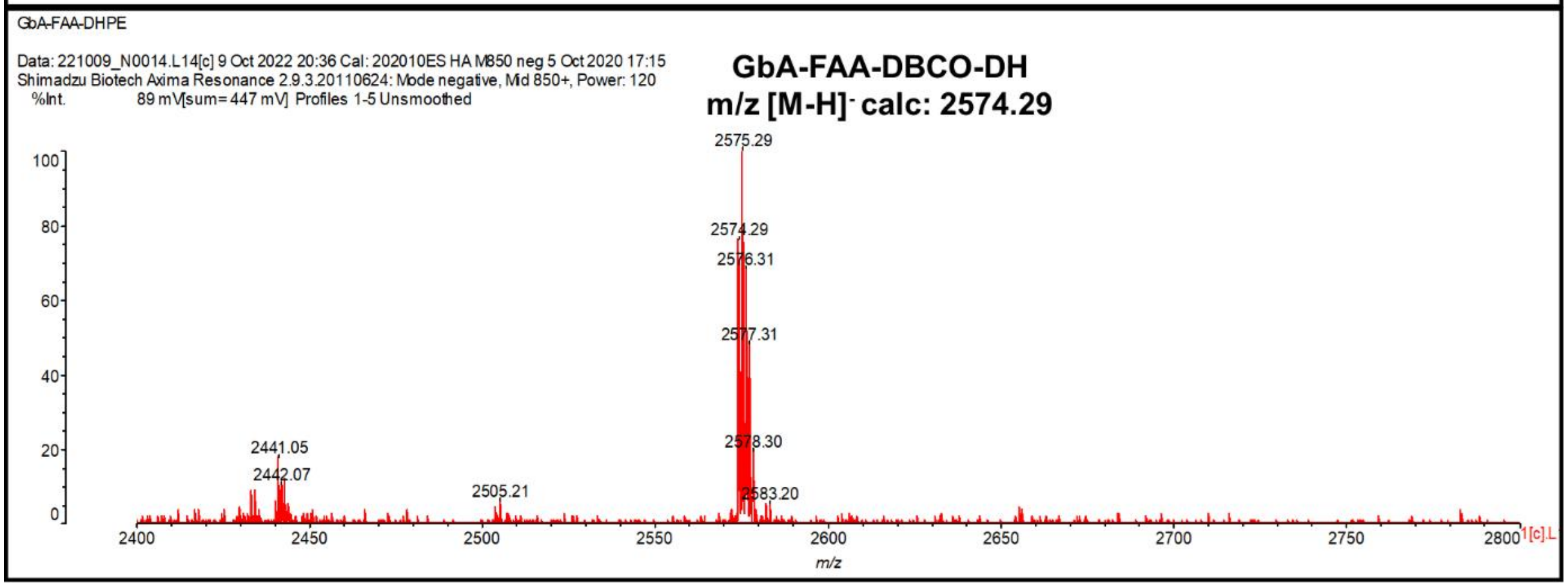

Probe No 21

GloboB-FAA

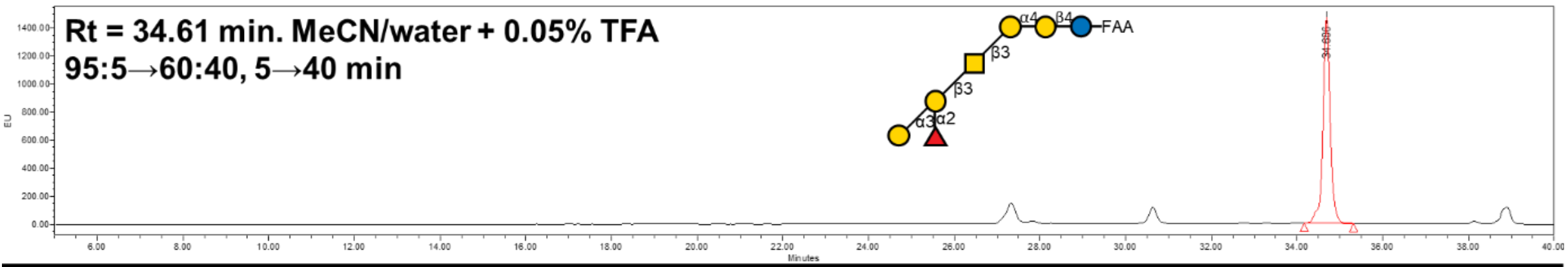

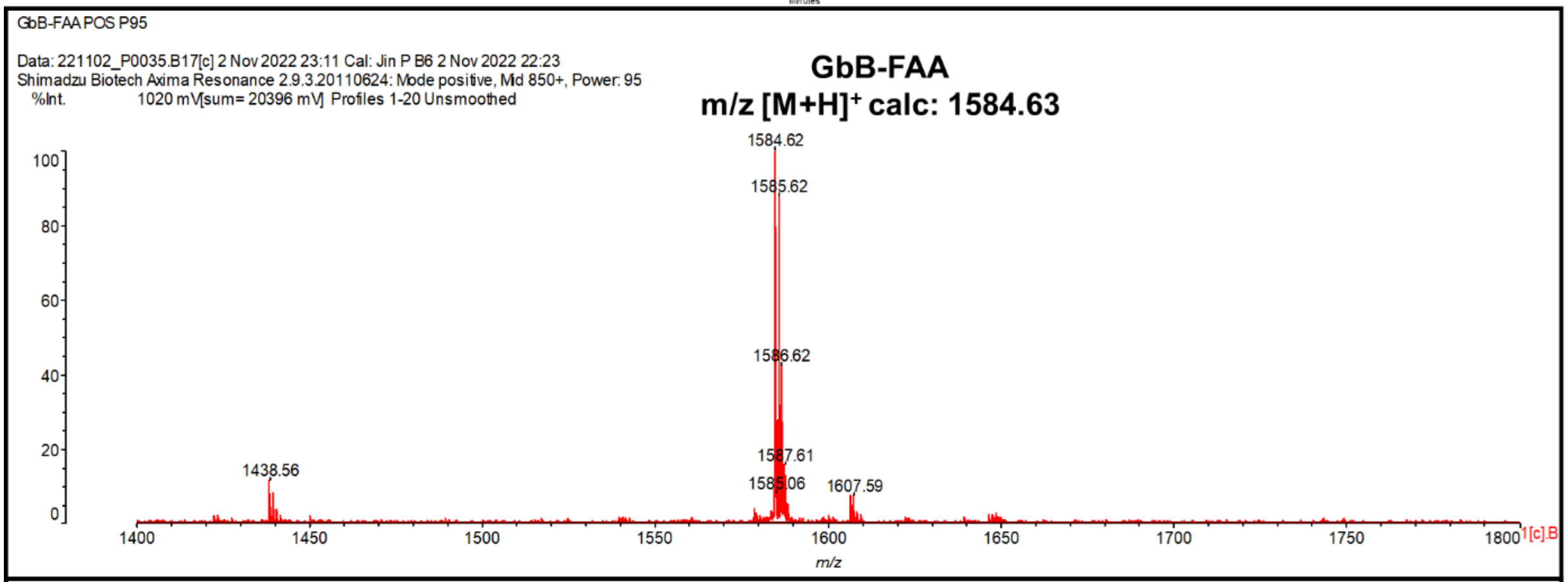

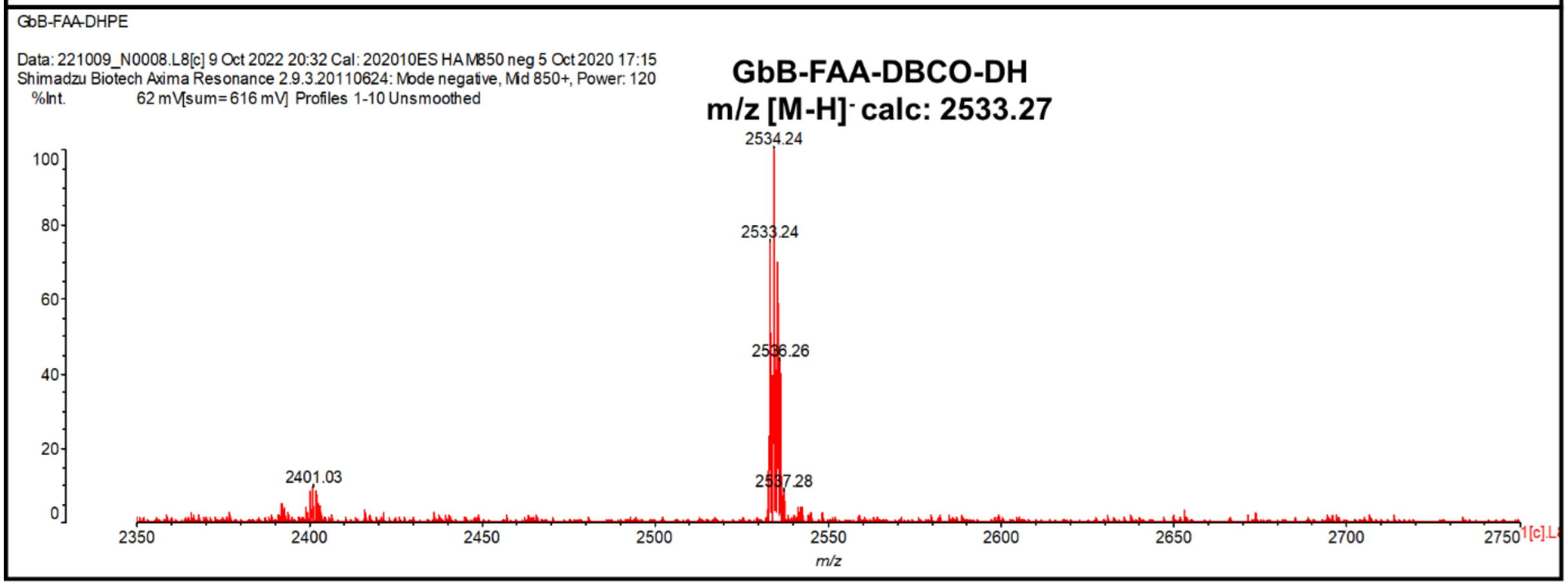

Probe No 22

3SL-FAA

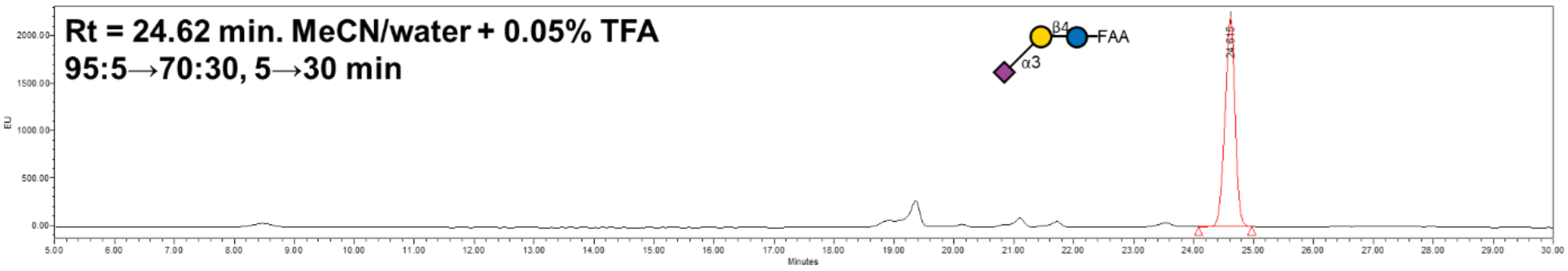

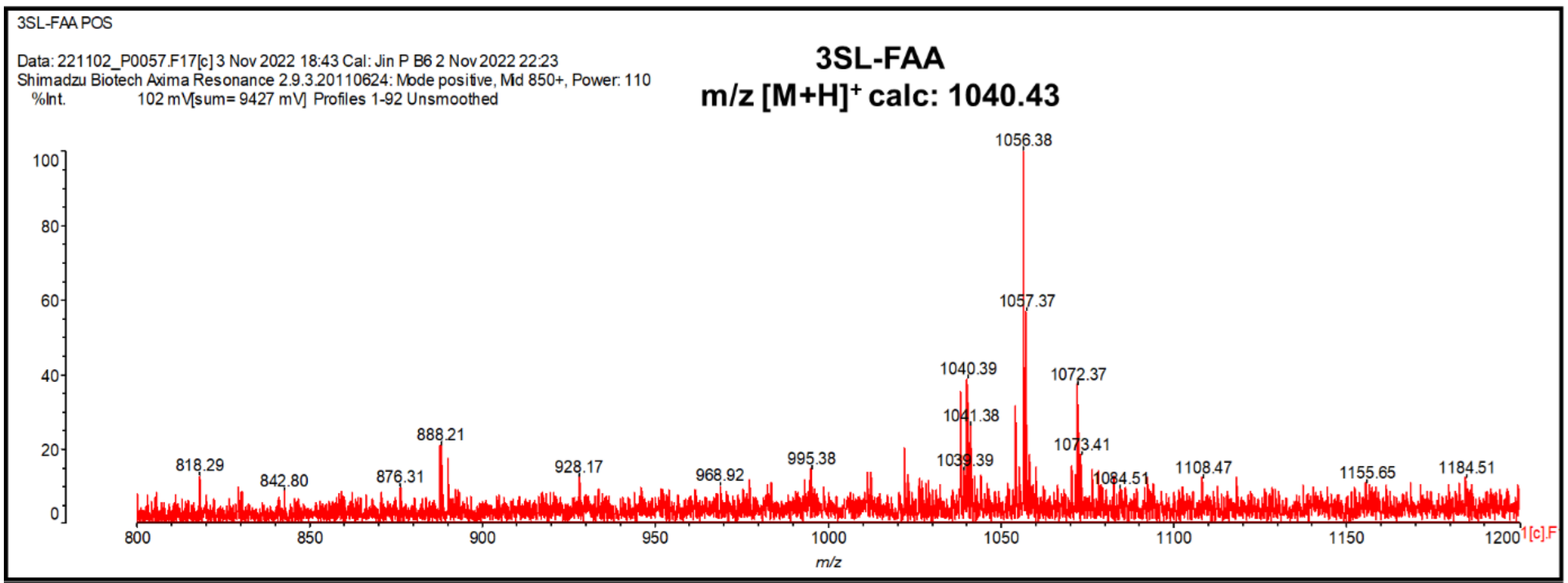

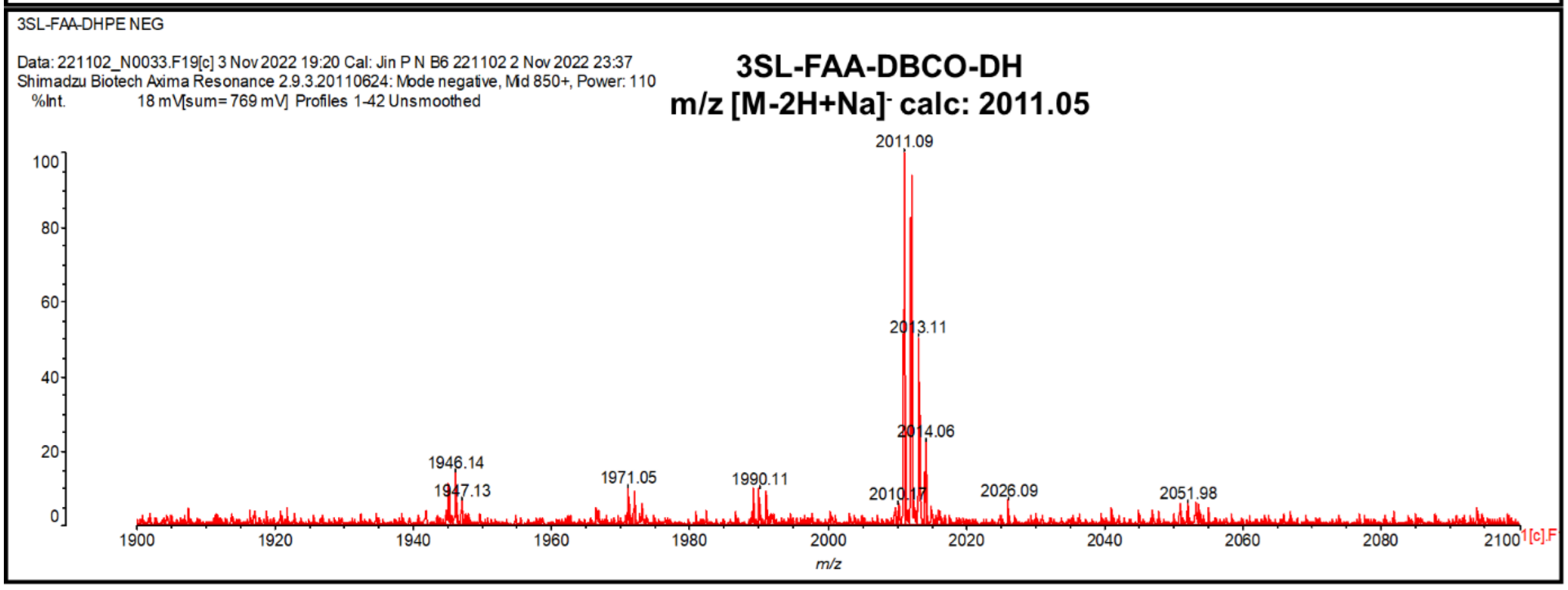

Probe No 23

LSTa-FAA

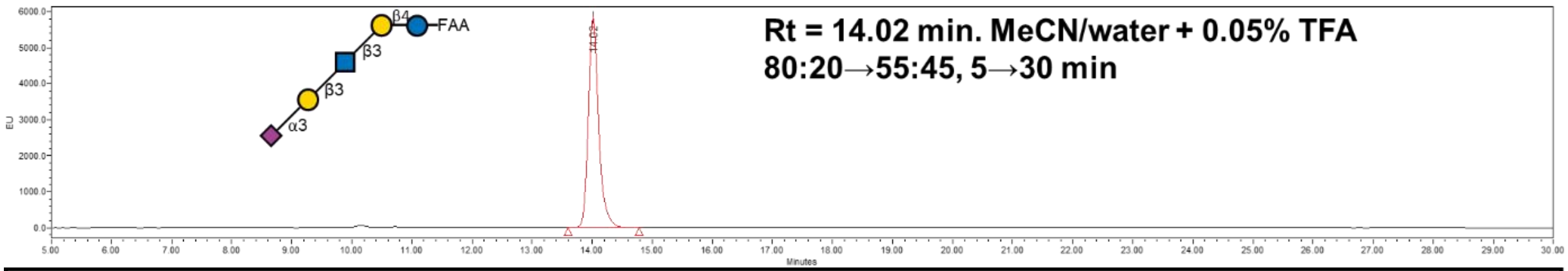

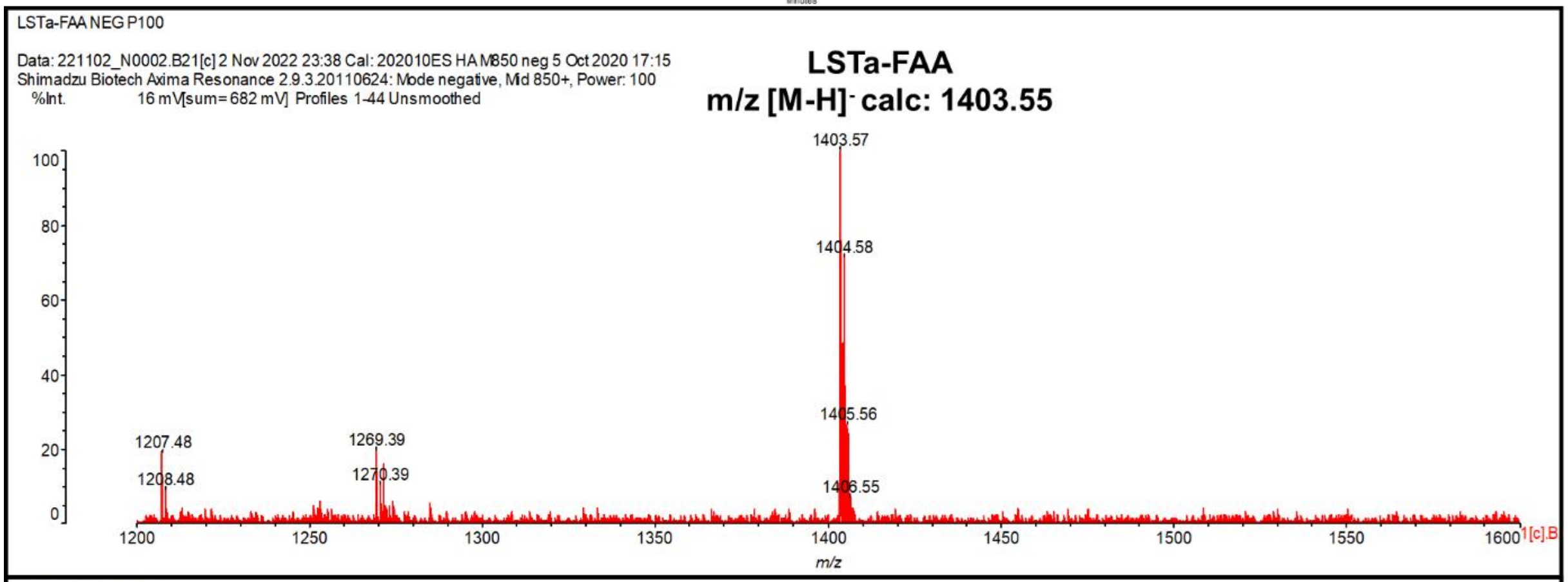

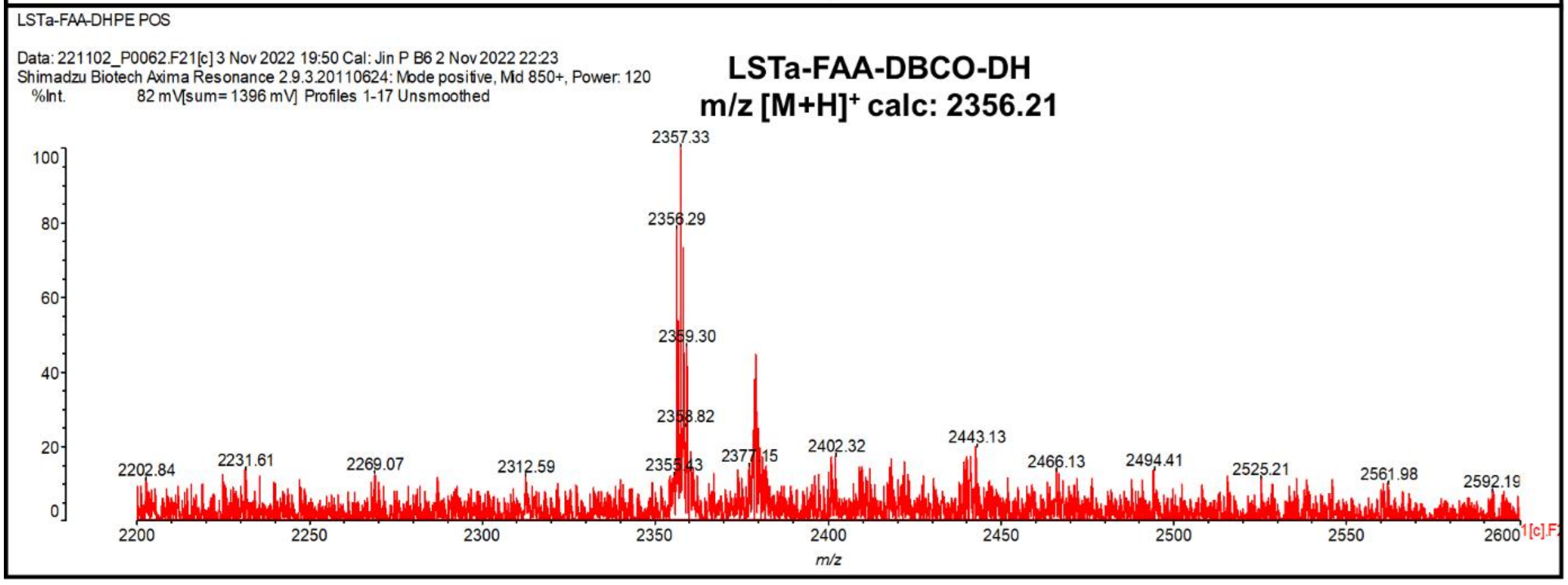

Probe No 24

LSTd-FAA

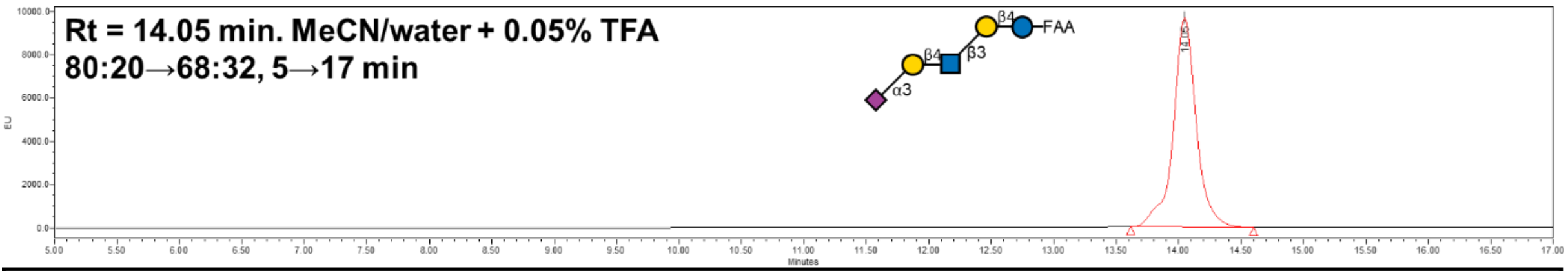

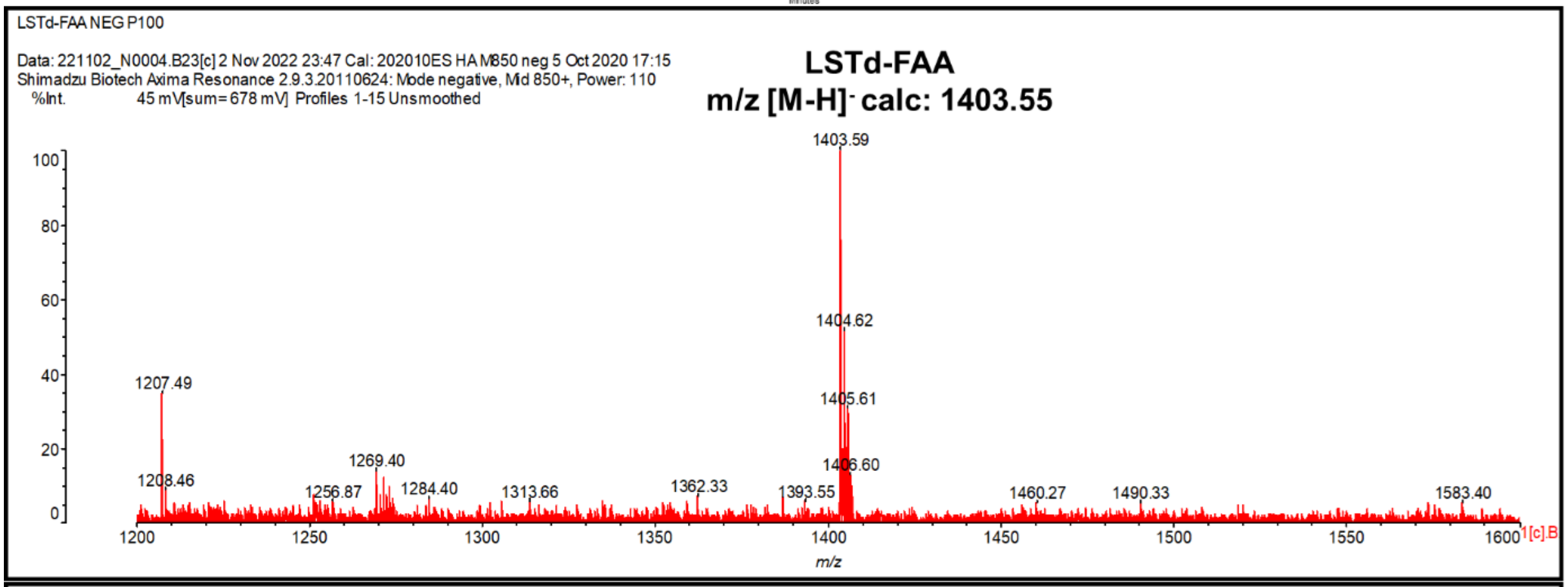

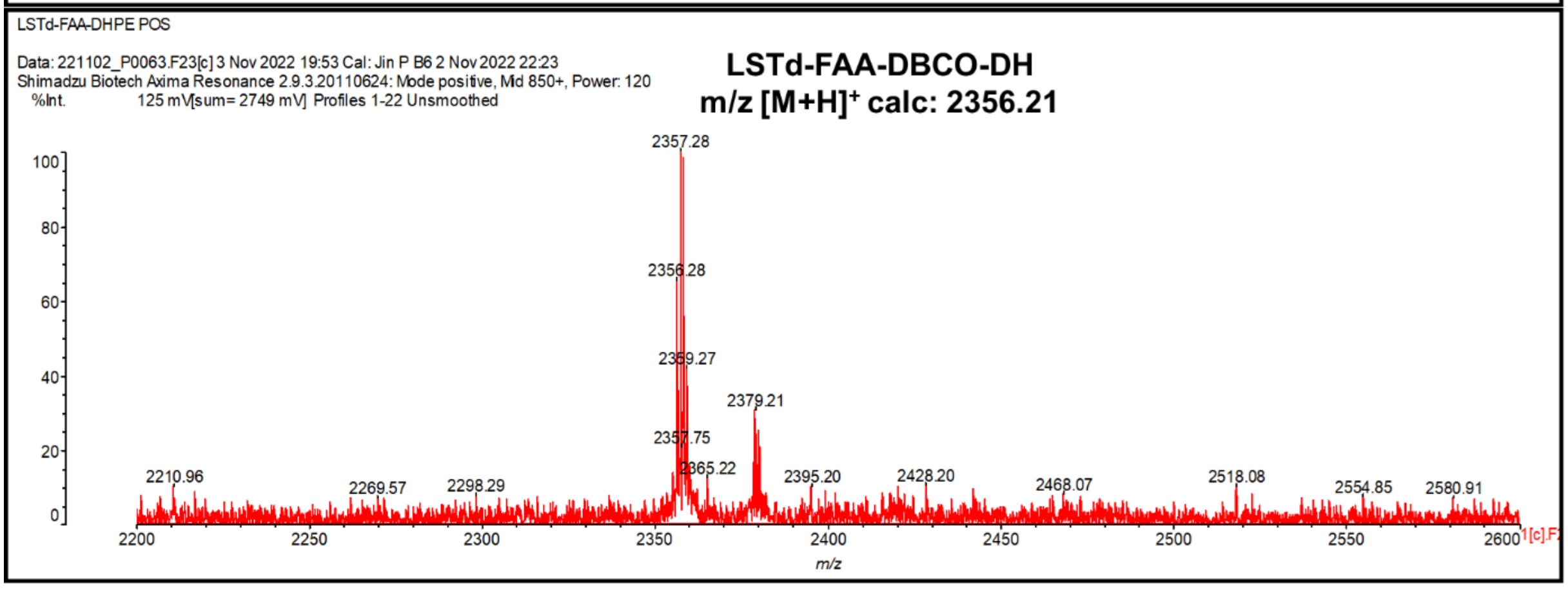

Probe No 25

LSTc-FAA

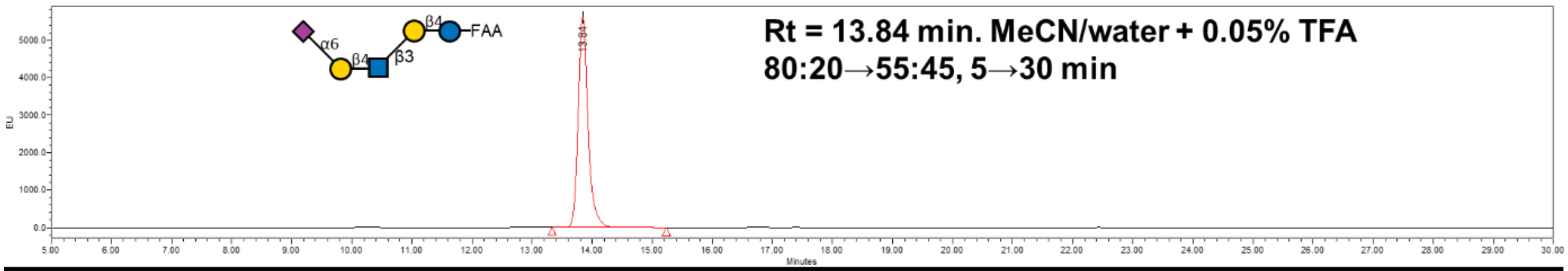

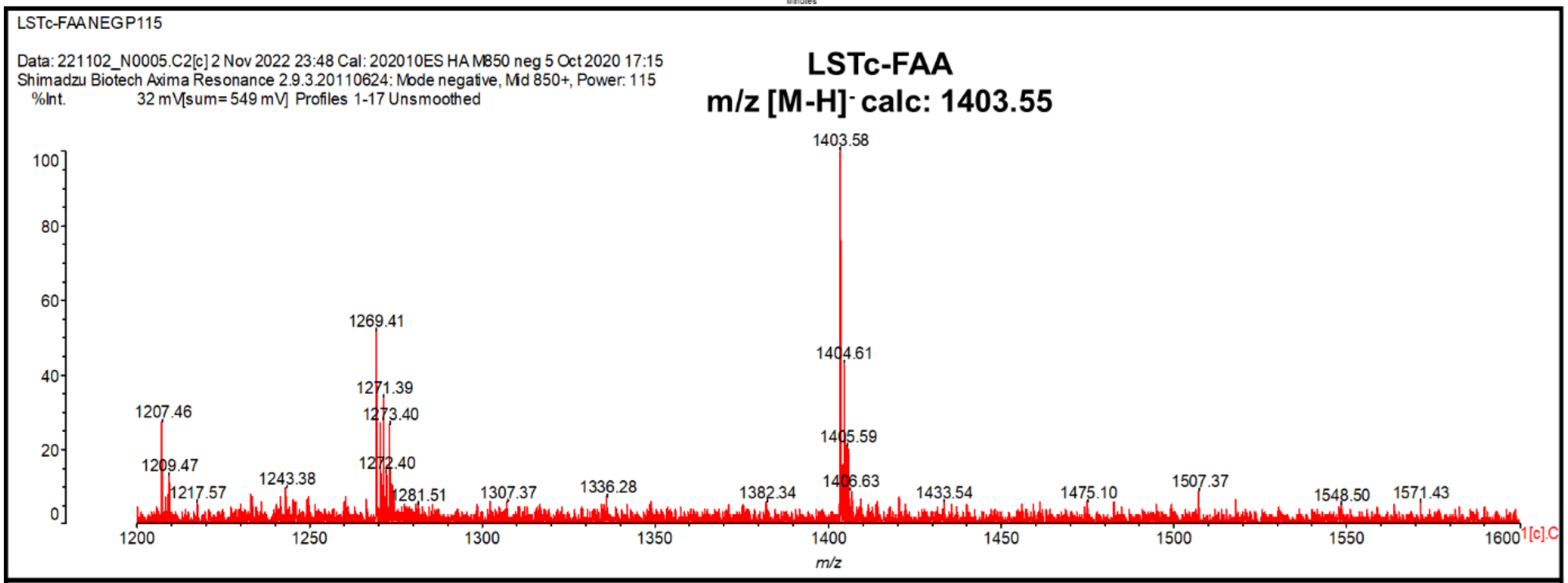

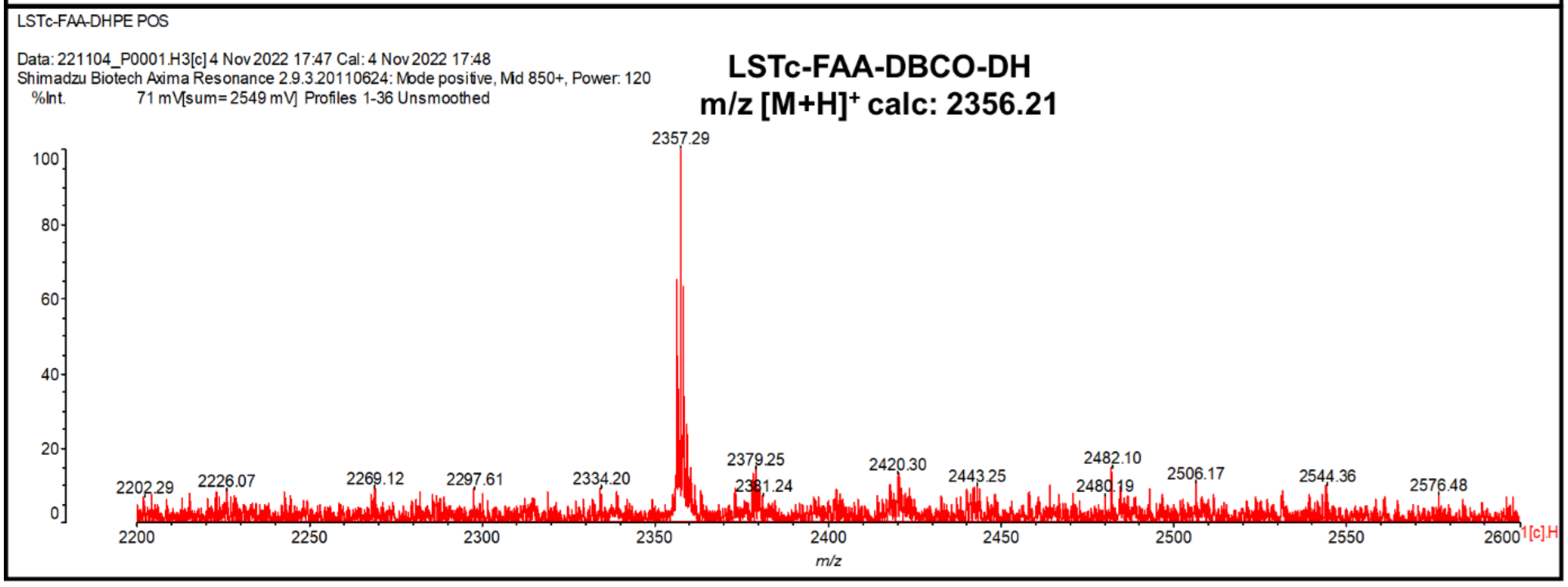

Probe No 26

LSTb-FAA

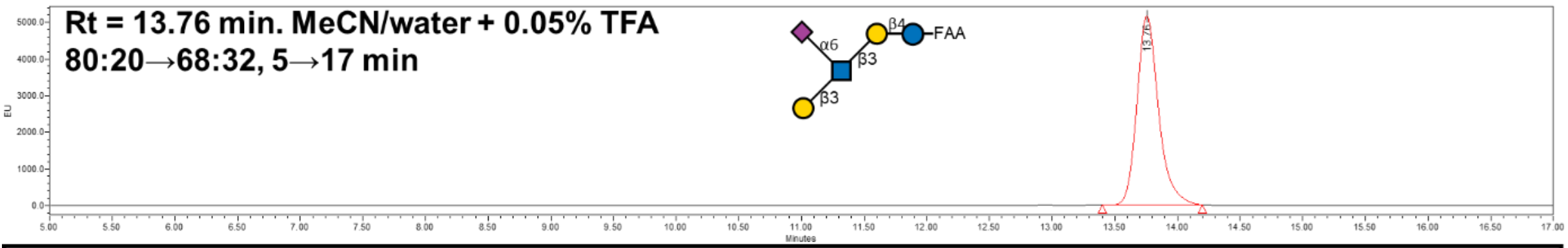

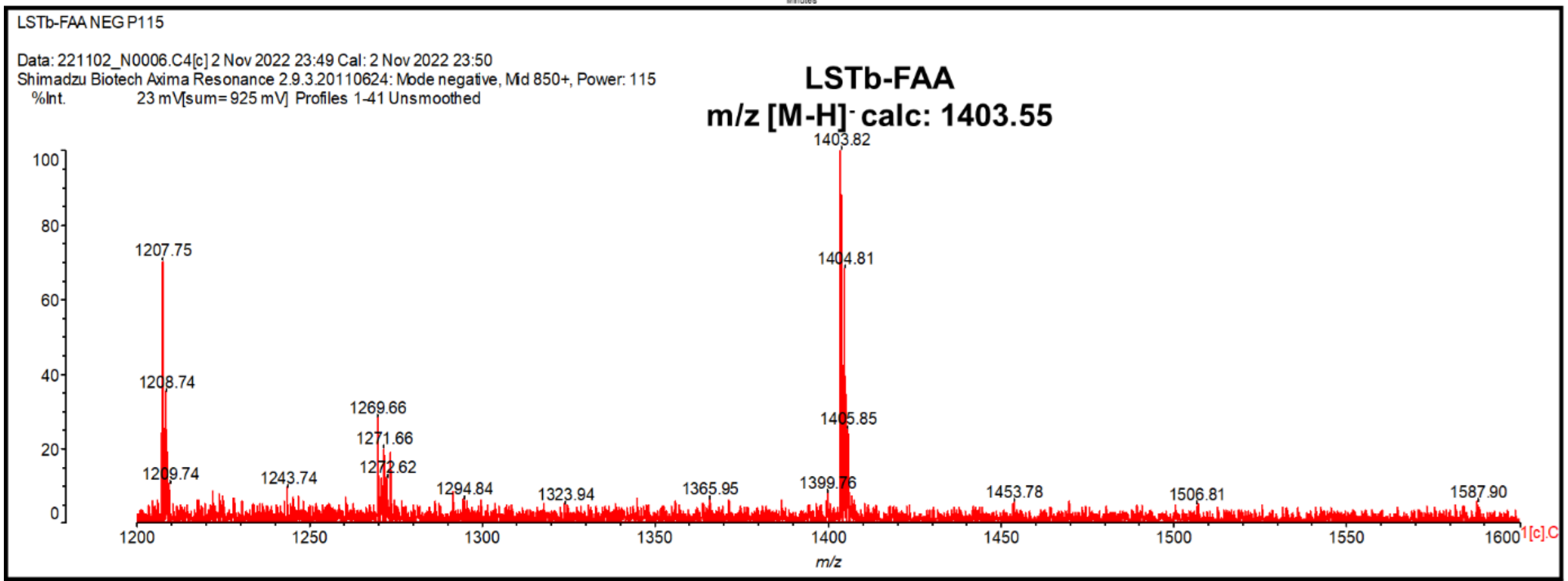

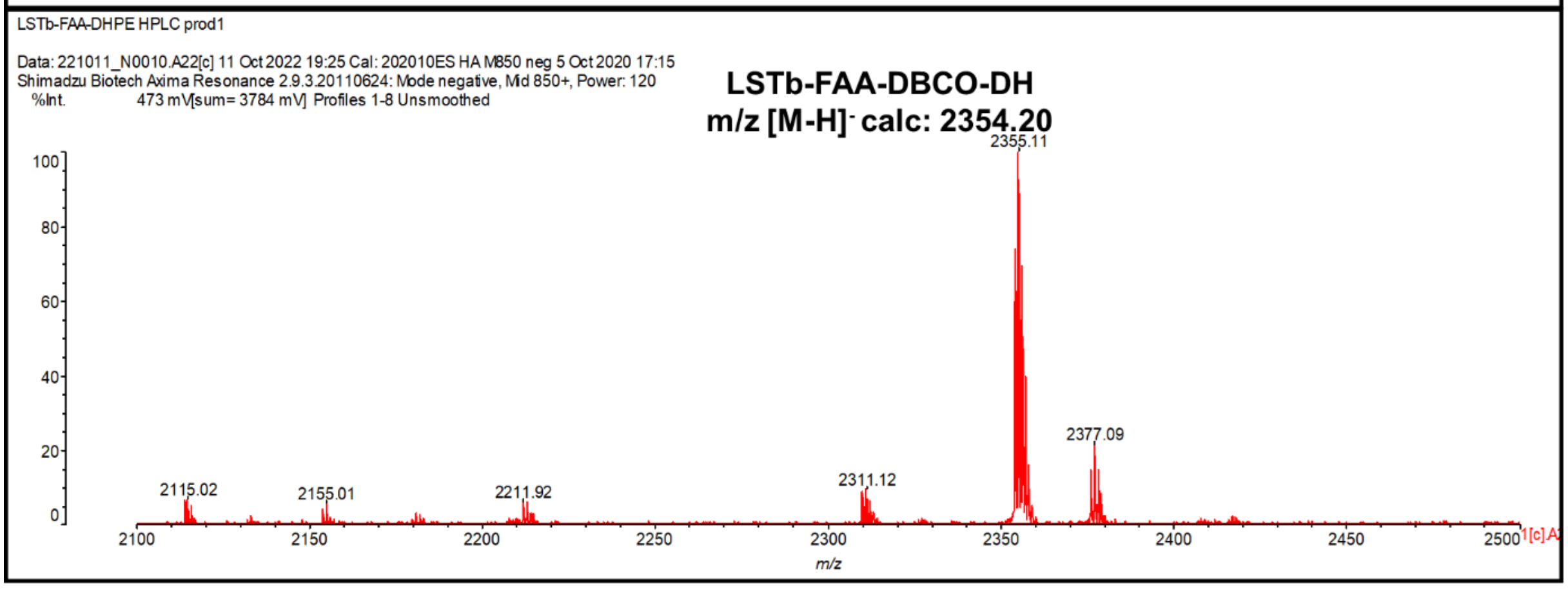

Probe No 27

SSEA4-FAA

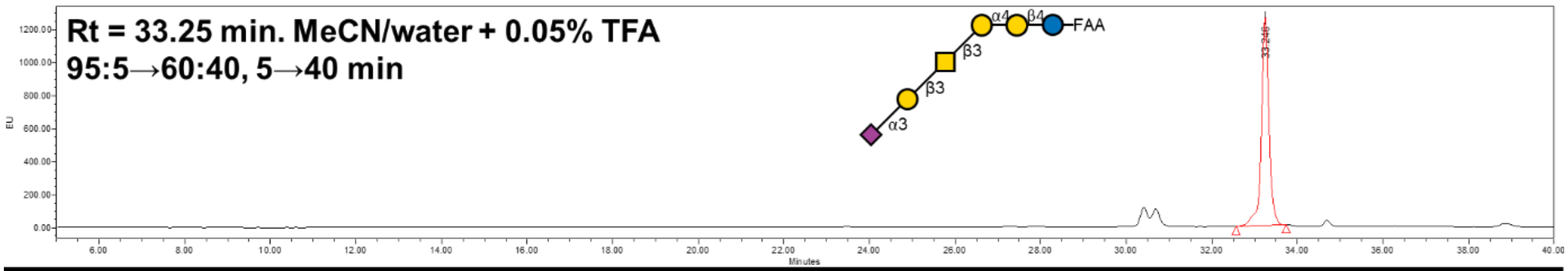

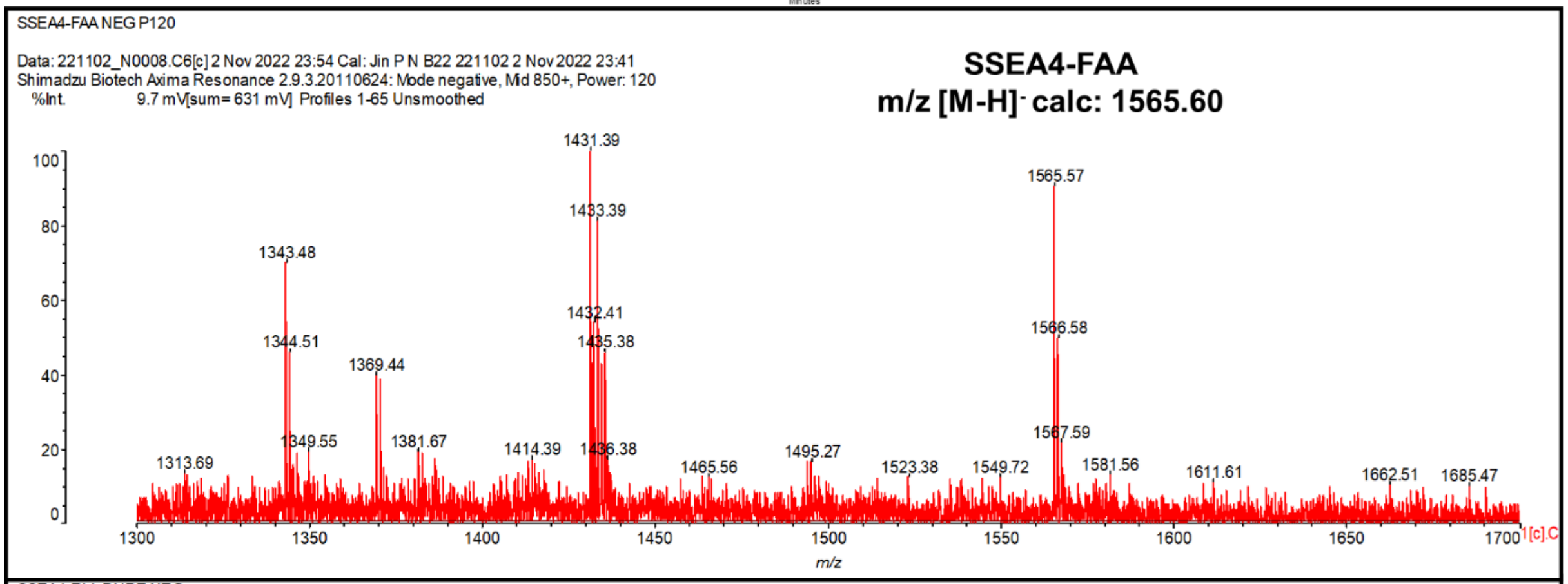

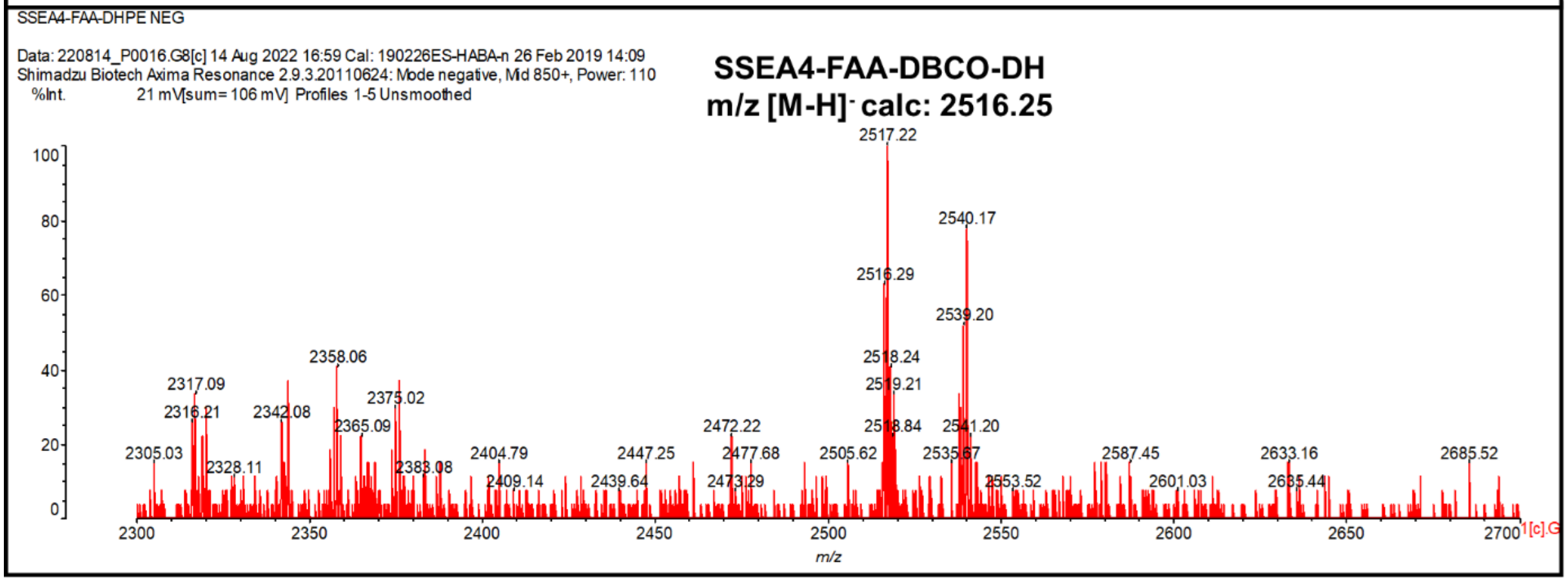

Probe No 28

SLex penta-FAA

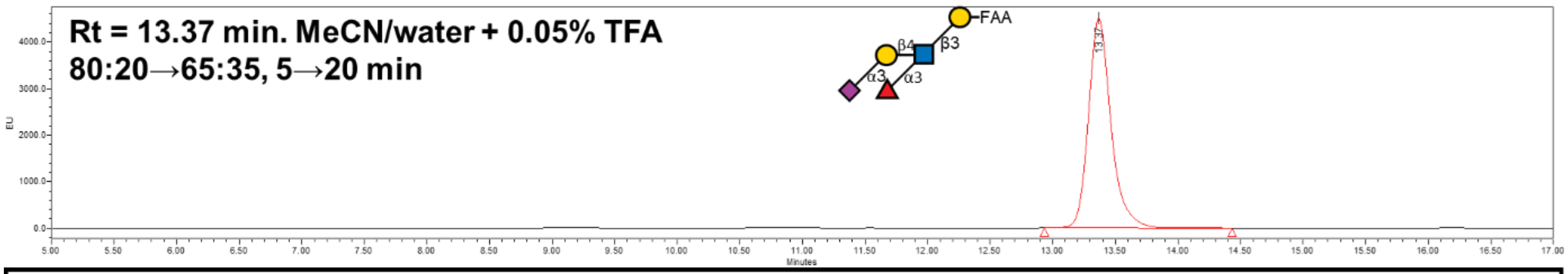

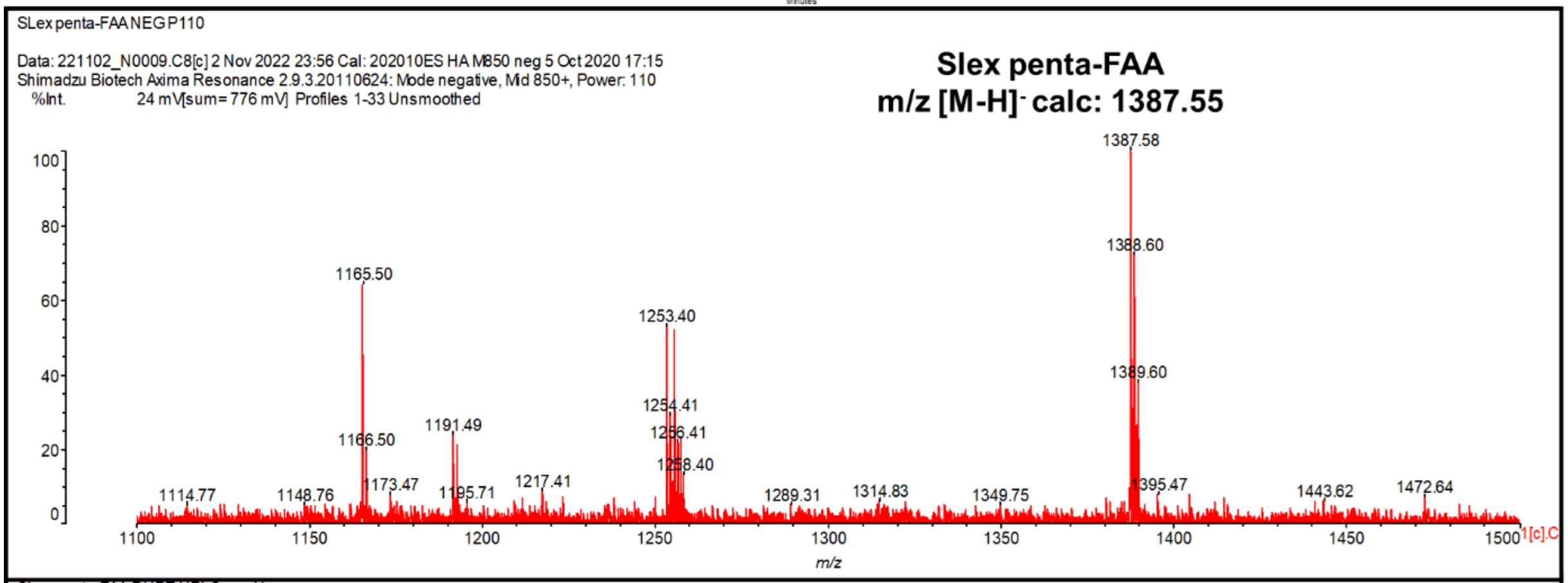

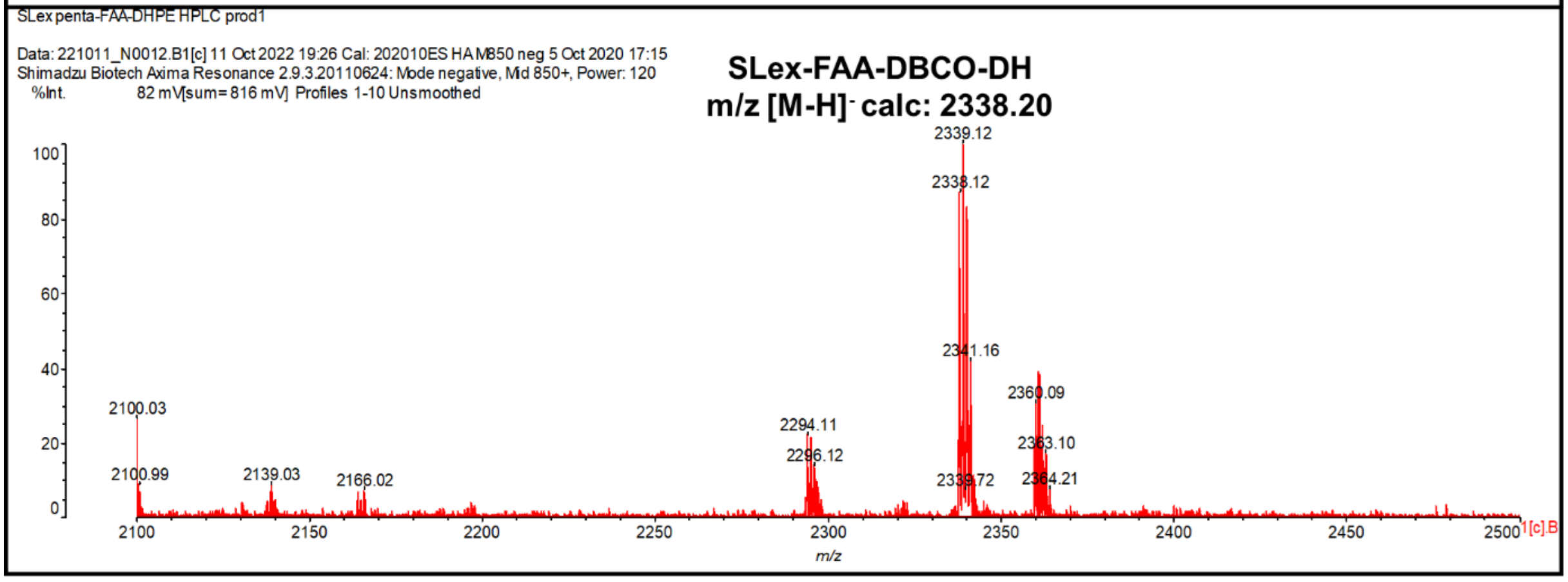

Probe No 29

GM1a-FAA

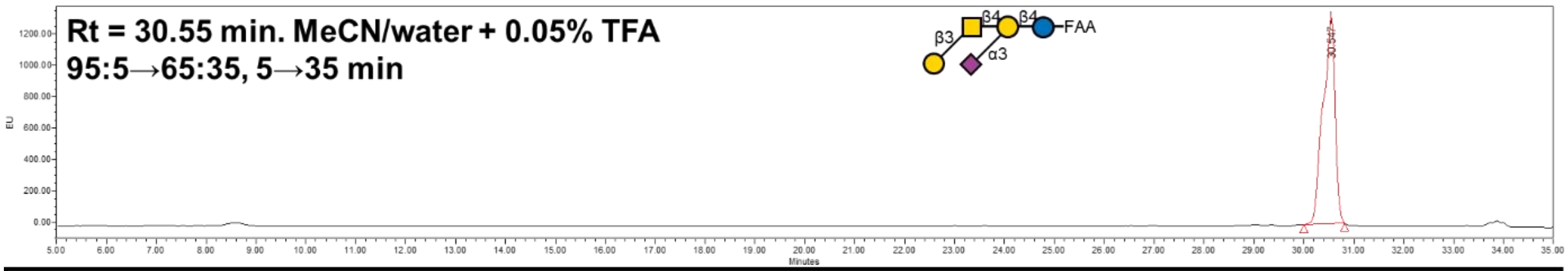

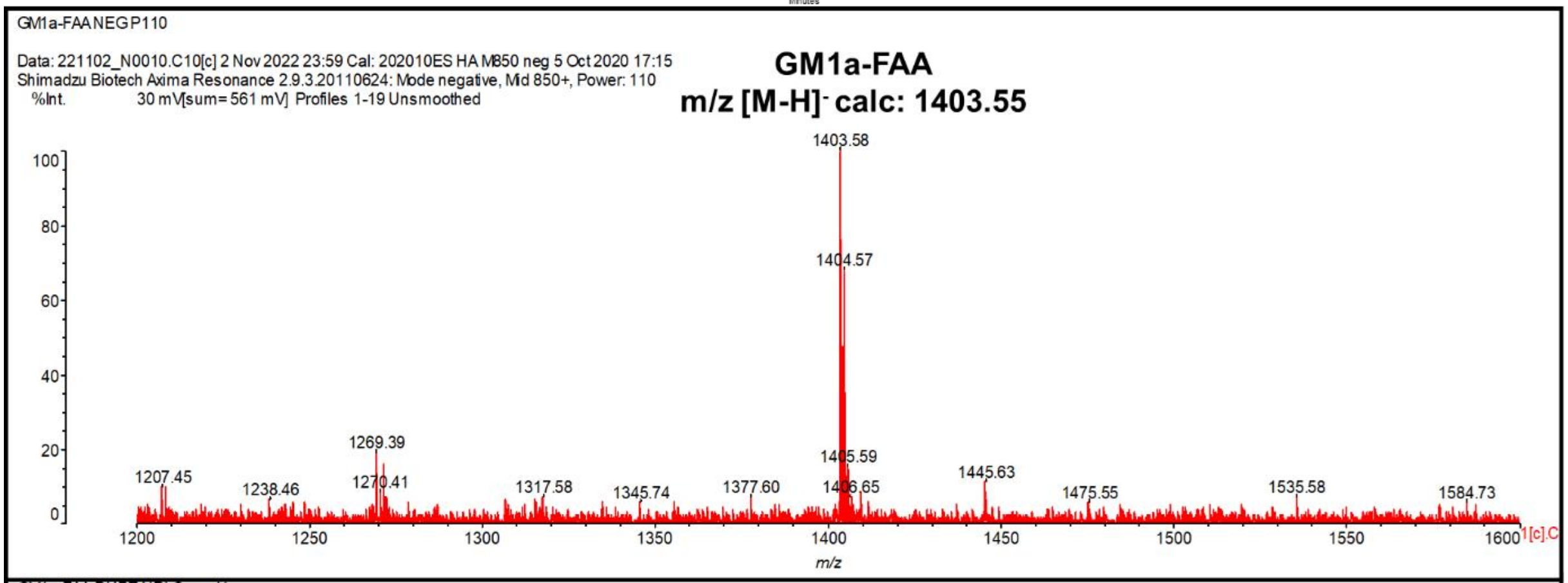

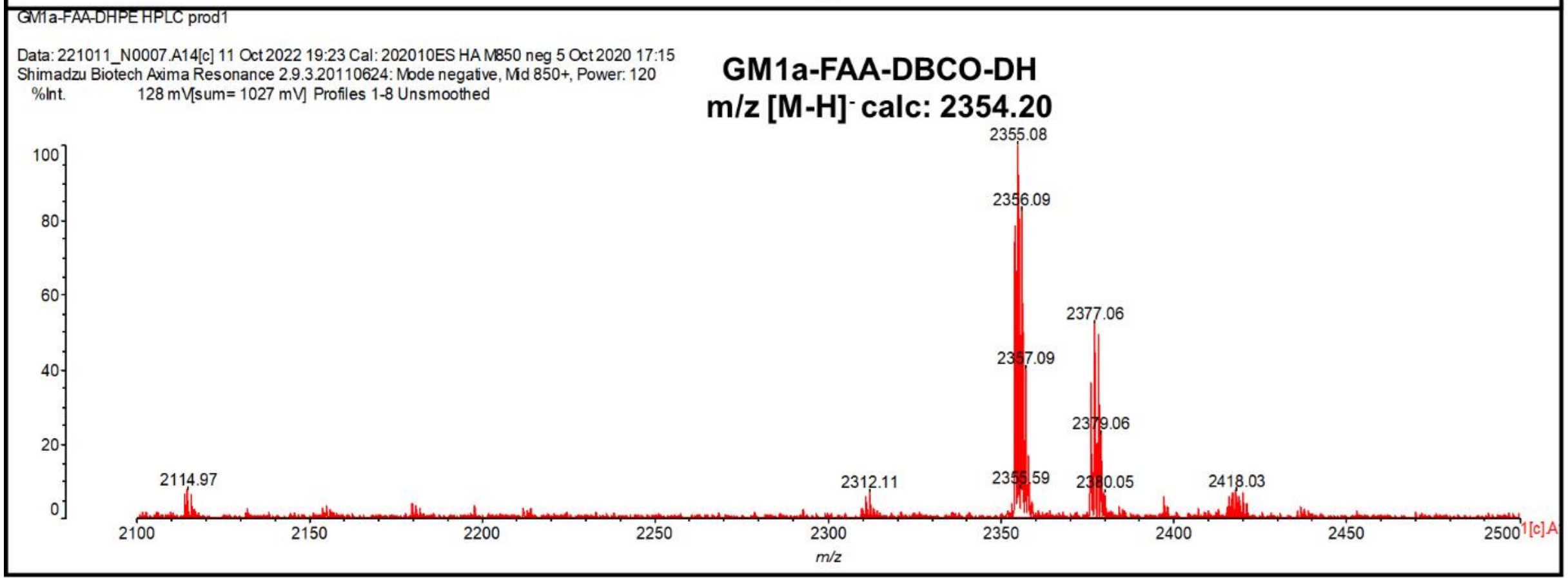

Probe No 30

GM1b-FAA

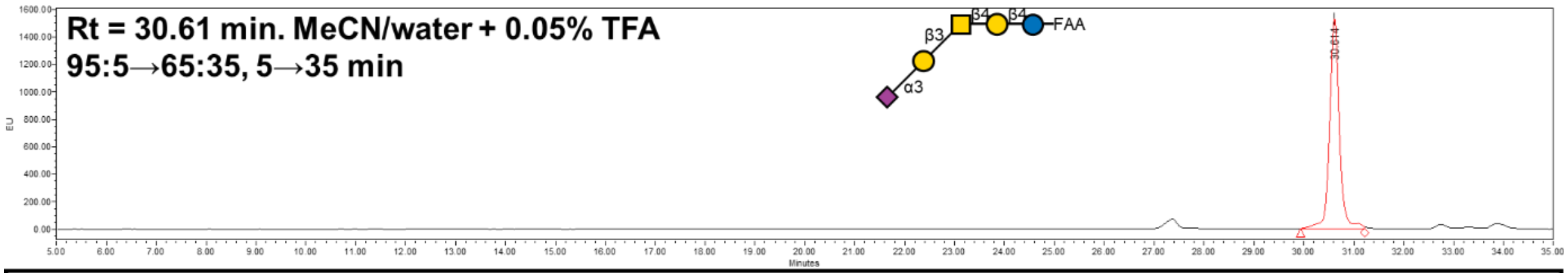

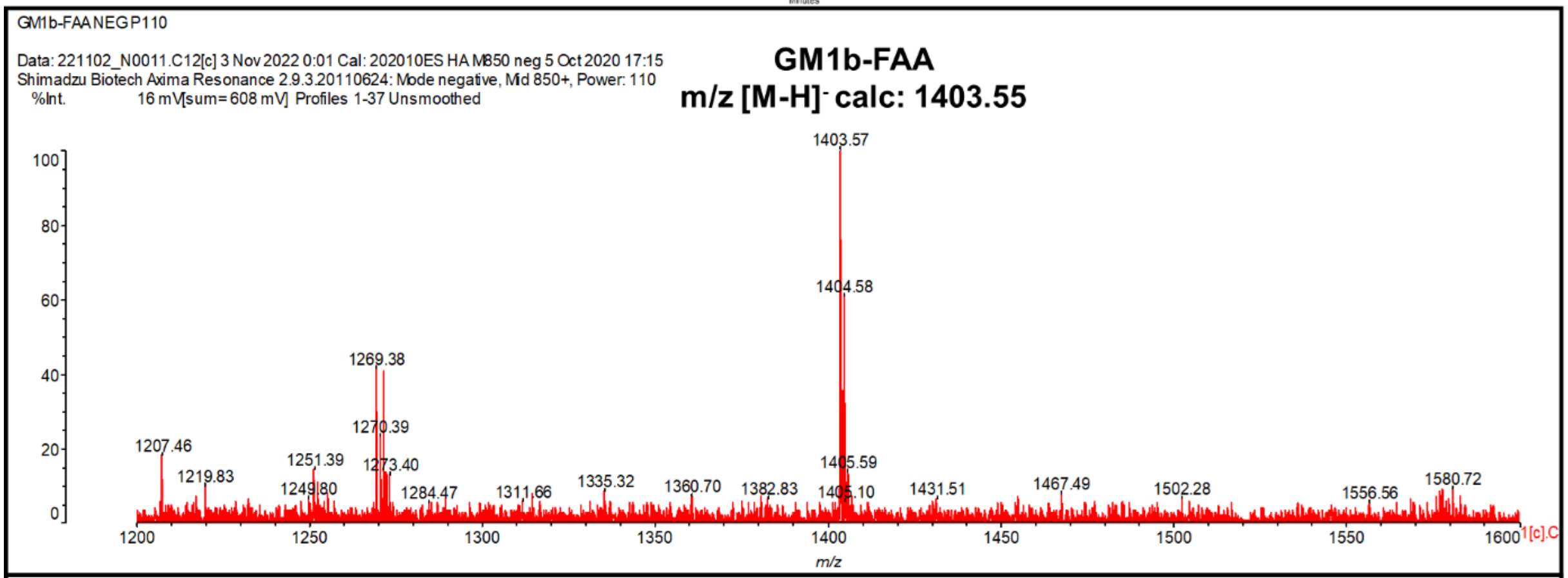

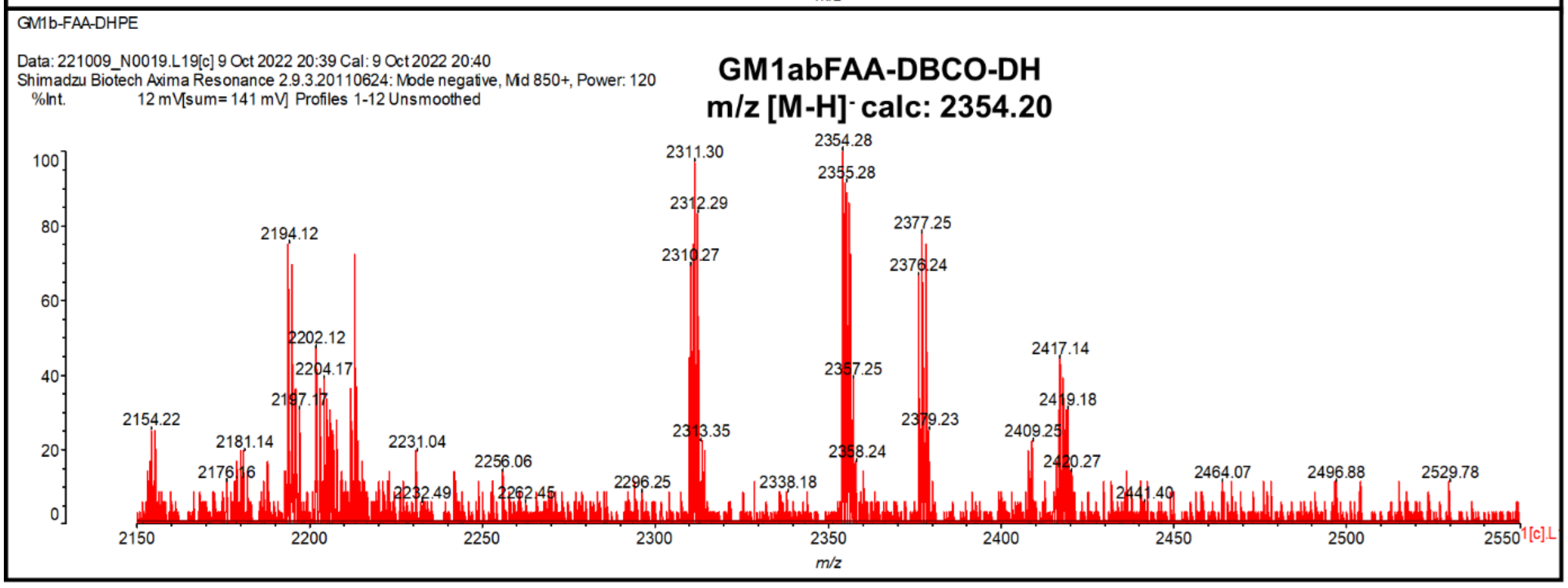

Probe No 31

GD1a-FAA

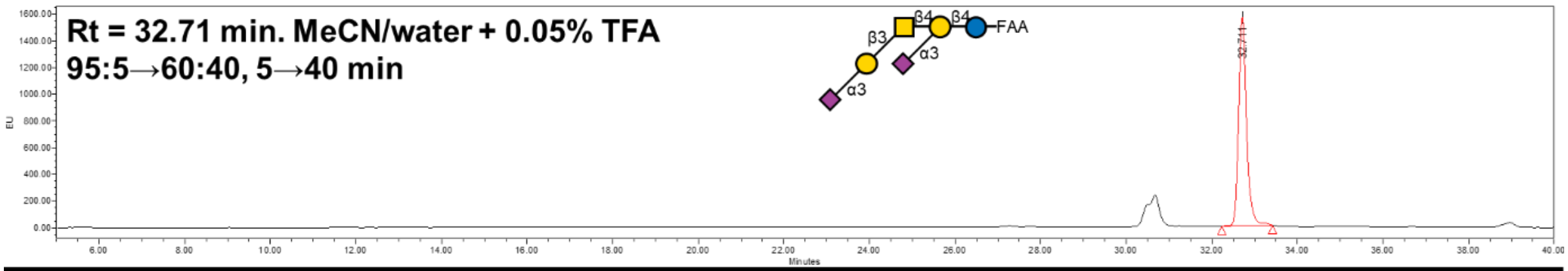

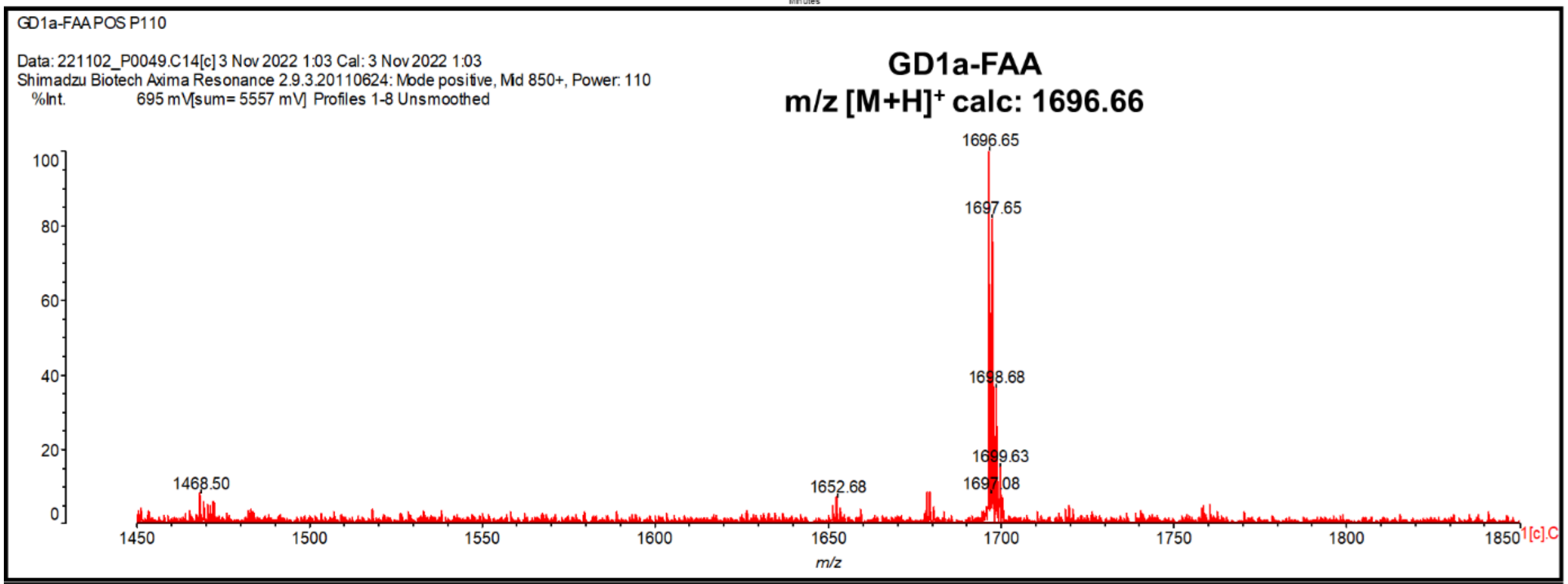

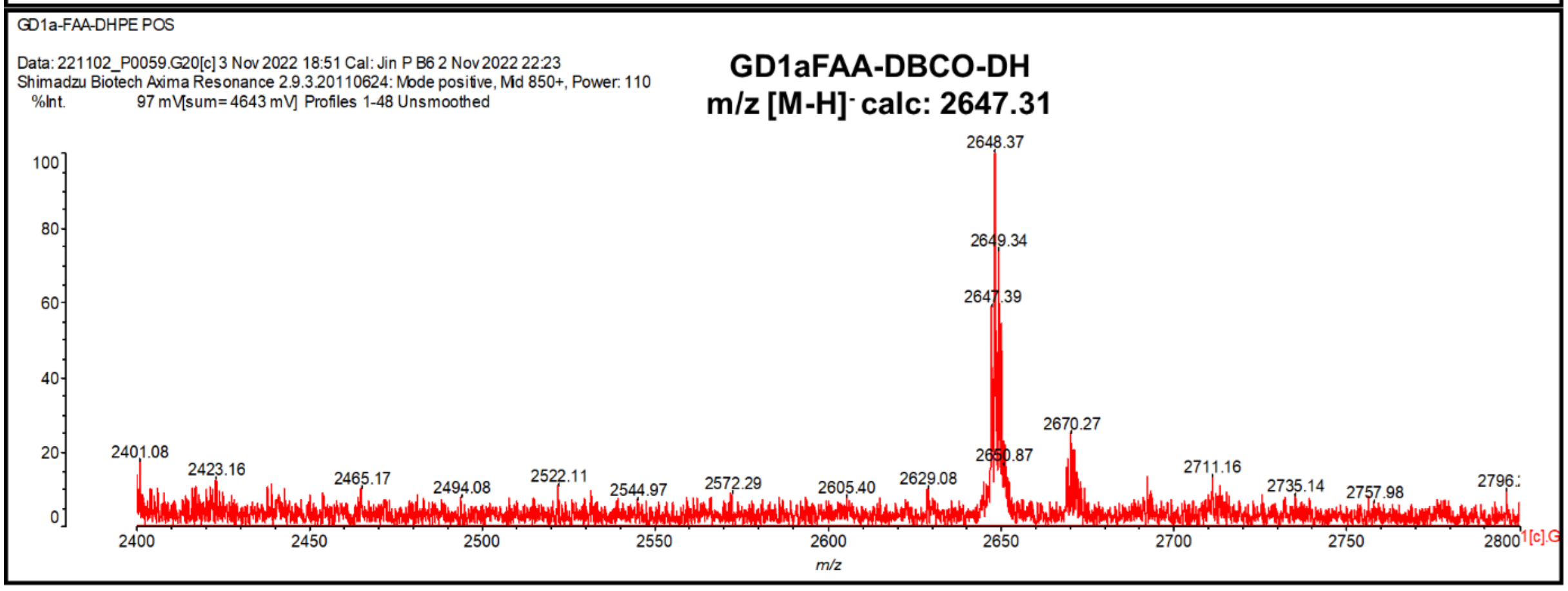

Probe No 32

GalGalNAc asialo GM1-FAA

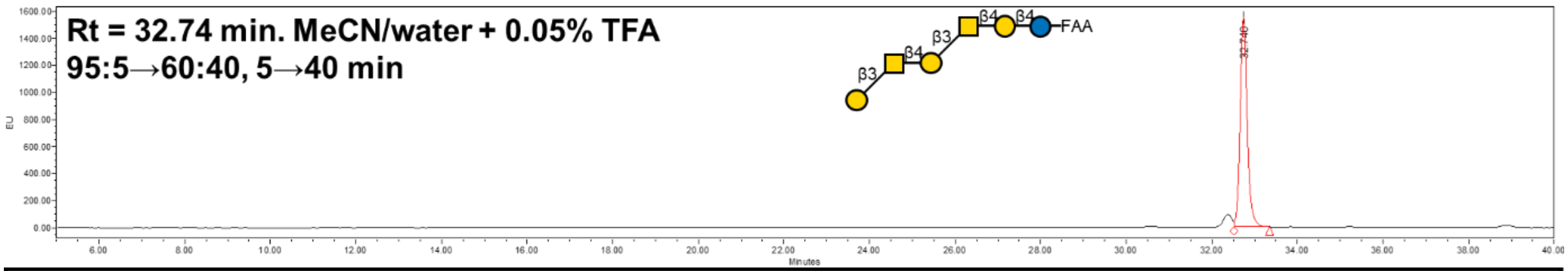

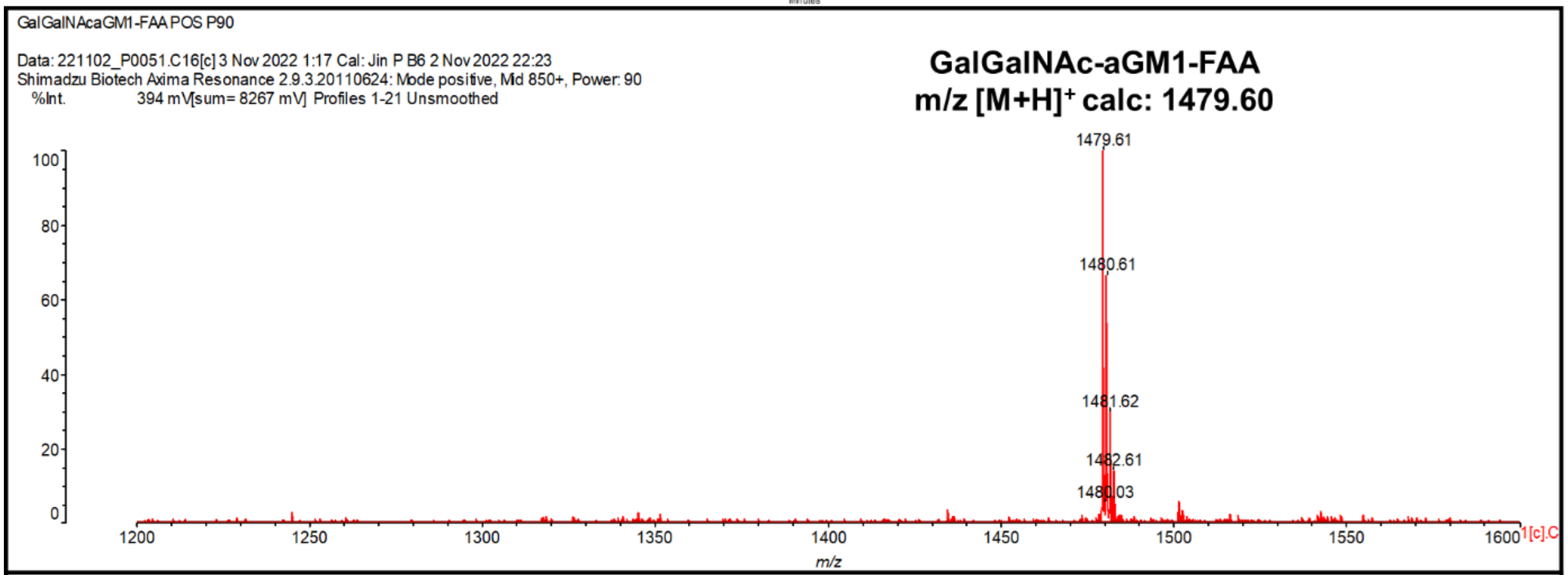

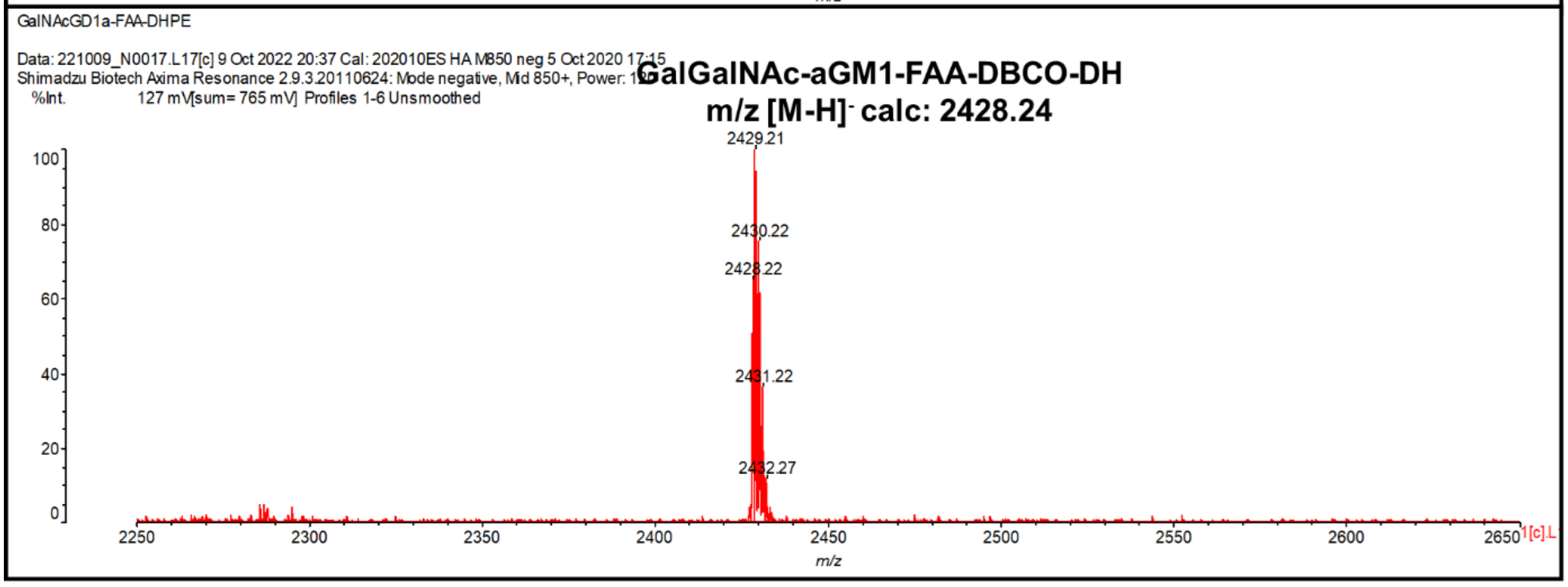

Probe No 33

GT1a-FAA

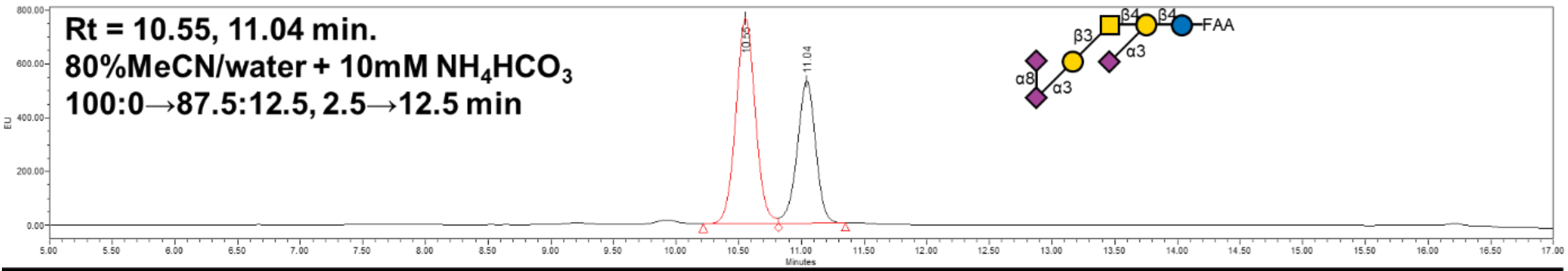

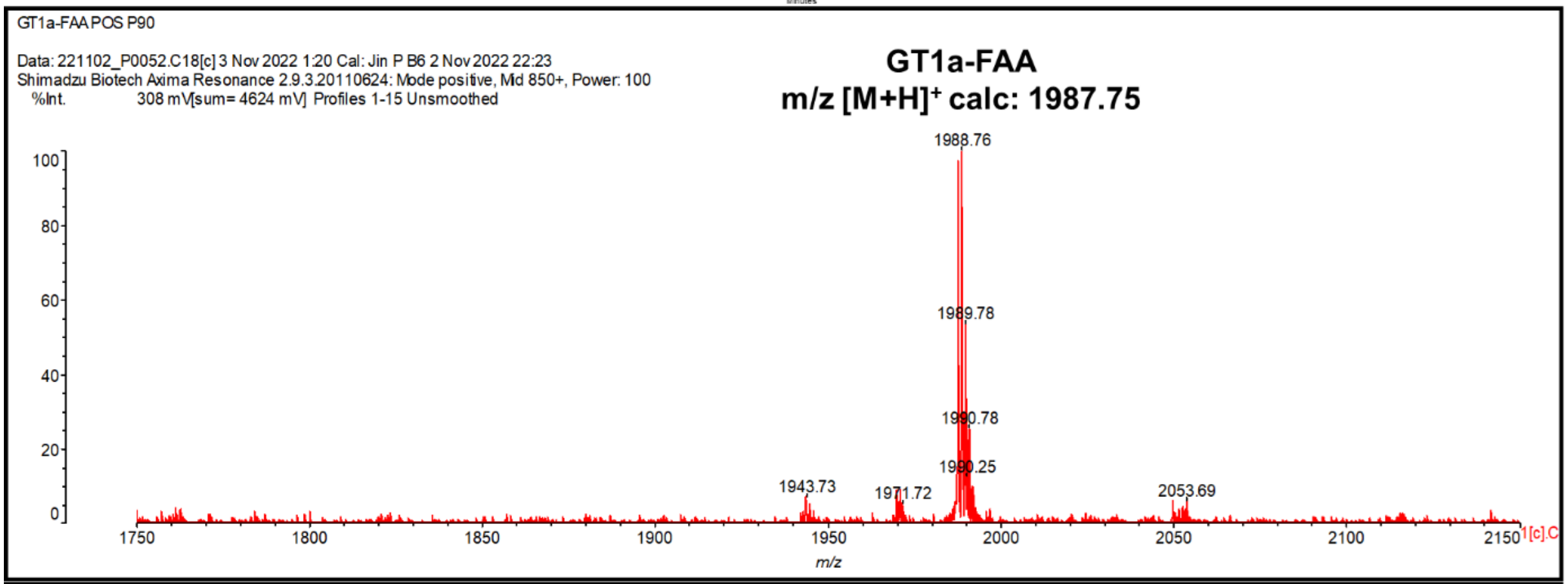

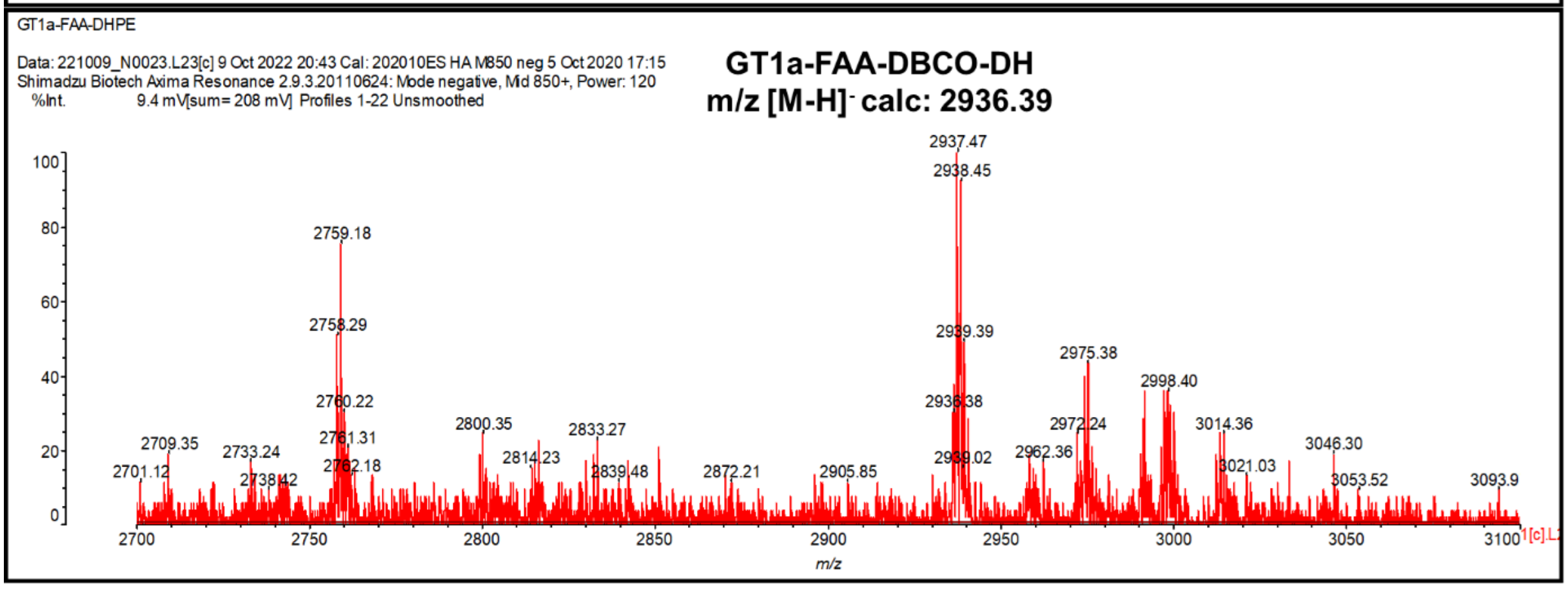

Probe No 34

GD2-FAA

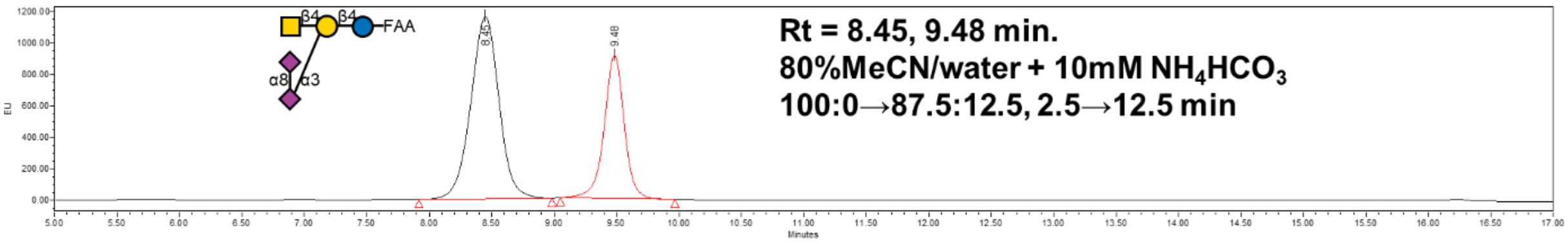

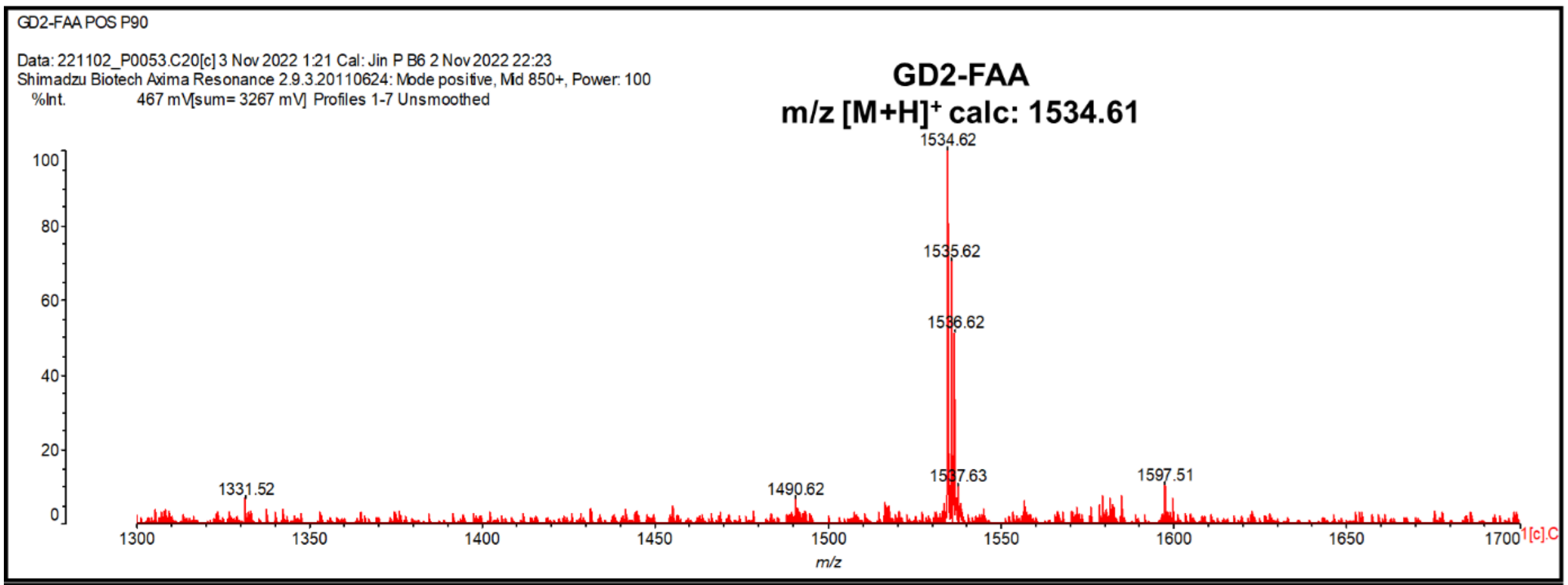

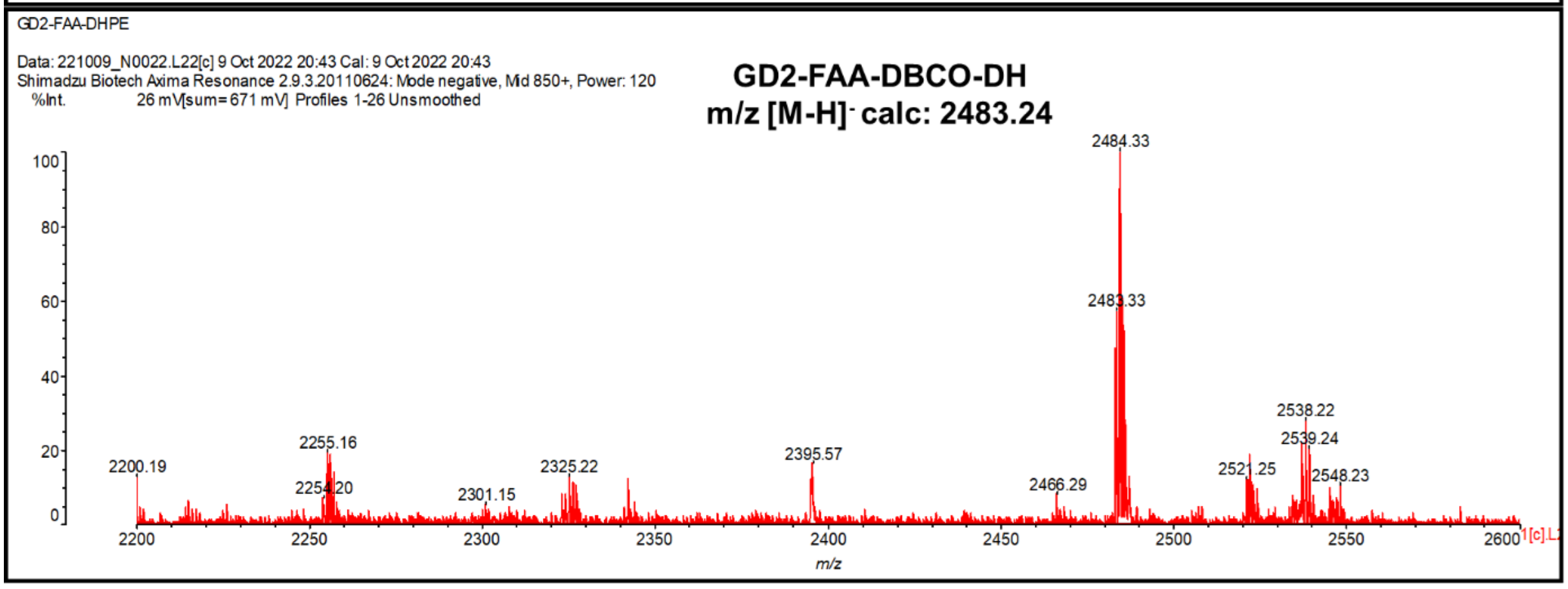

Probe No 35

GD1b-FAA

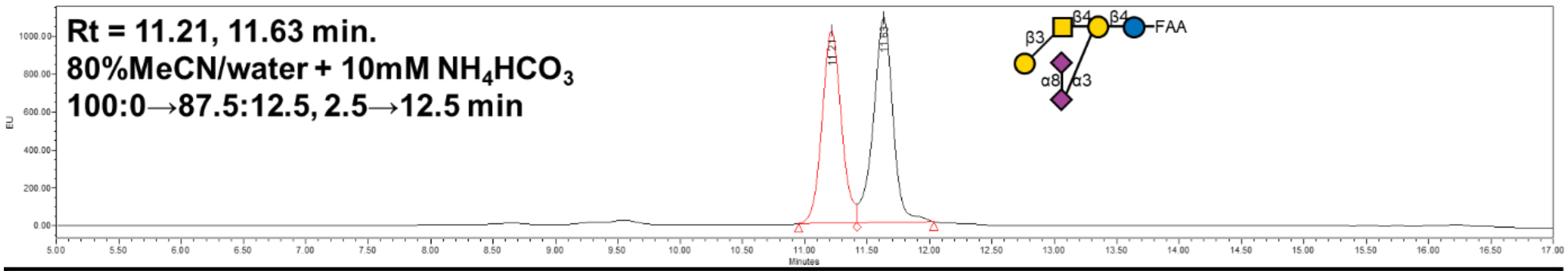

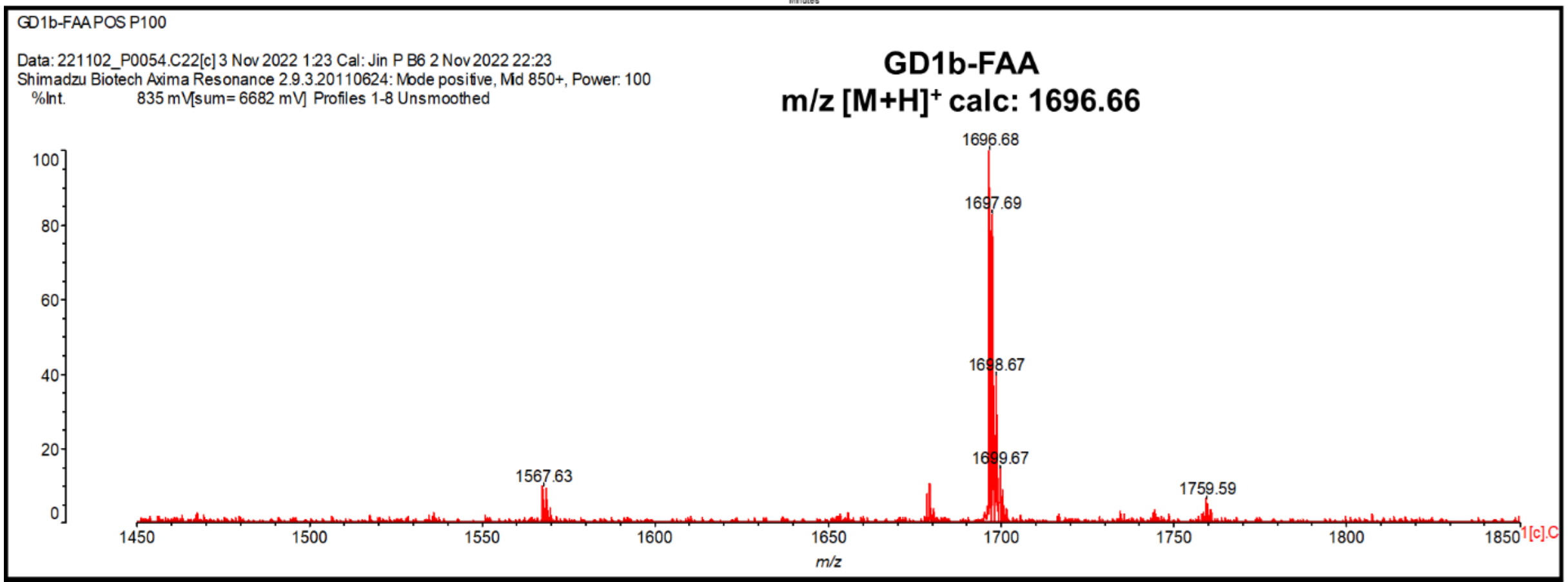

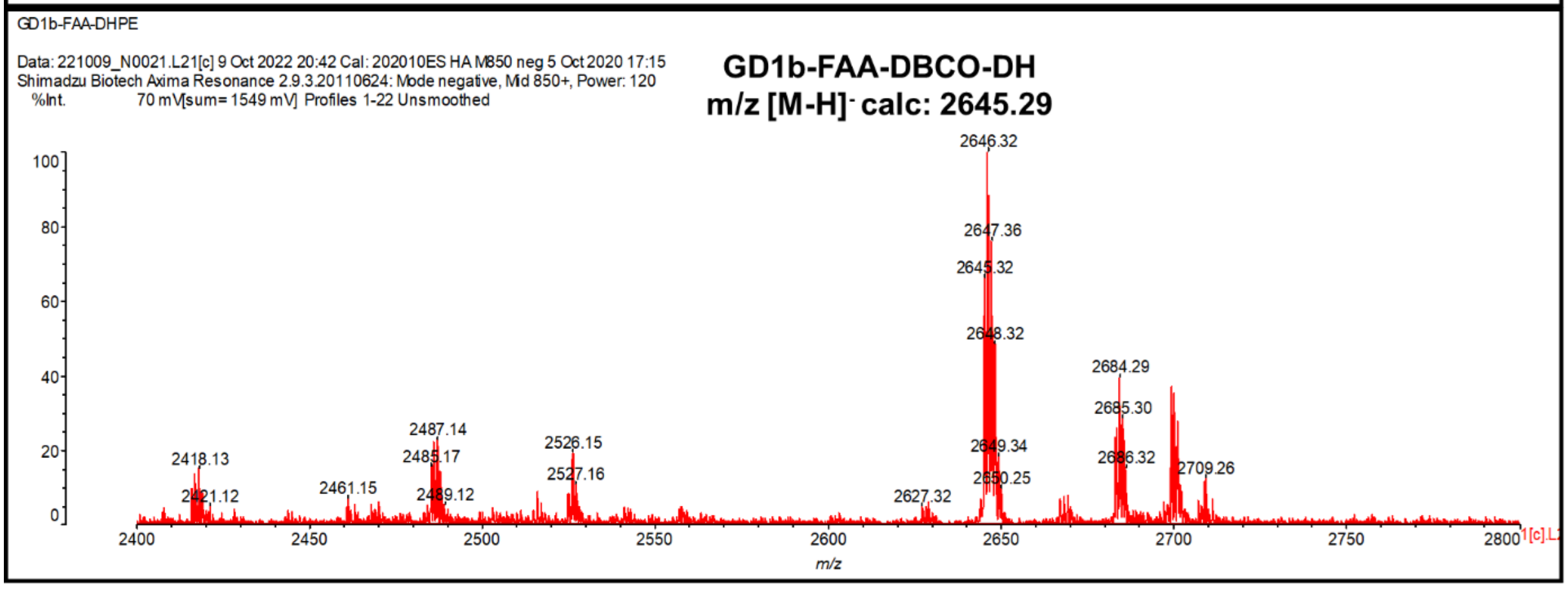

Probe No 36

H5N4-FAA

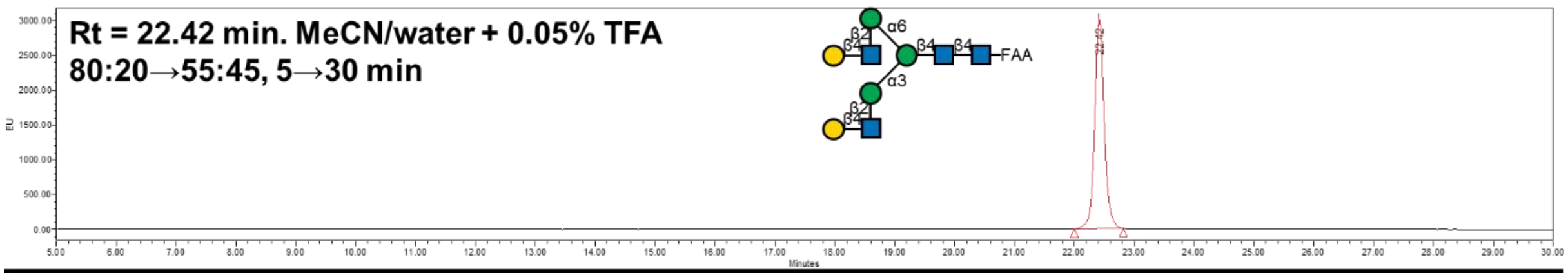

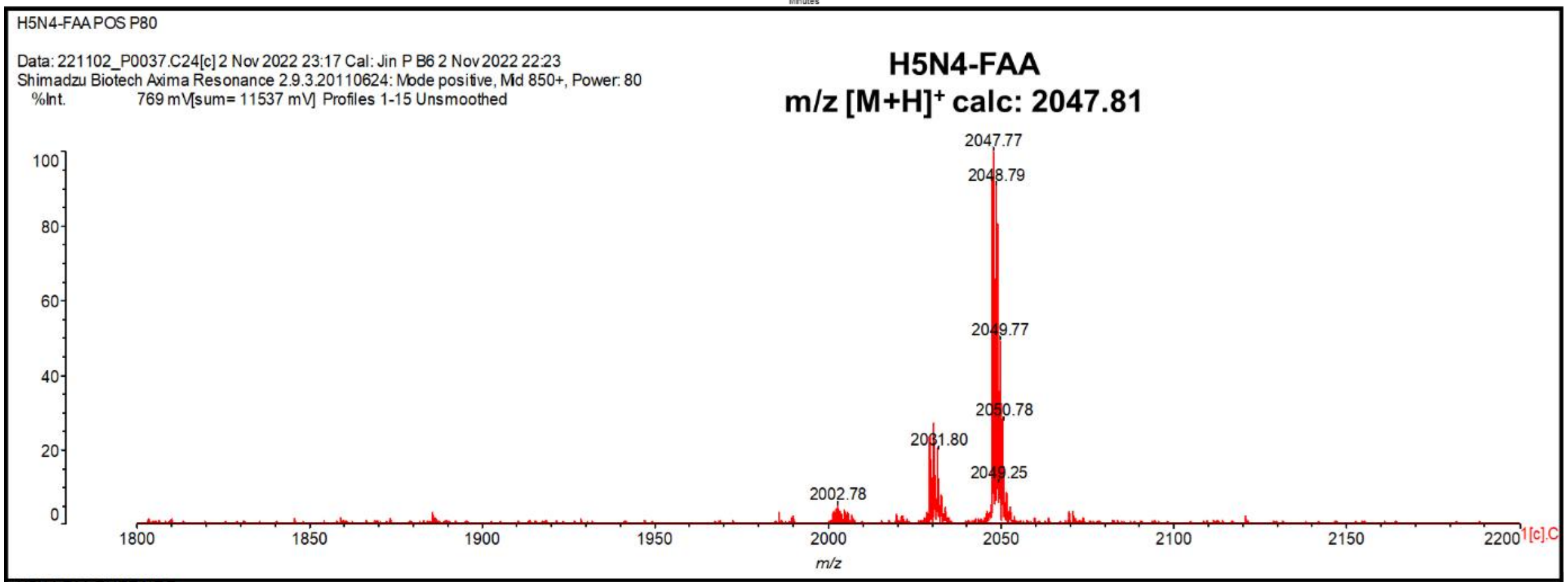

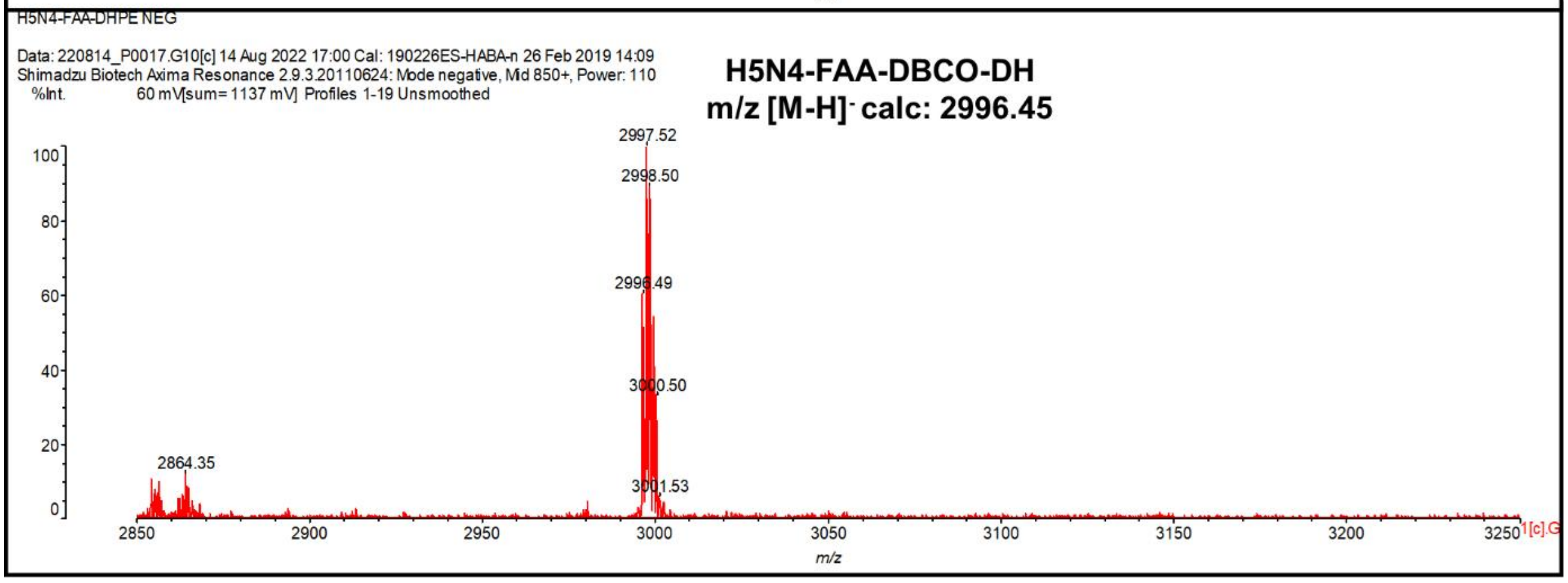

Probe No 37

F2H5N4(H2)-FAA

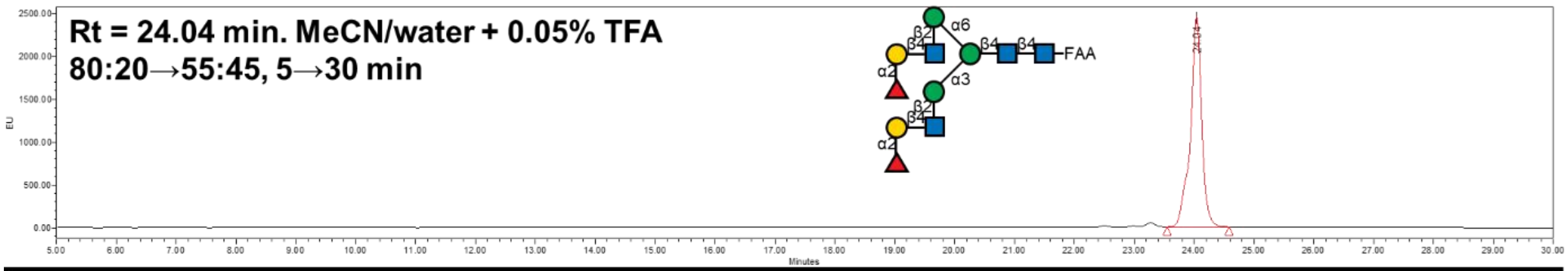

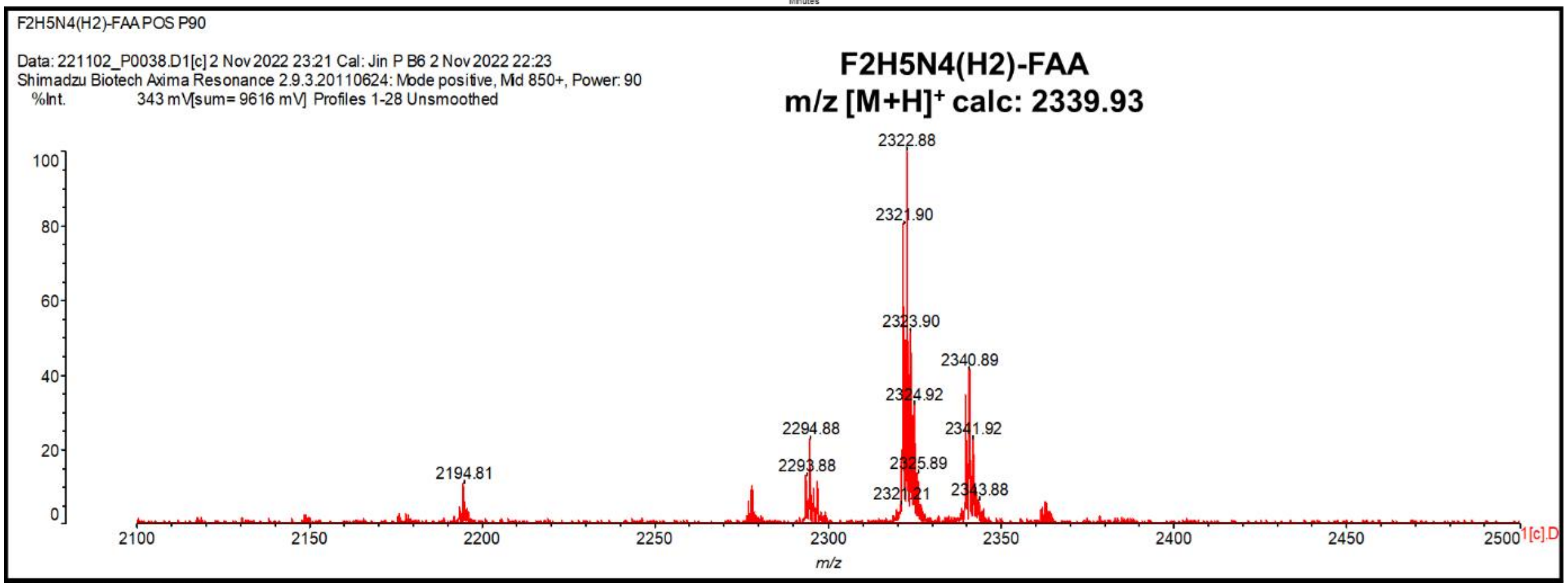

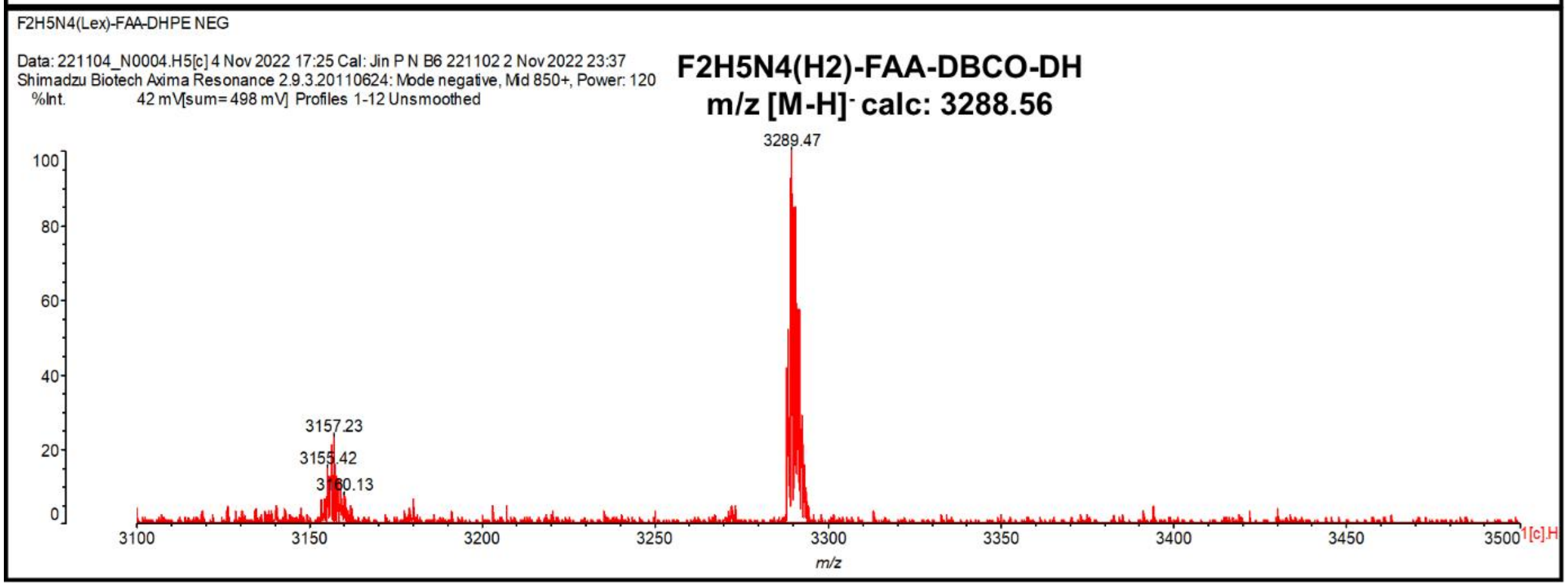

Probe No 38

F2H5N4(Lex)-FAA

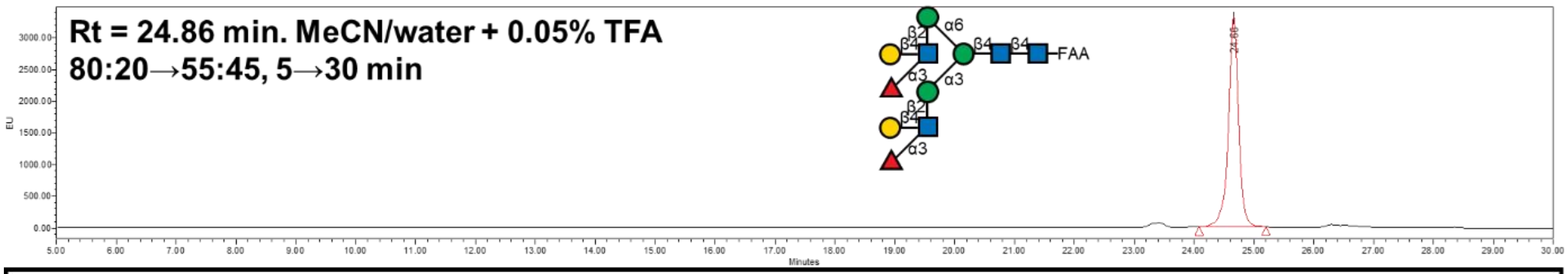

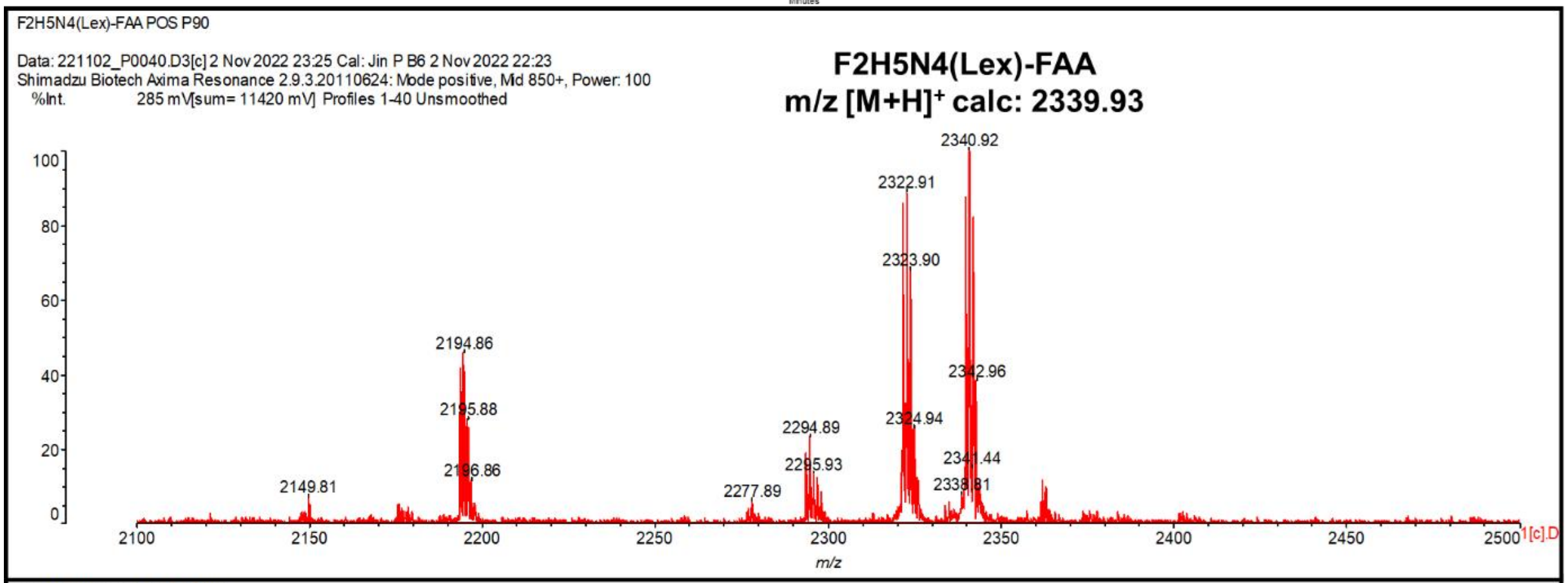

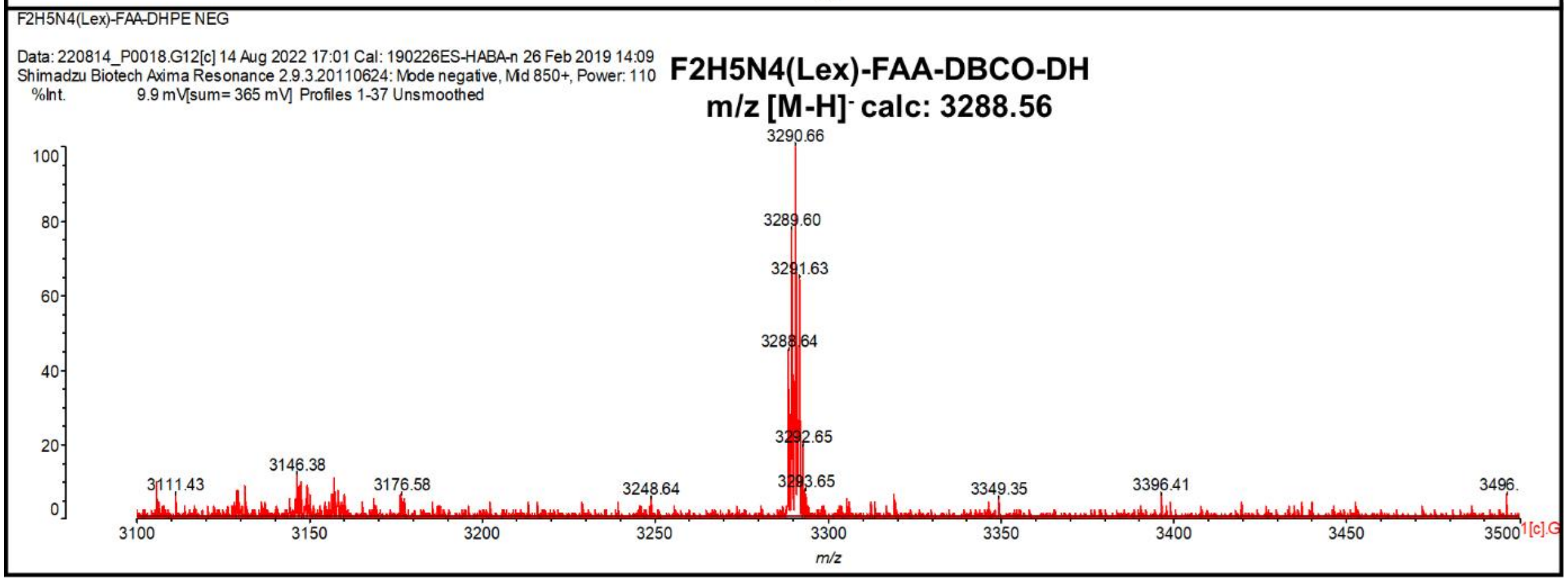

Probe No 9

F4H5N4(Ley)-FAA

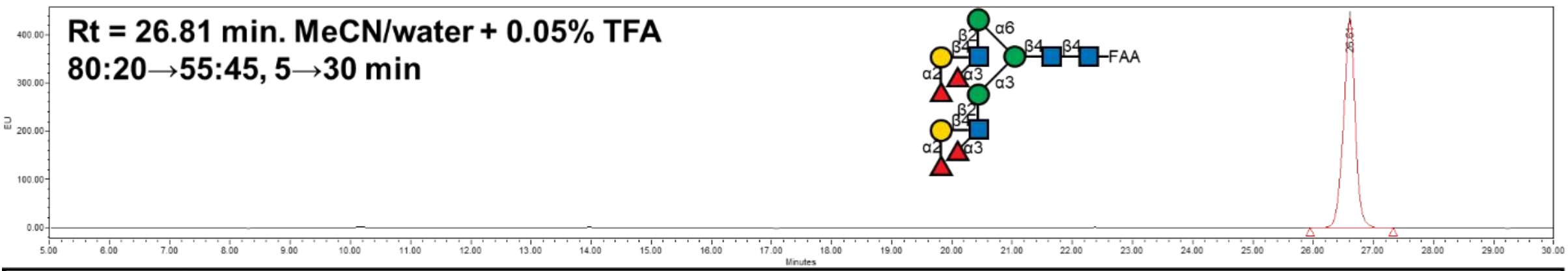

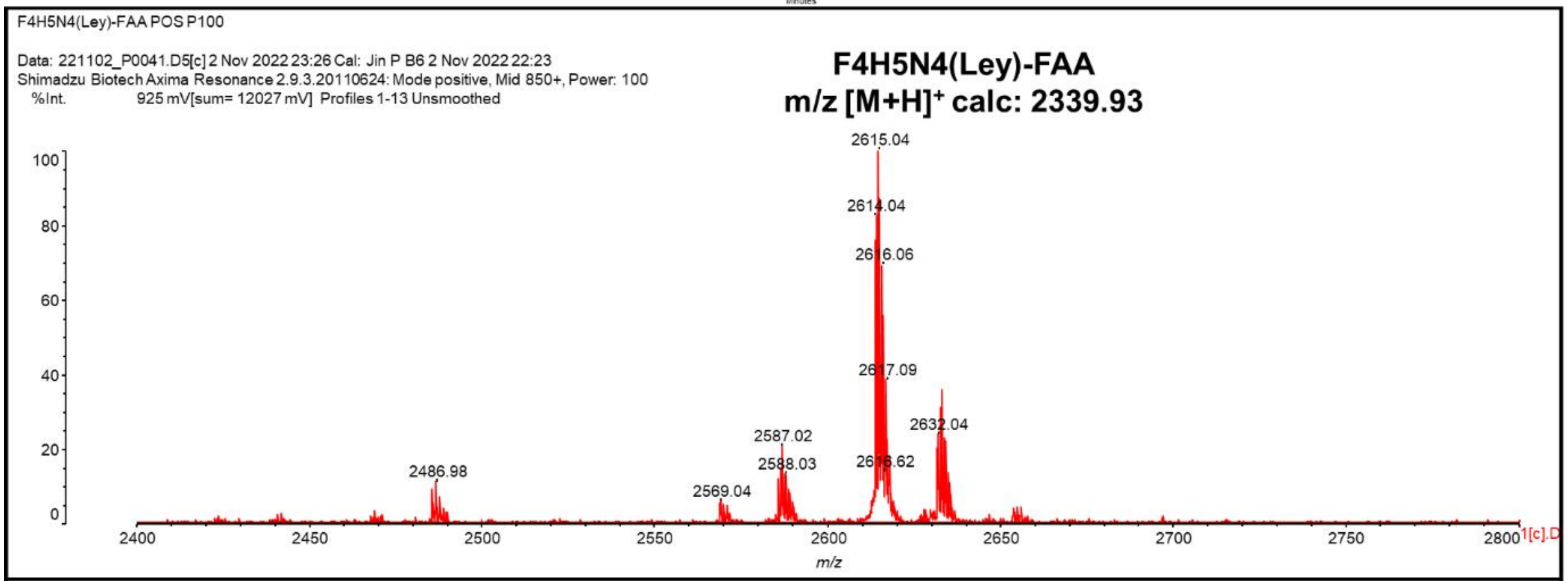

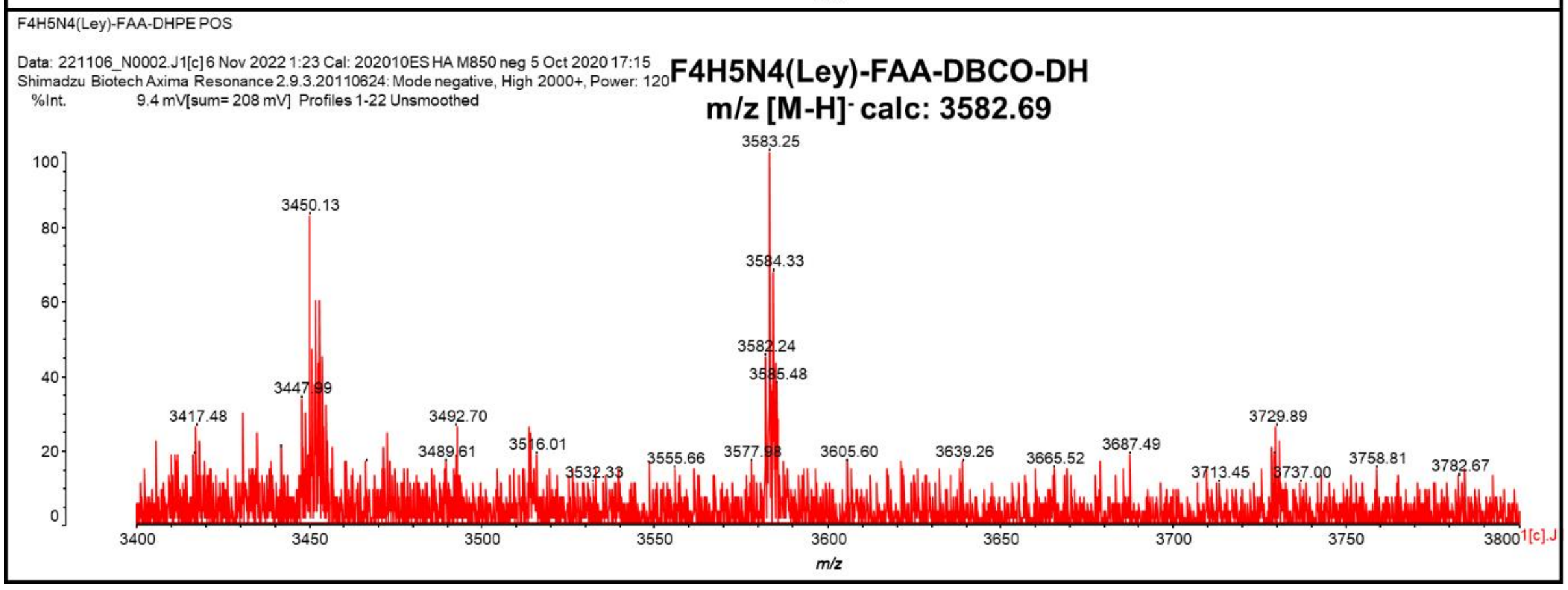

Probe No 40

A2H5N4(2-3Sia)-FAA

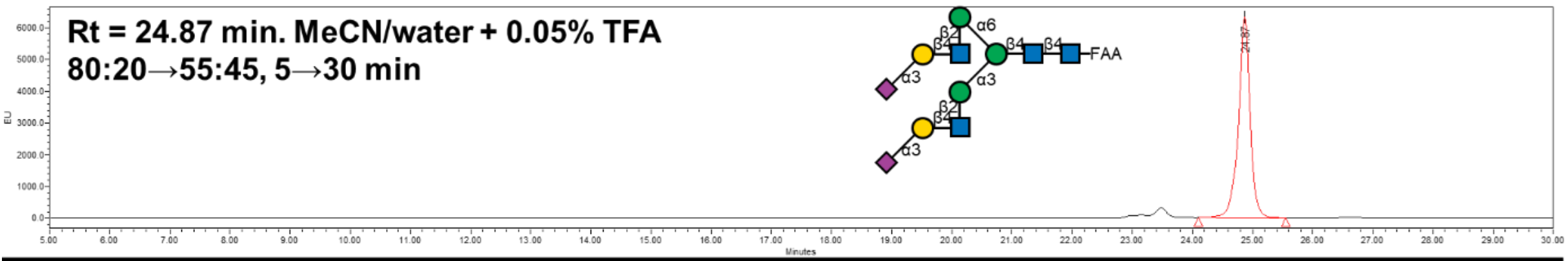

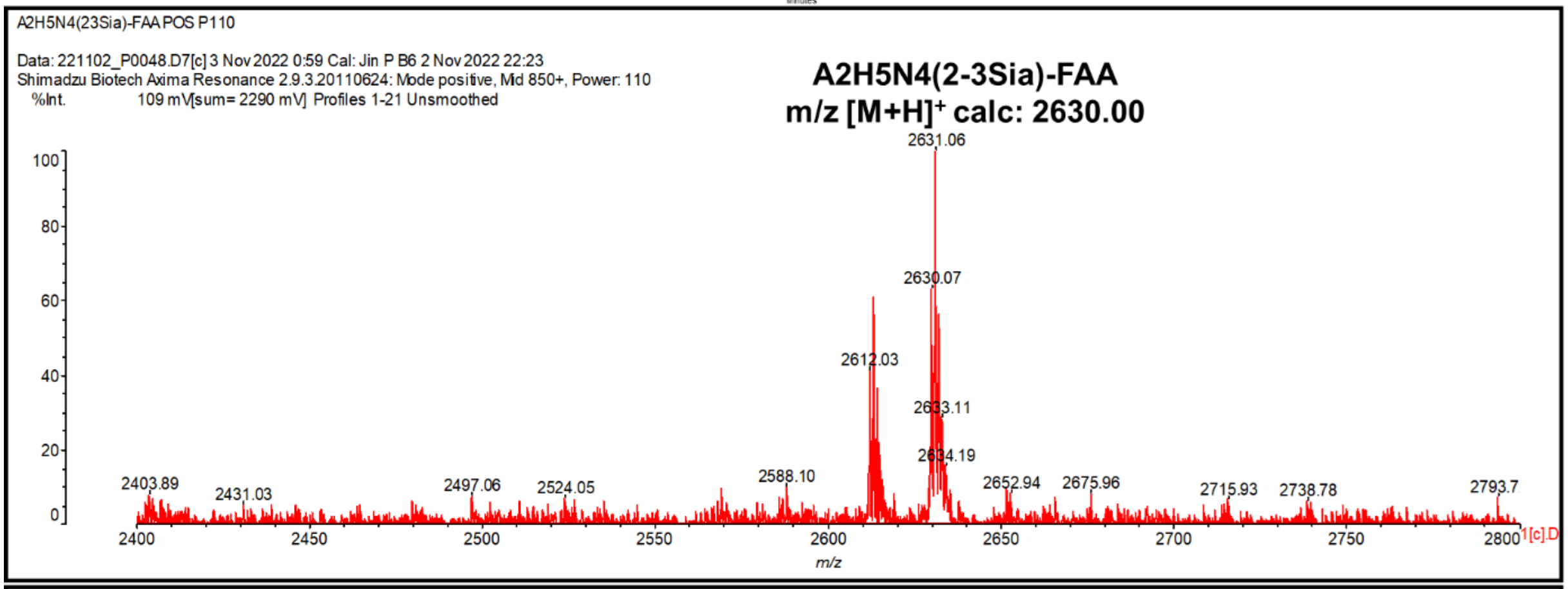

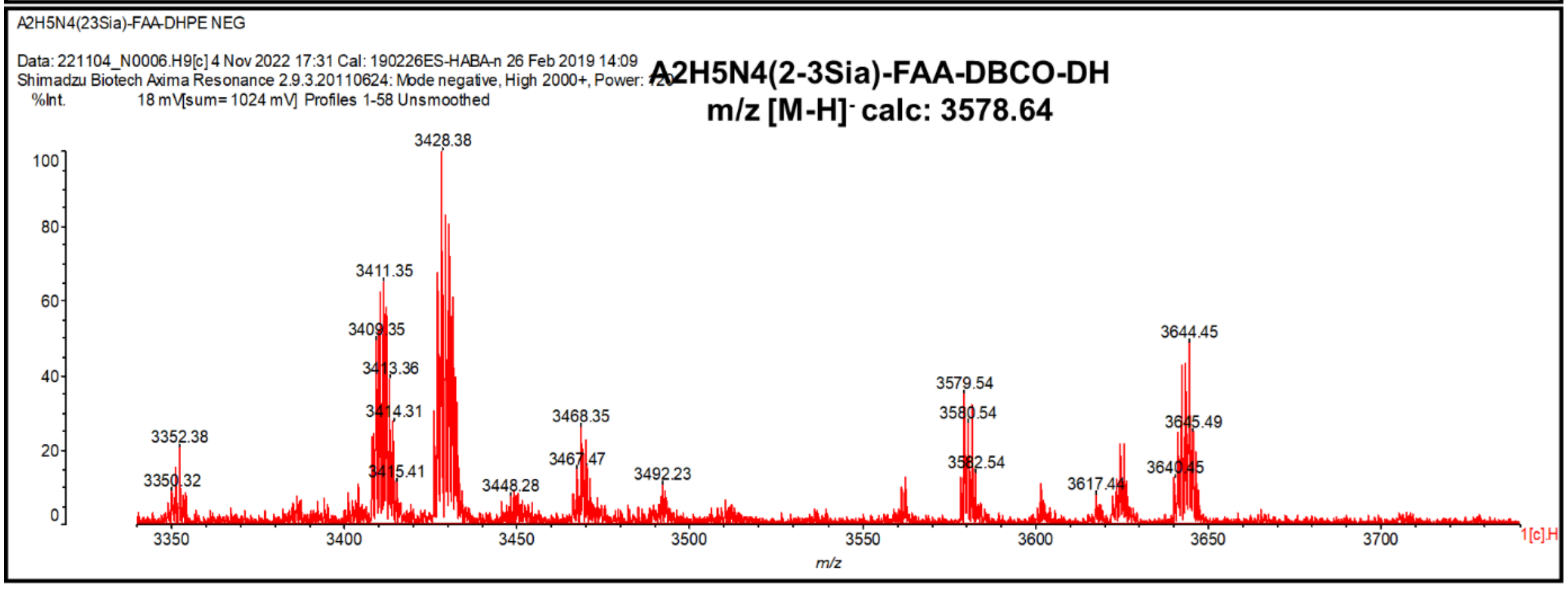

Probe No 41

A2F2H5N4(SLex)-FAA

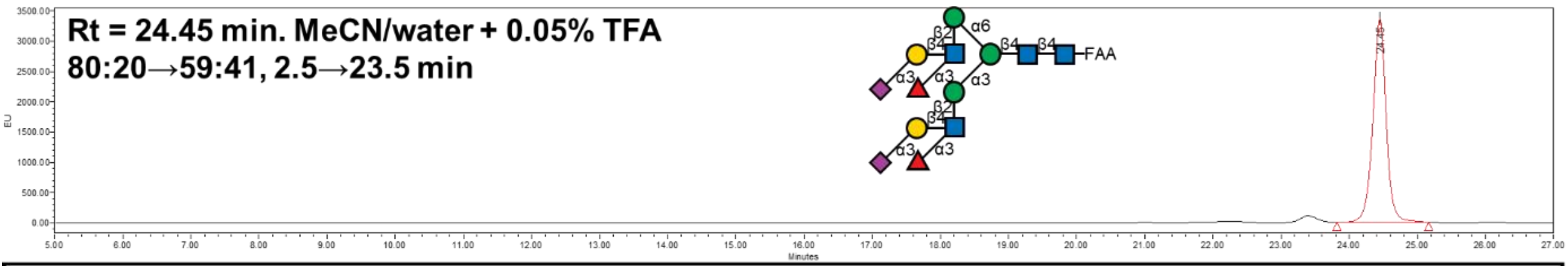

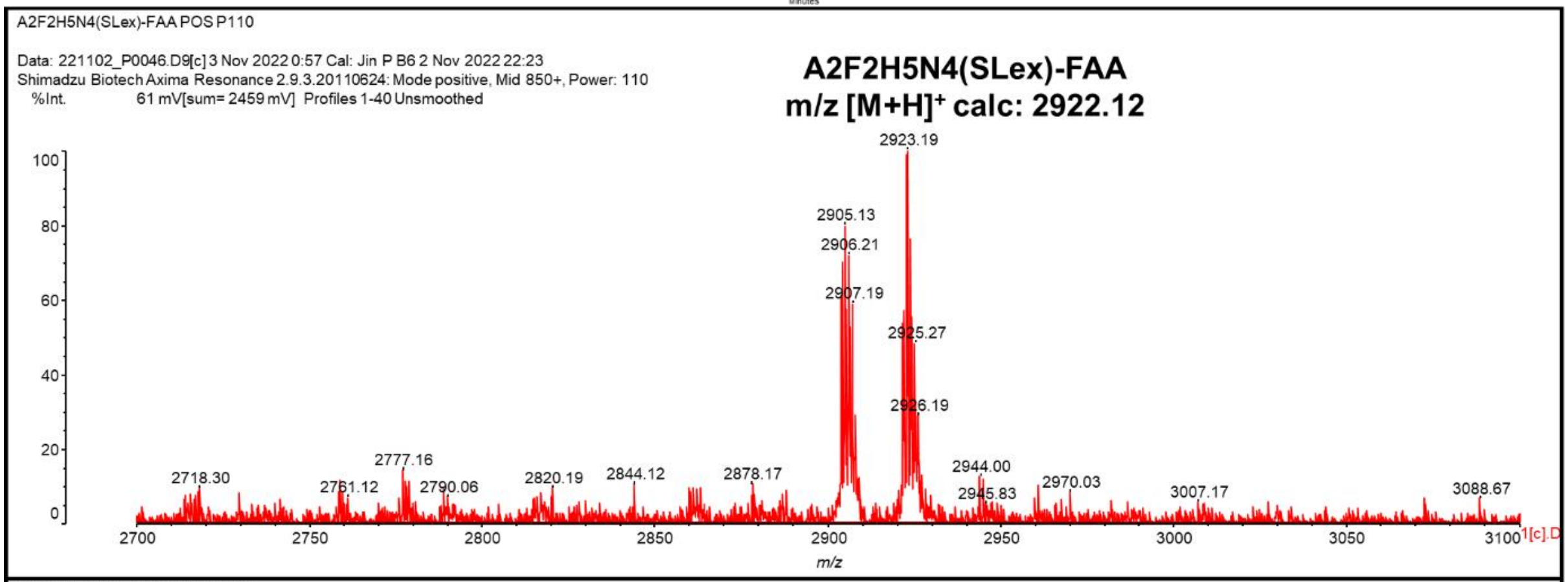

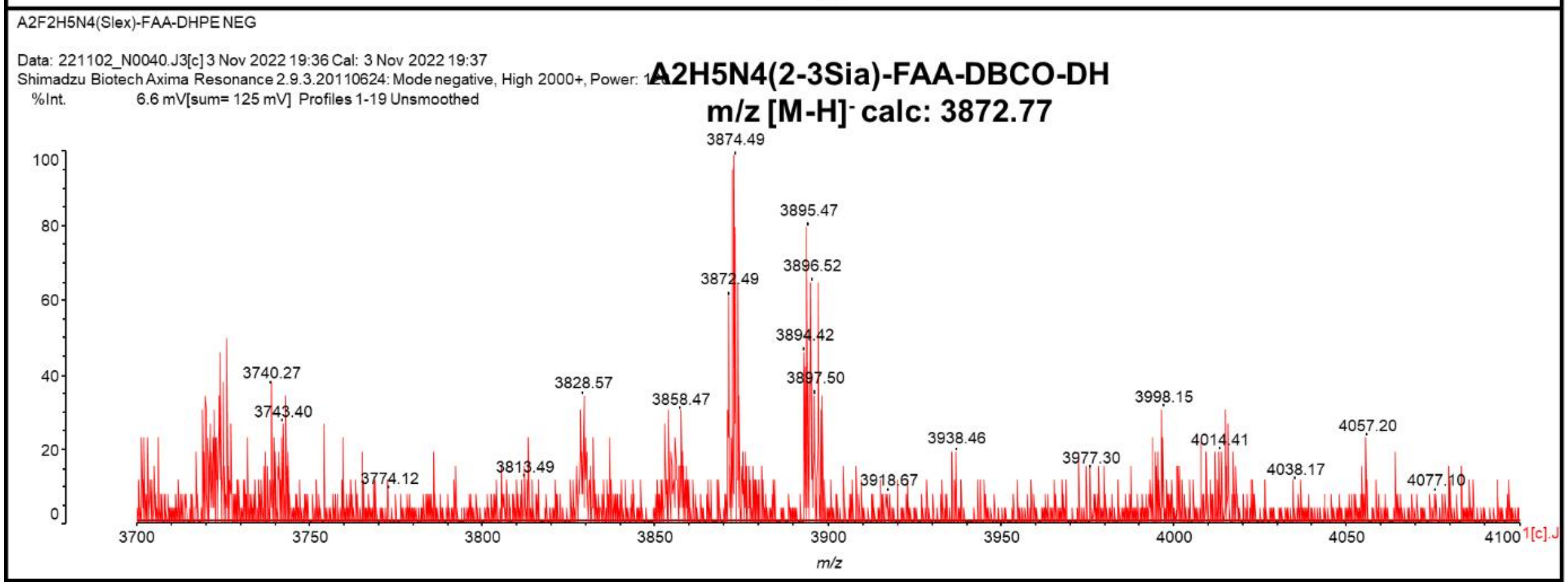

Probe No 42

A2H5N4(2-6Sia)-FAA

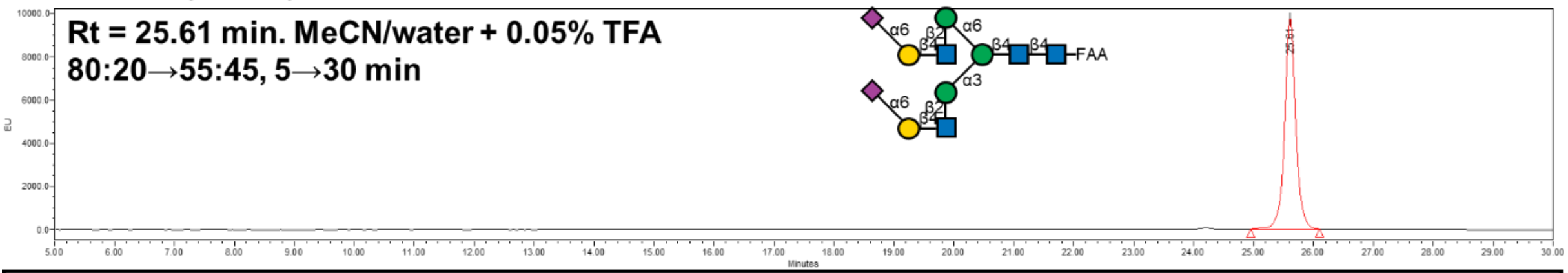

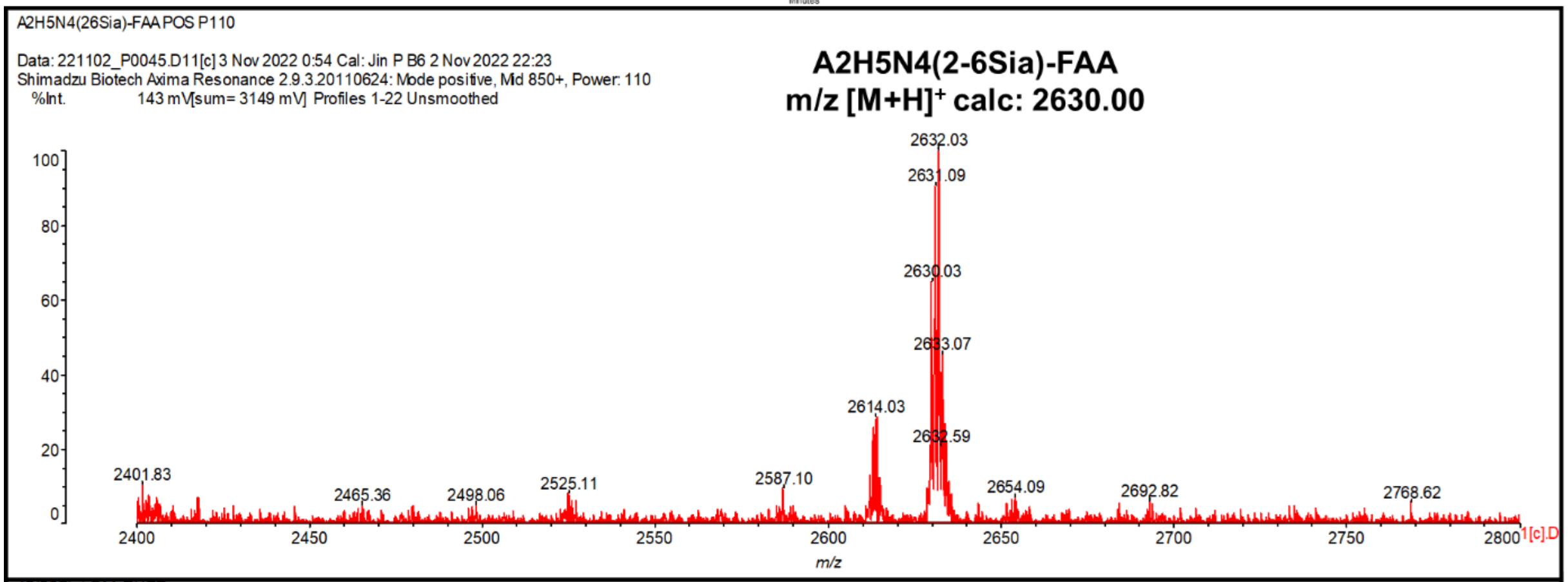

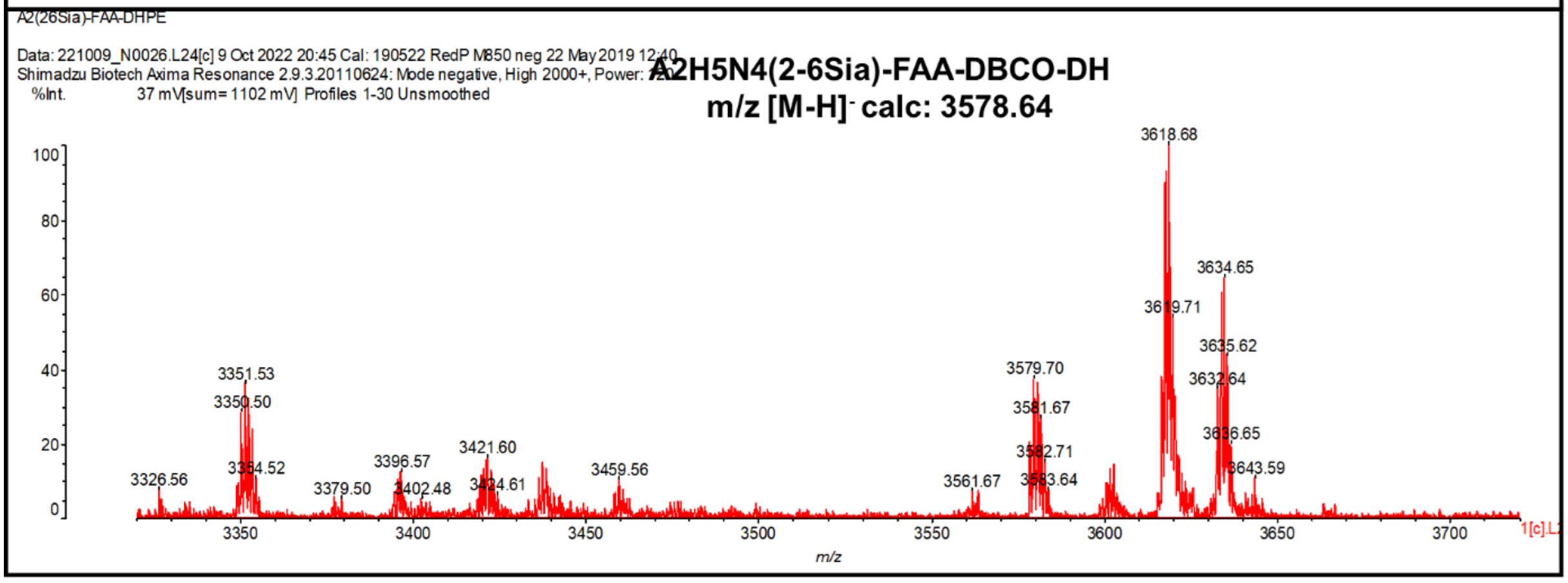

Probe No 43

M5N2-FAA

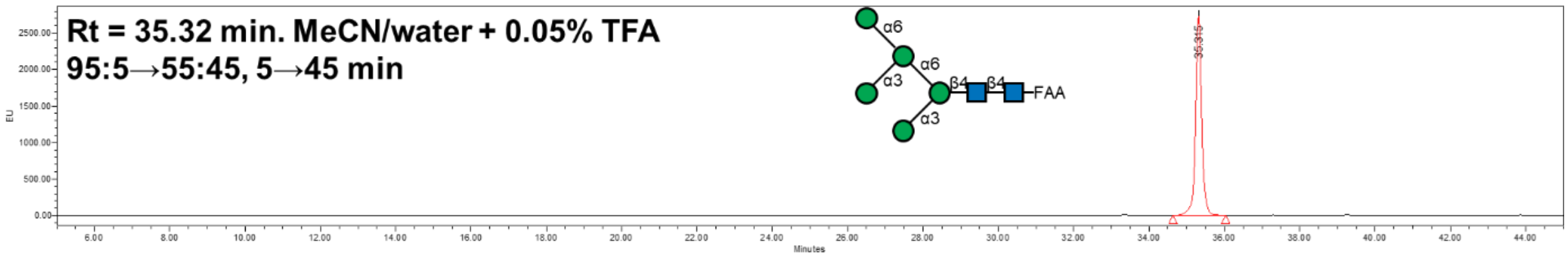

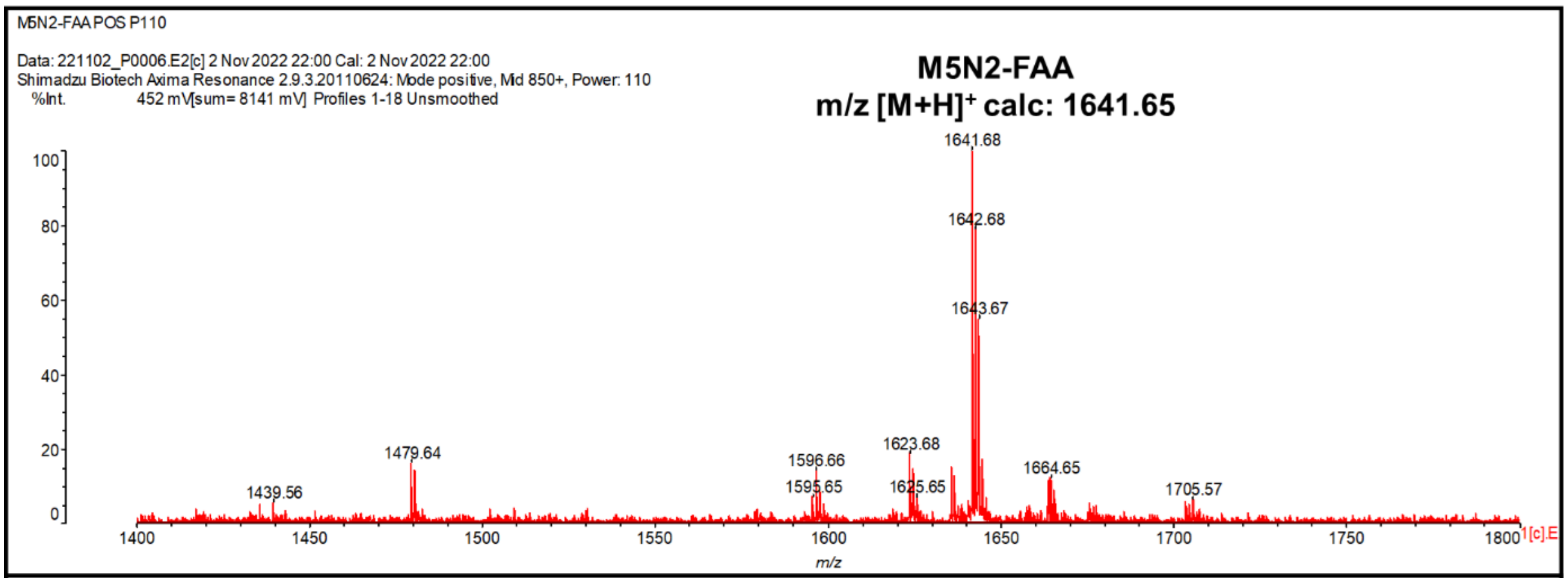

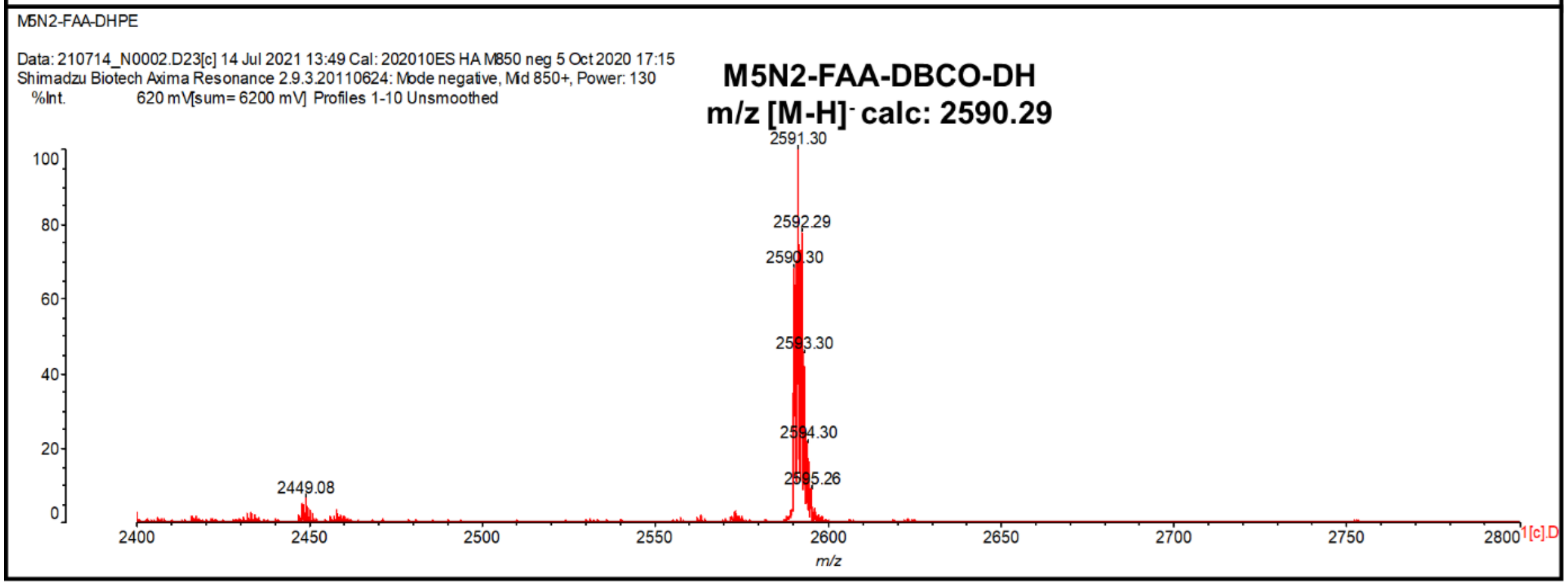

Probe No 44

M9N2-FAA

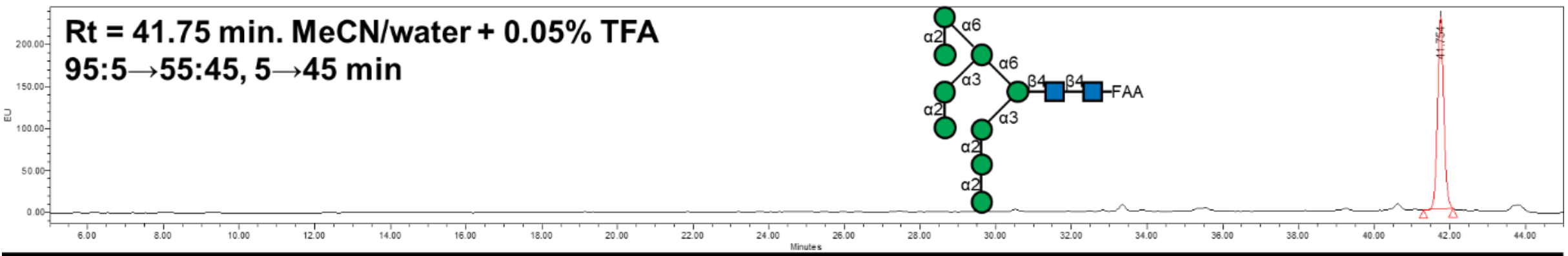

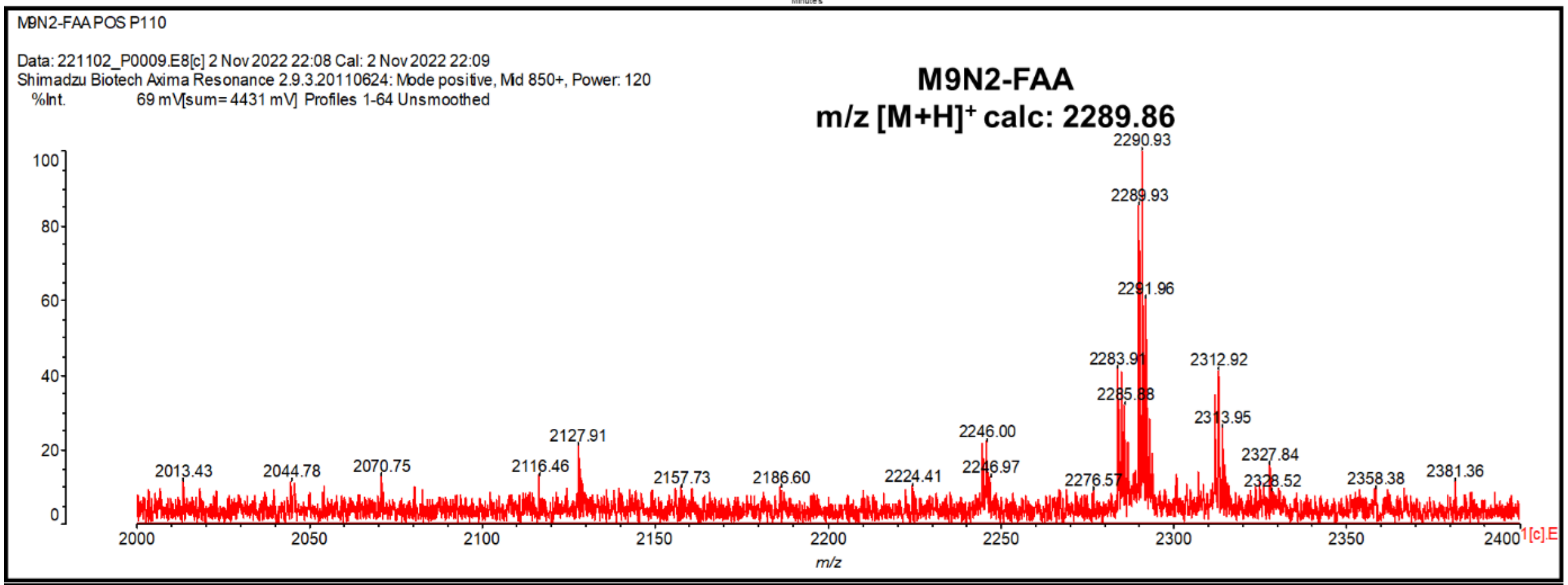

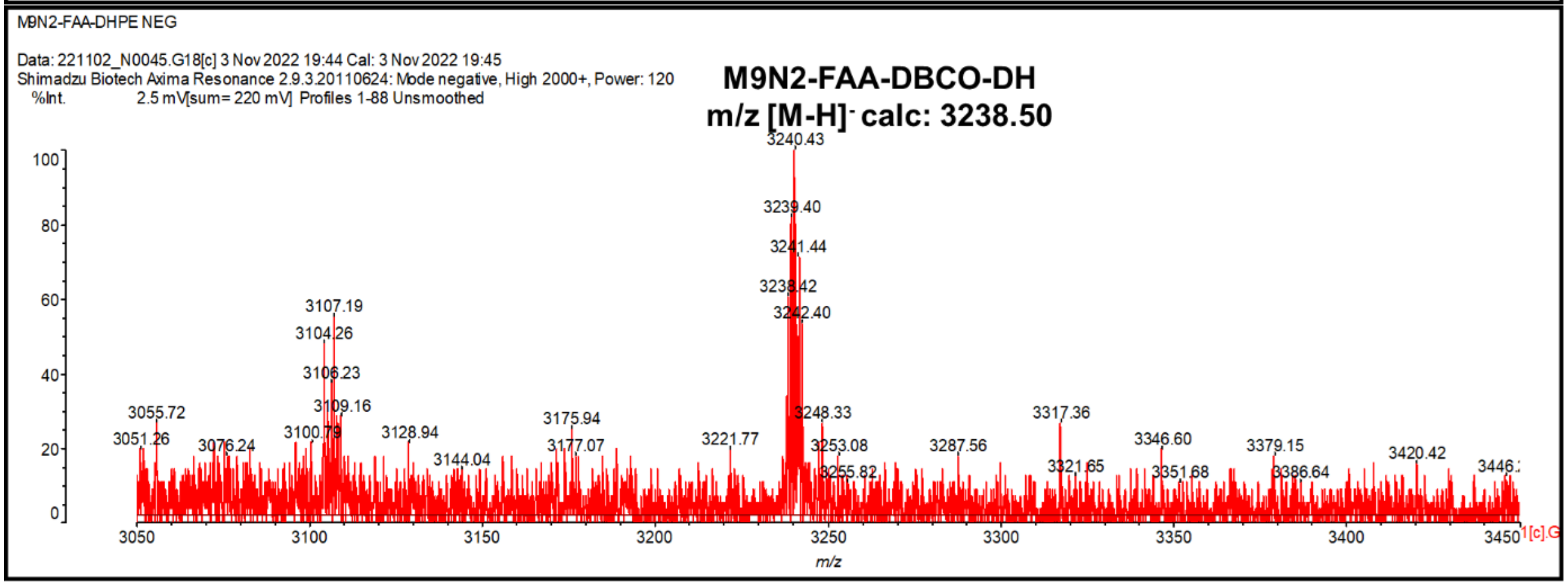

Supplementary Fig. 16. Examples of MALDI spectra of Fmoc deprotected glycan probes.

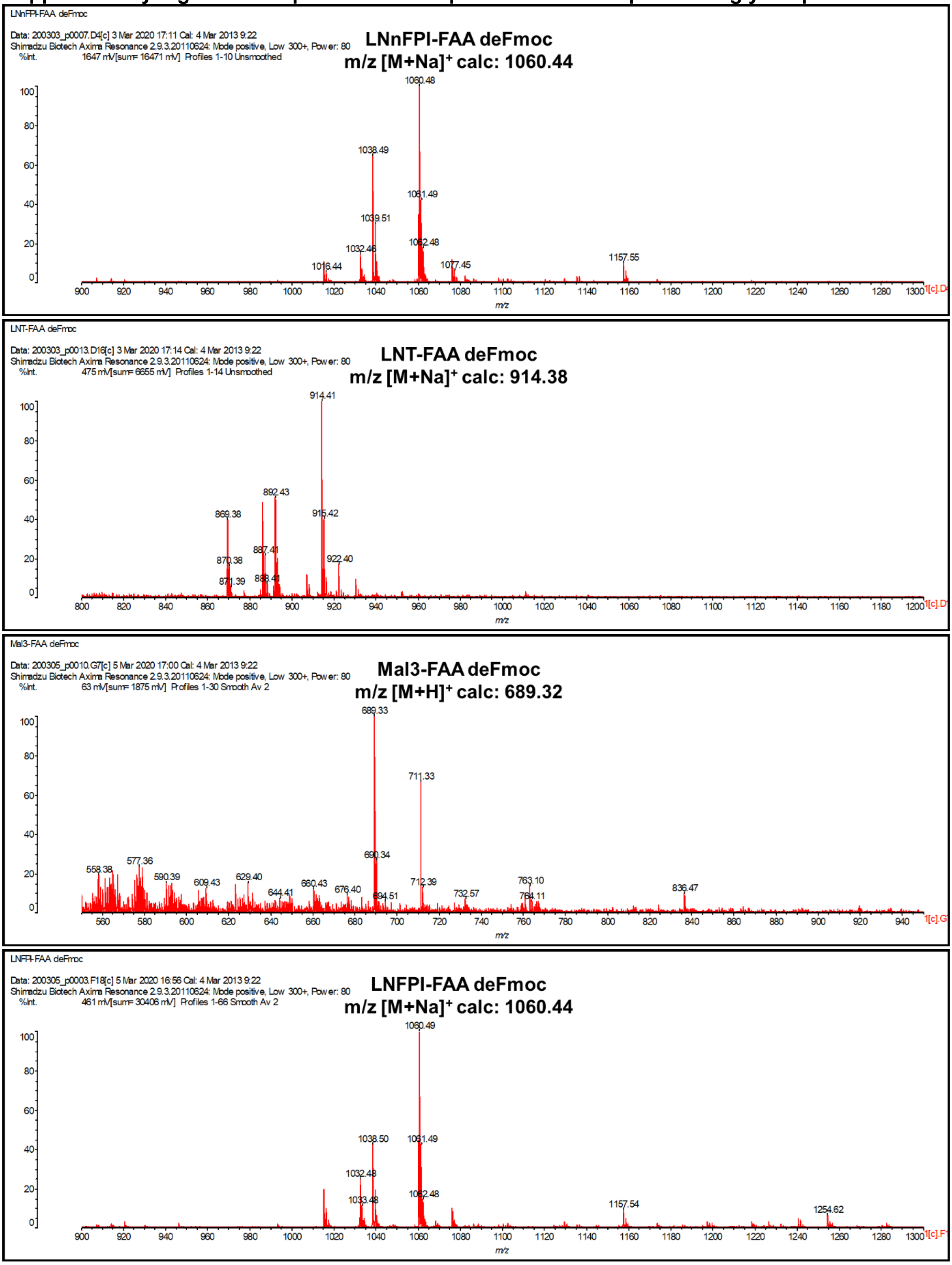

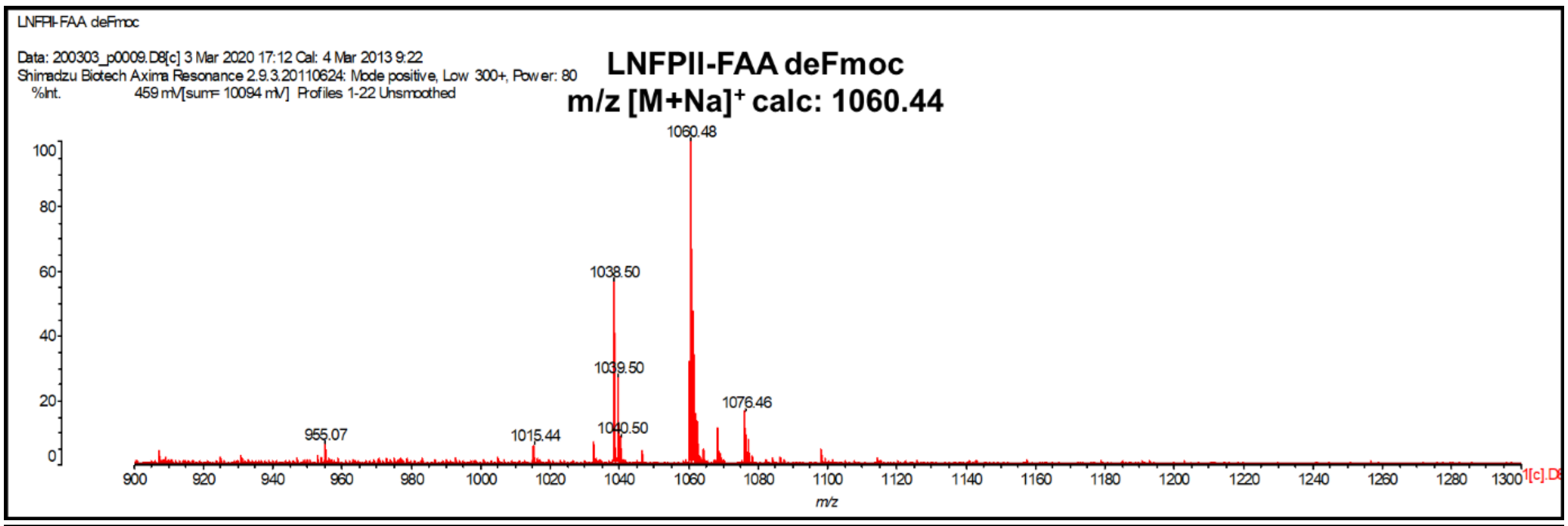
LNFPII-FAA deFmoc
m/z [M+Na]+ calc: 1060.44
1060.48
1038.50
1039.50
1076.46
1040.50
1015.44
955.07
m/z

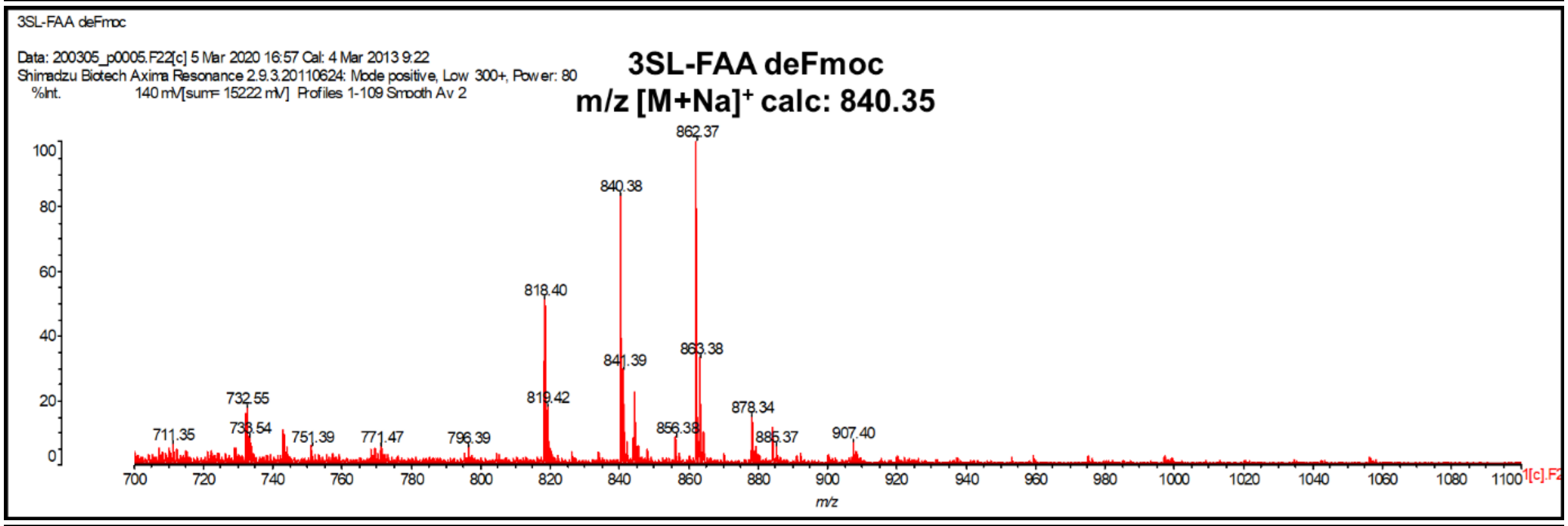
3SL-FAA deFmoc
m/z [M+Na]+ calc: 840.35
862.37
840.38
818.40
841.39
863.38
819.42
732.55
878.34
733.54
856.38
885.37
907.40
711.35
751.39
771.47
796.39
m/z

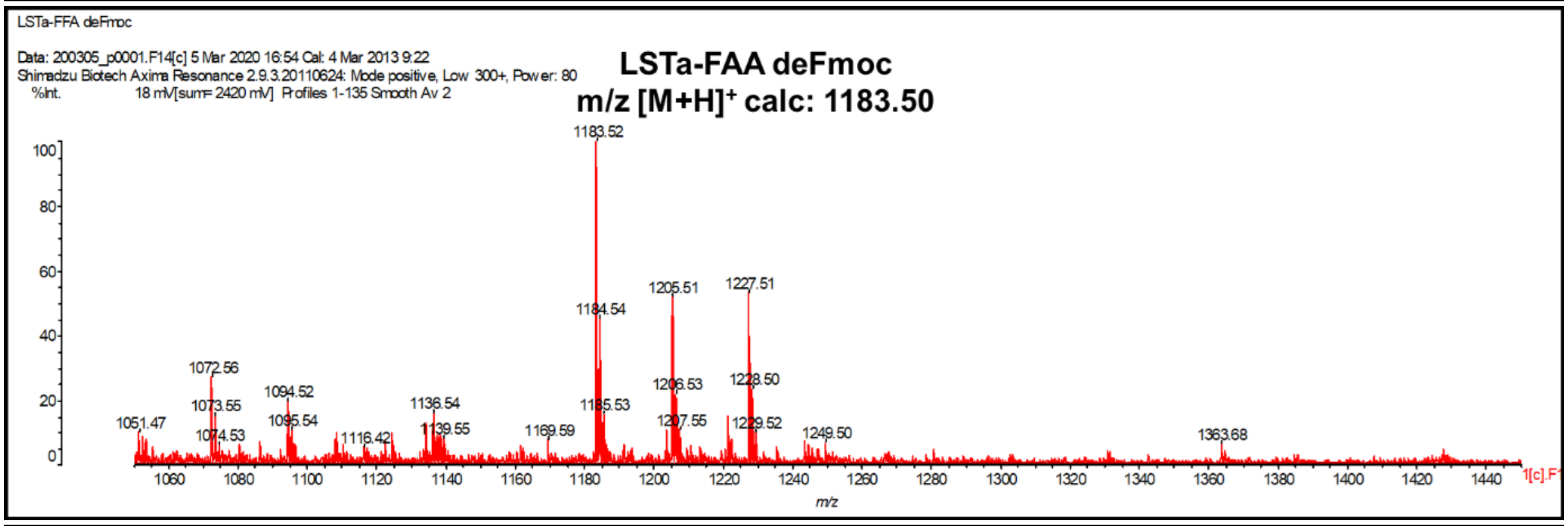
LSTa-FAA deFmoc
m/z [M+H]+ calc: 1183.50
1183.52
1205.51
1227.51
1184.54
1072.56
1206.53
1228.50
1094.52
1136.54
1073.55
1185.53
1051.47
1095.54
1207.55
1229.52
1074.53
1116.42
1139.55
1169.59
1249.50
1363.68
m/z

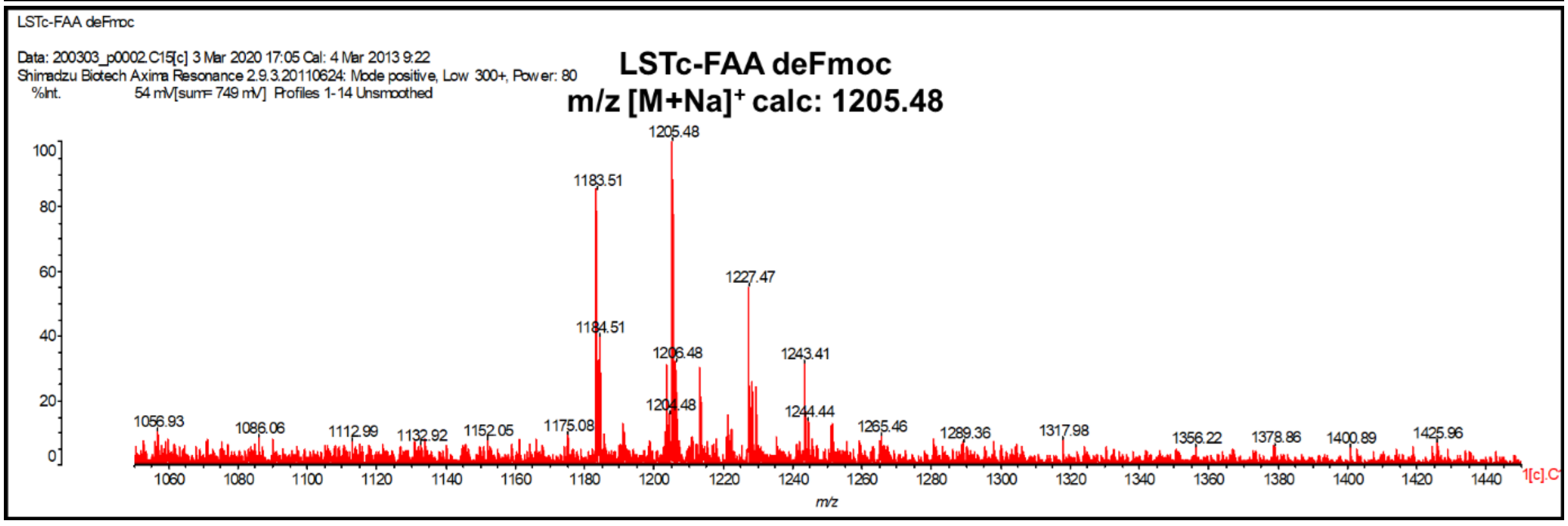
LSTc-FAA deFmoc
m/z [M+Na]+ calc: 1205.48
1205.48
1183.51
1227.47
1184.51
1206.48
1243.41
1204.48
1244.44
1056.93
1086.06
1175.08
1112.99
1132.92
1152.05
1265.46
1289.36
1317.98
1356.22
1378.86
1400.89
1425.96
m/z

Supplementary Fig. 17. MALDI spectra of biotinylated glycan probes.

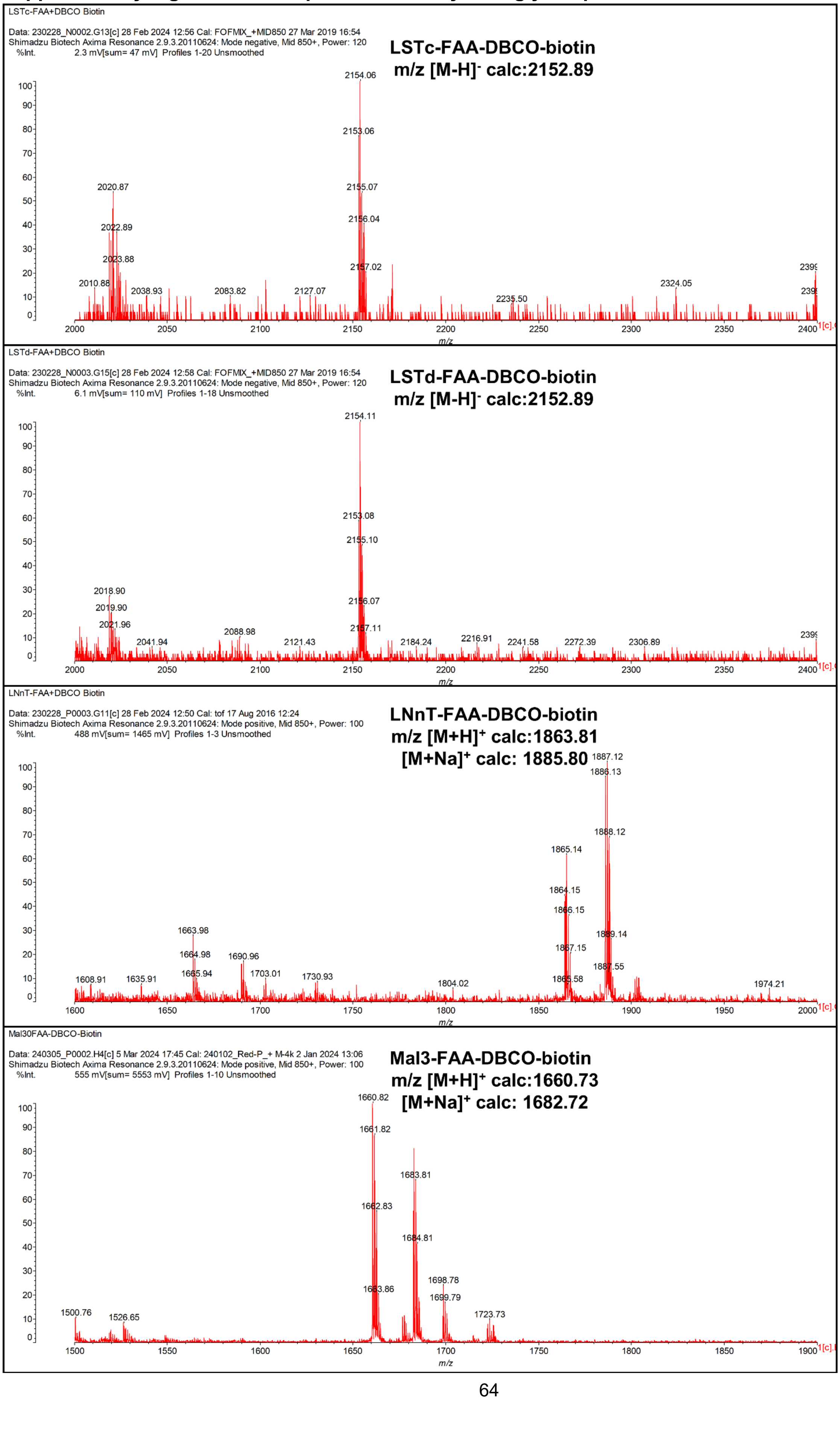

Supplementary Fig. 18. MALDI spectra of F_{545} labeled glycan probes.

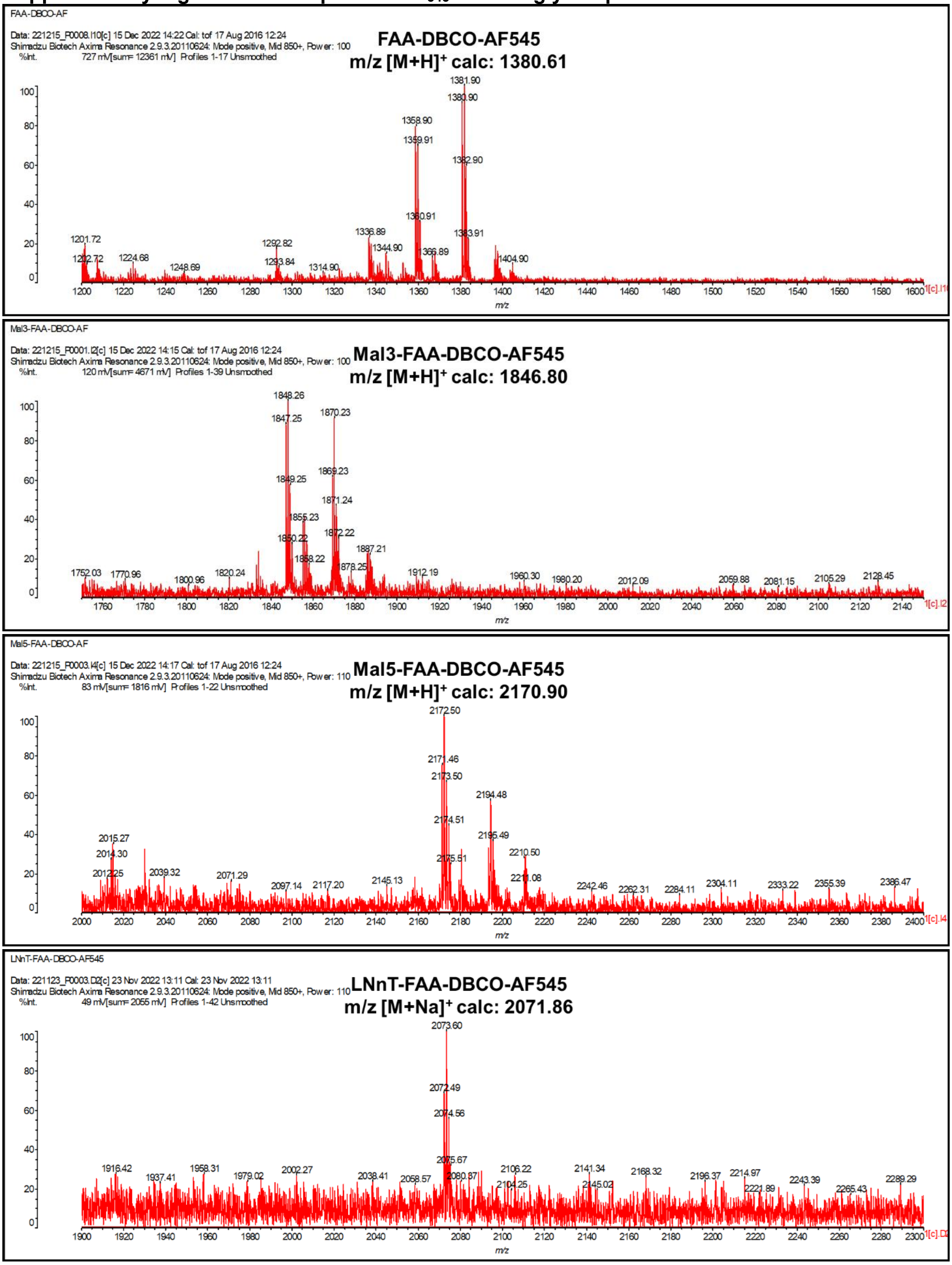

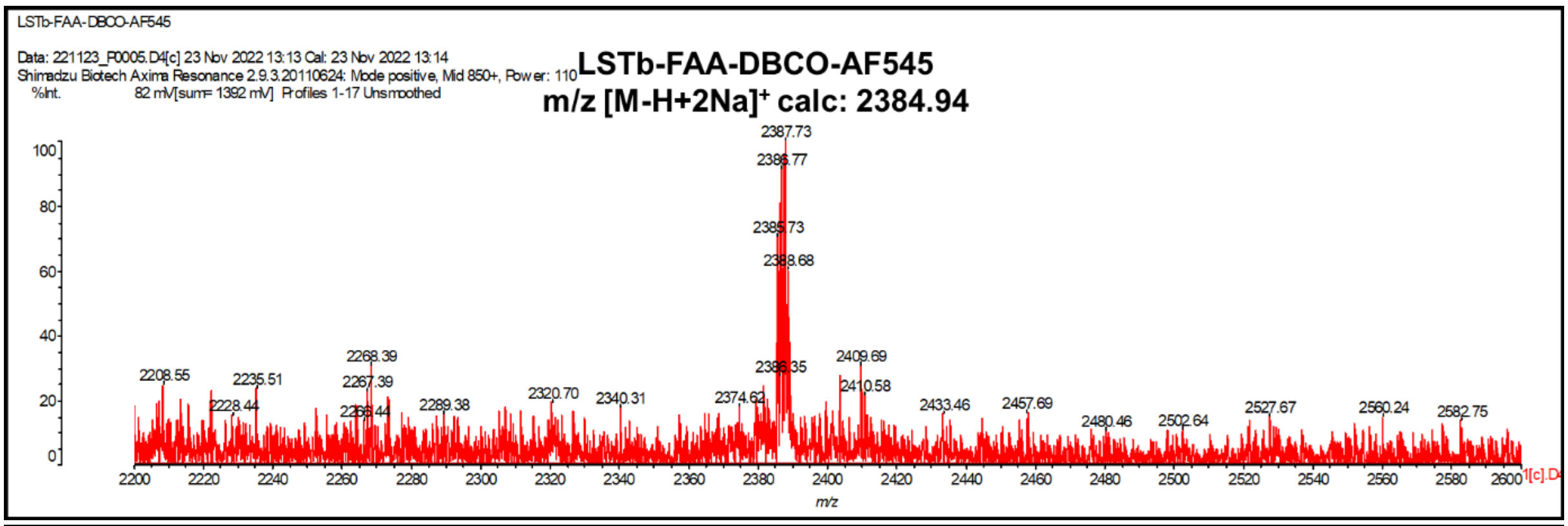
LSTb-FAA-DBCO-AF545
Data: 221123_P0005.D4[c] 23 Nov 2022 13:13 Cal: 23 Nov 2022 13:14
Shimadzu Biotech Axima Resonance 2.9.3.20110624: Mode positive, Mid 850+, Power: 110
%Int. 82 mV[sum= 1392 mV] Profiles 1-17 Unsmoothed
LSTb-FAA-DBCO-AF545
m/z [M-H+2Na]+ calc: 2384.94
2387.73
m/z

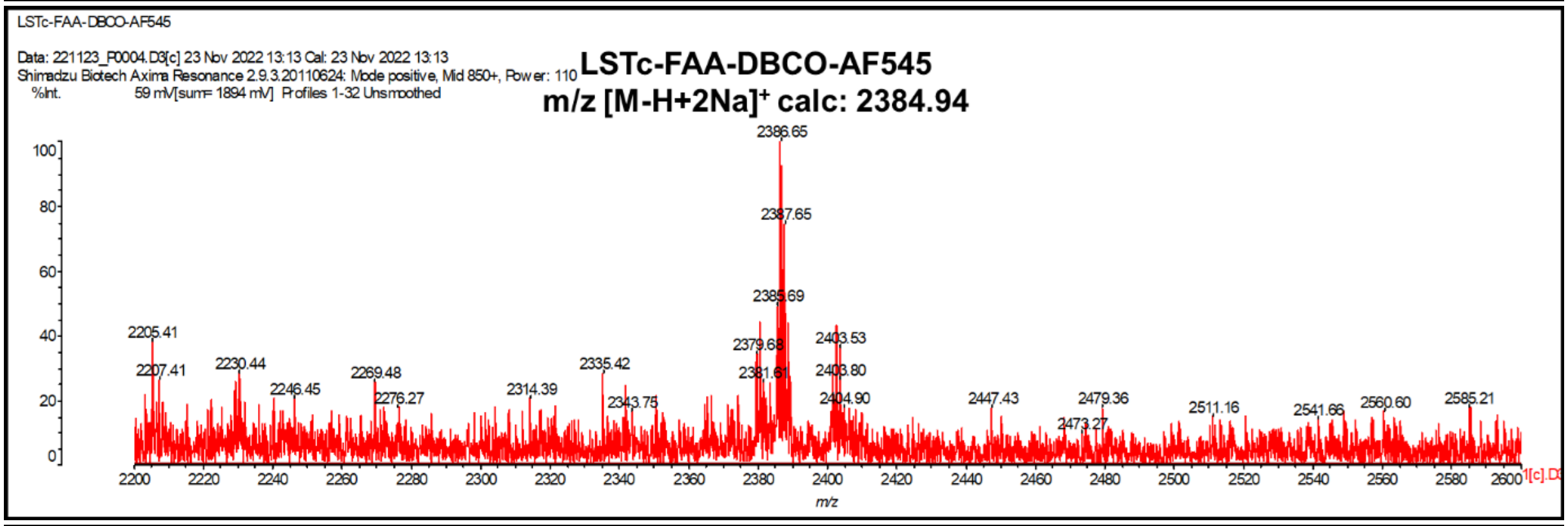
LSTc-FAA-DBCO-AF545
Data: 221123_P0004.D3[c] 23 Nov 2022 13:13 Cal: 23 Nov 2022 13:13
Shimadzu Biotech Axima Resonance 2.9.3.20110624: Mode positive, Mid 850+, Power: 110
%Int. 59 mV[sum= 1894 mV] Profiles 1-32 Unsmoothed
LSTc-FAA-DBCO-AF545
m/z [M-H+2Na]+ calc: 2384.94
2386.65
m/z

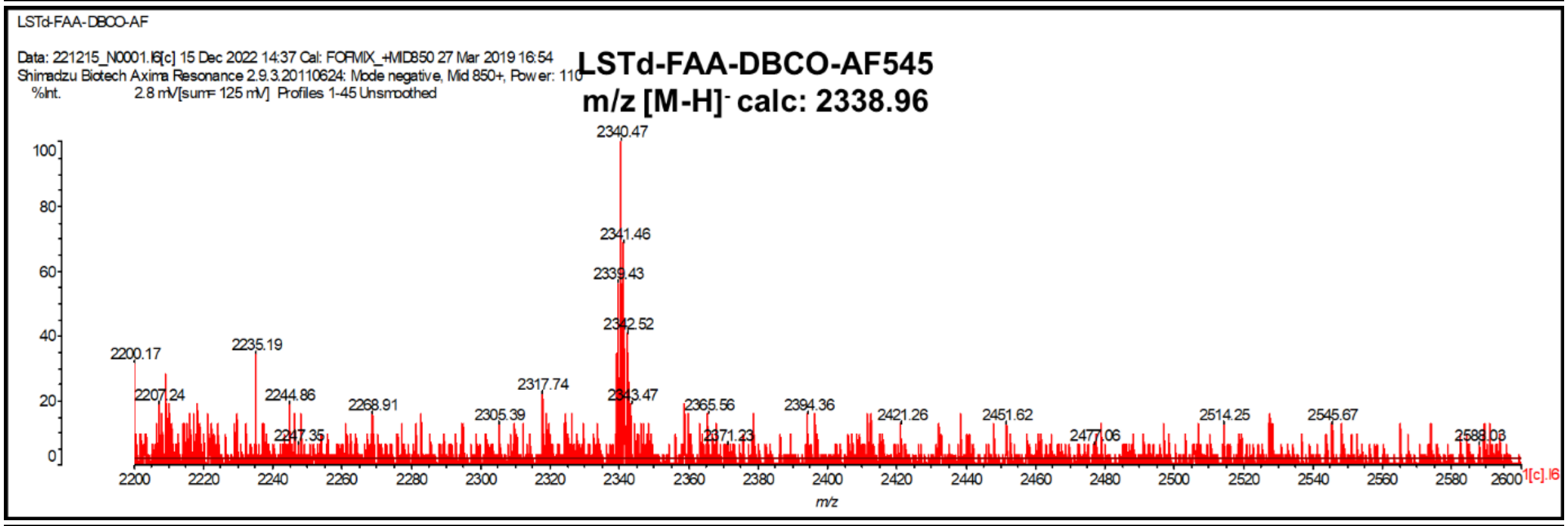
LSTd-FAA-DBCO-AF
Data: 221215_N0001.I6[c] 15 Dec 2022 14:37 Cal: FORMIX_+MID850 27 Mar 2019 16:54
Shimadzu Biotech Axima Resonance 2.9.3.20110624: Mode negative, Mid 850+, Power: 110
%Int. 2.8 mV[sum= 125 mV] Profiles 1-45 Unsmoothed
LSTd-FAA-DBCO-AF545
m/z [M-H]- calc: 2338.96
2340.47
m/z

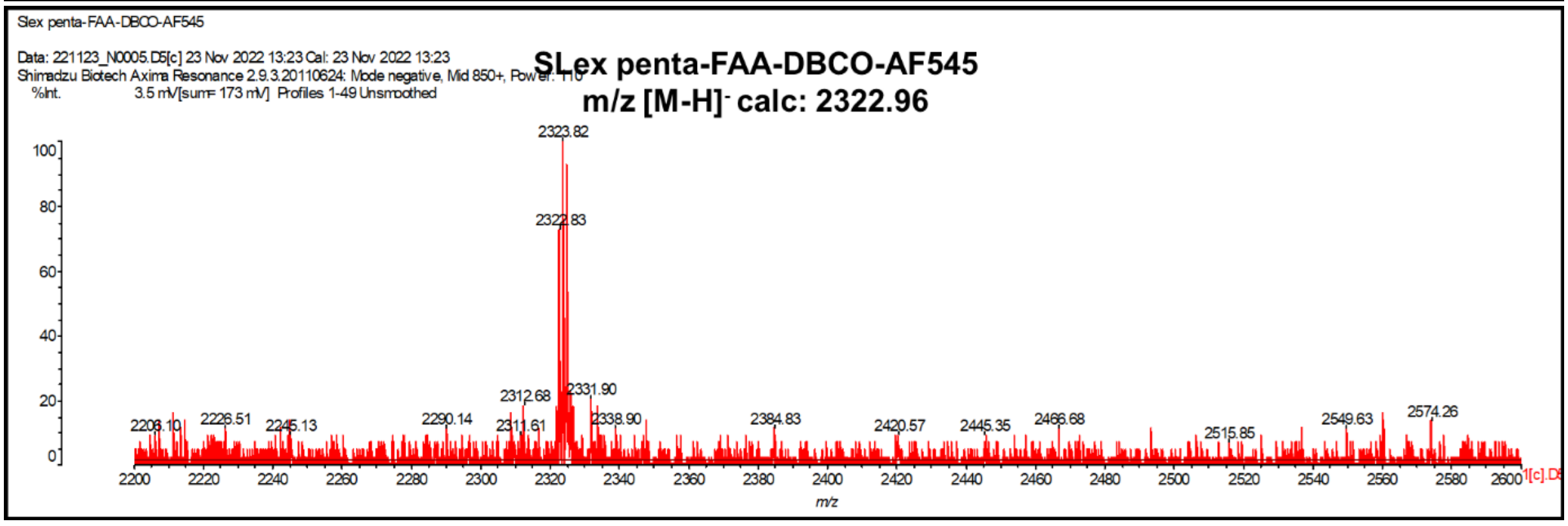
Slex penta-FAA-DBCO-AF545
Data: 221123_N0005.D5[c] 23 Nov 2022 13:23 Cal: 23 Nov 2022 13:23
Shimadzu Biotech Axima Resonance 2.9.3.20110624: Mode negative, Mid 850+, Power: 110
%Int. 3.5 mV[sum= 173 mV] Profiles 1-49 Unsmoothed
SLex penta-FAA-DBCO-AF545
m/z [M-H]- calc: 2322.96
2323.82
m/z

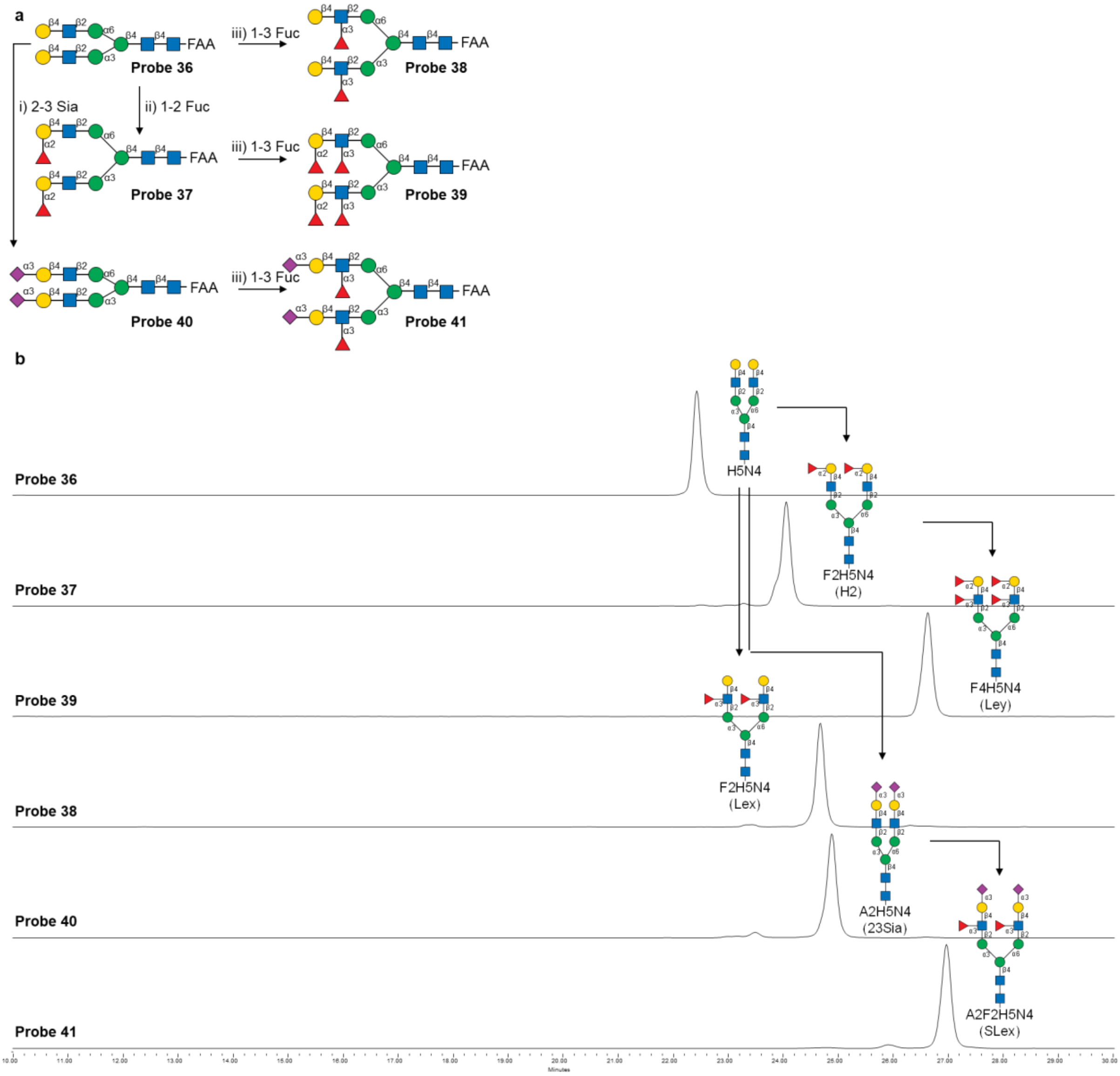

Supplementary Fig. 19. Enzymatic modifications of N glycan probe 36 to generate biantennary *N*-glycan probes 37-41.

i) Sialylation by 2α→3 sialyltransferase from *Pasteurella multocida*. ii) Fucosylation by 1α→2 fucosyltransferase from *Helicobacter hepaticus*. iii) Fucosylation by 1α→3/4 fucosyltransferase from *Helicobacter mustelae*.

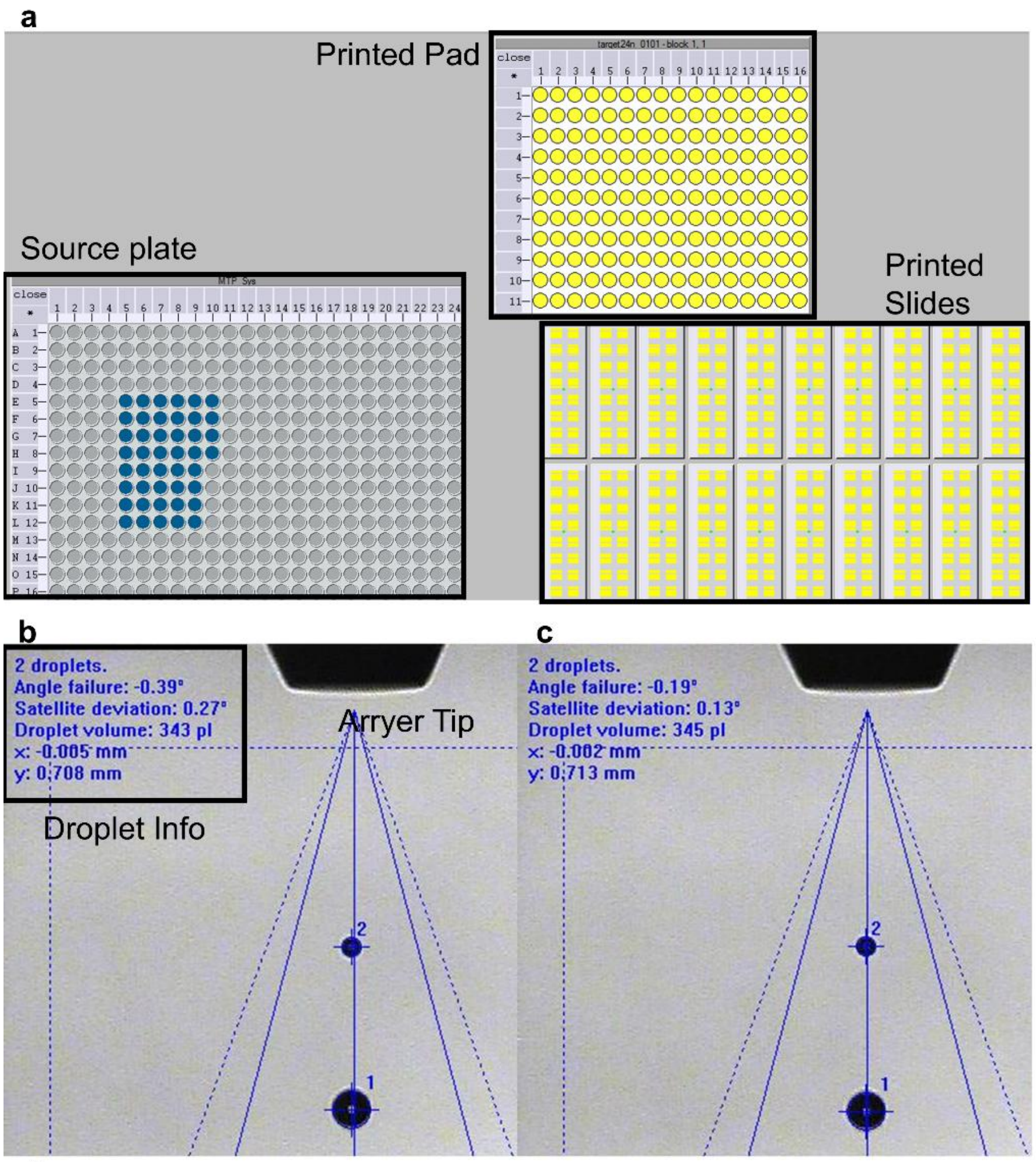

d

/*Pre-spotting: Results and data from strobo-check*/

Tip: 1	Pass: 1	Test: true	Speed: 3.07	Volume: 343	nDrop: 2
Tip: 1	Pass: 2	Test: true	Speed: 3.05	Volume: 345	nDrop: 2
Tip: 1	Pass: 3	Test: true	Speed: 3.05	Volume: 345	nDrop: 2
Tip: 1	Pass: 4	Test: true	Speed: 3.07	Volume: 344	nDrop: 2
Tip: 1	Pass: 5	Test: true	Speed: 3.07	Volume: 344	nDrop: 2

/*Post-spotting: Results and data from strobo-check*/

Tip: 1	Pass: 1	Test: true	Speed: 3.10	Volume: 345	nDrop: 2
Tip: 1	Pass: 2	Test: true	Speed: 3.07	Volume: 349	nDrop: 2

Supplementary Fig. 20. Spotting record of covalent 44 probe FAA glycan array.
a Arrayer software display monitoring the printing status. The yellow circles indicating successfully printed spots. **b** Stroboscope image of the pre-spotting check of the droplets before the printing of probe 24. **c** Stroboscope image of the post-spotting check of the droplets after the printing of probe 24. **d** Log documents the printing process, including 5 pre-spotting checks and 2 post-spotting checks. During these checks, all droplet volumes remained within ±10% of the set value of 340 pL, and the droplet speed stayed within ±10% of the set value of 3.0 m/s. These results indicate that the printing process passed the instrument's self-check program; however, on-slide visualization revealed that only a minimal amount of probe was immobilized.

Supplementary Fig. 21. Array images.

Array layout for covalent arrays

	4 repeats C1-4	4 repeats C5-8	4 repeats C9-12	4 repeats C13-16
**R1**	1	2	3	4
**R2**	5	6	7	8
**R3**	9	10	11	12
**R4**	13	14	15	16
**R5**	17	18	19	20
**R6**	21	22	23	24
**R7**	25	26	27	28
**R8**	29	30	31	32
**R9**	33	34	35	36
**R10**	37	38	39	40
**R11**	41	42	43	44

R = row, C = column

Array layout for noncovalent arrays

	Low / High C1-4	Low / High C5-8	Low / High C9-12	Low / High C13-16
**R1**	1	2	3	4
**R2**	5	6	7	8
**R3**	9	10	11	12
**R4**	13	14	15	16
**R5**	17	18	19	20
**R6**	21	22	23	24
**R7**	25	26	27	28
**R8**	29	30	31	32
**R9**	33	34	35	36
**R10**	37	38	39	40
**R11**	41	42	43	44
**R12**	45	46	47	48
**R13**	49	50	51	52
**R14**	53	54	55	

Each low/high level contain 2 repeats

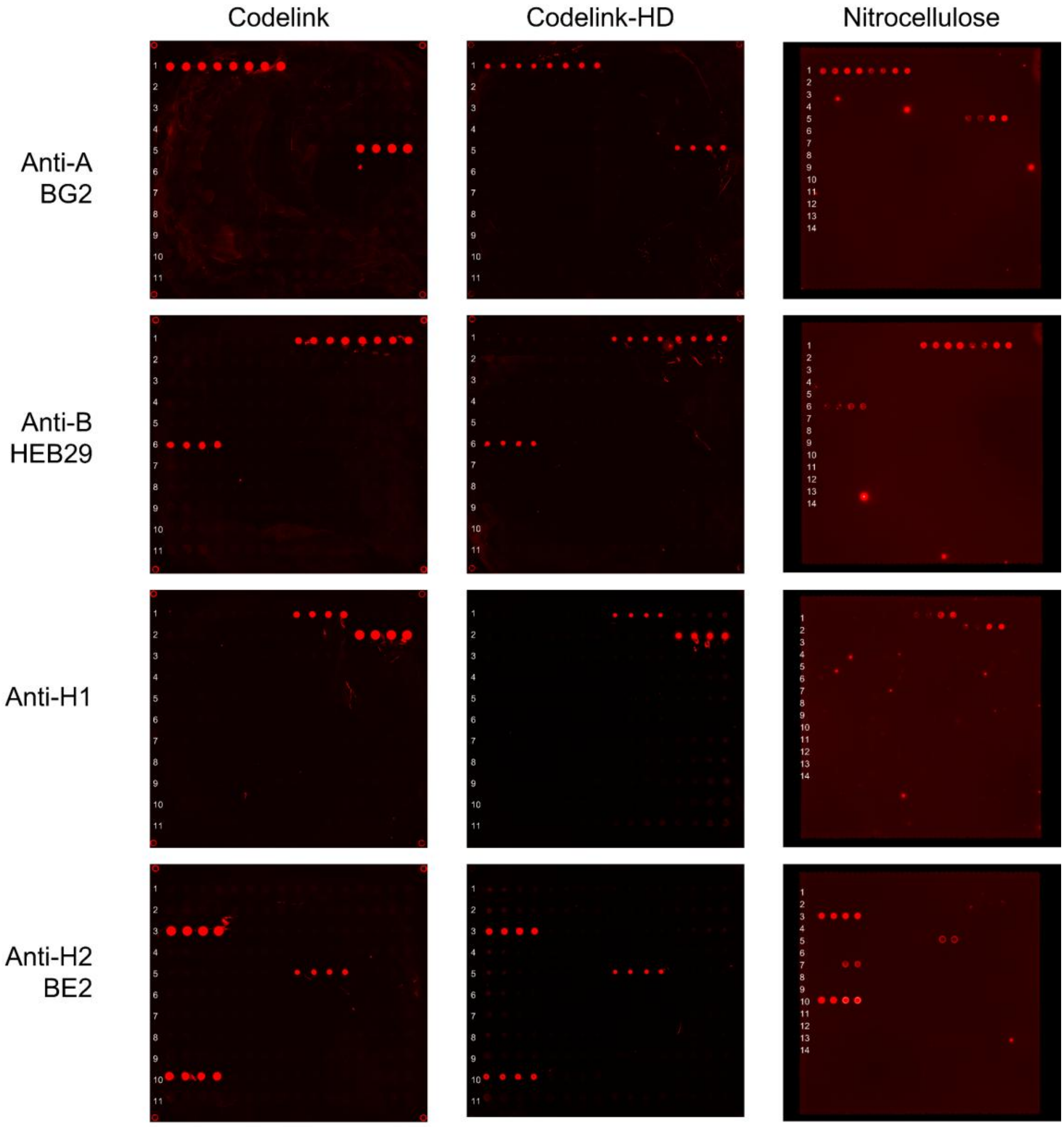

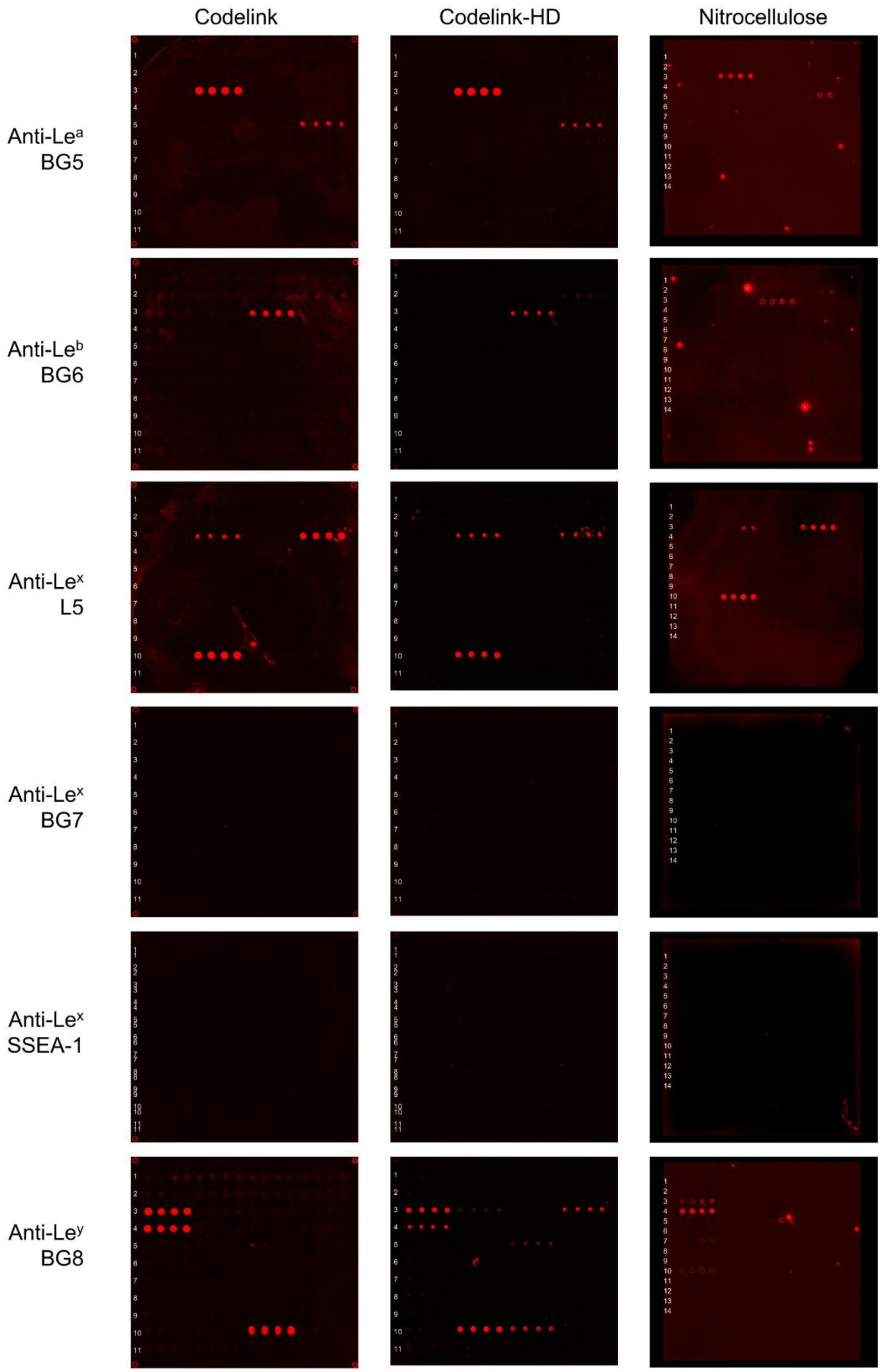
Codelink
Codelink-HD
Nitrocellulose
Anti-Le^{a} BG5
Anti-Le^{b} BG6
Anti-Le^{x} L5
Anti-Le^{x} BG7
Anti-Le^{x} SSEA-1
Anti-Le^{y} BG8

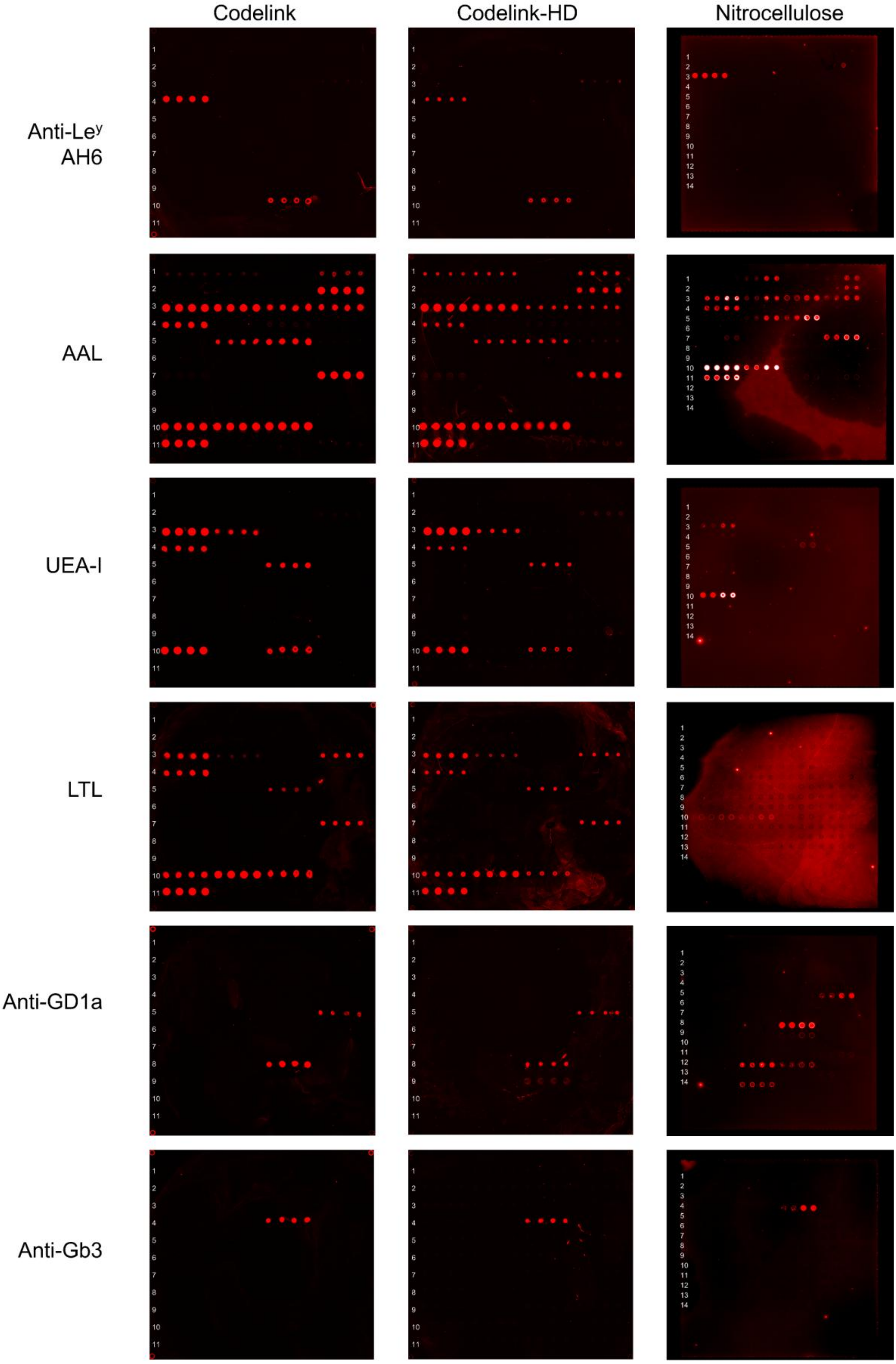
Codelink
Codelink-HD
Nitrocellulose
Anti-Le^y AH6
AAL
UEA-I
LTL
Anti-GD1a
Anti-Gb3

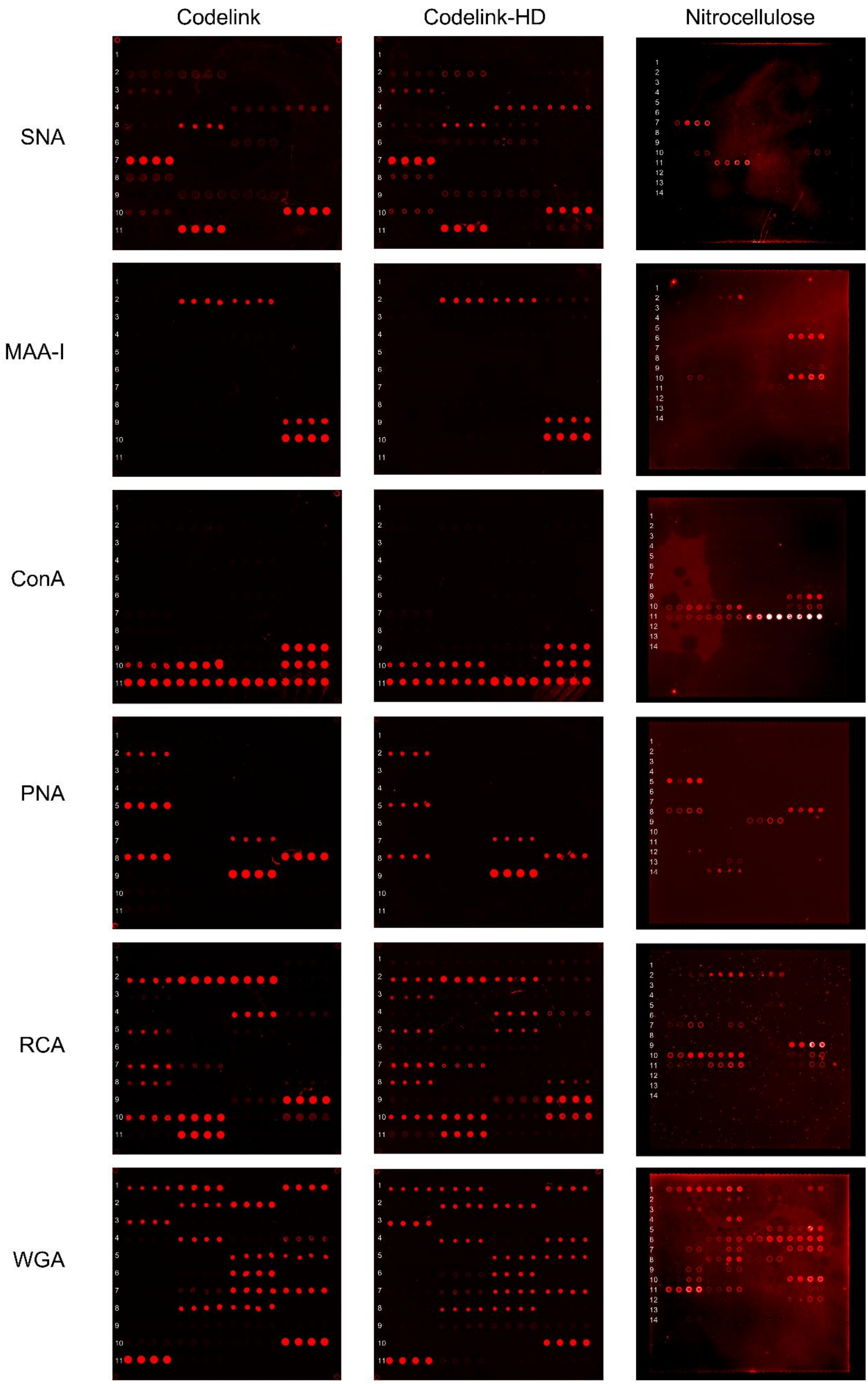
Codelink
Codelink-HD
Nitrocellulose
SNA
MAA-I
ConA
PNA
RCA
WGA

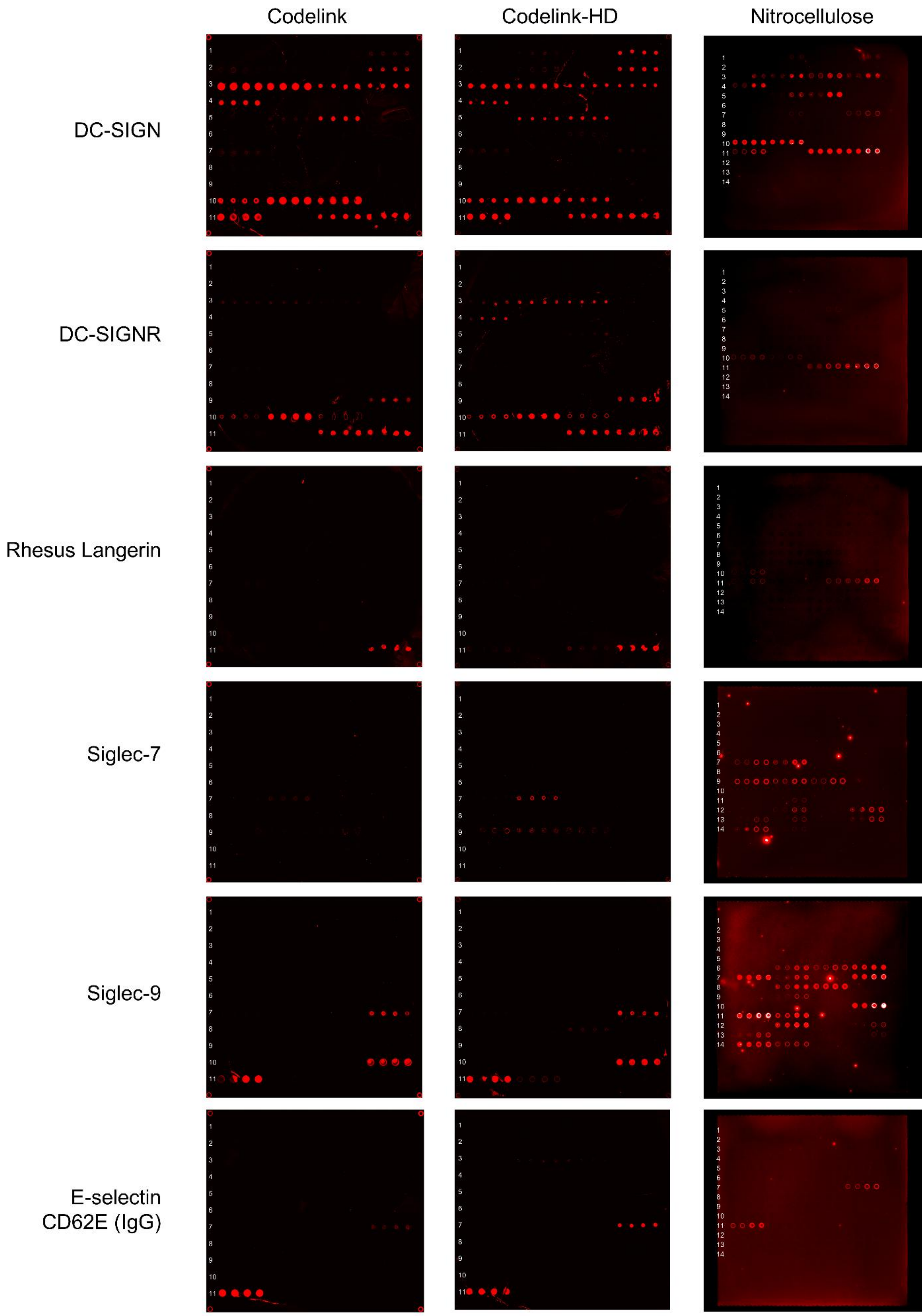
Codelink
Codelink-HD
Nitrocellulose
DC-SIGN
DC-SIGNR
Rhesus Langerin
Siglec-7
Siglec-9
E-selectin CD62E (IgG)

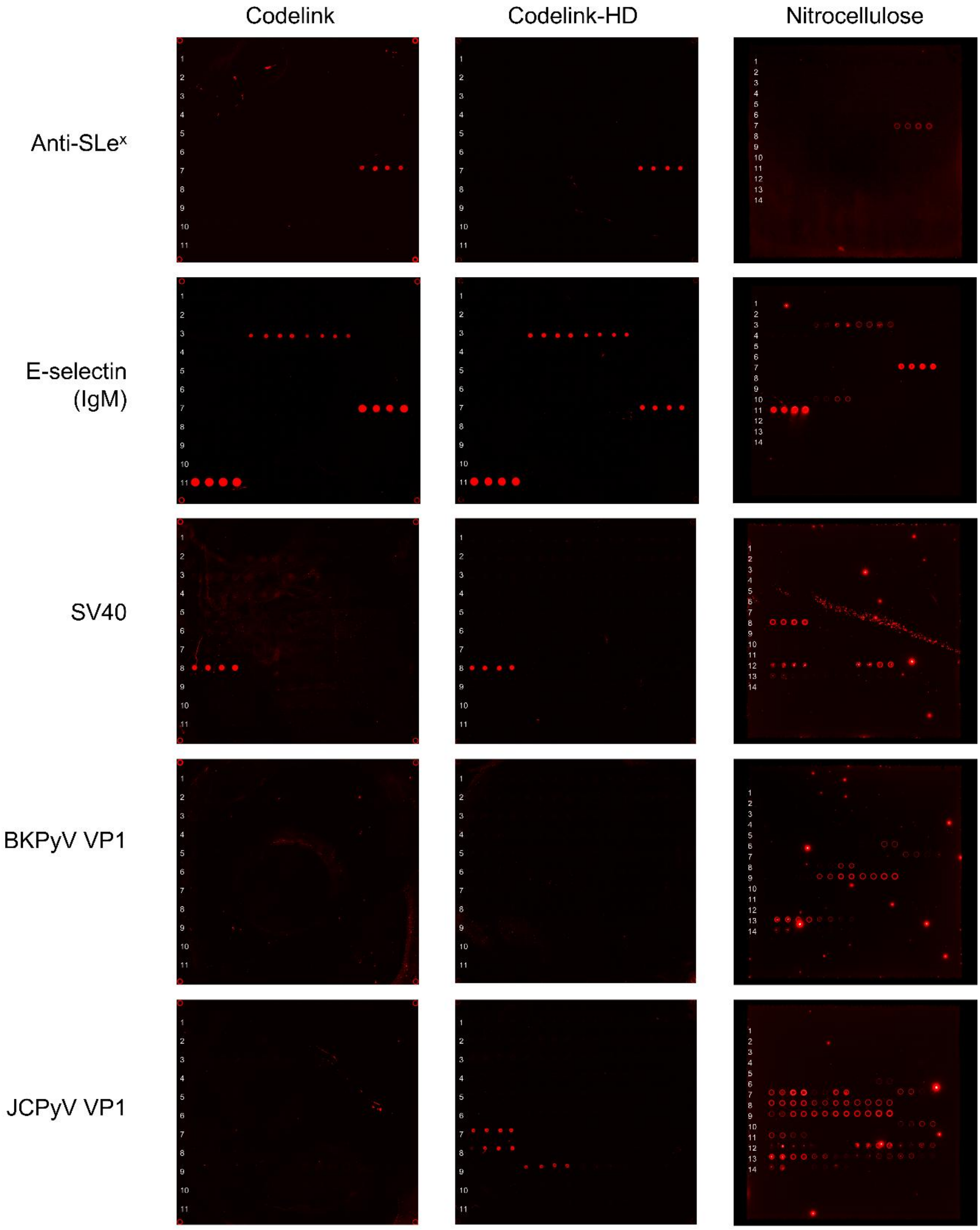
Codelink
Codelink-HD
Nitrocellulose
Anti-SLe^x
E-selectin (IgM)
SV40
BKPyV VP1
JCPyV VP1

	Codelink	Codelink-HD	Nitrocellulose

Classical CTB 5 μg/mL

El Tor CTB 5 μg/mL

LTB 5 μg/mL

Classical CTB 0.5 μg/mL

El Tor CTB 0.5 μg/mL

LTB 0.5 μg/mL

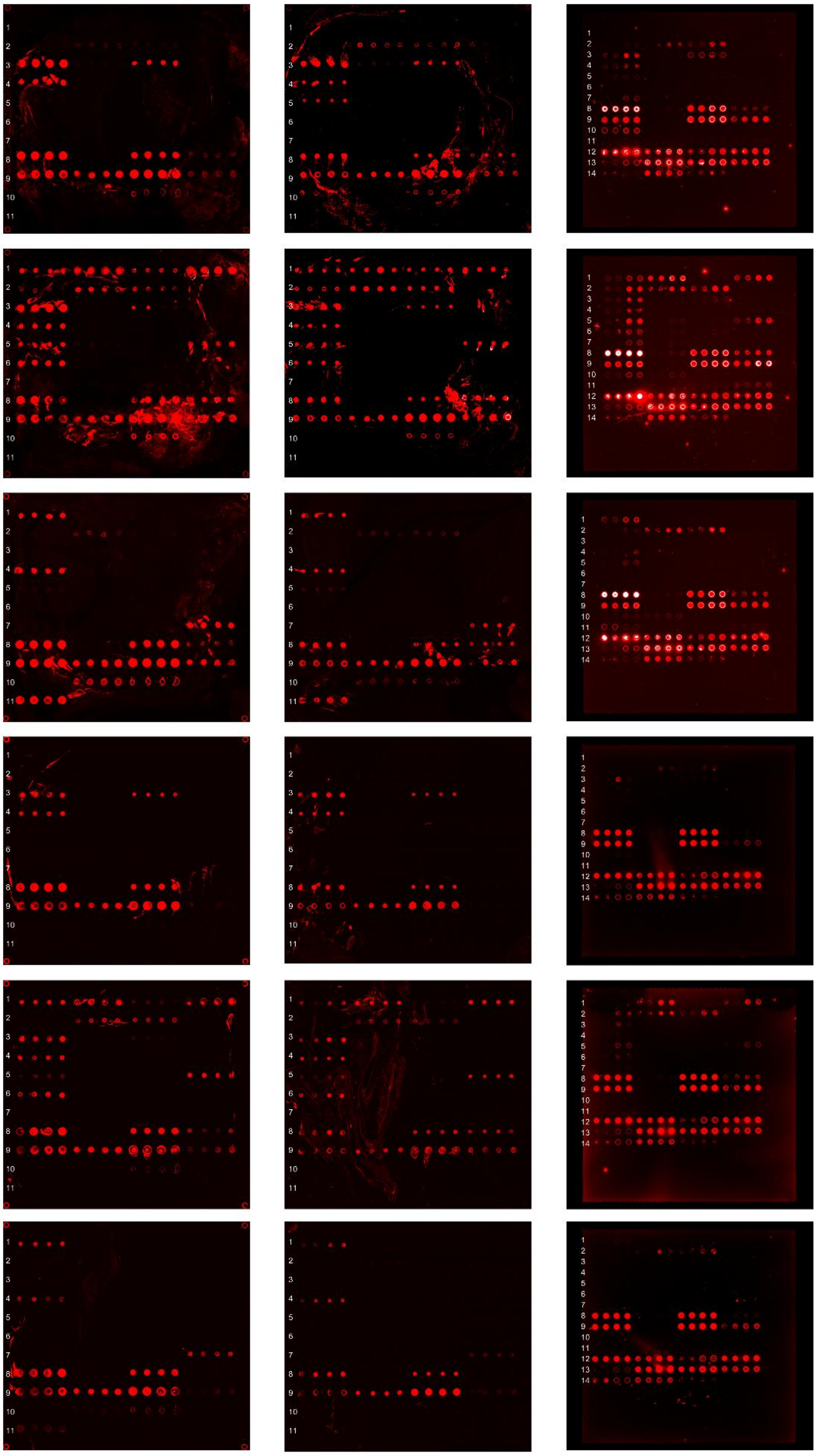

SUPPLEMENTARY TABLES

Supplementary Table 1. Highlights of glycan binding readouts from covalent and noncovalent arrays

	Equivalent in noncovalent and covalent	Subtle but significant differences	Striking difference	
			Preference for the covalent arrays	Preference for the noncovalent array
Plant lectins (9)	AAL, ConA	UEA-I, PNA, RCA120, SNA, MAA-I, WGA	LTL	
	• Lack of binding of AAL to blood group A and B glycans as recently reported[11].<br>• In addition to Le^x and Le^y, LTL also bound to SLe^x and H type 2 (not reported in microarray studies).			
Anti-glycan antibodies (14)	Anti-A (BG2)<br>Anti-B (HEB29)<br>Anti-H1 (17-206)<br>Anti-H2 (BE2)<br>Anti-Le^a (BG5)<br>Anti-Le^b (BG6)<br>Anti-Le^x (anti-L5, BG-7, anti-SSEA-1)<br>Anti-Le^y (AH6)<br>Anti-SLe^x<br>Anti-GD1a<br>Anti-Gb3	Anti-Le^y (BG8)		
	• Anti-H type 1 (clone 17-206) bound weakly to blood group B-type 1 in addition to H-type 1 probes.<br>• Lack of binding anti-H type 2 to globo H analog 2 (not reported).<br>• Anti-Le^x (anti-L5) weakly bound to Le^a probe LNFPII as previously reported[8].<br>• Anti-Le^y BG8 (clone F3) bound to H type 2, and strong binding to Le^x probes observed only on the HD slide.<br>• Lack of binding of anti-Le^x (BG-7 as with anti-SSEA-1) to short chain Le^x and Le^x on biantennary N-glycan.<br>• Lack of binding of anti-SLe^x to SLe^x presented on short outer arms of biantennary N-glycan (reminiscent of the "masking effect"[30]).			
Immune lectins (7)	hE-selectin<br>mE-selectin	hDC-SIGN<br>hDC-SIGNR<br>Rhesus<br>Langerin		hSiglec-7<br>hSiglec-9
	• Binding of DC-SIGNR to Le^x-terminating complex type *N*-glycan (not reported).<br>• Murine E-selectin (IgM construct) but not his-tagged human E-selectin gave weak binding to Le^a, Le^b probes, similar to the binding observed with high-density E-selectin expression on cells[16].			
Viral adhesins (3)	SV40 VP1			JCPyV VP1<br>BKPyV VP1
	• Binding of SV40 VP1 to disialyl glycolipid GalNAc-GD1a (not reported).<br>• JCPyV VP1 binding to several sialyl glycans beyond its known ligand LSTc.			
Bacterial toxins (3)		cCTB<br>El Tor CTB<br>LTBh		
	• The three toxins all bound strongly to a range of gangliosides, including GM1a and GD1b.<br>• Covalent and noncovalent arrays revealed differential binding to low affinity glycan ligands.<br>• The toxins also have differing preference for blood-group probes:<br>cCTB: A type 1, Le^y, SLe^x;<br>El Tor CTB: H type 2, Le^y, Le^b;<br>LTBh: A and B, H type 2, Le^y.			

Supplementary Table 2. Designations of sequence-defined glycans.
Their sequences are in **Supplementary Data 1** and **2**. The definition of the color pattern was given in **Figure 2** in the main text.

No*	Short name	Full name of reducing glycans used for FAA probe preparation	Vendor	Code
1	A-penta-T1	Blood group A antigen pentaose type 1	Elicityl	GLY036-1
2	A-hexa-T2	Blood group A antigen hexaose type 2	Elicityl	GLY037-2
3	B-hexa-T1	Blood group B antigen hexaose type 1	Elicityl	GLY040-1
4	B-hexa-T2	Blood group B antigen hexaose type 2	Elicityl	GLY040-2
5	LNT	Lacto-N-tetraose	Dextra	L403
6	LNnT	Lacto-N-neotetraose (LNnT / neo-LNT)	Dextra	GLY021
7	LN3	Tri-N-Acetyl-D-Lactosamine	Elicityl	GLY007-2
8	LNFPI	Lacto-N-fucopentaose I	Dextra	L502
9	LNnFPI	Blood group H antigen pentaose type 2 / Lacto-N-neofucopentaose I (LnNFP I)	Elicityl	GLY033-2
10	LNFPII	Lacto-N-fucopentaose II	Dextra	L503
11	Le^b	Lewis b (Le^b) pentaose	Elicityl	GLY056
12	Le^x	Lewis x (Le^x) tetraose	Elicityl	GLY050
13	Le^y	Lewis y (Le^y) pentaose	Elicityl	GLY052
14	FA-penta	Forssman antigen pentaose	Elicityl	GLY132
15	Gb3	Globotriaose (Gb3) / Pk antigen	Elicityl	GLY120
16	Gb4	Globotetraose (Gb4) / P antigen	Elicityl	GLY121
17	Gb5	Globopentaose/Stage Specific Embryonic Antigen 3a (SSEA-3a)	Elicityl	GLY122
18	Globo-H	Globo-H hexaose /Stage Specific Embryonic Antigen 3b (SSEA-3b)	Elicityl	GLY123
19	Globo-H-T2	Globo-H analogue type 2	Elicityl	GLY194-2
20	Globo-A	Globo-A heptaose	Elicityl	GLY124
21	Globo-B	Globo-B heptaose	Elicityl	GLY125
22	3'-SL	3'-Sialyllactose	Dextra	SL302
23	LST a	LS-Tetrasacchride a	Dextra	SLN503
24	LSTd	LS-Tetrasaccharide d/ Sialyl-lacto-N-neotetraose d (Sodium salt)	Elicityl	GLY083
25	LST c	LS-Tetrasacchride c	Dextra	SLN506
26	LSTb	LS-Tetrasaccharide b	Dextra	SLN516
27	SSEA4-hexa	SSEA-4 hexaose/ Stage-Specific Embryonic Antigen-4 hexaose	Elicityl	GLY131
28	SLe^x Penta	Sialyl Lewis x (sLe x) pentaose - Sodium salt	Elicityl	GLY053
29	GM1a	GM1a Ganglioside oligosaccharide	Elicityl	GLY096
30	GM1b	GM1b Ganglioside oligosaccharide)	Elicityl	GLY097
31	GD1a	GD1a Ganglioside oligosaccharide)	Elicityl	GLY098
32	GalGalNAc asialo GM1	GalGalNAc-aGM1 (GalGalNAc asialo GM1) Ganglioside oligosaccharide analogue	Elicityl	GLY174
33	GT1a	GT1a Ganglioside oligosaccharide)	Elicityl	GLY100
34	GD2	GD2 Ganglioside oligosaccharide)	Elicityl	GLY094
35	GD1b	GD1b Ganglioside oligosaccharide)	Elicityl	GLY099
36	H5N4	Asialo, galactosylated, biantennary (NA2)	Dextra	C0920
37	F2H5N4(H2)	FAA-tagged, enzymatically modified from probe 36		
38	F2H5N4(Le^x)	FAA-tagged, enzymatically modified from probe 36		
39	F2H5N4(Le^y)	FAA-tagged, enzymatically modified from probe 36		
40	A2H5N4(2-3Sia)	FAA-tagged, enzymatically modified from probe 36		
41	A2F2H5N4(SLe^x)	FAA-tagged, enzymatically modified from probe 36		
42	A2H5N4(2-6Sia)	Disialo (2,6), biantennary (A2)	Dextra	SC1120
43	M5N2	Oligomannose-5 glycan	Dextra	MC0731
44	M9N2	Oligomannose-9 glycan	Dextra	MC1131
**	Glc2	Cellobiose: Glcβ1→4Glc	Dextra	BC121
**	Mal3	Maltotriose: Glc(α1→4Glc)$_2$	Dextra	G302
**	Mal5	Maltopentaose: Glc(α1→4Glc)$_4$	Dextra	G502
**	Mal7	Maltoheptaose: Glc(α1→4Glc)$_6$	Dextra	G702

* Glycan numbers refer to positions in sequence-defined array.
** Additional glycans were used as reference FAA-tagged compounds.

Supplementary Table 3. Anti-carbohydrate antibodies (in total 14).

Antibody	Other designation	Clone	Vendor or collaborator	Catalogue #	Isotype	Dilution used
Anti-Le^a	BG5	T174	Biolegend	922202	Mouse IgG	1:50
Anti-Le^b	BG6	T218	Biolegend	922302	Mouse IgM	1:50
Anti-Le^x	Anti-L5*		Andrea Streit		Rat IgM	1:50
Anti-Le^x	BG7	P12	Biolegend	912901	Mouse IgM	1:50
Anti-Le^x	Anti-SSEA1	MC-480	DHSB	MC-480-c	Mouse IgM	1:50
Anti-Le^y	BG8	F3	Biolegend	912501	Mouse IgM	1:50
Anti-Le^y	AH6**	AH6	Henrik Clausen		Mouse IgM	1:5
Anti-A	BG2	T36	Biolegend	921902	Mouse IgG	1:10
Anti-B	HEB29	HEB-29	Abcam	Ab2524	Mouse IgM	1:10
Anti-H type 1	H(O) type 1	17-206	Invitrogen	14-9810-82	Mouse IgG	1:50
Anti-H type 2	BE2**	BE2	Henrik Clausen		Mouse IgM	1:2
Anti-SLe^x	CD15S	CSLEX1	BD Pharmingen	551344	Mouse IgM	1:50
Anti-GD1a		GD1a-1	Millipore	MAB5606	Mouse IgG	1:250
Anti-Gb3		BGR23	TCI	A2506	Mouse IgG	1:140

* Rat anti-L5 purified from culture supernatant was a gift from Dr Andrea Streit (King's College London)[25].
** Anti-H type 2 (BE2)[9] and Anti-Le^y (AH6)[26] was kindly provided by Henrik Clausen Lab (Copenhagen Center for Glycomics).

Supplementary Table 4. Detection antibodies.

	Vendor	Catalogue #	Stock conc.	Dilution used
Anti-Mouse IgG (biotinylated)	Merck	B7264	0.4 mg/mL	1:200
Anti-Mouse IgM (biotinylated)	Vector	BA-2020	0.5 mg/mL	1:200
Anti-Rat IgM (biotinylated)	Rockland	612-4607	1.0 mg/mL	1:200
Anti-Rabbit IgG (biotinylated)	Merck	B7389	0.6 mg/mL	1:200
Anti-Human IgG (biotinylated)	Vector	BA-3000	1.5 mg/mL	1:200
Anti-Human IgM (biotinylated)	Vector	BA-3020	0.5 mg/mL	1:200
Anti-His (Mouse IgG isoform)	Merck	SAB4200620	1.0 mg/mL	1:100
Anti-Cholera toxin (Rabbit IgG)	Merck	C3062	1.0 mg/mL	1:200

Supplementary Table 5. Biotinylated plant lectins (in total 10).

	Vendor	Cat	Stock conc.	Used conc.
Aleuria Aurantia Lectin (AAL)	Vector Laboratories	B-1395	2 mg/mL	20 µg/mL
Ulex Europaeus Agglutinin I (UEA-I)	Vector Laboratories	B-1065	2 mg/mL	50 µg/mL
Lotus Tetragonolobus Lectin (LTL)	Vector Laboratories	B-1325	2 mg/mL	100 µg/mL
Concanavalin A (ConA)	Vector Laboratories	B-1005	5 mg/mL	5 µg/mL
Sambucus Nigra Lectin (SNA)	Vector Laboratories	B-1305	2 mg/mL	50 µg/mL
Maackia Amurensis Lectin I (MAA-I)	Vector Laboratories	B-1315	2 mg/mL	100 µg/mL
Maackia Amurensis Lectin II (MAA-II)	Vector Laboratories	B-1265	1 mg/mL	100 µg/mL
Peanut Agglutinin (PNA)	Vector Laboratories	B-1075	5 mg/mL	50 µg/mL
Ricinus Communis Agglutinin I (RCA 120)	Vector Laboratories	B-1085	5 mg/mL	5 µg/mL
Wheat Germ Agglutinin (WGA)	Vector Laboratories	B-1025	5 mg/mL	20 µg/mL

Supplementary Table 6. Glycan binding receptors of the immune system (in total 7).

	Source	Tag	Incubation conditions
Human DC-SIGN	Sino Biological, 10200-H01H	hFc-tagged	5 µg/mL; pre-complexed with biotinylated anti-human IgG (1:2 by weight)
Human DC-SIGNR	Sino Biological, 10559-H01H	hFc-tagged	20 µg/mL; pre-complexed with biotinylated anti-human IgG (1:2 by weight)
Rhesus Langerin	Sino Biological, 90159-C01H	hFc-tagged	20 µg/mL; pre-complexed with biotinylated anti-human IgG (1:2 by weight)
Human Siglec-7	R&Dsystems, 1138-SL-050	hFc-tagged	5 µg/mL; pre-complexed with biotinylated anti-human IgG (1:1 by weight)
Human Siglec-9	R&Dsystems, 1139-SL-050	hFc-tagged	3 µg/mL; pre-complexed with biotinylated anti-human IgG (1:1 by weight)
Human E-Selectin (CD62E)	SinoBiological, 10335-h08h	His-tagged	20 µg/mL; pre-complexed protein:anti-His: biotinylated anti-mIgG (8:6:3 by weight)
Murine E-Selectin IgM*	John B. Lowe, University of Michigan Medical School[27]	Human IgM	1:4 dilution followed by detection with biotinylated anti-human IgM

* E-Selectin was kindly provided by John B. Lowe, University of Michigan Medical School.

Supplementary Table 7. Microbial glycan binding proteins (in total 6).

	Source	Tag	Incubation conditions
Wild type (WT) BKPyV VP1	Thilo Stehle laboratory (University of Tübingen)	His-tagged	150 µg/mL; pre-complexed protein:anti-His: biotinylated anti-mouse IgG (4:2:1 by weight)
SV40 VP1			
JCPyV VP1 (Mad-1)			
Classical *Vibrio cholerae* Toxin Subunit B (cCTB)	Merck, c9903	N/A	5 µg/mL and 0.5 µg/mL
El Tor Cholera Toxin Subunit B (El Tor CTB)[28, 29]	Prepared by Turnbull Lab (for details please see the Supporting Information of Mandal et al.[18]).		
Escherichia Coli heat-labile Toxin (LTBh)			

Supplementary Table 8. MIRAGE compliant Glycan Microarray Document
Supplementary Glycan Microarray Document Based on MIRAGE Guidelines (doi:10.3762/mirage.3)

	Description
1. Sample: Glycan Binding Sample	
Description of Sample	The anti-carbohydrate antibodies, biotinylated plant lectins, immune lectin receptors, viral VP1 proteins, and bacterial toxins used in this study, in total 36, are detailed in **Supplementary Table 3-7**.
Sample modifications	Not relevant.
Assay protocol	Microarray binding analyses were performed at R.T. essentially as described[1, 5, 6]. In brief, the NC slides were treated with blocking buffers for 1 h prior to incubations, and the covalent slides were used without any prior slide blocking step. All overlay steps of the VP1 virus proteins were performed in blocking buffer A containing 0.3%BSA, 0.33% Casein in HEPES-Buffered Saline (HBS) (5 mM HEPES buffer pH 7.4, 150 mM NaCl, 5mM $CaCl_2$), whereas the incubations of plant lectins and anti-carbohydrate antibodies were performed in blocking buffer B containing 1%BSA, 10 mM $CaCl_2$ in HBS. The concentration of the antibodies and lectins are indicated in **Supplementary Table 3-7**. Stepwise incubations were performed for 1 h per overlay step, except for the final detection step with Alexa Fluor-647-labeled streptavidin (Molecular Probes), which was 30min. Incubation of His-tagged VP1 virus proteins, which were performed with a single pre-complexed condition using VP1 protein:anti-His:biotin-anti-mIgG 4:2:1 (by weight) for 1.5 h.<br><br>For on-array visualization of printed covalent probes, the slide was incubated with a solution of DBCO-PEG_4-Biotin in 1:1 DMF:H_2O (100 µM) at R.T. overnight (100 ul per sub-array). The final detection step was carried out by 30 min overlay with 1 µg/mL Alexa Fluor-647-labeled streptavidin (Molecular Probes, Eugene).
2. Glycan Library	
Glycan description for defined glycans	The sequence information on the 44 FAA-glycan probes and 55 lipid-linked glycan probes (44 FAA-NGL probes and 11 natural glycolipid controls) included in the covalent and noncovalent microarrays are in **Fig. 2**, **Supplementary Data 1** and **2**, respectively.
Glycan description for undefined glycans	Not relevant.
Glycan modifications	The synthesis of covalent and NGL probes were described above under Supplementary Methods.
3. Printing Surface; e.g., Microarray Slide	
Description of surface	For covalent printing, NHS-coated TRIDIA (Codelink) and TRIDIA-High Density (Codelink-HD) glass microarray slides from Surmodics (Minnesota, USA) were used.<br><br>For noncovalent array, UniSart® Microarray slide with 16 nitrocellulose membrane pads from Sartorius (Stedim, Goettingen, Germany) were used.
Manufacturer	Indicated above.
Custom preparation of surface	Not relevant.

Noncovalent Immobilization	The lipid-linked oligosaccharide probes were formulated as liposomes by adding carrier lipids, 1,2-dihexanoyl-sn-glycero-3-phosphocholine (DHPC) and cholesterol (both from Merck) for arraying and non-covalent immobilization on nitrocellulose-coated glass slides essentially as described[1].
Covalent Immobilization	The free amine groups of the FAA tagged glycans were covalently attached to the NHS-coated glass slides via amide coupling.<br><br>The printing condition and post-printing slide treatment are described under Section 4 below. Upon completion of the printing process, the slides were incubated overnight in a humidity tray at R.T. and 60% humidity. The slides were immersed for 1 hour in a 100 mM Sodium Borate buffer (pH 8.5) containing 50 mM Ethanolamine. Subsequently, the slides are washed four times with a PBS and Tween 20% solution, followed by a rinse with Nanopure water. The slides were then dried and stored vacuum-sealed bags at -20°C.
4. Arrayer (Printer)	
Description of Arrayer	The printing of the microarrays was performed with Nano-Plotter from Gesim, Germany.
Dispensing mechanism	Non-contact liquid delivery with four dispensing tips.
Glycan deposition	For covalent printing, triplicate droplets (approximate 0.33 nL x3) were dispensed for each spot.<br><br>For noncovalent printing, single droplet (approximate 0.33 nL) was dispensed for each spot.
Printing conditions	For covalent microarray printing, a 100 μM printing solution prepared in 100 mM phosphate buffer (pH 8.5) was used, unless otherwise specified. Arrays were printed over a 6-hour period at R.T. under 58% relative humidity and subsequently left overnight in the printer chamber (R.T. and ~60% humidity).<br><br>For noncovalent arrays, the 'liposome' printing solutions (aqueous based) contained 100 pmol/μL of DHPC and cholesterol as lipid carriers in addition to the lipid-linked glycan probes. The concentrations of the lipid-linked glycan probes were 5 and 15 pmol/μL for the 2 and 5 fmol per spot levels, respectively. The printing solutions also contained Cy3 NHS ester (GE Healthcare) at 20 ng/ml as a marker to monitor the printing process. The resulting arrays were printed at R.T. under 58% relative humidity.
5. Glycan Microarray with "Map"	
Array layout	For the covalent arrays, each slide contained 16 pads arranged in an 8 × 2 format. Each subarray comprised 44 glycans, which were spotted in quadruplicate within a single row, with a total of 11 rows printed per subarray.<br><br>For the noncovalent arrays, each slide also contained 16 pads in an 8 × 2 format. Each subarray included 44 NGLs and 11 glycosylceramides, each printed at two concentrations (2 and 5 fmol per spot) in duplicate (resulting in four spots per glycan in a row). A total of 14 rows were printed per subarray.<br><br>The array images are in **Supplementary Fig. 21**.
Glycan identification and quality control	The details of glycan sequences are included in **Supplementary Data 1** and **2**. Quality control with sequence-specific lectins and anti-carbohydrate antibodies were listed in **Supplementary Data 1**.

6. Detector and Data Processing	
Scanning hardware	GenePix 4300A (Molecular Devices, Berkshire, UK)
Scanner settings	The scanning of the microarray slides after incubation and drying was carried out at a resolution of 5 μm/pixel, using the red laser channel (scan wavelength 635 nm).<br>For noncovalent NC slides PMT Voltages were set at 350V and the scan power ranging from 30% to 100% was used to avoid signal saturation. For covalent slides, PMT Voltages were set at 400V and the scan power at 100% was used.
Image analysis software	GenePix® Pro 7 (Molecular Devices, Berkshire, UK)
Data processing	The gpr files were processed in pre-set Excel templates.<br>The software reported the background-subtracted mean fluorescence intensity and the number of pixels for each spot. Total fluorescence per spot was calculated as the product of these values and subsequently normalised by division by 1000 to bring the values into a range comparable to the mean fluorescence intensity. Total fluorescence was used in preference to mean intensity as it accounts for variation in spot size, in particular for the covalent arrays, and therefore better reflects the overall signal per spot.
**7. Glycan Microarray Data Presentation**	
Data presentation	Binding results are presented as histogram charts in the main text **Fig. 4-6** and additional figures in **Supplementary Fig. 3-7.**<br>Binding scores and relative intensities of binding (matrices) are shown in **Supplementary Data 3** and **4**.
**8. Interpretation and Conclusion from Microarray Data**	
Data interpretation	No software or algorithms were used to interpret processed data.
Conclusions	The conclusion of the findings in different FAA arrays with different GPBs were summarized in **Supplementary Table 1**.

SUPPLEMENTARY METHODS

General methods

Commercial chemicals and solvents were of the highest grade available. All chemicals were from Merck (Dorset, UK) unless otherwise stated. Thin layer chromatography (TLC) was performed on Silica Gel F254 (Merck, Dorset, UK) with detection by UV-light. High performance TLC (HPTLC) for glycolipids (GLs) and neoglycolipids (NGLs) was performed on Silica Gel plates (Merck, Dorset, UK). For HPTLC analyses, glycans, GLs and NGLs were applied onto the silica gel plates with a TLC applicator (Camag Linomat 5, Muttenz, Switzerland) and the TLC tank was equilibrated with the developing solvents (as indicated) for 30 min prior to chromatography. Lipids were detected by UV-light after staining with primulin solution[1] and glycans were visualized by staining with orcinol solution[1]. The quantitation based on readouts from the chromatogram plates was performed using a Camag TLC Scanner 3. Silica and C18 cartridges used for the purification of glycans were purchased from WATERS (Manchester, UK). Cellulose powder (10-100 µm particle size) used for column chromatography was from Merck. Flash column chromatography was performed on silica gel 60 Å (35-70 mm, Acros, Geel, Belgium). ^{1}H-NMR, as well as 2D HSQC and HMBC NMR spectra were recorded at 303 K with a Bruker Avance III HD 500 MHz NMR spectrometer using $CDCl_3$ (internal $CDCl_3$, δH 7.26; δC 77.16 ppm at 303 K) or Methanol-d4 (internal MeOD-D_4, δH 4.87; δC 49.13 ppm at 303 K). HPLC purification of derivatized glycans was performed on a WATERS E2695 system with 2489 UV and 2475 fluorescence detectors. For normal phase (NP) HPLC, an Amide HPLC column (3.5 µm, 4.6 mm x 250 mm, Xbridge, WATERS) was used. High resolution MS spectra (HR-ESI-MS) were recorded with a Waters Synapt G2-S mass spectrometer. HPLC fractions were analysed on a Shimadzu Axima Resonance MALDI QIT-TOF mass spectrometer (Milton Keynes, Buckinghamshire, UK).

Abbreviations of chemicals: Hexafluorophosphate azabenzotriazole tetramethyl uronium (HATU), 1-Hydroxy-7-azabenzotriazole (HOAt), Dimethylformamide (DMF), N,N-Diisopropylethylamine (DIPEA), Dibenzocyclooctyne -N-hydroxysuccinimidyl ester (DBCO-NHS), 1,2-Dihexadecyl-*sn*-glycero-3-phosphoethanolamine (DHPE) and Dibenzocyclooctyne-PEG4-Fluor 545 (DBCO-PEG4-Flour545), Dibenzocyclooctyne-PEG4-biotin conjugate (DBCO-PEG4-Biotin).

Synthesis of the FAA linker

(9*H*-fluoren-9-yl)methyl (*S*)-(5-azido-1-((2-((*tert*-butoxycarbonyl) amino)ethyl)amino)-1-oxopentan-2-yl) carbamate (Compound **1**)

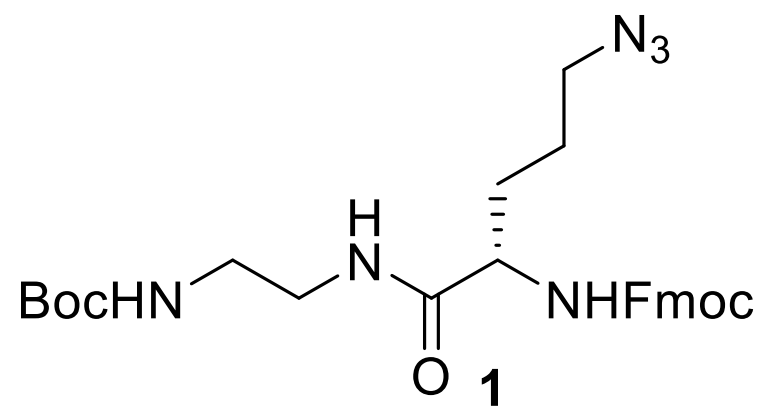

1

To a solution of Fmoc-L-azidoornithine (3.0 g, 7.9 µmol, 1 eq) with HATU (3.0 g, 7.9 mmol, 1 eq) and HOAt (1.07 g, 7.9 mmol, 1 eq) in DMF (20 mL), DIPEA (2.47 mL, 15.8 mmol, 2 eq) was added. The activated amino acid mixture was then slowly added to a solution of N-Boc-ethylenediamine (1.87 mL, 11.8 mmol, 1.5 eq) in DMF (2 mL). After incubating at room temperature (R.T.) for 2 h, TLC indicated a full conversion of the starting material Fmoc-L-azidoornithine into a single product. The reaction mixture was then diluted with 50 mL $CHCl_3$ and extracted three times with 50 mL H_2O. The residue was concentrated under reduced pressure and purified by flash column chromatography ($CHCl_3$:MeOH 99:1 to 80:20) to give compound **1** in 81% yield (3.34 g, 6.4 umol). R_f = 0.4 (cyclohexane:ethyl acetate:acetic acid 3:2:0.01). The MS and NMR spectra are in **Supplementary Fig. 10** and **Supplementary Fig. 12**. ***HR-ESI-MS***, *m/z*: 545.2593 ([M+Na]$^+$, calc. 545.2483). ***^{1}H-NMR*** (500 MHz, Chloroform-*d*) δ = 7.75 (d, *J*=7.6, 2H, H-4-; H-5-Fmoc), 7.57 (dd, *J*=7.5, 2.7, 2H, H-1-; H-8-Fmoc), 7.38 (td, *J*=7.5, 3.2, 2H, H-2-; H-7-Fmoc), 7.32 – 7.26 (m, 2H, H-3-; H-6-Fmoc), 7.05 (s, 1H, $(CH_2)_2N\mathit{H}CO$), 5.77 (d, *J*=8.1, 1H, Fmoc-N*H*), 5.11 (t, *J*=6.3, 1H, Boc-N*H*), 4.43 (dd, *J*=10.6, 7.1, 1H, Fmoc CH_2), 4.33 (dd, *J*=10.6, 7.0, 1H, Fmoc CH_2), 4.19 (m, 2H, C*H*α, H-9-Fmoc), 3.38 – 3.05 (m, 6H, $BocNHCH_2C\mathit{H}_2$, CH_2δ, $BocNHC\mathit{H}_2CH_2$), 1.90 (p, *J*=6.2, 1H, CH_2β), 1.70 (dt, *J*=14.3, 7.4, 1H, CH_2β), 1.61 (p, *J*=7.2, 2H, CH_2γ), 1.41 (s, 9H,C(CH_3)$_3$). ***^{13}C-NMR*** (126 MHz, $CDCl_3$) δ = 172.02 (*C*=OCHα), 156.97 (Boc *C*=O), 156.31(Fmoc *C*=O), 143.84, 143.78 (C-1$_a$-; C-8$_a$-Fmoc), 141.39 (C-4$_a$-; C-5$_a$-Fmoc), 127.84 (C-2-; C-7-Fmoc), 127.16 (C-3-; C-6-Fmoc), 125.18, 125.11 (C-1-; C-8-Fmoc), 120.10, 120.08 (C-4-; C-5-Fmoc), 79.85 (*C*(CH_3)$_3$), 67.10 (Fmoc *C*H_2), 54.49 (*C*Hα), 51.03 (*C*H_2δ), 47.21 (C-9-Fmoc), 40.78 ($BocNHCH_2\mathit{C}H_2$), 40.16 ($BocNH\mathit{C}H_2CH_2$), 30.20 (*C*H_2β), 28.43(C(*C*H_3)$_3$), 24.95 (*C*H_2γ).

(9*H*-fluoren-9-yl)methyl (*S*)-(1-((2-aminoethyl)amino)-5-azido-1-oxopentan-2-yl)carbamate (Compound **2, FAA linker)**

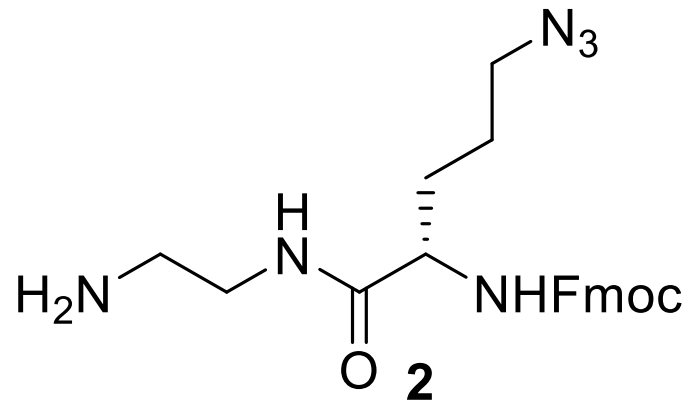

2

To an Eppendorf tube containing compound **1** (20 mg, 38.3 µmol), 200 µL 2 M HCl in MeOH was added and the vial was vortexed vigorously at R.T. for 2 h until full solubilisation of the starting material. The reaction was monitored to completion by TLC; and the solvent was removed to semi-dryness under reduced pressure, diluted with H_2O and freeze-dried twice to give compound **2** (FAA linker) as a white powder in quantitative yield. R_f = 0.25 (ethyl acetate:acetic acid:MeOH:H_2O 60:3:3:2). ***HR-ESI-MS***, *m/z*: 423.2335 ($[M+H]^+$, calc. 423.2139). The MS and NMR spectra are in **Supplementary Fig. 11** and **Supplementary Fig. 13**. ***^{1}H-NMR*** (500 MHz, Methanol-d_4) δ = 7.70 (d, *J*=7.5, 2H, H-4-; H-5-Fmoc), 7.58 (d, *J*=7.4, 2H, H-1-; H-8-Fmoc), 7.30 (t, *J*=7.4, 2H, H-2-; H-7-Fmoc), 7.22 (td, *J*=7.5, 1.1, 2H, H-3-; H-6-Fmoc), 4.39 (dd, *J*=10.6, 6.7, 1H, Fmoc C*H*$_2$), 4.27 (dd, *J*=10.7, 6.6, 1H, Fmoc C*H*$_2$), 4.13 (t, *J*=6.5, 1H, H-9-Fmoc), 3.95 (dd, *J*=8.9, 5.0, 1H, C*H*α), 3.47 (dt, *J*=14.3, 5.9, 1H, $NH_2CH_2CH_2$), 3.33 (dt, *J*=14.2, 5.8, 1H, $BocNHCH_2CH_2$), 3.28 – 3.18 (m, 2H, C*H*$_2$δ), 2.97 (m, 2H, $NH_2CH_2CH_2$), 1.84 – 1.73 (m, 1H, C*H*$_2$β), 1.63 – 1.45 (m, 3H, C*H*$_2$β, C*H*$_2$γ). ***^{13}C-NMR*** (126 MHz, MeO**d** δ = 175.80 (*C*=OCHα), 158.77 (Fmoc *C*=O), 145.22, 145.07 (C-1$_a$-; C-8$_a$-Fmoc), 142.60 (C-4$_a$-; C-5$_a$-Fmoc), 128.80 (C-2-; C-7-Fmoc), 128.14 (C-3-; C-6-Fmoc), 126.20, 126.10 (C-1-; C-8-Fmoc), 120.95, 120.93 (C-4-; C-5-Fmoc), 67.92 (Fmoc *C*H$_2$), 56.32 (*C*Hα), 51.96 (*C*H$_2$δ), 48.38 (C-9-Fmoc), 40.81 ($NH_2CH_2CH_2$), 38.13 ($NH_2CH_2CH_2$), 29.96 (*C*H$_2$β), 26.41 (*C*H$_2$γ).

Preparation of 2AB- and AEAB- tagged glycans for comparing with FAA-tagged glycans' chromatographic profiles

To quantify the amounts of tagged glycans based on their florescence intensities, calibration curves were generated using 2AB- and FAA-tagged maltotriose as standards (**Supplementary Fig. 14**). The results indicated that FAA-linked maltotriose elicited a fluorescence signal 14 times stronger than the 2AB labelled glycan.

For comparing coupling efficiency, 3 nmol each of the following six sequence-defined glycans were mixed and tagged with FAA: cellobiose (Glc2), maltotriose (Mal3), α2,3-sialyllactose (3SL), lacto-N-tetraose (LNT), maltopentaose (Mal5) and maltoheptaose (Mal7), in a 10 µL solution containing 0.27 µmol FAA linker (15 eq). The HPLC profile of the tagged glycan mixture is in **Supplementary Fig. 1a**.

Aliquots, 0.5%, of the oligomannose *N*-glycan mixture from 40 mg RNAse B and of the complex type *N*-glycan mixture from fetuin (~ 10 ug scale) were tagged with 2AB[2] and AEAB[3] using reported procedures. In brief, a labelling solution containing 0.35 M 2-AB or AEAB with 0.5 M sodium cyanoborohydride in 7:3 DMSO/acetic acid was prepared and added to the lyophilized glycans. The reaction mixtures were incubated for 2 h at 60 °C for 2-AB labelling and 65 °C for AEAB labelling, respectively. Excess reagents were removed by paper chromatography, and the labelled glycans were subsequently analysed by HPLC profiling. The HPLC chromatograph of the tagged glycans are in **Supplementary Fig. 1b**-**g**. The glycan labelling protocol using FAA was further optimized based on the established 2-AB/AEAB procedures with several modifications. Specifically, the reaction was carried out at pH 6 and 50 °C, instead of 30% acetic acid at 60–65 °C, in order to minimize degradation of acid-labile glycans. Furthermore, the concentration of the labelling reagent was reduced from 0.35 M to 0.18 M, and the incubation time was extended from 2 h to 4 h, thereby enabling complete labelling under milder conditions.

Enzymatic modifications on FAA-*N*-glycan probe 36

The synthesis of biantennary *N*-glycan probes 37-41 (**Supplementary Fig. 19**) was performed by enzymatic modifications using published procedures[4]. In brief, for 2-3 sialyation, to a solution of probe 36 (100 nmol, 1.0 eq) and cytidine-5'-monophosphate-5-N-acetylneuraminic acid (CMP-Sia) (2.5 µmol, 25 eq) in 10 uL 0.1M Tris·HCl buffer containing 10 mM $MgCl_2$, 2→3 sialyltransferase from *Pasteurella multocida* (Merck, Dorset, UK) (15 mU) and alkaline phosphatase from calf intestine (CIAP) (Merck, Dorset, UK) (10 U) were added sequentially. The reaction was kept at 37 °C for 2 h and the product was purified on NP HPLC. For 1-2 fucosylation, to a microtube containing dry probe 36 (25 nmol, 1.0 eq) and 6-deoxy-β-L-galactopyranosylguanosine 5′-diphosphate (GDP-Fuc) (0.4 µmol, 16 eq), 25 uL 0.1M Tris·HCl buffer containing 10 mM $MgCl_2$, 1→2 fucosyltransferase from *Helicobacter hepaticus* (Chemily, Georgia, US) (40 mU) and CIAP (10 U) were added. The reaction was kept at 37 °C for 16 h and the product was purified on NP HPLC. For 1-3 fucosylation, to a solution of probe 36, 37 and 40 (10 nmol, 1.0 eq respectively) and GDP-Fuc (0.1 µmol, 10 eq)

in 10 uL 0.1M Tris·HCl buffer containing 10 mM $MgCl_2$ and 10 mM $MnCl_2$, 1→3/4 fucosyltransferase from *Helicobacter mustelae* (Chemily, Georgia, US) (10 mU) and CIAP (10 U) were added. The reaction was kept at 37 ^oC for 16 h and the product was purified on NP HPLC. The characterizations of the products are in **Supplementary Fig. 15**.

Generation of Fmoc-deprotected FAA glycan probes for covalent arrays
For covalent arrays the Fmoc group was removed from the quantified FAA-tagged glycans. One nmol of each in H_2O, was added to a plastic Eppendorf vial containing 15 mg piperazine resin (Merck, Dorset, UK) and was topped up with H_2O to 100 µL. The reaction was kept at R.T. for 16 h and MS analysis indicated full deprotection of the Fmoc group. The resin was separated by filtration and the freshly prepared probe in the filtrate was lyophilized to dryness. To each glycan probe (1 nmol), 10 µL of 0.1 M phosphate buffer (pH 8.5) was added to have the probe at 100 µM. Selected MS spectra of FAA glycan probes after Fmoc removal are in **Supplementary Fig. 16**.

Generation of FAA-NGL probes for noncovalent glycan arrays
To a solution of DHPE (3.0 mg, 4.5 µmol, 1 eq) in anhydrous $CHCl_3$:MeOH 2:1 (1.5 mL), DBCO-NHS (3.6 mg, 9 µmol, 2 eq, Conju-probe, San Diego, US) with DIPEA (3.2 µL, 20 µmol, 4 eq) in $CHCl_3$ (0.5 mL) was added dropwise. The reaction mixture was stirred at R.T. for 16 h and followed by HPTLC. The reaction mixture was first extracted three times with 2 mL H_2O and purified by flash column chromatography (solvent $CHCl_3$:MeOH 10:0 to 8.5:1.5) to give DBCO-DHPE in 82% yield (3.5 mg, 3.7 µmol). R_f = 0.4 ($CHCl_3$:MeOH 9:1). ***HR-ESI-MS***, *m/z*: 949.6464 ($[M-H]^-$, calc. 949.6435).

For conversion to NGLs, 10 nmol of dry FAA-tagged glycans were each dissolved in 40 µL $CHCl_3$:MeOH:100mM ammonium acetate in H_2O 25:25:8 containing 40 nmol of DBCO-DHPE. The reaction was kept at R.T. for 16 h and the NGLs purified by NP HPLC with an amide column using $CHCl_3$:MeOH:H_2O (Solvent A 130:70:9 to Solvent B 10:20:8). A short gradient was used here to effectively remove excess DBCO-DHPE. The column was then kept in 95% solvent A for 5 min to elute DBCO-DHPE followed by switching to 90% B for 4 min isocratically to elute the NGL product. The MS spectra are shown in **Supplementary Fig. 15**. The products were quantified[1].

SUPPLEMENTARY DISCUSSION

Details of binding signals given by fucosylated glycans using antibodies and plant lectins
The **anti-H type 1** antibody (clone 17-206) could tolerate terminal α-Gal and bound to the blood group B-hexa type 1 probe (#3, **Fig. 4a**). The **anti-H type 2** antibody BE2 bound strongly to the H type 2 pentasaccharide LNnFPI (#9) and biantennary *N*-glycan F2H5N4 (#37) but not the H type 2 globo-sequence GbH2 (#19, **Fig. 4b**), indicating the importance of backbone sequence for binding by this antibody.

Of the three **anti-Le^x** antibodies, only anti-L5 bound to the two sequences (#12, #38) that display Le^x motif on a trisaccharide backbone (**Supplementary Fig. 3**). The BG7 (clone P12) resembles anti-SSEA-1[7] which was known to require a longer backbone[6]; neither antibody gave binding signals in the covalent or noncovalent FAA glycan arrays (**Fig. 4g, Supplementary Fig. 3**). Weak binding of anti-L5 to Le^a probe LNFPII (#10) (**Supplementary Fig. 3e**) was detected as previously noted in the original chromatogram binding study[8].

The commercial **anti-Le^y**, BG8 (clone F3) gave binding signals with H type 2 probe LNnFPI (#9, **Fig. 4b**), in contrast to the anti-Le^y monoclonal antibody AH6 from Henrik Clausen Lab[9], which showed binding exclusively to Le^y related probes (#13 and #39, **Supplementary Fig. 3f**). Worthy of note, in the covalent HD array only, the commercial anti-Le^y BG8 gave strong binding also to the two Le^x probes (#12 and #38) in the absence of the blood group H fucose. Thus the high glycan density of the HD covalent array may increase the likelihood of detecting binding to 'weak' binders, reminiscent of findings from an earlier study on anti-HIV antibody recognition[10].

AAL showed binding to blood group H glycans and a broad spectrum of other fucosylated glycans. However, a lack of significant binding to blood group A and B related sequences was observed in all three types of arrays (**Fig 4d**). This is in accord with information provided in the recently published lectin guide[11] and data from our conventional NGL-based screening arrays (unpublished).

Details of binding signals given by two anti-ganglioside antibodies
Anti-GD1a gave binding to GD1a glycan probes (#31) in covalent and noncovalent array, and GD1a ceramide (#46) in noncovalent array (**Supplementary Fig. 4a**).

Anti-Gb3 gave binding to Gb3 glycan probes (#15) in all three arrays specifically (**Supplementary Fig. 4b**).

Details of binding signals given by Sia, Man, Gal, GalNAc terminating glycans using plant lectins
SNA bound to the two terminal α2-6 sialylated probes (#25, #42) in both covalent and NGL arrays as predicted. Surprisingly weak binding to terminal α2-3 sialylated biantennary *N*-glycan (#40) was also observed (**Supplementary Fig. 5a**).

MAA-I gave binding signals with terminal α2-3 Sia on type 2 LacNAc probes (#24, #40 **Supplementary Fig. 5b**) as predicted, but not to probe LSTa with α2-3-linked Sia on type 1 LacNAc backbone (#23). On the covalent arrays weak bindings to the type 2 LacNAc probes in the absence of sialylation (#6, #7, #36 **Supplementary Fig. 5b**) was observed, which is consistent with its specificity to type 2 backbones.

ConA Binding signals to high-mannose and complex type *N*-glycans were in accord with previous knowledge (**Supplementary Fig. 6a**).

PNA gave binding with all of the Gal-GalNAc terminating globoside and ganglioside glycan probes (#17, #29, #32, #35) as predicted (**Supplementary Fig. 6b**).

RCA120 binding to terminal type2 LacNAc (#6, #7, #36) 2-6 sialyl LacNAc probes (#41) are in accordance with previous knowledge. The relatively weak to biantennary Le^x terminating *N*-glycan is noted (**Supplementary Fig. 6c**).

WGA binding to NH-acetyl terminating glycan probes, GalNAc terminating blood group A related (#1, #2, #20), Forssmann antigen (#14) and Sia terminating (#23, #24, #27, #28) are in accord with previous knowledge (**Supplementary Fig. 6d**). The relatively strong binding on the covalent arrays to LacNAc or polyLacNAc based sequences (#4, #7, #9, #19) likely to reflect the relatively high concentration of the lectin used in this study[12].

Details of binding signals given by CLRs

DC-SIGN in common with the other two CLRs is known to bind to oligomannose glycans[13, 14]. In addition, it binds to fucosyl glycans including H type 2 and Le^a, Le^b, Le^x and Le^y-related sequences [15]. Under the assay conditions used, the top binders for DC-SIGN in noncovalent array were oligomannose *N*-glycans (#43, #44) and biantennary H type 2 (#37) and Le^x-terminating complex type *N*-glycans (#37, #38), followed by H type 2 globo-sequence GbH2 (#19) and the Lewis type antigens (#10-#13). The preference for the oligomannose *N*-glycan probes was less prominent in the covalent arrays (**Fig 5a**). **DC-SIGNR** showed a more restricted binding profile towards *N*-glycan probes including oligomannose (#43, #44) *N*-glycans with all three arrays. Notably, DC-SIGNR exhibited strong binding to the Le^x-*N*-glycan (#38) in the covalent arrays, whereas only weak binding was observed in the noncovalent array (**Fig 5b**). To our knowledge, this finding represents novel evidence for the binding of DC-SIGNR to glycans beyond oligo/high-mannose *N*-glycans[13]. **Langerin** bound almost exclusively to the high-mannose $Man_9GlcNAc_2$ probe in three types of FAA arrays. The binding intensity was considerably weaker in the CL array than the HD and noncovalent arrays (**Fig 5c**). The lack of binding to $Man_5GlcNAc_2$ highlights the requirement of α1,2-linked Man for binding by this CLR, in accord with the recent findings using an oligomannose isomer-focused array[14].

Details of binding signals given by E-selectins

E-selectin: The high avidity IgM construct of murine E-selectin gave strong binding both with the covalent and noncovalent arrays to the potent SLe^x terminating probes (#28, #41, **Fig. 5h**) and relatively weak binding to asialo-Le^a, Le^b probes (#10, #11) which are low affinity ligands for E-selectin; this is in accord with previous observation in cell based analyses[16]. The His-tagged human E-selectin gave binding signals with the two SLe^x probes (#28, #41, **Fig. 5g**) but not with the Le^a and Le^b probes.

Details of binding signals given by bacterial toxins on non-sialyl glycans

With the three types of FAA arrays, binding of the three toxins could be detected to non-sialyl glycans[17, 18, 19, 20]. Greater differences were observed in the different array formats than with the ganglioside related sequences. For example, binding to Le^y probe (#13), the known ligand for the three toxins[21], was well detected in the covalent CL array but only weakly in the HD arrays and surprisingly almost absent in the noncovalent array. In contrast, the LacNAc-terminating glycans were more prominently bound in the noncovalent array compared to the covalent arrays: the complex type *N*-glycan (#36) was bound by all three toxins, and LNnT (#5) and LN3 (#6) by the cCTB and LTBh. The binding of the three CTB-related toxins to blood group antigens was observed with varying intensities in the three arrays (**Fig. 6**) and these are described in detail as below.

cCTB, binding was observed to blood group A type 1 (#1) in both covalent and noncovalent arrays. This has not been reported to our knowledge. In the covalent arrays, additional binding was detected to the two Le^y terminating (#13, #39) and two SLe^x glycan probes (#28, #41) in agreement with the results obtained from inhibition of cell binding studies using soluble oligosaccharides[22].

El Tor CTB showed binding exclusively to H type 2 probe LNnFPI (#9) among all the ABO antigens tested. This finding provides an explanation for the blood group O association with the severity of cholera caused by the El Tor biotype of *V. cholerae*[23]. This toxin also bound to α1-2-fucosylated Lewis antigens Le^b (#11) and Le^y (#13), particularly with the CL array.

LTBh is reported to bind to blood group A and B but not H-active glycolipids on human red blood cells[24]. In this study, we were able to detect LTBh binding to H- in addition to A- and B-oligosaccharides (#2, #4, #9), mostly on type 2 sequences. However, little binding was detected to the N-glycan probe with short type 2 H outer arms (#37) and Globo-H analog GbH2 (#19) having the H-type 2 epitope. LTBh also bound well to globopentaose Gb5 (#17), Globo-A and B probes (#20, #21), but not Globo-H (#18).

SUPPLEMENTARY REFERENCES

1. Liu Y*, et al.* Neoglycolipid-Based Oligosaccharide Microarray System: Preparation of NGLs and Their Noncovalent Immobilization on Nitrocellulose-Coated Glass Slides for Microarray Analyses. *Methods Mol Biol*. **808,** 117-36 (2012)

2. Bigge JC, Patel TP, Bruce JA, Goulding PN, Charles SM, Parekh RB. Nonselective and Efficient Fluorescent Labeling of Glycans Using 2-Amino Benzamide and Anthranilic Acid. *Analytical Biochemistry* **230**, 229-238 (1995).

3. Song X, Xia B, Stowell SR, Lasanajak Y, Smith DF, Cummings RD. Novel fluorescent glycan microarray strategy reveals ligands for galectins. *Chemistry & biology* **16 1**, 36-47 (2009).

4. Li L*, et al.* Efficient chemoenzymatic synthesis of an N-glycan isomer library. *Chemical Science* **6**, 5652-5661 (2015).

5. Gao C, Wei M, McKitrick TR, McQuillan AM, Heimburg-Molinaro J, Cummings RD. Glycan Microarrays as Chemical Tools for Identifying Glycan Recognition by Immune Proteins. *Front Chem* **7**, 833 (2019).

6. Blixt O*, et al.* Printed covalent glycan array for ligand profiling of diverse glycan binding proteins. *Proc Natl Acad Sci U S A* **101**, 17033-17038 (2004).

7. Gooi HC, Feizi T, Kapadia A, Knowles BB, Solter D, Evans MJ. Stage-specific embryonic antigen involves αl→ 3 fucosylated type 2 blood group chains. *Nature* **292**, 156-158 (1981).

8. Streit A*, et al.* The Lex Carbohydrate Sequence Is Recognized by Antibody to L5, a Functional Antigen in Early Neural Development. *Journal of Neurochemistry* **66**, 834-844 (1996).

9. Young WW, Portoukalian J, Hakomori S. Two monoclonal anticarbohydrate antibodies directed to glycosphingolipids with a lacto-N-glycosyl type II chain. *Journal of Biological Chemistry* **256**, 10967-10972 (1981).

10. Kong L*, et al.* Supersite of immune vulnerability on the glycosylated face of HIV-1 envelope glycoprotein gp120. *Nature Structural & Molecular Biology* **20**, 796-803 (2013).

11. Bojar D*, et al.* A Useful Guide to Lectin Binding: Machine-Learning Directed Annotation of 57 Unique Lectin Specificities. *ACS Chemical Biology* **17**, 2993-3012 (2022).

12. Wu Z*, et al.* Identification of the binding roles of terminal and internal glycan epitopes using enzymatically synthesized N-glycans containing tandem epitopes. *Organic & Biomolecular Chemistry* **14**, 11106-11116 (2016).

13. Guo Y*, et al.* Structural basis for distinct ligand-binding and targeting properties of the receptors DC-SIGN and DC-SIGNR. *Nature Structural & Molecular Biology* **11**, 591-598 (2004).

14. Gao C*, et al.* Differential recognition of oligomannose isomers by glycan-binding proteins involved in innate and adaptive immunity. *Science Advances* **7**, eabf6834 (2021).

15. van Liempt E*, et al.* Specificity of DC-SIGN for mannose- and fucose-containing glycans. *FEBS Letters* **580**, 6123-6131 (2006).

16. Larkin M*, et al.* Spectrum of sialylated and nonsialylated fuco-oligosaccharides bound by the endothelial-leukocyte adhesion molecule E-selectin. Dependence of the carbohydrate binding activity on E-selectin density. *Journal of Biological Chemistry* **267**, 13661-13668 (1992).

17. Holmner A, Askarieh G, Okvist M, Krengel U. Blood group antigen recognition by Escherichia coli heat-labile enterotoxin. *J Mol Biol* **371**, 754-764 (2007).

18. Mandal PK*, et al.* Towards a structural basis for the relationship between blood group and the severity of El Tor cholera. *Angew Chem Int Ed Engl* **51**, 5143-5146 (2012).

19. Heggelund JE, Burschowsky D, Bjornestad VA, Hodnik V, Anderluh G, Krengel U. High-Resolution Crystal Structures Elucidate the Molecular Basis of Cholera Blood Group Dependence. *PLoS Pathog* **12**, e1005567 (2016).

20. Heim JB, Hodnik V, Heggelund JE, Anderluh G, Krengel U. Crystal structures of cholera toxin in complex with fucosylated receptors point to importance of secondary binding site. *Sci Rep* **9**, 12243 (2019).

21. Mandal PK*, et al.* Towards a Structural Basis for the Relationship Between Blood Group and the Severity of El Tor Cholera. *Angewandte Chemie International Edition* **51**, 5143-5146 (2012).

22. Wands AM*, et al.* Fucosylated Molecules Competitively Interfere with Cholera Toxin Binding to Host Cells. *ACS Infectious Diseases* **4**, 758-770 (2018).

23. Harris JB*, et al.* Blood Group, Immunity, and Risk of Infection with <i>Vibrio cholerae</i> in an Area of Endemicity. *Infection and Immunity* **73**, 7422-7427 (2005).

24. Barra JL, Monferran CG, Balanzino LE, Cumar FA. Escherichia coli heat-labile enterotoxin preferentially interacts with blood group A-active glycolipids from pig intestinal mucosa and A- and B-active glycolipids from human red cells compared to H-active glycolipids. *Molecular and Cellular Biochemistry* **115**, 63-70 (1992).

25. Streit A*, et al.* The Le(x) carbohydrate sequence is recognized by antibody to L5, a functional antigen in early neural development. *J Neurochem* **66**, 834-844 (1996).

26. Abe K, McKibbin JM, Hakomori S. The monoclonal antibody directed to difucosylated type 2 chain (Fuc alpha 1 leads to 2Gal beta 1 leads to 4[Fuc alpha 1 leads to 3]GlcNAc; Y Determinant). *J Biol Chem* **258**, 11793-11797 (1983).

27. Galustian C, Childs RA, Stoll M, Ishida H, Kiso M, Feizi T. Synergistic interactions of the two classes of ligand, sialyl-Lewis(a/x) fuco-oligosaccharides and short sulpho-motifs, with the P- and L-selectins: implications for therapeutic inhibitor designs. *Immunology* **105**, 350-359 (2002).

28. Olsvik O*, et al.* Use of automated sequencing of polymerase chain reaction-generated amplicons to identify three types of cholera toxin subunit B in Vibrio cholerae O1 strains. *Journal of Clinical Microbiology* **31**, 22-25 (1993).

29. Lebens M, Holmgren J. Structure and arrangement of the cholera toxin genes in Vibrio cholerae O139. *FEMS Microbiology Letters* **117**, 197-202 (1994).

30. Li L*, et al.* Microarray analyses of closely related glycoforms reveal different accessibilities of glycan determinants on N-glycan branches. *Glycobiology* **30**, 334-345 (2019).
