# Supplementary material for: Versatile Glycan Probes for Multiplatform Investigation of Glycan Interactions with Proteins, Viruses, and Cells
Source: Nat Commun. 2026 Jul 17;17:8750. doi: 10.1038/s41467-026-75206-2 (PMC13493820; doi:10.1038/s41467-026-75206-2)
Supplement: Supplementary file 2 [file 41467_2026_75206_MOESM2_ESM.pdf]

Description of Additional Supplementary Files

File Name: Supplementary Data 1

Description: Probe list of noncovalent FAA NGL array

File Name: Supplementary Data 2

Description: Probe list of covalent FAA glycan array

File Name: Supplementary Data 3

Description: Binding data matrix of antibodies and plant lectins on FAA glycan arrays

File Name: Supplementary Data 4

Description: Binding data matrix of other GBPs on FAA glycan arrays
