# Supplementary material for: Versatile Glycan Probes for Multiplatform Investigation of Glycan Interactions with Proteins, Viruses, and Cells
Source: Nat Commun. 2026 Jul 17;17:8750. doi: 10.1038/s41467-026-75206-2 (PMC13493820; doi:10.1038/s41467-026-75206-2)
Supplement: Supplementary file 3 [file 41467_2026_75206_MOESM7_ESM.pdf]

nature portfolio

Corresponding author(s): Dr. Yan Liu

Last updated by author(s): Oct 23, 2025

Reporting Summary

Nature Portfolio wishes to improve the reproducibility of the work that we publish. This form provides structure for consistency and transparency in reporting. For further information on Nature Portfolio policies, see our Editorial Policies and the Editorial Policy Checklist.

Statistics

For all statistical analyses, confirm that the following items are present in the figure legend, table legend, main text, or Methods section.

n/a	Confirmed	
☐	☒	The exact sample size (n) for each experimental group/condition, given as a discrete number and unit of measurement
☐	☒	A statement on whether measurements were taken from distinct samples or whether the same sample was measured repeatedly
☐	☒	The statistical test(s) used AND whether they are one- or two-sided<br>*Only common tests should be described solely by name; describe more complex techniques in the Methods section.*
☒	☐	A description of all covariates tested
☒	☐	A description of any assumptions or corrections, such as tests of normality and adjustment for multiple comparisons
☐	☒	A full description of the statistical parameters including central tendency (e.g. means) or other basic estimates (e.g. regression coefficient) AND variation (e.g. standard deviation) or associated estimates of uncertainty (e.g. confidence intervals)
☐	☒	For null hypothesis testing, the test statistic (e.g. F, t, r) with confidence intervals, effect sizes, degrees of freedom and P value noted<br>*Give P values as exact values whenever suitable.*
☒	☐	For Bayesian analysis, information on the choice of priors and Markov chain Monte Carlo settings
☒	☐	For hierarchical and complex designs, identification of the appropriate level for tests and full reporting of outcomes
☒	☐	Estimates of effect sizes (e.g. Cohen's d, Pearson's r), indicating how they were calculated

Our web collection on statistics for biologists contains articles on many of the points above.

Software and code

Policy information about availability of computer code

Data collection	N/A
Data analysis	N/A

For manuscripts utilizing custom algorithms or software that are central to the research but not yet described in published literature, software must be made available to editors and reviewers. We strongly encourage code deposition in a community repository (e.g. GitHub). See the Nature Portfolio guidelines for submitting code & software for further information.

Data

Policy information about availability of data

All manuscripts must include a data availability statement. This statement should provide the following information, where applicable:

- Accession codes, unique identifiers, or web links for publicly available datasets
- A description of any restrictions on data availability
- For clinical datasets or third party data, please ensure that the statement adheres to our policy

All data supporting the findings are available within the article and its Supplementary Information. Raw and processed microarray binding matrices, as well as BLI source data, are provided in Supplementary Data 1–6.

Research involving human participants, their data, or biological material

Policy information about studies with human participants or human data. See also policy information about sex, gender (identity/presentation), and sexual orientation and race, ethnicity and racism.

Reporting on sex and gender	N/A
Reporting on race, ethnicity, or other socially relevant groupings	N/A
Population characteristics	N/A
Recruitment	N/A
Ethics oversight	N/A

Note that full information on the approval of the study protocol must also be provided in the manuscript.

Field-specific reporting

Please select the one below that is the best fit for your research. If you are not sure, read the appropriate sections before making your selection.

[x] Life sciences [ ] Behavioural & social sciences [ ] Ecological, evolutionary & environmental sciences

For a reference copy of the document with all sections, see nature.com/documents/nr-reporting-summary-flat.pdf

Life sciences study design

All studies must disclose on these points even when the disclosure is negative.

Sample size	Sample sizes were determined empirically based on reproducibility in previous glycan array and BLI studies.<br>For microarray assays, each glycan probe was printed in quadruplicate (n = 4) for covalent arrays and duplicate (n = 2) on two sample levels for noncovalent arrays.<br>For BLI analyses, each experiment was performed in duplicates (n = 2).<br>For flow cytometry analyses, each experiment was performed in three independent biological replicates (n = 3).
Data exclusions	No data were excluded from the analyses.<br>All replicates were included unless a printing or technical error (e.g., misprinted spot) was documented and noted in the figure legends.
Replication	For the glycan array binding experiments, each sample was analyzed once, as the array platform is highly standardized and has well-established reproducibility in the field. Replicate printing of each glycan probe (duplicate or quadruplicate spots) provided internal technical replicates that yielded consistent signal patterns.<br>For the BLI analyses, two samples were re-tested upon reviewers’ request, showing reproducible binding curves.<br>Flow cytometry assays were independently repeated at least three times, producing consistent and reproducible results.<br>Replication outcomes are detailed in the figure legends and Methods sections.
Randomization	Randomization was not applicable.<br>Experimental conditions (e.g., glycan sequences and array positions) were predefined and systematically printed or processed; no treatment groups or random assignment were involved.
Blinding	Blinding was not applicable.<br>Data collection and analysis were performed using automated instruments (GenePix, Octet, FACS), which minimize user bias.<br>Interpretation was based on quantitative signal outputs rather than subjective scoring.

Reporting for specific materials, systems and methods

We require information from authors about some types of materials, experimental systems and methods used in many studies. Here, indicate whether each material, system or method listed is relevant to your study. If you are not sure if a list item applies to your research, read the appropriate section before selecting a response.

Materials & experimental systems

n/a	Involved in the study
☐	☒ Antibodies
☐	☒ Eukaryotic cell lines
☒	☐ Palaeontology and archaeology
☒	☐ Animals and other organisms
☒	☐ Clinical data
☒	☐ Dual use research of concern
☒	☐ Plants

Methods

n/a	Involved in the study
☒	☐ ChIP-seq
☐	☒ Flow cytometry
☒	☐ MRI-based neuroimaging

Antibodies

Antibodies used	A total of 14 monoclonal antibodies recognizing carbohydrate antigens were used for glycan array binding analyses.<br>Detailed information is provided in Supplementary Table 3.<br>Key antibodies include:<br>Anti-Lea (BG5, clone T174, BioLegend, Cat. #922202, Mouse IgG, 1:50)<br>Anti-Leb (BG6, clone T218, BioLegend, Cat. #922302, Mouse IgM, 1:50)<br>Anti-Lex (BG7, clone P12, BioLegend, Cat. #912901, Mouse IgM, 1:50)<br>Anti-Lex (SSEA-1) (clone MC-480, DHSB, Cat. #MC-480-c, Mouse IgM, 1:50)<br>Anti-Ley (BG8, clone F3, BioLegend, Cat. #912501, Mouse IgM, 1:50)<br>Anti-Ley (AH6) (Henrik Clausen Lab, Mouse IgM, 1:5)<br>Anti-A (BG2, clone T36, BioLegend, Cat. #921902, Mouse IgG, 1:10)<br>Anti-B (HEB29, Abcam, Cat. #Ab2524, Mouse IgM, 1:10)<br>Anti-H type 1 (clone 17-206, Invitrogen, Cat. #14-9810-82, Mouse IgG, 1:50)<br>Anti-H type 2 (BE2, Henrik Clausen Lab, Mouse IgM, 1:2)<br>Anti-SLex (CD15s, clone CSLEX1, BD Pharmingen, Cat. #551344, Mouse IgM, 1:50)<br>Anti-GD1a (clone GD1a-1, Millipore, Cat. #MAB5606, Mouse IgG, 1:250)<br>Anti-Gb3 (clone BGR23, TCI, Cat. #A2506, Mouse IgG, 1:140)<br>Anti-Lex (L5) (Rat IgM, gift from Dr Andrea Streit, King's College London)
Validation	All primary antibodies were validated for glycan specificity by functional testing on the FAA glycan arrays. Each antibody produced binding profiles consistent with its known carbohydrate epitope as reported in the literature and manufacturer documentation.<br>Reference validation data are available from manufacturer datasheets (BioLegend, BD Pharmingen, Abcam, Millipore, Invitrogen, TCI) and published studies describing carbohydrate specificity (e.g., Young et al., J. Biol. Chem. 1981; Streit et al., J. Neurochem. 1996; Abe et al., J. Biol. Chem. 1983).<br>One key point of this work is to assess whether the antibodies display the expected binding to their antigenic glycan structures, which had not been systematically reported before.<br>These findings are described in the main text section "Recognition of fucosylated glycans by antibodies and lectins" and in the Supplementary Discussion, with a concise summary provided in Supplementary Table 1.<br>No unexpected cross-reactivity beyond known glycan motifs was observed, confirming that all antibodies were valid and specific for glycan array applications.

Eukaryotic cell lines

Policy information about cell lines and Sex and Gender in Research

Cell line source(s)	HEK 293-derived cell lines were used in this study, including HEK Siglec-9 and the control HEK Null1 cell line.<br>Both were obtained from R&D Systems (Minneapolis, USA) as part of the validated Siglec cell platform.<br>The parental HEK 293 line originates from human embryonic kidney tissue (female donor).
Authentication	Cell line identity was confirmed by the supplier (R&D Systems) through standard authentication procedures, including morphology and expression analysis of the transfected Siglec-9 receptor.<br>No additional independent authentication was performed in our laboratory.
Mycoplasma contamination	All cell lines were routinely tested and confirmed negative for mycoplasma contamination using a PCR-based detection kit (Lonza MycoAlert).
Commonly misidentified lines<br>(See ICLAC register)	No commonly misidentified or cross-contaminated cell lines listed in the ICLAC register were used in this study.

Plants

Seed stocks	N/A
Novel plant genotypes	N/A
Authentication	N/A

Flow Cytometry

Plots

Confirm that:

- [x] The axis labels state the marker and fluorochrome used (e.g. CD4-FITC).
- [x] The axis scales are clearly visible. Include numbers along axes only for bottom left plot of group (a 'group' is an analysis of identical markers).
- [x] All plots are contour plots with outliers or pseudocolor plots.
- [x] A numerical value for number of cells or percentage (with statistics) is provided.

Methodology

Sample preparation	HEK Siglec-9 and HEK Null1 cell lines (R&D Systems) were cultured under standard conditions (DMEM + 10 % FBS + 1 % penicillin/streptomycin, 37 °C, 5 % CO_2).<br>Cells were detached with 0.25 % trypsin-EDTA, washed twice in cold PBS containing 1 % BSA, and resuspended at 1×10^6 cells/mL.<br>Cells were incubated with fluorescently labelled FAA-tagged glycan probes (100 µM, 1 h at 4 °C), washed twice, and immediately analyzed by flow cytometry.
Instrument	Flow cytometry data were acquired on a BD FACSCalibur (Becton Dickinson, San Jose, CA, USA) equipped with a 488-nm argon laser and 530/585-nm emission filters.
Software	Data were collected and analyzed using CellQuest Pro software (BD Biosciences).<br>No custom code was used; only standard software functions were applied for data acquisition and histogram generation.
Cell population abundance	All analyzed samples consisted of homogeneous HEK cell suspensions with > 95 % viable cells, as assessed by forward/side scatter (FSC/SSC) profiles and trypan-blue exclusion.<br>No cell sorting was performed; thus, the entire viable population was included in the analysis.
Gating strategy	Cells were first gated by FSC/SSC to exclude debris and doublets.<br>Subsequent gates defined single, live cells.<br>Fluorescence gates were set using unstained and negative-control (HEK Null1) samples to define background levels.<br>HEK Siglec-9 cells exhibiting fluorescence above this threshold were defined as positive binding populations.<br>Mean fluorescence intensity (MFI ± SD) values from three independent experiments are reported in Figure 8.

- [ ] Tick this box to confirm that a figure exemplifying the gating strategy is provided in the Supplementary Information.
