# Supplementary material for: Versatile Glycan Probes for Multiplatform Investigation of Glycan Interactions with Proteins, Viruses, and Cells
Source: Nat Commun. 2026 Jul 17;17:8750. doi: 10.1038/s41467-026-75206-2 (PMC13493820; doi:10.1038/s41467-026-75206-2)
Supplement: Supplementary file 4 [file 41467_2026_75206_MOESM8_ESM.pdf]

nature portfolio

Peer Review File

Versatile Glycan Probes for Multiplatform Investigation of Glycan Interactions with Proteins, Viruses, and Cells

Corresponding Author: Dr Yan Liu

This file contains all reviewer reports in order by version, followed by all author rebuttals in order by version.

Version 0:

Reviewer comments:

Reviewer #1

(Remarks to the Author)
In this manuscript titled "Versatile Glycan Probes for Multiplatform 1 Investigation of Glycan Interactions with Proteins, Viruses, and Cells" by Yu et al., the major source of novelty is a new multifunctional linker for immobilizing glycans into arrays. Using this linker, they demonstrate the following: (1) print glycans with fluorophores linked at different concentrations, and show that fluorescence is dependent on concentration of printing solution, providing a readout, (ii) print covalently using codelink slides and noncovalently using lipid tails, and show that these maintain expected biological activity, (iii) test binding to viral and immune glycan binding proteins (GBPs), and (iv) show that this chemistry can be used for BLI analysis. There is a lot of work here, a lot, and the experiments and results are of the highest quality, and the conclusions supported by extensive Supporting Information (although images of all arrays should be provided). This is another example of the high-quality research for which the Imperial College laboratory is well-known.

The concern that this Reviewer has, and they acknowledge that it is a subjective one, is whether this manuscript solves any problems of significance in the field of glycan arrays or glycobiology. What is reported in this manuscript is a new chemistry for making monolayer arrays, and is another addition to the pantheon of such chemistries along with all of the associated challenges. These include (1) differences in selectivity or binding affinity to other chemistries that have been reported, leading to further confusion as to actual specificities of GBPs when comparing to previously published results. Who is to know which chemistry more accurately reflects the correct selectivity of the GBPs when two different surface chemistries differ in their selectivity. (2) Inherently weak binding that is unable to reproduce the binding in the biological context because of the 2D (flat) nature of the array. Increasingly, researchers are moving to polymeric scaffolds in an effort to access cluster glycoside effects. And (3) the inability to determine the absolute density of the glycans in the array, although with fluorescent probes they can determine relative density. As such, using this approach, very little can be learned when using this chemistry in terms of how multivalency or cooperativity can affect binding and in turn, selectivity.

A few minor comments/questions that the authors should address:
(1) In line 119, the authors refer to a "semi-quantitative measurements" of the glycans on the surface by the scanner. The Reviewer is not comfortable with this language as it suggests that the amount of glycan deposited on the surface is being measured, which it is not. The authors should substitute the language "semi-quantitative", which is vague, with a more descriptive phrase that denotes clearly that this is a relative measurement, and that the density of the glycan on the surface is unknown.

(2) in line 327, the authors state "...similar or equivalent for the majority -- 18 of 36 GBPs investigated...". The definition of majority, according to Miriam Webster is "a number or percentage equaling more than half of a total". The key part of this definition is "more than half". As such, the use of the word "majority" in this context is incorrect.

Otherwise, the manuscript is very clear and well-written.

Reviewer #2

(Remarks to the Author)
Congratulations on your excellent work. I see a lot of potential to use the BLI technology together with the glycan probes in the field of virology.

Here are my comments:

The authors used biotin to bring glycan probes to the sensor surface.

Would the authors expect different BLI results if they would capture the FAA glycans per amine coupling or non-covalently with an additional lipid, like non-covalent printing?

Have you tried this? This question is just out of personal interest and has nothing to do with the quality of the data presented.

The most important question for me in the BLI part is about the saturated signals of H1N1 and H5N1.
Do the authors have a more detailed explanation as to why the dependence on sugar loading cannot be recognized? Or even a certain randomness of the obtained virus signal? At first glance, it looks as if the virus binds non-specifically to the sensor surface, but this would also be seen with LSTc-FAA. With H1N1 it even looks as if the lowest sugar concentration gives the highest virus signal and the signal decreases with increasing sugar concentration. It is not possible to say for sure because the replicates are missing and therefore no statement can be made about the measurement accuracy. Why were H1N1 and H5N1 not also measured in the negative control?

Looking forward to the answers

Reviewer #3

(Remarks to the Author)
In this work, the authors develop tri-functional FAA probes that allow for versatile glycan array platforms. They assess binding activities of antibodies, lectins, and immune receptors using their systems, demonstrating different preferences of these various glycan binding proteins. While the work is interesting and provides an advance to the canonical glycan array platforms, the article is extremely disorganized and difficult to parse because of this. Additionally, a substantial amount of optimization, experimental design, and/or comparison to traditional glycan arrays are not addressed.

Notable issues:
- The SI figures and associated text are nearly impossible to follow. The first SI figure addressed is #6 with no reference to 1-5 in the text. The next SI figure referenced is SI Fig 19, followed by SI Fig 1f, then SI 17. Most of the SI tables are never referenced.
- Also, why is there discussion/text in the SI? Was this because of word limits? The discussion contained in the SI would be useful to have in the main text.
- In Figure SI 6, the authors suggest that these methods demonstrate "similar peak shapes", which does not appear to be true. Further, they say that the FAA platform affords higher sensitivity, however, the ion counts are nearly impossible to read. These claims should be further substantiated.
- The authors refer to an "optimized linker" but do not discuss the optimization, some discussion on this would be appreciated.
- At one point it is stated that 39 reducing glycans are being used, however, there are 44 in Figure 2. Why the discrepancy?
- Does SPAAC need to be used for this work? Would copper click not work?
- Fig 3c - all of these spots look very similar, is that expected? How do these measurements compare to a typical glycan array? For this experiment, it be worthwhile to investigate what the limit of detection is of their method, especially compared to traditional arrays. Also, the authors determine that 100 uM is "optimal" but it is unclear why this is the case.
- Man9-GN2 - this nomenclature is unusual, please standardize glycan structures throughout.
- At one point the authors reference "data not shown", please include this data.
- The authors do not address that a major drawback of all glycan array-like platforms is that often glycan binding proteins are also reliant on the presence of a protein backbone.
- It is unclear to me what the benefits are of using a covalent vs. non-covalent system.

Version 1:

Reviewer comments:

Reviewer #1

(Remarks to the Author)
All of this Reviewer's concerns have been addressed, including the additional datasets, the massaging of the language, and the responses regarding significance.

Reviewer #2

(Remarks to the Author)
My primary concern has been satisfactorily resolved, and the additional experiments successfully demonstrated a dependency on sugar loading. All of my questions have been addressed comprehensively, and I therefore have no further objections.

Reviewer #3

(Remarks to the Author)
The authors have addressed all of my concerns, and I appreciate their efforts. I believe the manuscript is now ready for publication.

Open Access This Peer Review File is licensed under a Creative Commons Attribution 4.0 International License, which permits use, sharing, adaptation, distribution and reproduction in any medium or format, as long as you give appropriate credit to the original author(s) and the source, provide a link to the Creative Commons license, and indicate if changes were made.
In cases where reviewers are anonymous, credit should be given to 'Anonymous Referee' and the source.
The images or other third party material in this Peer Review File are included in the article's Creative Commons license, unless indicated otherwise in a credit line to the material. If material is not included in the article's Creative Commons license and your intended use is not permitted by statutory regulation or exceeds the permitted use, you will need to obtain permission directly from the copyright holder.
To view a copy of this license, visit https://creativecommons.org/licenses/by/4.0/

Nature Communications manuscript NCOMMS-25-35413

Point-by-point responses to the reviewers' comments are provided in blue font.

Reviewer #1 (Remarks to the Author):

In this manuscript titled "Versatile Glycan Probes for Multiplatform 1 Investigation of Glycan Interactions with Proteins, Viruses, and Cells" by Yu et al., the major source of novelty is a new multifunctional linker for immobilizing glycans into arrays. Using this linker, they demonstrate the following: (1) print glycans with fluorophores linked at different concentrations, and show that fluorescence is dependent on concentration of printing solution, providing a readout, (ii) print covalently using codelink slides and noncovalently using lipid tails, and show that these maintain expected biological activity, (iii) test binding to viral and immune glycan binding proteins (GBPs), and (iv) show that this chemistry can be used for BLI analysis. There is a lot of work here, a lot, and the experiments and results are of the highest quality, and the conclusions supported by extensive Supporting Information (although images of all arrays should be provided). This is another example of the high-quality research for which the Imperial College laboratory is well-known.

*We sincerely appreciate the reviewer's thoughtful and encouraging comments on our work. In line with the suggestion, we have now provided the array images of all the arrays, which are included in **Supplementary Fig. 21**. Moreover, our plan is to deposit all the microarray data, including raw images (tiff files), quantified array results (gpr files), together with processed data to the newly developed International Glycan Microarray Data Repository (currently under beta-testing). We have indicated this new development in the MIRAGE document (**Supplementary Table 8**).*

The concern that this Reviewer has, and they acknowledge that it is a subjective one, is whether this manuscript solves any problems of significance in the field of glycan arrays or glycobiology. What is reported in this manuscript is a new chemistry for making monolayer arrays, and is another addition to the pantheon of such chemistries along with all of the associated challenges. These include (1) differences in selectivity or binding affinity to other chemistries that have been reported, leading to further confusion as to actual specificities of GBPs when comparing to previously published results. Who is to know which chemistry more accurately reflects the correct selectivity of the GBPs when two different surface chemistries differ in their selectivity. (2) Inherently weak binding that is unable to reproduce the binding in the biological context because of the 2D (flat) nature of the array. Increasingly, researchers are moving to polymeric scaffolds in an effort to access cluster glycoside effects.

Despite great advances with existing glycan microarray systems worldwide, it should be acknowledged that in all microarray platforms, covalent or non-covalent, glycans are artificially presented for binding studies. Glycan microarrays remain screening approaches, and lack of binding in one system cannot conclude that a protein does not recognize glycans. There are many possible causes of such "false negatives". Our aim in this study is to reduce the chances of such results, draw attention to the importance of glycan presentation, and provide flexibility across different technological platforms to enhance confidence in knowledge gained on glycan-mediated interactions.

We point out with respect that in the microarrays described in the present studies the glycans are already presented on 3D matrices on planar surfaces. We consider the lipid-linked glycan probes, when printed in the liposomal formulation, may offer a more favourable clustered and oriented presentation compared to the amino-glycans printed on 3D polymer coated glass slides. Furthermore, the versatility of the FAA strategy allows glycan probes to be conjugated into polymeric scaffolds via either azido or amino groups. As part of future work, we will conduct comparative studies of FAA–glycan polymers and glycan-coated liposomes (NGL-based) to evaluate their binding capacities in both solution and solid-phase formats.

And (3) the inability to determine the absolute density of the glycans in the array, although with fluorescent probes they can determine relative density. As such, using this approach, very little can be learned when using this chemistry in terms of how multivalency or cooperativity can affect binding and in turn, selectivity.

We agree with the reviewer that the absolute amount of the probes immobilized on the array is difficult to determine. Our experimental design in Fig. 3 aimed to assess the feasibility of addressing this issue. Although the visualized probe signal is not quantitatively linear with the printing concentration, the on-slide fluorescence provides a means to confirm whether each glycan probe was successfully delivered and whether comparable amounts of probes have been immobilized.

A few minor comments/questions that the authors should address:

(1) In line 119, the authors refer to a "semi-quantitative measurements" of the glycans on the surface by the scanner. The Reviewer is not comfortable with this language as it suggests that the amount of glycan deposited on the surface is being measured, which it is not. The authors should substitute the language "semi-quantitative", which is vague, with a more descriptive phrase that denotes clearly that this is a relative measurement, and that the density of the glycan on the surface is unknown.

We thank the reviewer for this helpful suggestion and agree that the term “semi-quantitative” may be misleading in this context. We have revised the subheading to “On-array visualization of immobilized FAA glycan probes”, as well as in later part of discussions. In the text, we have now clarified that the fluorescent signals do not reflect the absolute amount of glycans on the array surface.

(2) in line 327, the authors state "...similar or equivalent for the majority -- 18 of 36 GBPs investigated...". The definition of majority, according to Miriam Webster is "a number or percentage equaling more than half of a total". The key part of this definition is "more than half". As such, the use of the word "majority" in this context is incorrect.

We thank the reviewer for pointing out the inaccuracy in our use of the term “majority”, and we have revised the text accordingly under Discussion (pages 7-8). We have pointed out that the 36 GBPs tested were not intended as a general survey of all GBPs. Rather, they were selected as a representative set, focusing on cases where we anticipated potential differences between the covalent and noncovalent arrays based on prior knowledge. Thus, the finding that about half of these representative GBPs showed similar or equivalent binding adds confidence in the robustness and reliability of both array platforms, while also highlighting those GBPs that require specific glycan presentation for binding.

Otherwise, the manuscript is very clear and well-written.

Reviewer #2 (Remarks to the Author):

Congratulations on your excellent work. I see a lot of potential to use the BLI technology together with the glycan probes in the field of virology.

Here are my comments:

The authors used biotin to bring glycan probes to the sensor surface.

Would the authors expect different BLI results if they would capture the FAA glycans per amine coupling or non-covalently with an additional lipid, like non-covalent printing?

Have you tried this? This question is just out of personal interest and has nothing to do with the quality of the data presented.

We agree with the reviewer that comparing covalently and non-covalently coupled glycans in the BLI system would be of interest and is something we would like to pursue. Although various types of BLI sensor chemistries are available, our experience and advice from Sartorius suggest that the streptavidin-biotin system generally provides the most robust probe immobilization and reliable kinetic detection. For this reason, we initially focused our BLI studies on FAA-based biotinylated glycan probes.

We also considered covalent immobilization methods using carboxylic acid–covered sensor tips activated by EDC to capture amine groups, which could potentially yield reusable glycan-coated sensor tips for kinetic studies. However, since the amide-coupling conditions were developed for protein immobilization, systematic testing would be required to ensure reliable capture of amine-tagged glycans. To the best of our knowledge, no established method exists for immobilizing lipid-linked glycan probes on the sensor tips, although protocols for immobilizing glycolipids on SPR surfaces are available. We will certainly explore these approaches further as part of our future studies.

The most important question for me in the BLI part is about the saturated signals of H1N1 and H5N1.

Do the authors have a more detailed explanation as to why the dependence on sugar loading cannot be recognized? Or even a certain randomness of the obtained virus signal? At first glance, it looks as if the virus binds non-specifically to the sensor surface, but this would also be seen with LSTc-FAA. With H1N1 it even looks as if the lowest sugar concentration gives the highest virus signal and the signal decreases with increasing sugar concentration. It is not possible to say for sure because the replicates are missing and therefore no statement can be made about the measurement accuracy. Why were H1N1 and H5N1 not also measured in the negative control?

Looking forward to the answers

To address the reviewer's comments, we performed additional BLI experiments using H1N1 and H5N1 viruses against all glycan probes, including Mal3 and LNnT-FAA controls, across a broader glycan loading concentration from 0.1 to 266 nM (the original range was 11 - 532 nM). The results are now included as ***Supplementary Fig. 8****, with the corresponding source data provided in* ***Supplementary Data 6****. These findings are reproducible with respect to our previous experiments in* ***Fig. 7****, which demonstrated stronger interactions of the FAA sialyl glycan probes with the viruses compared to the PAA probes. As shown in panels d-e, when sugar loading is plotted on a log-mid scale to highlight the lower concentration range, the first three to four of the eight loading levels tested showed little binding for the sialylated FAA probes, whereas increased and stronger binding signals were observed at higher probe loading concentrations. The non-sialylated FAA probe controls included, as expected, displayed no detectable binding.*

Reviewer #3 (Remarks to the Author):

In this work, the authors develop tri-functional FAA probes that allow for versatile glycan array platforms. They assess binding activities of antibodies, lectins, and immune receptors using their systems, demonstrating different preferences of these various glycan binding proteins. While the work is interesting and provides an advance to the canonical glycan array platforms, the article is extremely disorganized and difficult to parse because of this. Additionally, a substantial amount of optimization, experimental design, and/or comparison to traditional glycan arrays are not addressed.

We appreciate the thoughtful comments by this reviewer and for recognizing that our work advances canonical glycan array platforms. We address below specific points below regarding organization and clarity and other specific points below under 'Notable issues'.

*We understand the reviewer's concern regarding the need to compare our array with conventional platforms. In this study, we aimed to test a range of GBPs to identify potential differences in binding patterns, as no systematic comparison of covalent versus non-covalent arrays has previously been performed. Our data obtained with antibodies and lectins of known glycan sequence specificity are overall in good agreement with published glycan array data from our lab and others. Additional observations (not previously reported) are summarized in **Supplementary Table 1** and discussed in the **Supplementary Discussion**. For GBPs that showed stronger binding on noncovalent arrays, such as Siglecs and PyV VP1 proteins, comparable "traditional glycan array" (e.g., CFG array) data are lacking - likely due to the weak or absent binding of these GBPs on covalent arrays. This notion is further supported by the results from the present study. Furthermore, natural glycolipids included as reference standards in the NGL-based noncovalent array (**Supplementary Data 2**) produced comparable results to the FAA–NGL probes, confirming the reliable performance of FAA glycan probes in microarray assays.*

Notable issues:

- The SI figures and associated text are nearly impossible to follow. The first SI figure addressed is #6 with no reference to 1-5 in the text. The next SI figure referenced is SI Fig 19, followed by SI Fig 1f, then SI 17. Most of the SI tables are never referenced.

We agree with the reviewer that the current figure numbering may have made the main text difficult to follow. Originally, the SI figures were arranged and numbered as part of the SI considered independently; for example, Figures S1–S5 appeared only in the SI Materials and Methods section, whereas Figure S6 was the first supplementary figure cited in the main text. To improve clarity and consistency, we have revised the numbering so that figures referenced in the main text are given priority over those appearing only in the SI. All SI tables were already cited within the Methods section of the main text.

- Also, why is there discussion/text in the SI? Was this because of word limits? The discussion contained in the SI would be useful to have in the main text.

We note, with respect, that inclusion of Supplementary Discussion is permitted under the Nature Communications publication guidelines. The decision to place parts of the focused discussion in the SI was made jointly by all co-authors, as detailed descriptions of differences in array binding results were considered too technical for the journal's broad readership and could disrupt the flow and focus of the main text. Since these discussions may still be of interest to researchers wishing to examine the fine glycan-binding specificities observed across different array platforms, we opted to present them in the SI for the benefit of readers specifically interested in these technical aspects.

- In Figure SI 6, the authors suggest that these methods demonstrate "similar peak shapes", which does not appear to be true. Further, they say that the FAA platform affords higher sensitivity, however, the ion counts are nearly impossible to read. These claims should be further substantiated.

*We have enlarged the y-axis labels of Figure S6 (now **Supplementary Fig.1**), which show fluorescent HPLC chromatograms (not MS spectra, as may have been misunderstood by the reviewer) of labelled N-glycan mixtures using three different tagging reagents: 2AB, AEAB, and FAA. Substantially stronger fluorescence signals from FAA-labelled glycans, compared to equivalent amounts of 2AB-labeled counterparts, clearly visible, support the higher sensitivity of the FAA linker. In response to the reviewer's comment, we added the phrase "for fluorescent detection" in line 103 and revised the **Supplementary Fig. 1** legend for clarity.*

Regarding "peak shape" comparisons, RNase B-derived oligomannose glycans exhibited highly similar chromatographic patterns across the three labels. In contrast, the more complex Fetuin N-glycan mixture showed some degree of peak overlap, potentially affecting overall chromatographic profiles. Notably, differences were observed between AEAB- and 2AB-labelled samples, whereas the FAA-labelled profile closely matched 2AB (the most widely used glycan profiling reagent).

- The authors refer to an "optimized linker" but do not discuss the optimization, some discussion on this would be appreciated.

*The term "optimized linker" was not used in the text to describe the FAA linker. Rather, the glycan labelling protocol using FAA was optimized in several aspects compared with conventional 2AB and AEAB labelling methods. Specifically, the reaction conditions were adjusted to pH 6 and 50 °C, replacing the conditions of 30% acetic acid at 60–65 °C typically used for 2AB or AEAB, thereby minimizing degradation of acid-labile glycans. In addition, the concentration of the labelling reagent was reduced from 0.35 M (commonly used for 2AB/AEAB) to 0.18 M, and the reaction time was extended from 2 hours to 4 hours to ensure complete labelling under milder conditions. Details of the optimized procedure are now provided in the **Supplementary Methods** (page 4).*

- At one point it is stated that 39 reducing glycans are being used, however, there are 44 in Figure 2. Why the discrepancy?

*The difference reflects an intentional expansion of the glycan set. As described in the **Methods** section ("Generation of multifunctional glycan probes"), the 39 sequence-defined glycans (**Supplementary Table 2**) were labelled with FAA. Additionally, five N-glycan probes were synthesized through enzymatic modification using FAA-linked probe 36 as the substrate.*

To further clarify this point, we have added the following sentence at the end of this paragraph (line 443): "All 44 probes were used for array generation."

- Does SPAAC need to be used for this work? Would copper click not work?

Both SPAAC and copper-assisted click chemistries are suitable for this purpose. We selected the SPAAC approach as it does not require additional reagents, facilitating high-throughput, in-solution conversion of FAA-linked glycans into NGLs, biotinlylated or fluorescent probes at the nanomole scale with quantitative yield. The SPAAC approach for NGL preparation was also described in our recent publication (Nat Commun. 2024. doi: 10.1038/s41467-024-51081-7).

- Fig 3c - all of these spots look very similar, is that expected? How do these measurements compare to a typical glycan array? For this experiment, it be worthwhile to investigate what the limit of detection is of their method, especially compared to traditional arrays. Also, the authors determine that 100 uM is "optimal" but it is unclear why this is the case.

In Fig. 3c, the spot sizes are similar because equal volumes of printing solution were dispensed. However, the fluorescence intensities vary as shown in Fig.3d, following a trend of increased signal with higher printing concentrations. This experiment was designed to assess how printing concentration influences glycan immobilization on covalent microarrays - an analysis not feasible with conventional arrays without protein binding. Using FAA probes and on-array SPAAC click chemistry we were able to perform such evaluation. As the glycan probe concentration-dependent effect was modest, a concentration of 100 µM (commonly used in covalent glycan arrays) was selected for subsequent.

- Man9-GN2 - this nomenclature is unusual, please standardize glycan structures throughout.

Thank you for the comment and we have now changed Man9-GN2 into $Man_9GlcNAc_2$.

- At one point the authors reference "data not shown", please include this data.

“Further glycan microarray analysis of H1N1 binding also detected interactions with LSTd, though markedly weaker than with LSTc (data not shown), supporting these observations.” We did not include the microarray data on influenza viruses, as these were obtained using a new sialyl glycan array reserved for a separate publication. However, we have now added ***Supplementary Fig. 9*** *to present the relevant microarray results with selected glycan probes.*

- The authors do not address that a major drawback of all glycan array-like platforms is that often glycan binding proteins are also reliant on the presence of a protein backbone.

We would like to point out, with respect, that to our knowledge most GBPs, including lectins and carbohydrate-specific antibodies, recognize glycan epitopes rather than the underlying carrier molecules such as protein or lipid backbones. Their binding is primarily determined by the specific glycan structure (e.g., monosaccharide composition, linkage, and branching). However, the peptide or lipid component of the carrier can significantly influence glycan presentation (e.g., through multivalency or spatial orientation), thereby affecting the overall avidity of the interaction. Most glycan array systems, including this study, focus on glycan-mediated interactions. For binding systems known to recognize peptide backbones, glycopeptide arrays can be used (e.g., cancer-specific antibodies recognizing mucin glycoepitopes). This is not within the scope of the present study.

- It is unclear to me what the benefits are of using a covalent vs. non-covalent system.

We agree that this is an important point to address. Accordingly, we have revised the Discussion section and added the following text to incorporate this aspect:

The experience gained from the present study indicates that, for evaluating glycan binding by GBPs with lower affinities, where multivalent engagement is crucial for detectable binding, the noncovalent NGL-based array platform may offer advantages. However, covalent arrays on glass surfaces can be superior for analyzing the binding of fluorescently labelled proteins or microbes, such as whole bacterial cells, owing to their lower background compared with the nitrocellulose-coated surfaces used in noncovalent arrays.

Thus, the comparisons presented here are not intended to assess the superiority of one platform over another, but rather to highlight their complementary nature. A lack of binding in one array platform does not necessarily indicate that a protein is unable to recognize glycans. Our aim is to draw attention to the importance of glycan presentation, minimize the likelihood of “false negative” results in glycan array analyses, and promote flexibility across different technological platforms to enhance confidence in the knowledge gained on glycan-mediated interactions.

When determining the glycan specificity of a novel GBP, it is advantageous to collect data from both covalent and noncovalent array platforms wherever possible. A good example is the characterization of the binding specificities of a novel melon lectin CMA1, achieved by integrating datasets from the CFG covalent array and our NGL-based non-covalent array.
